# Supplementary material for: Direct visualization of HIV-1 core nuclear import and its interplay with the nuclear pore
Source: EMBO Rep. 2025 Aug 29;26(21):5133–53. doi: 10.1038/s44319-025-00567-6 (PMC12592377; doi:10.1038/s44319-025-00567-6)
Supplement: Supplementary file 1 — Table EV1 [file 44319_2025_567_MOESM1_ESM.docx]

**Table EV1|** Shape distribution of HIV-1 VLP cores in multiple states.

|  | Unassociated | Approaching | Docking | Traversing | Imported |
| --- | --- | --- | --- | --- | --- |
| Cone-shaped | 17 | 31 | 50 | 33 | 2 |
| tube-shaped | 40 | 62 | 72 | 117 | 43 |
| Total | 57 | 93 | 122 | 150 | 45 |
