# Supplementary material for: Direct visualization of HIV-1 core nuclear import and its interplay with the nuclear pore
Source: EMBO Rep. 2025 Aug 29;26(21):5133–53. doi: 10.1038/s44319-025-00567-6 (PMC12592377; doi:10.1038/s44319-025-00567-6)
Supplement: Supplementary file 2 — Table EV2 [file 44319_2025_567_MOESM2_ESM.docx]

**Table EV2|** Cryo-FIB lamella preparation

| **Method** | Correlative milling | Correlative milling | Correlative lift-out |
| --- | --- | --- | --- |
| Microscope | Plasma FIB Arctis | Conventional FIB Aquilos 2 | Conventional FIB Aquilos 2 |
| Voltage (keV) | 30 | 30 | 30 |
| Ion beam source | Argon | Gallium | Gallium |
| Sputtering coating prior to milling (seconds) | 12 | No | 12 |
| GIS coating time (second) | 50 | 30 | 30 |
| Bulk milling current | N/A | N/A | 5-7 nA |
| Milling current | 0.74-2 nA | 0.1-0.5 nA | 0.1-0.5 nA |
| Polishing current | 60 pA | 30 pA | 30 pA |
| Sputtering coating post polishing (seconds) | No | No | No |
| Fluorescence microscope | iFLM (100 ×) | METEOR (50 ×) | iFLM (20 ×) |
| Number of lamellae | 35 | 85 | 5 |
