# Supplementary material for: Direct visualization of HIV-1 core nuclear import and its interplay with the nuclear pore
Source: EMBO Rep. 2025 Aug 29;26(21):5133–53. doi: 10.1038/s44319-025-00567-6 (PMC12592377; doi:10.1038/s44319-025-00567-6)
Supplement: Supplementary file 3 — Table EV3 [file 44319_2025_567_MOESM3_ESM.docx]

| **Sample** | Arctis lamellae | Aquilos 2 lamellae | Aquilos 2  Lift-out lamellae | HIV-1 virions | HIV-1 VLPs |
| --- | --- | --- | --- | --- | --- |
| Microscope | FEI Titan Krios G3 | FEI Titan Krios G3 | FEI Titan Krios G4 | FEI Titan Krios G2 | FEI Titan Krios G2 |
| Voltage (keV) | 300 | 300 | 300 | 300 | 300 |
| Detector | Falcon 4i | Falcon 4i | Falcon 4i | Gatan K3 | Gatan K3 |
| Energy-filter | Selectris X | Selectris X | Selectris X | Gatan BioQuantum | Gatan BioQuantum |
| Slit width (eV) | 10 | 10 | 10 | 20 | 20 |
| Super-resolution mode | No | No | No | No | Yes |
| Physical pixel size (Å/pixel) | 1.903 | 1.94 | 2 | 1.34 | 0.831 |
| Defocus range (µm) | -3 to -5, increment 0.3 | -3 to -5, increment 0.3 | -2 to -3, increment 0.3 | -1.5 to -3, increment 0.3 | -3 to -4, increment 0.3 |
| Acquisition scheme | Dose-Symmetric,  -52⁰/52⁰, 2⁰ step, group 2 | Dose-Symmetric,  -52⁰/52⁰, 2⁰ step, group 2 | Dose-Symmetric,  -60⁰/60⁰, 2⁰ step, group 2 | Dose-Symmetric,  -60⁰/60⁰, 3⁰ step, group 3 | Single-shot micrographs |
| Total dose (electrons/Å^2^) | 159 | 159 | 152.5 | 123 | 22 |
| Number of frames | 10 | 10 | EER | 10 | 59 |
| Number of lamellae | 35 | 85 | 5 | N/A | N/A |
| Number of tomograms/micrographs | 97 | 167 | 5 | 58 | 2000 |
|  | | | | | |
| **Subtomogram averaging** | CA hexamers of outside HIV-1 VLP cores | CA hexamers of traversing HIV-1 VLP cores | CA hexamers of imported HIV-1 VLP cores | Cytoplasmic ring of NPC | Inner ring of NPC |
| Number of subtomograms | 1222 | 1085 | 1106 | 332 | 332 |
| Resolution at 0.143 FSC cut-off (Å) | 12 | 13.5 | 13.5 | 32 | 38 |

**Table EV3|** Cryo-ET data collection and structure determination of CA hexamers
