# Supplementary material for: Direct visualization of HIV-1 core nuclear import and its interplay with the nuclear pore
Source: EMBO Rep. 2025 Aug 29;26(21):5133–53. doi: 10.1038/s44319-025-00567-6 (PMC12592377; doi:10.1038/s44319-025-00567-6)
Supplement: Supplementary file 5 — Movie EV1 [file 44319_2025_567_MOESM5_ESM.zip › Movie EV1-legend.docx]

**Movie EV1** | A video showcasing the tomogram and segmented volume of two HIV-1 VLP cores queuing up on the NPC.
