# Supplementary figures and images for: Direct visualization of HIV-1 core nuclear import and its interplay with the nuclear pore (part 2 of 3)
Source: EMBO Rep. 2025 Aug 29;26(21):5133–53. doi: 10.1038/s44319-025-00567-6 (PMC12592377; doi:10.1038/s44319-025-00567-6)

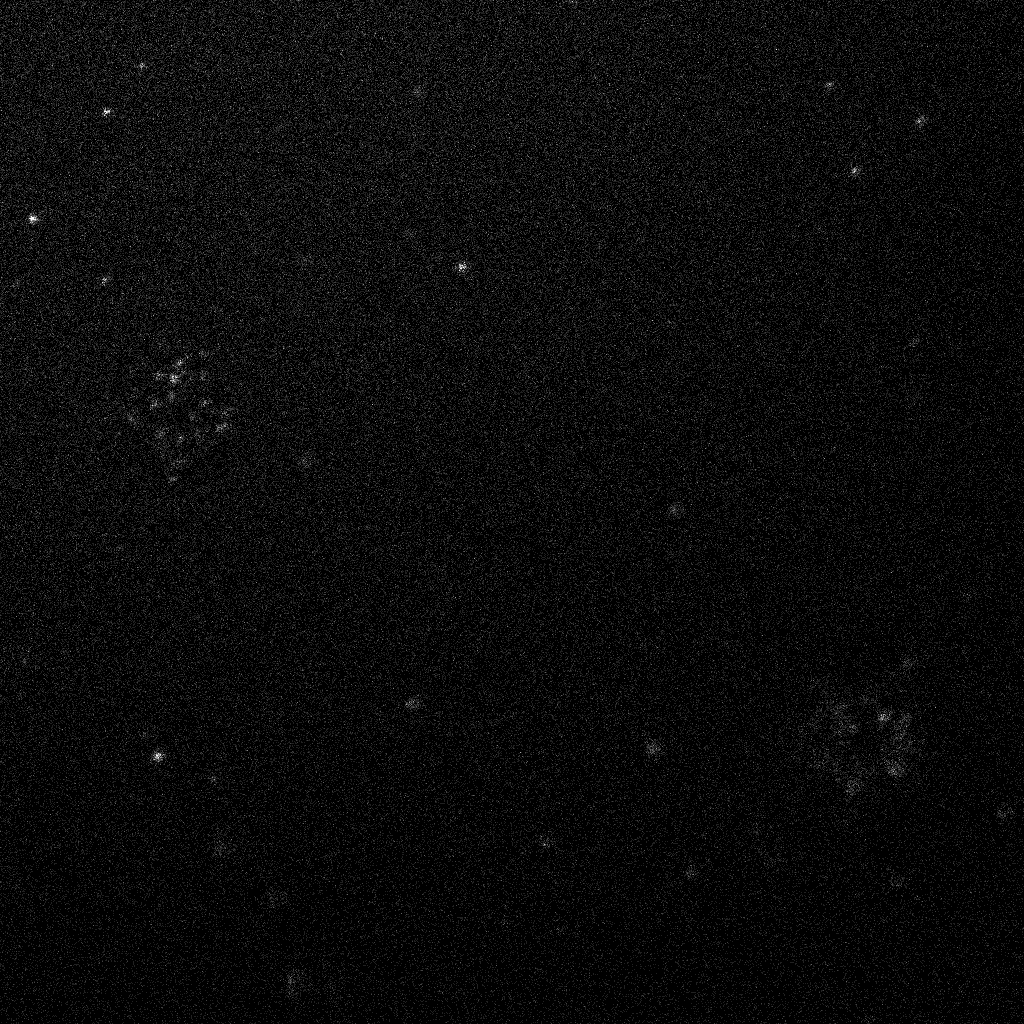

Supplement: Supplementary file 8 — Source data Fig. 1 [file 44319_2025_567_MOESM8_ESM.zip › Fig1/1D/Counted_nuclei/Mechanical/Nuc_15+16+17/Nuc_15+16+17_z00_RAW_ch00.tif]

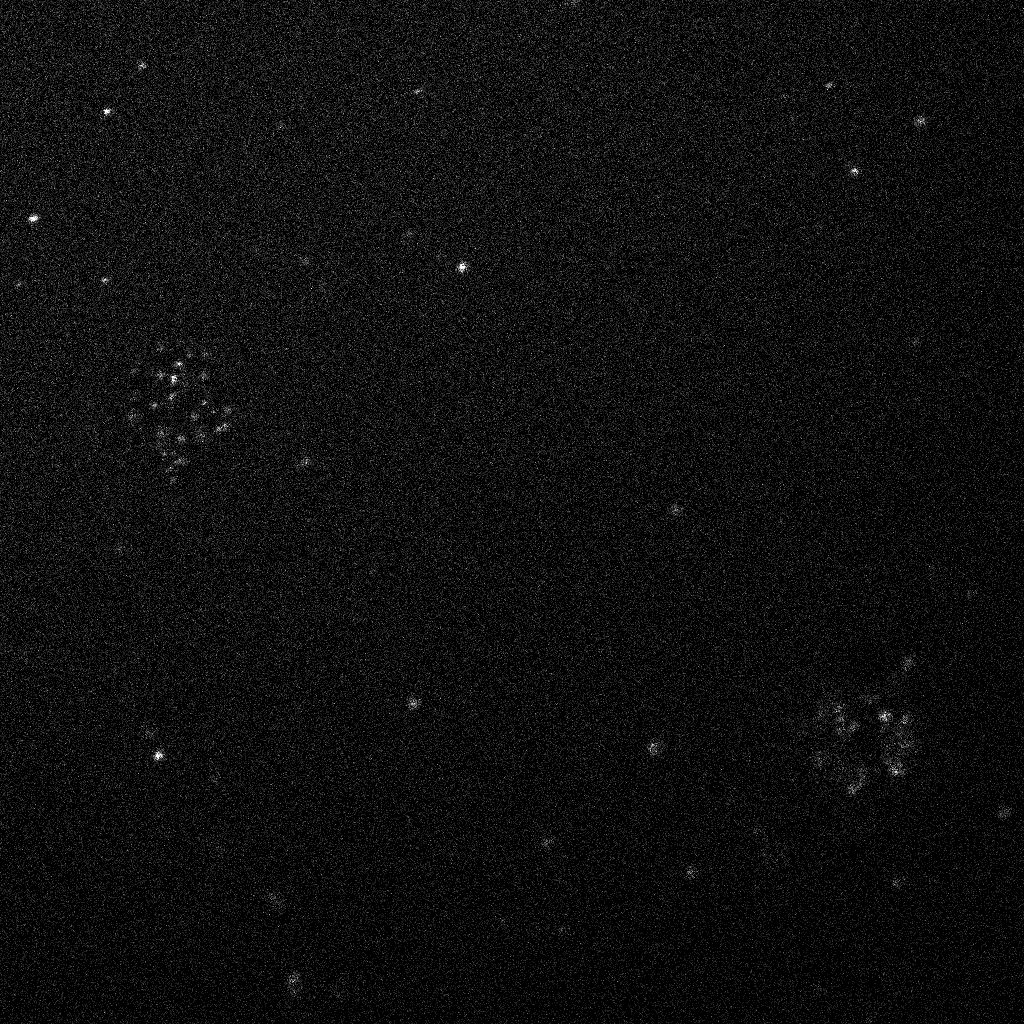

Supplement: Supplementary file 8 — Source data Fig. 1 [file 44319_2025_567_MOESM8_ESM.zip › Fig1/1D/Counted_nuclei/Mechanical/Nuc_15+16+17/Nuc_15+16+17_z01_RAW_ch00.tif]

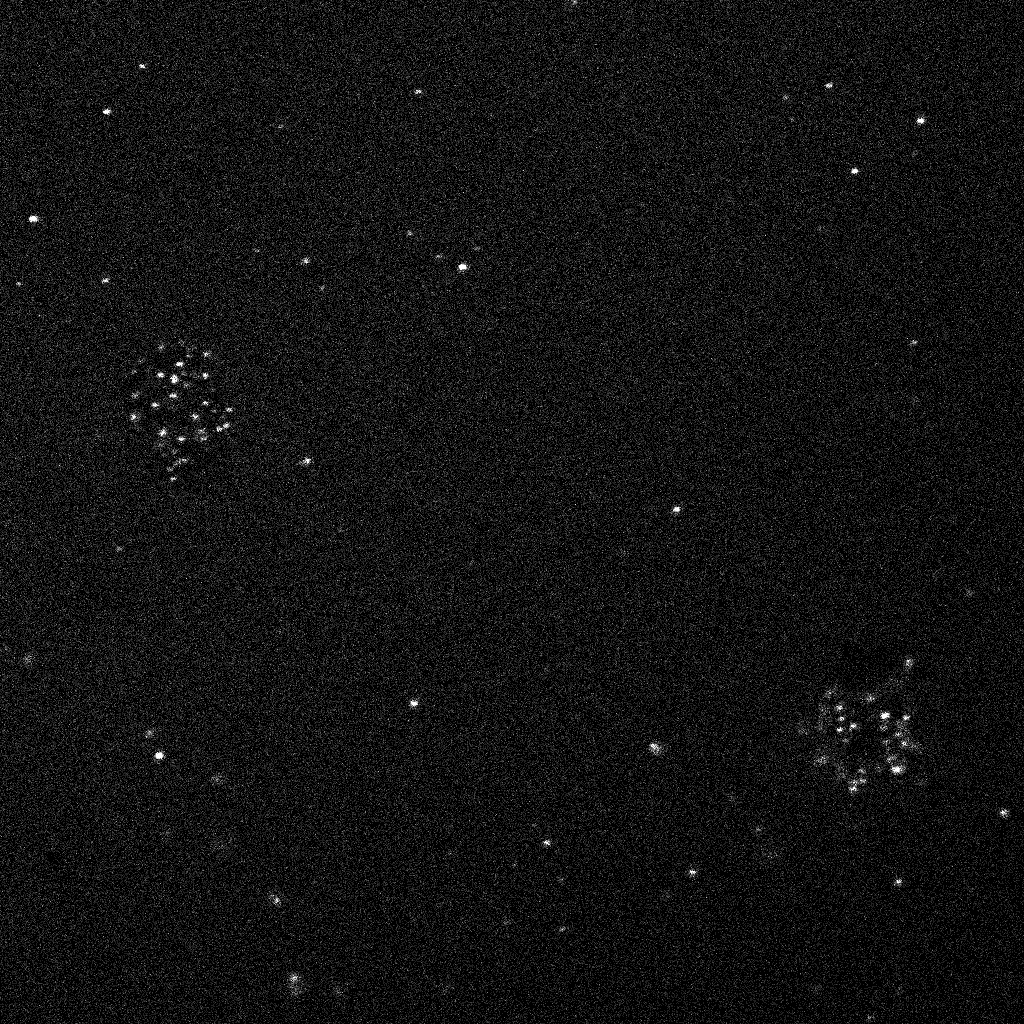

Supplement: Supplementary file 8 — Source data Fig. 1 [file 44319_2025_567_MOESM8_ESM.zip › Fig1/1D/Counted_nuclei/Mechanical/Nuc_15+16+17/Nuc_15+16+17_z02_RAW_ch00.tif]

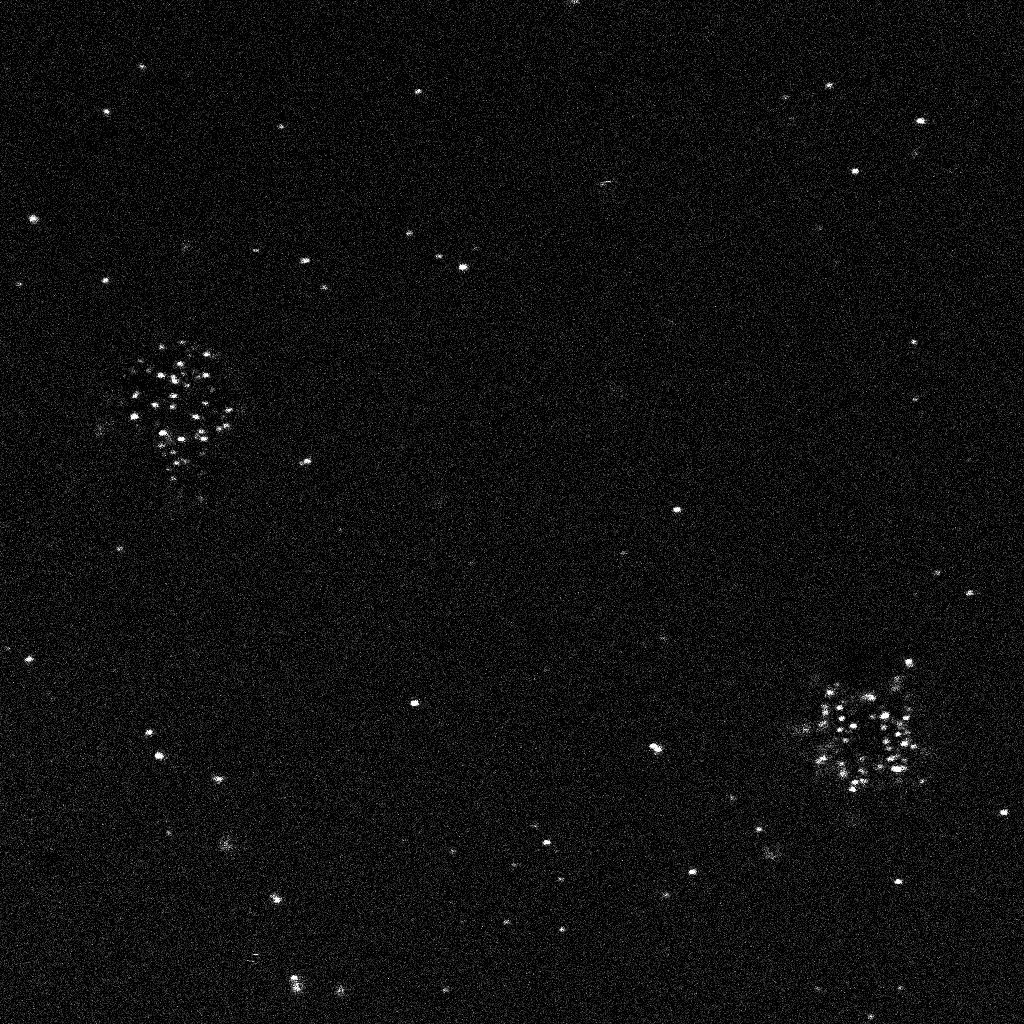

Supplement: Supplementary file 8 — Source data Fig. 1 [file 44319_2025_567_MOESM8_ESM.zip › Fig1/1D/Counted_nuclei/Mechanical/Nuc_15+16+17/Nuc_15+16+17_z03_RAW_ch00.tif]

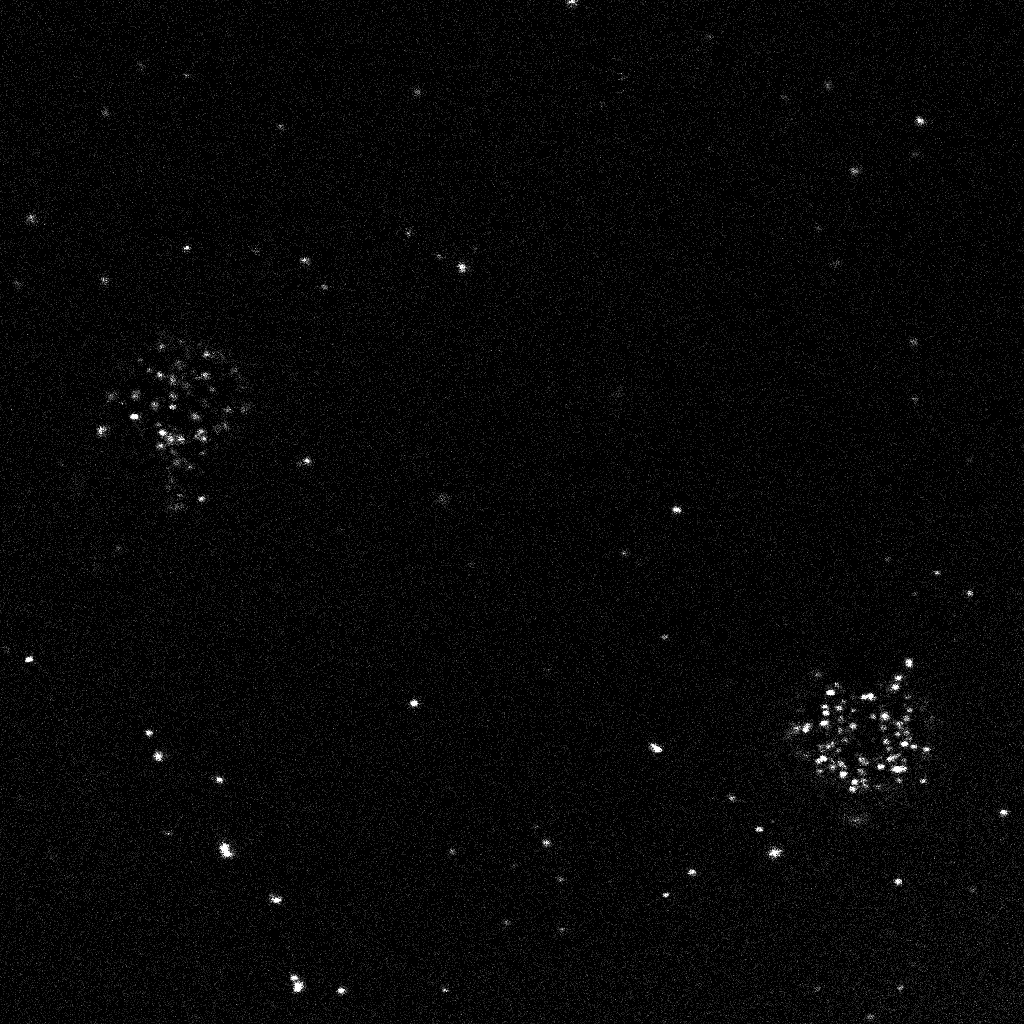

Supplement: Supplementary file 8 — Source data Fig. 1 [file 44319_2025_567_MOESM8_ESM.zip › Fig1/1D/Counted_nuclei/Mechanical/Nuc_15+16+17/Nuc_15+16+17_z04_RAW_ch00.tif]

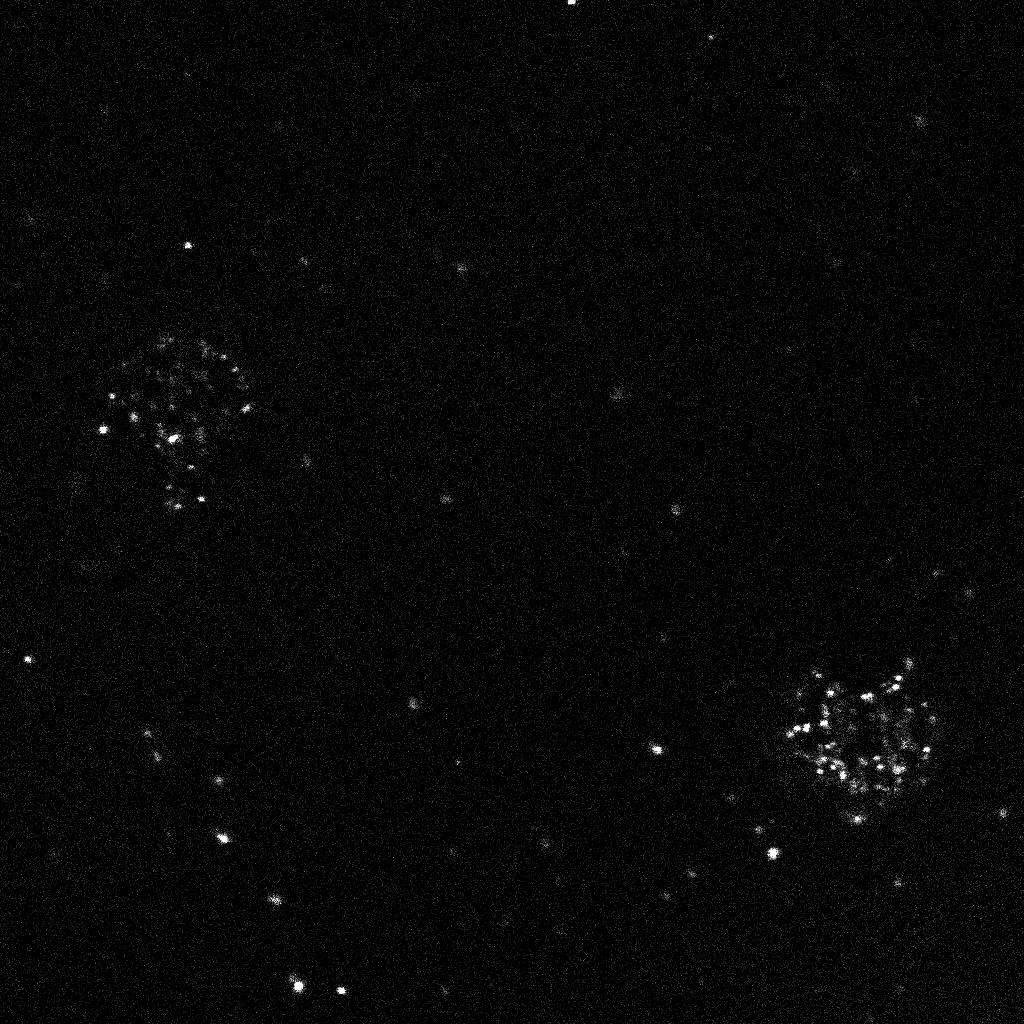

Supplement: Supplementary file 8 — Source data Fig. 1 [file 44319_2025_567_MOESM8_ESM.zip › Fig1/1D/Counted_nuclei/Mechanical/Nuc_15+16+17/Nuc_15+16+17_z05_RAW_ch00.tif]

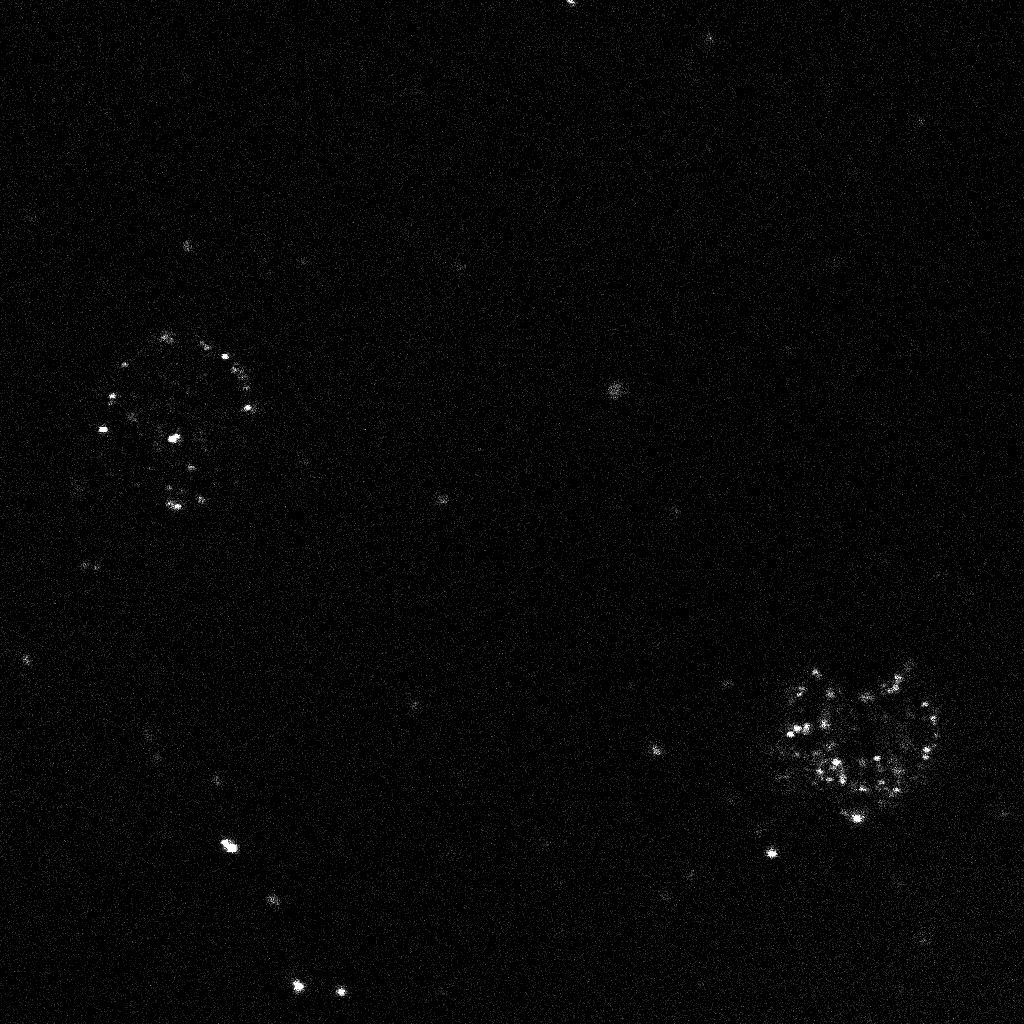

Supplement: Supplementary file 8 — Source data Fig. 1 [file 44319_2025_567_MOESM8_ESM.zip › Fig1/1D/Counted_nuclei/Mechanical/Nuc_15+16+17/Nuc_15+16+17_z06_RAW_ch00.tif]

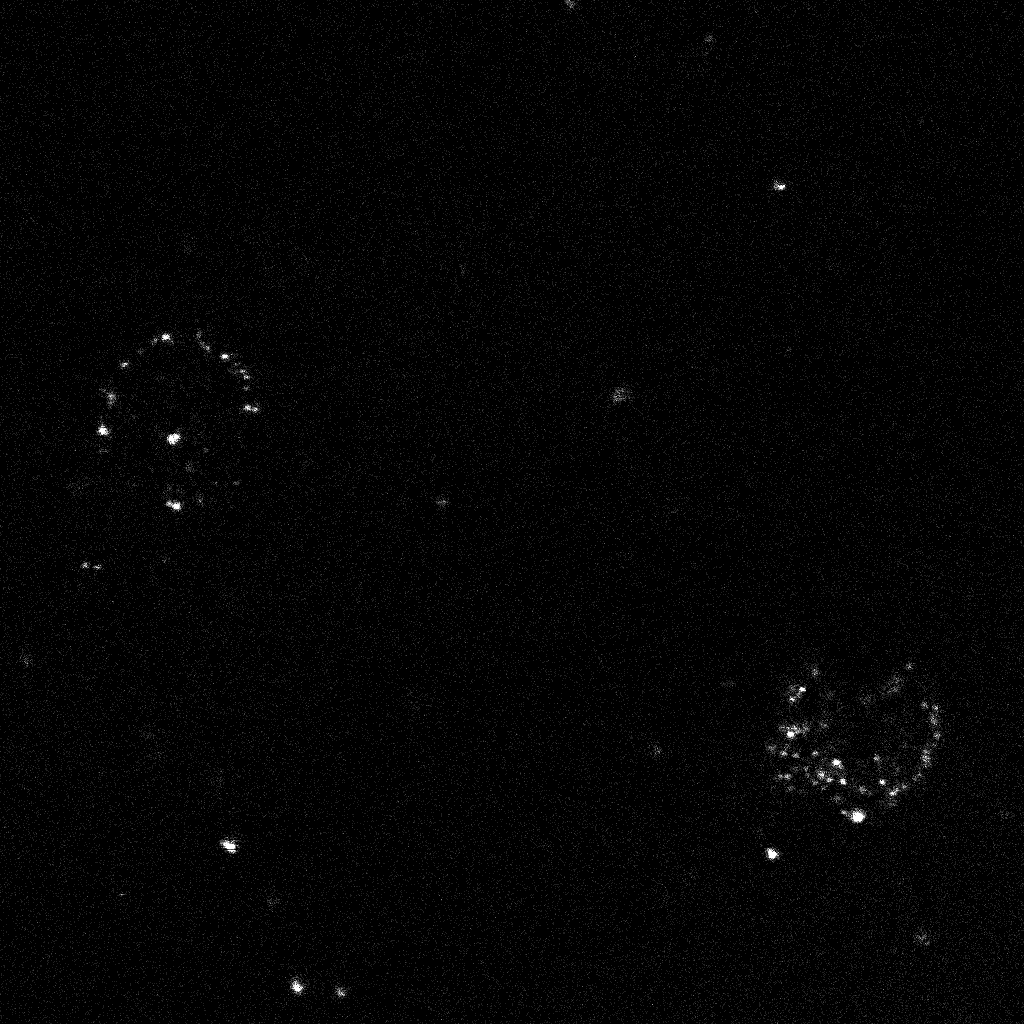

Supplement: Supplementary file 8 — Source data Fig. 1 [file 44319_2025_567_MOESM8_ESM.zip › Fig1/1D/Counted_nuclei/Mechanical/Nuc_15+16+17/Nuc_15+16+17_z07_RAW_ch00.tif]

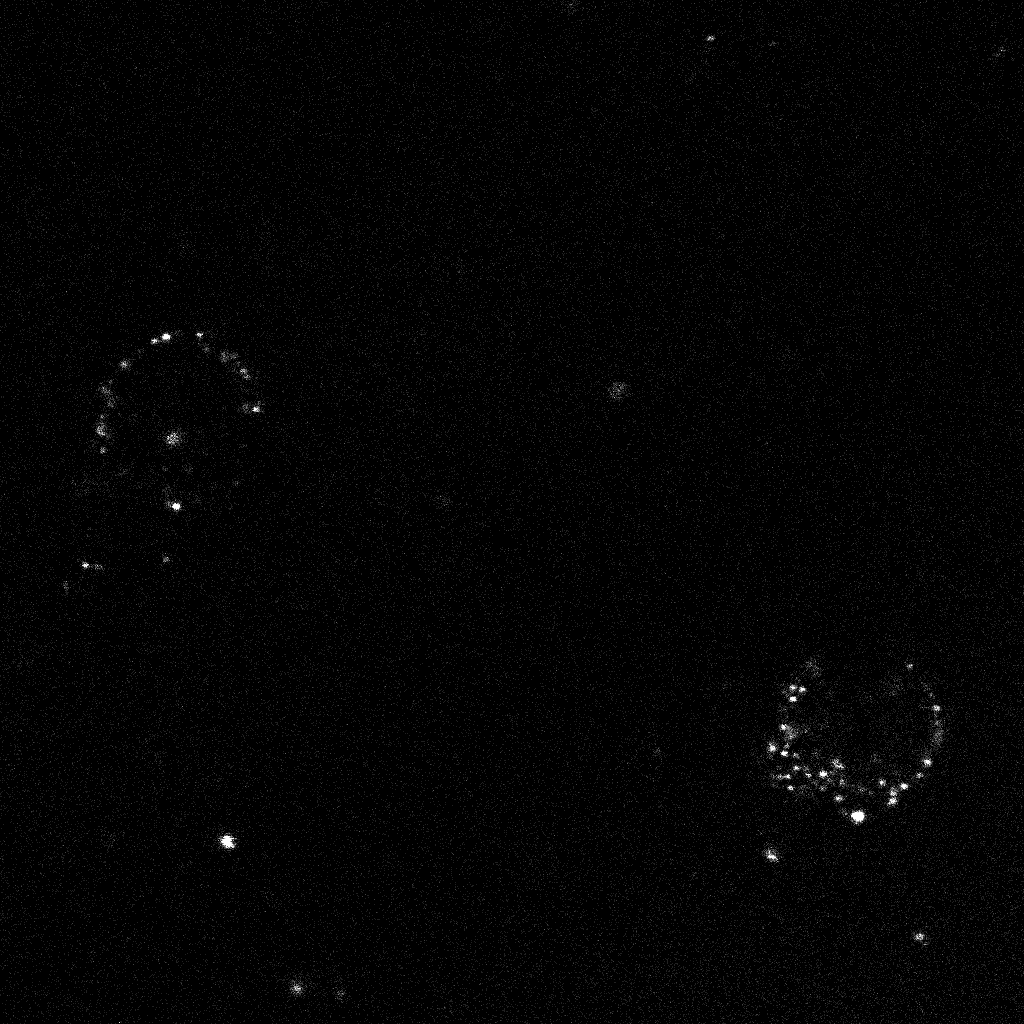

Supplement: Supplementary file 8 — Source data Fig. 1 [file 44319_2025_567_MOESM8_ESM.zip › Fig1/1D/Counted_nuclei/Mechanical/Nuc_15+16+17/Nuc_15+16+17_z08_RAW_ch00.tif]

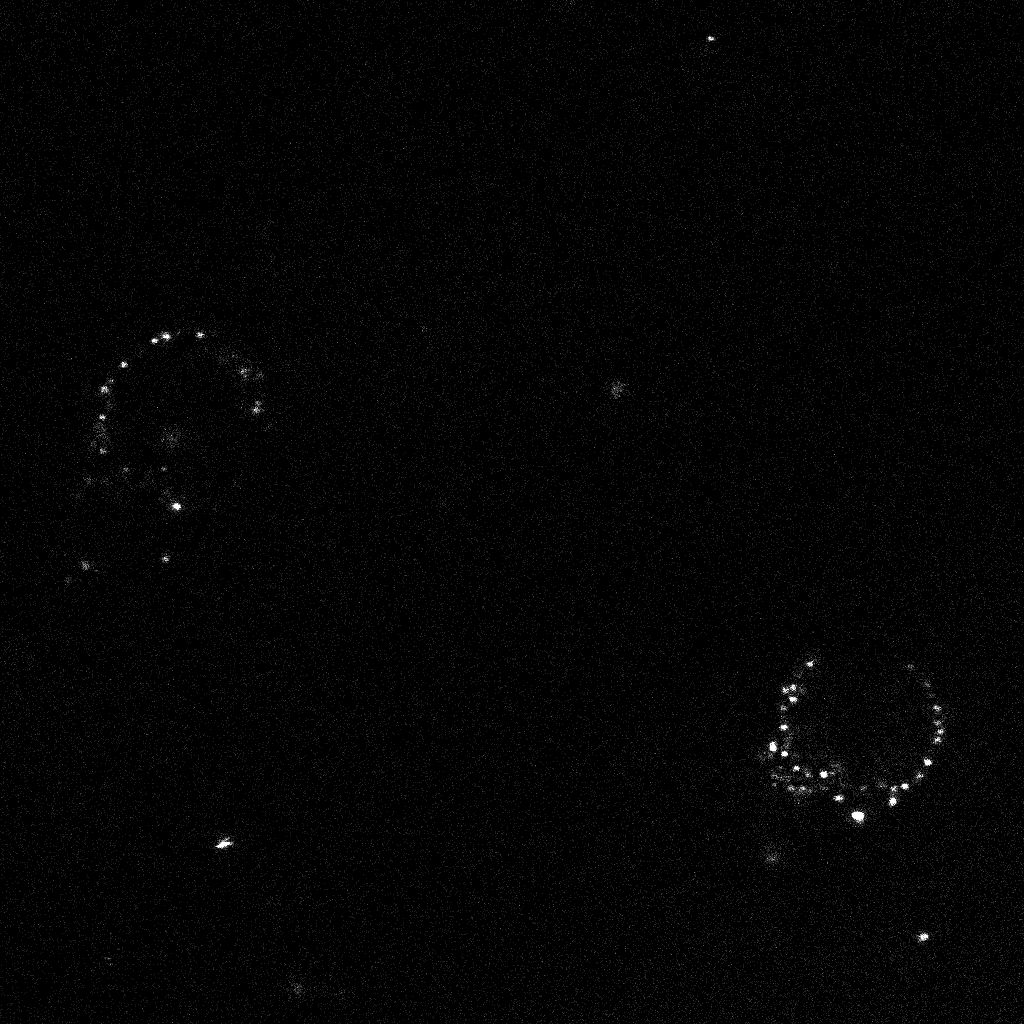

Supplement: Supplementary file 8 — Source data Fig. 1 [file 44319_2025_567_MOESM8_ESM.zip › Fig1/1D/Counted_nuclei/Mechanical/Nuc_15+16+17/Nuc_15+16+17_z09_RAW_ch00.tif]

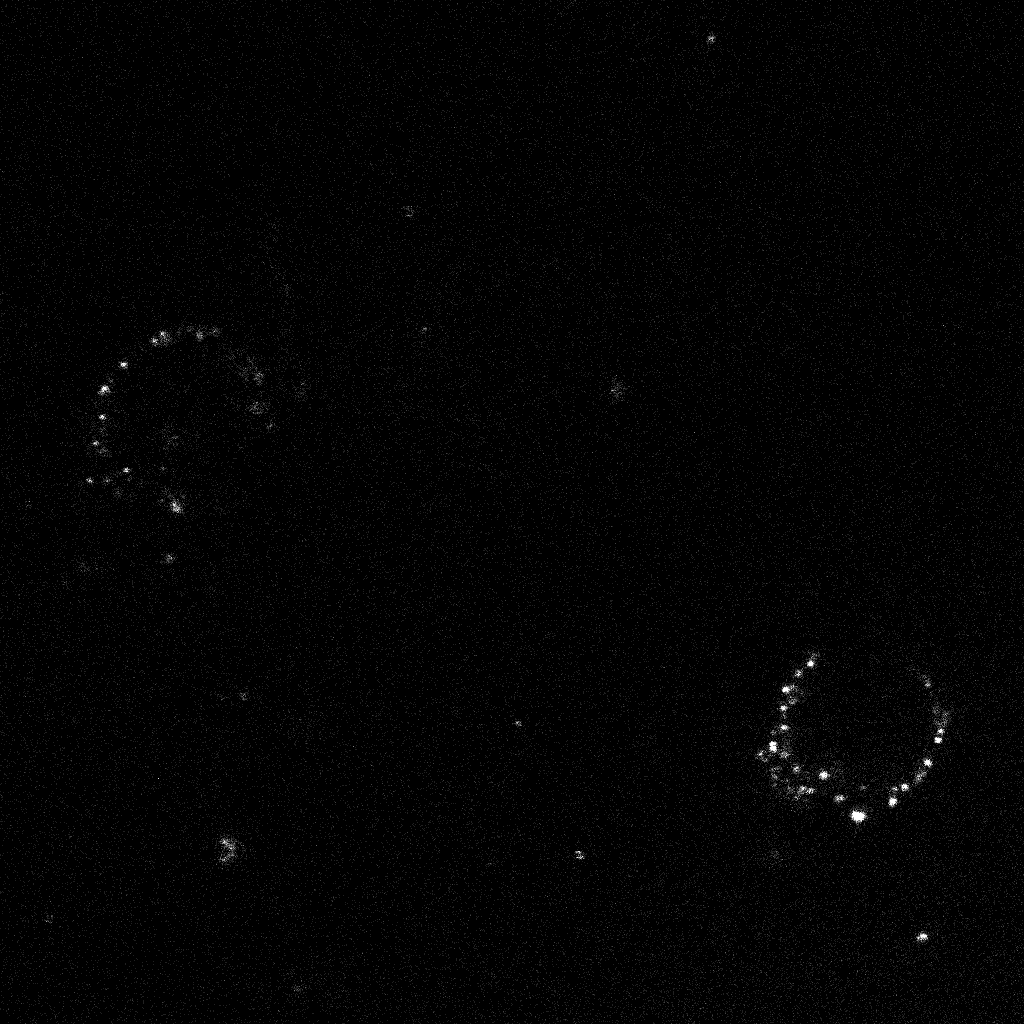

Supplement: Supplementary file 8 — Source data Fig. 1 [file 44319_2025_567_MOESM8_ESM.zip › Fig1/1D/Counted_nuclei/Mechanical/Nuc_15+16+17/Nuc_15+16+17_z10_RAW_ch00.tif]

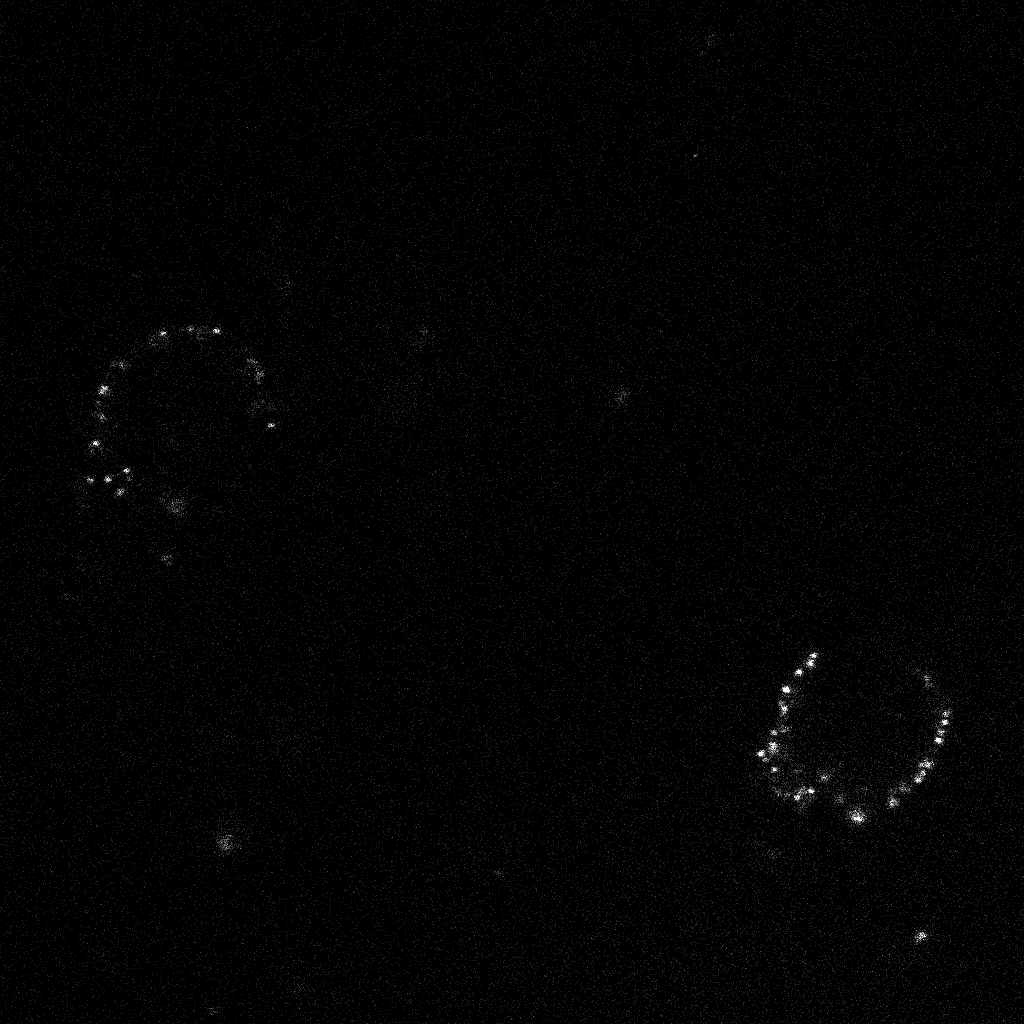

Supplement: Supplementary file 8 — Source data Fig. 1 [file 44319_2025_567_MOESM8_ESM.zip › Fig1/1D/Counted_nuclei/Mechanical/Nuc_15+16+17/Nuc_15+16+17_z11_RAW_ch00.tif]

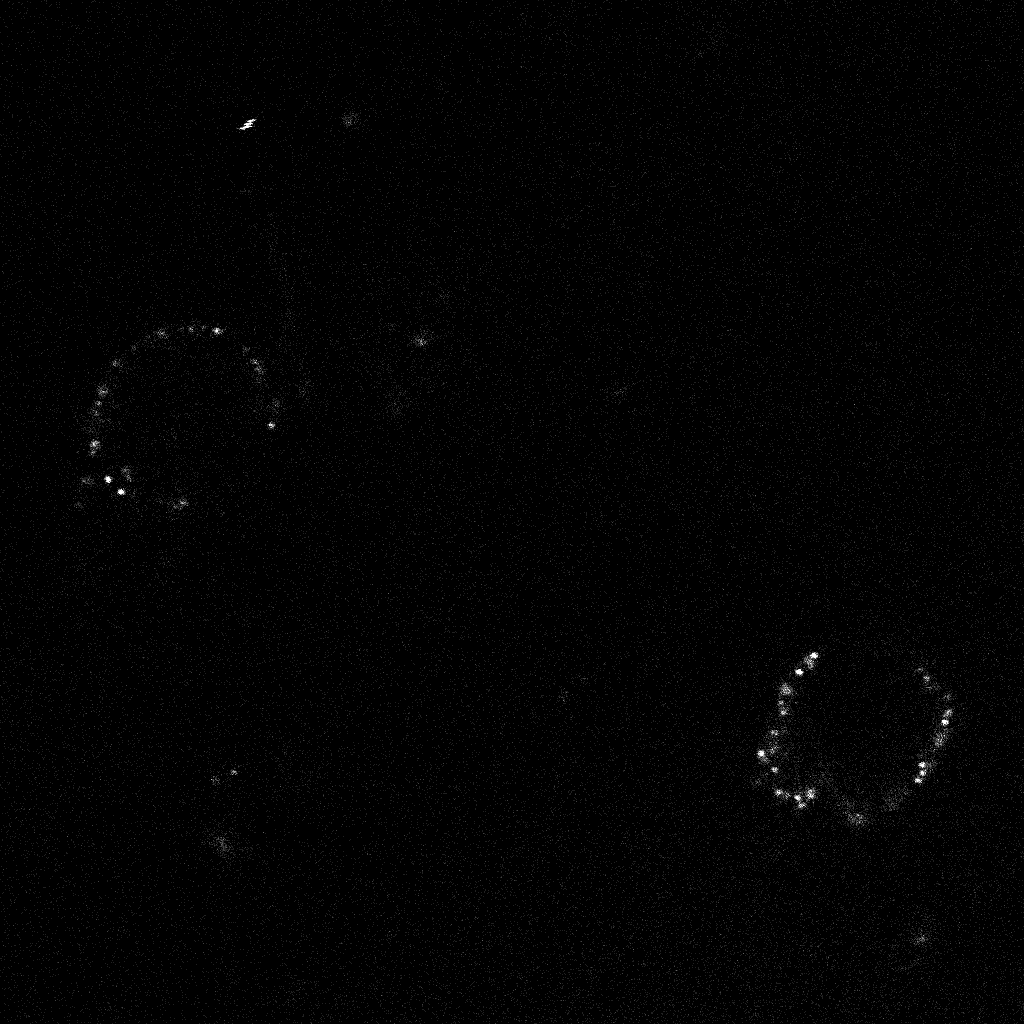

Supplement: Supplementary file 8 — Source data Fig. 1 [file 44319_2025_567_MOESM8_ESM.zip › Fig1/1D/Counted_nuclei/Mechanical/Nuc_15+16+17/Nuc_15+16+17_z12_RAW_ch00.tif]

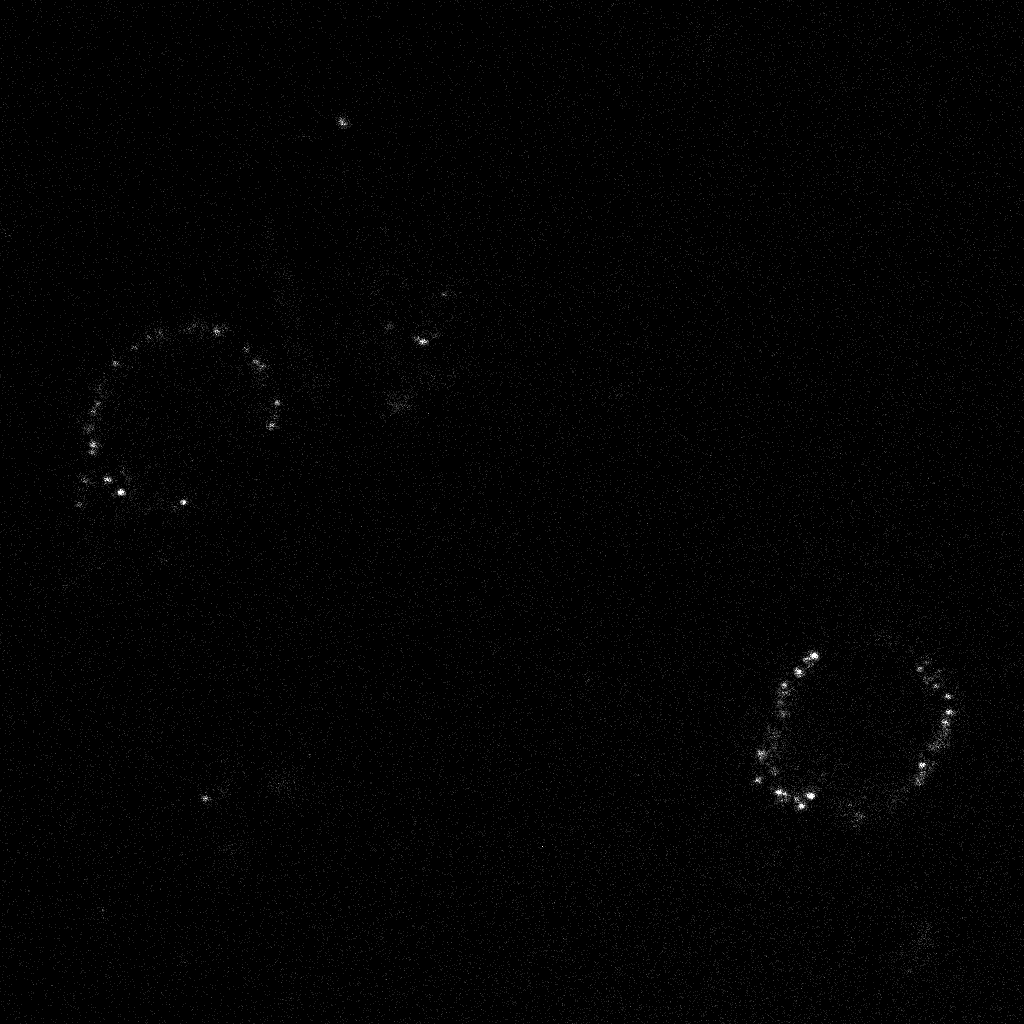

Supplement: Supplementary file 8 — Source data Fig. 1 [file 44319_2025_567_MOESM8_ESM.zip › Fig1/1D/Counted_nuclei/Mechanical/Nuc_15+16+17/Nuc_15+16+17_z13_RAW_ch00.tif]

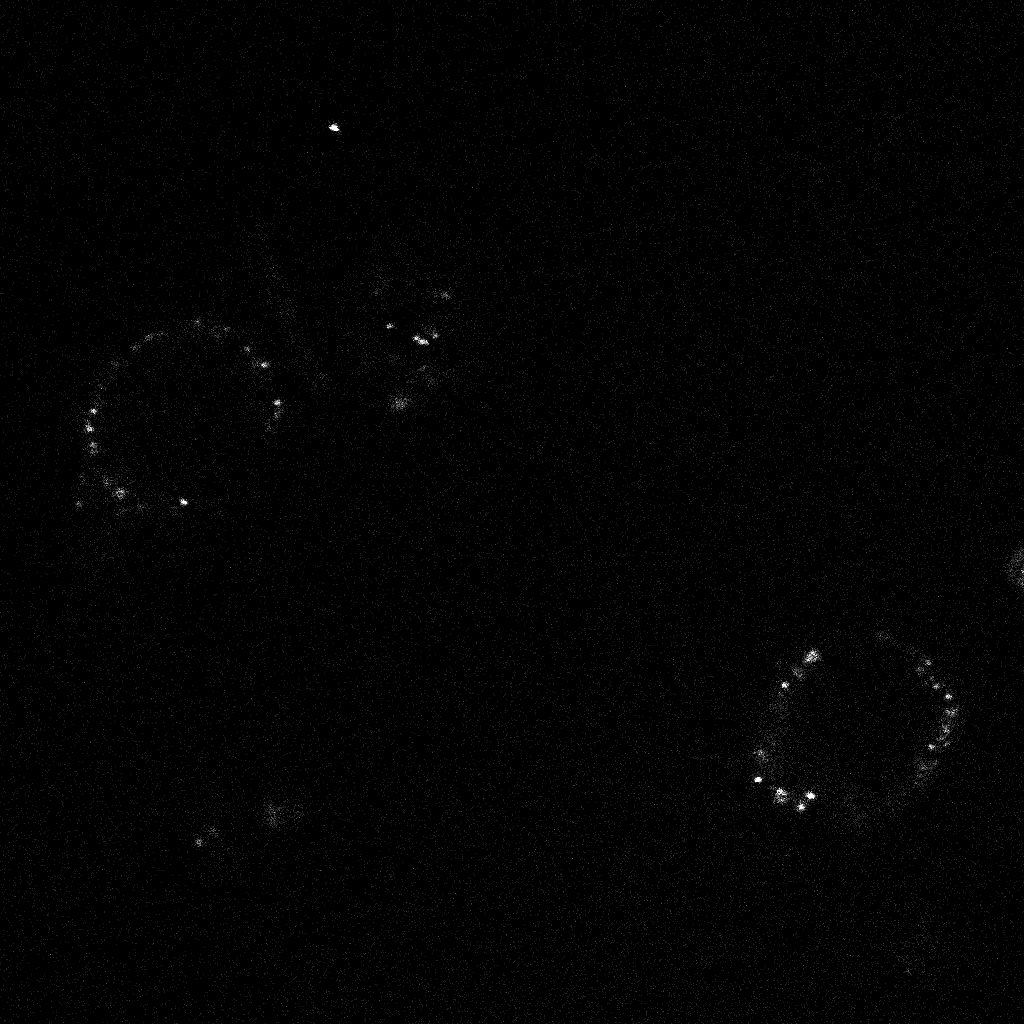

Supplement: Supplementary file 8 — Source data Fig. 1 [file 44319_2025_567_MOESM8_ESM.zip › Fig1/1D/Counted_nuclei/Mechanical/Nuc_15+16+17/Nuc_15+16+17_z14_RAW_ch00.tif]

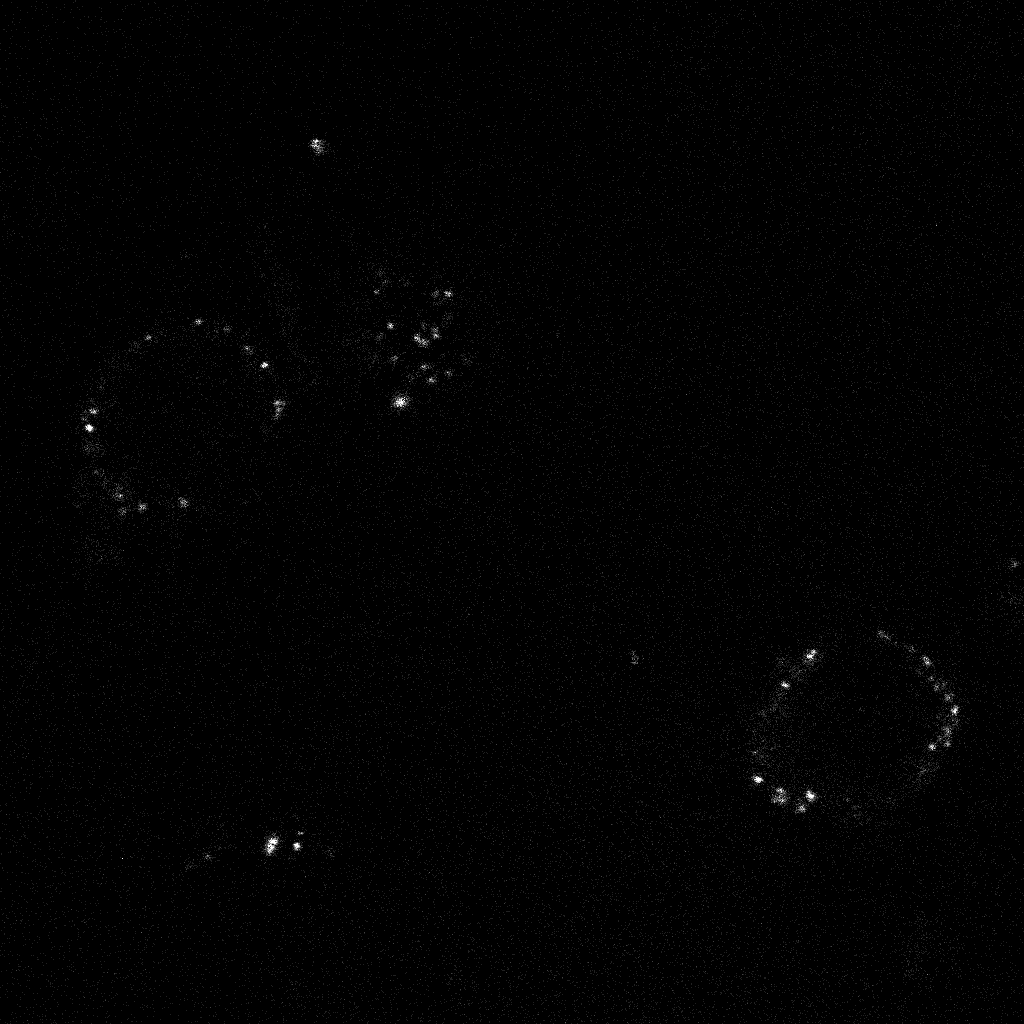

Supplement: Supplementary file 8 — Source data Fig. 1 [file 44319_2025_567_MOESM8_ESM.zip › Fig1/1D/Counted_nuclei/Mechanical/Nuc_15+16+17/Nuc_15+16+17_z15_RAW_ch00.tif]

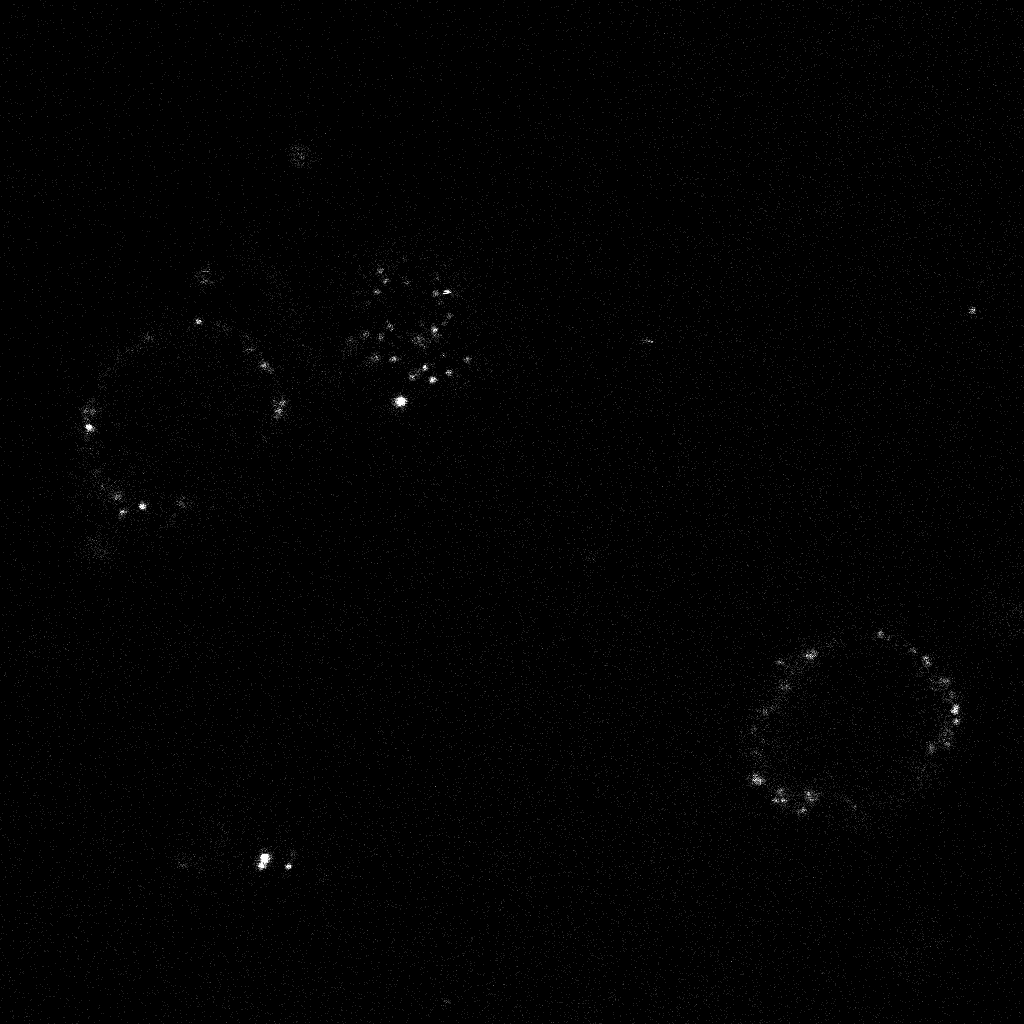

Supplement: Supplementary file 8 — Source data Fig. 1 [file 44319_2025_567_MOESM8_ESM.zip › Fig1/1D/Counted_nuclei/Mechanical/Nuc_15+16+17/Nuc_15+16+17_z16_RAW_ch00.tif]

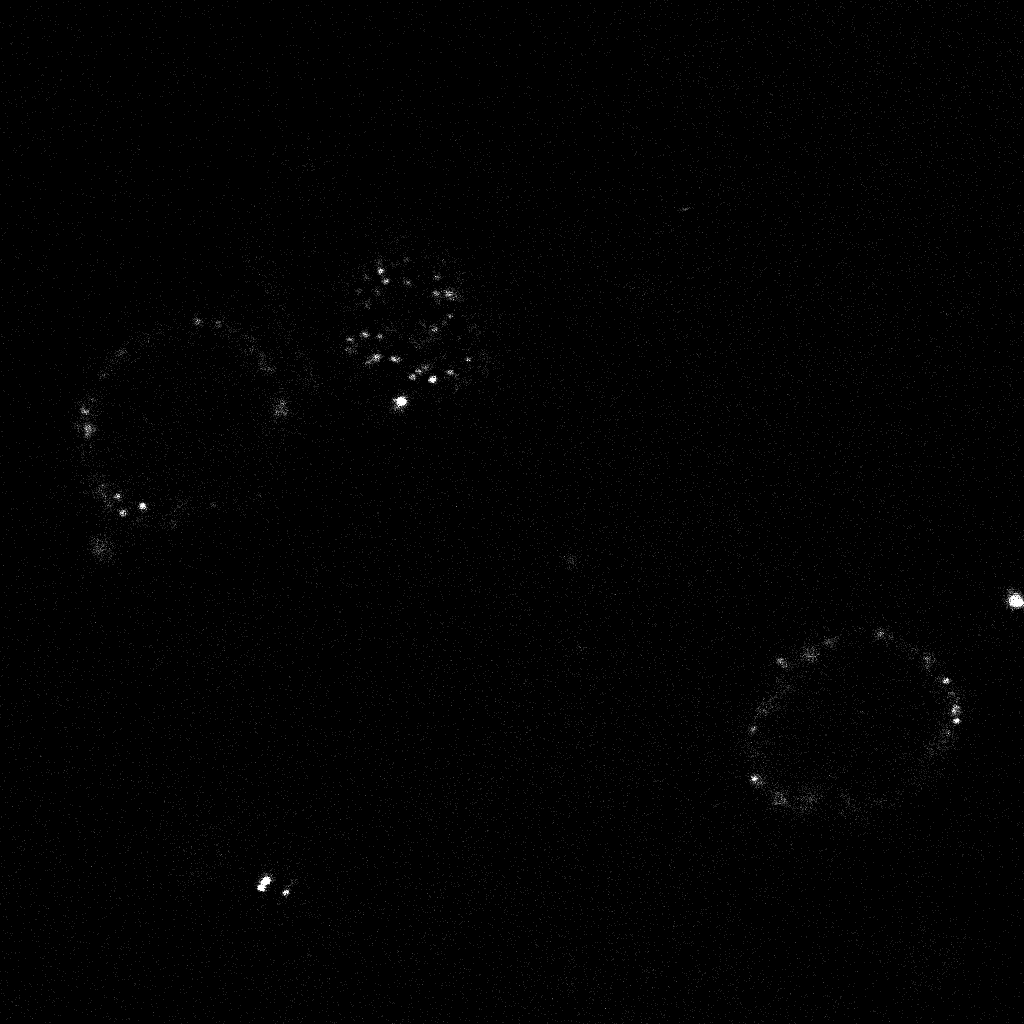

Supplement: Supplementary file 8 — Source data Fig. 1 [file 44319_2025_567_MOESM8_ESM.zip › Fig1/1D/Counted_nuclei/Mechanical/Nuc_15+16+17/Nuc_15+16+17_z17_RAW_ch00.tif]

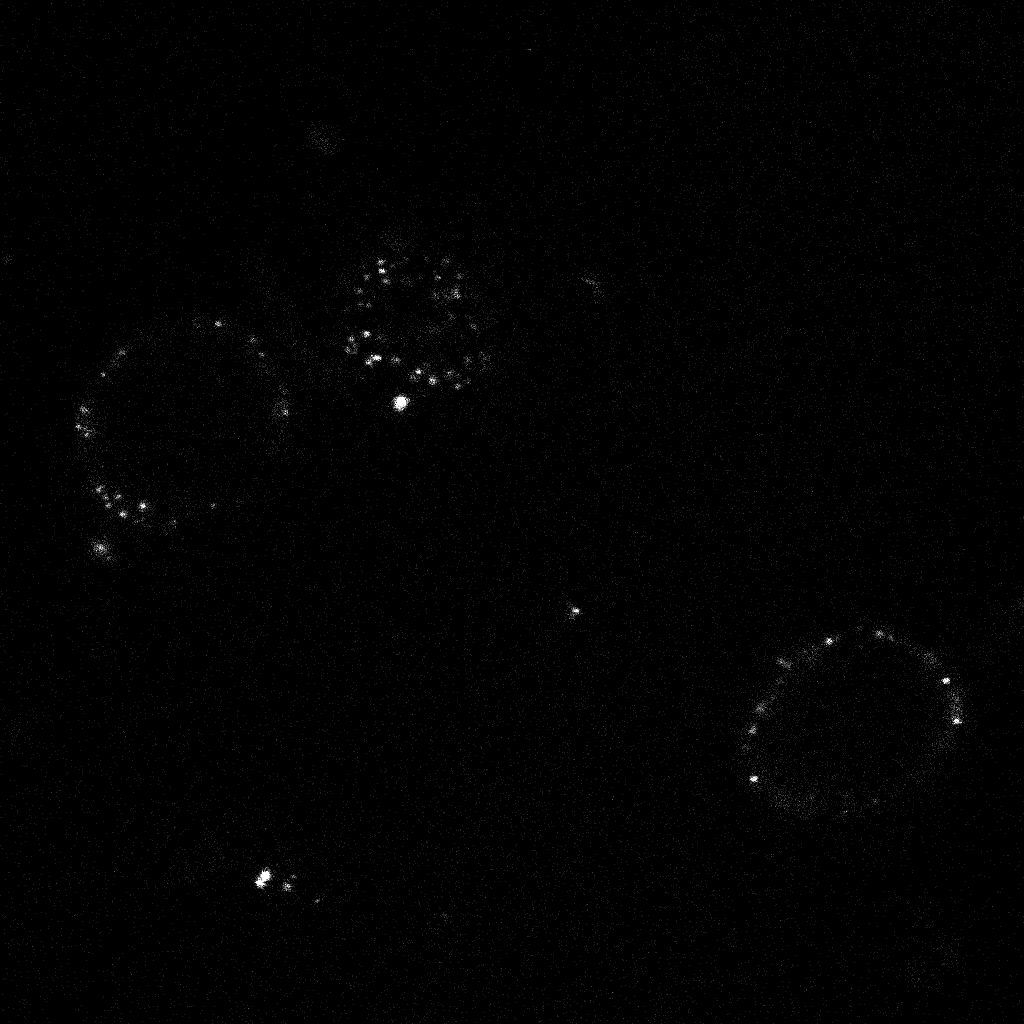

Supplement: Supplementary file 8 — Source data Fig. 1 [file 44319_2025_567_MOESM8_ESM.zip › Fig1/1D/Counted_nuclei/Mechanical/Nuc_15+16+17/Nuc_15+16+17_z18_RAW_ch00.tif]

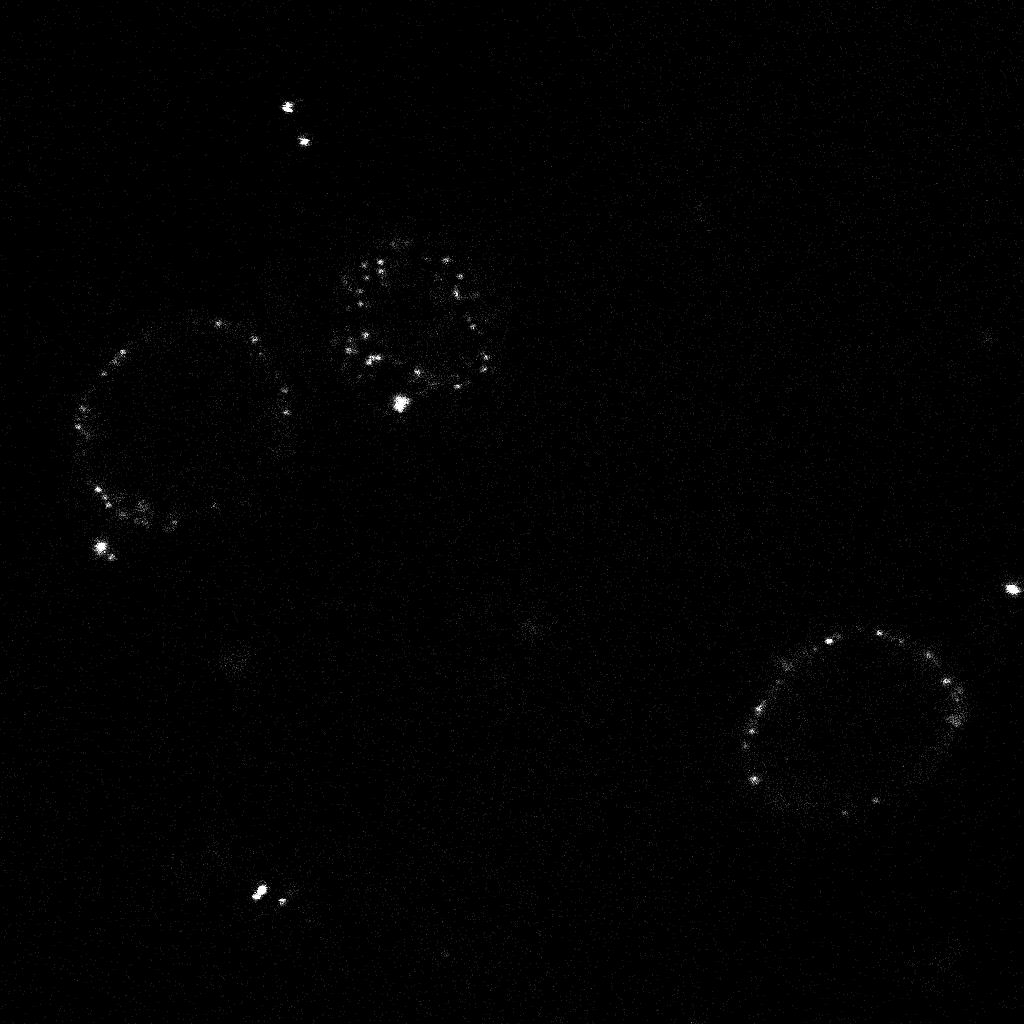

Supplement: Supplementary file 8 — Source data Fig. 1 [file 44319_2025_567_MOESM8_ESM.zip › Fig1/1D/Counted_nuclei/Mechanical/Nuc_15+16+17/Nuc_15+16+17_z19_RAW_ch00.tif]

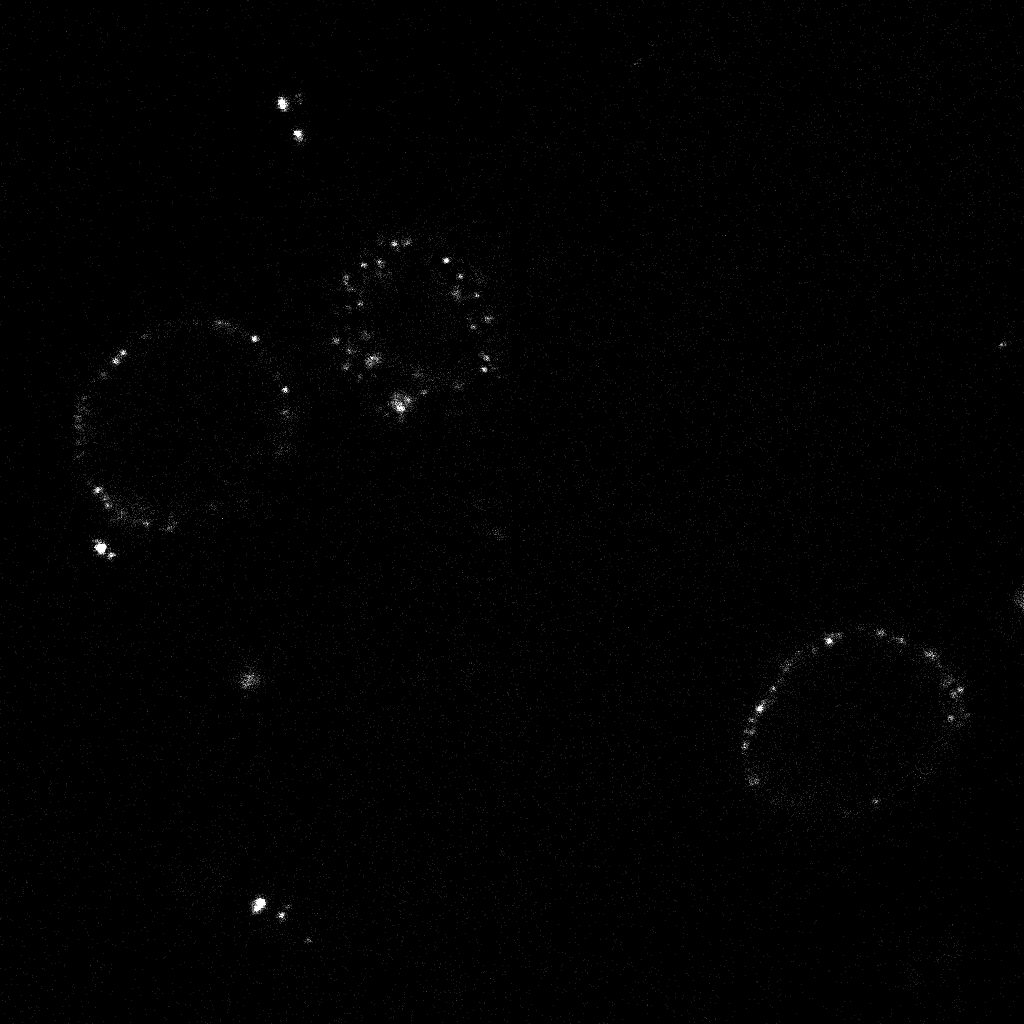

Supplement: Supplementary file 8 — Source data Fig. 1 [file 44319_2025_567_MOESM8_ESM.zip › Fig1/1D/Counted_nuclei/Mechanical/Nuc_15+16+17/Nuc_15+16+17_z20_RAW_ch00.tif]

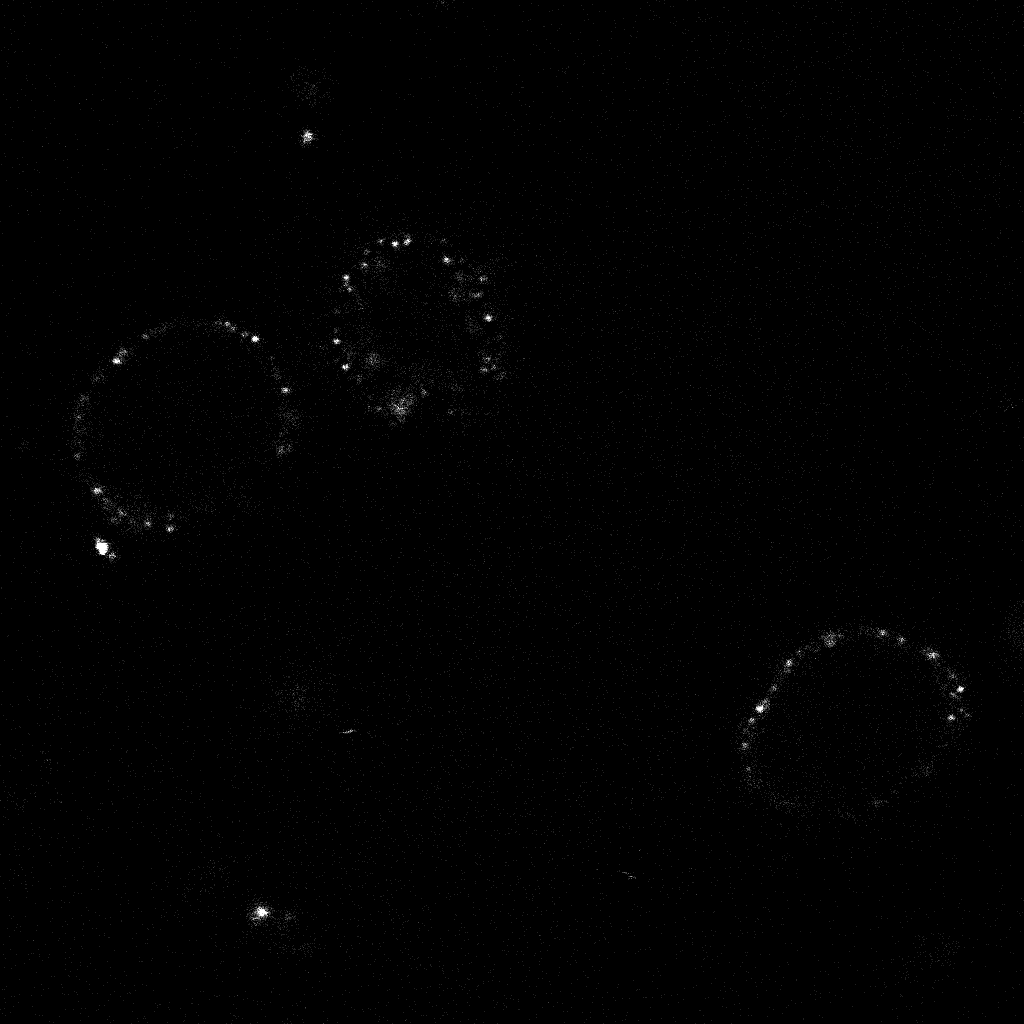

Supplement: Supplementary file 8 — Source data Fig. 1 [file 44319_2025_567_MOESM8_ESM.zip › Fig1/1D/Counted_nuclei/Mechanical/Nuc_15+16+17/Nuc_15+16+17_z21_RAW_ch00.tif]

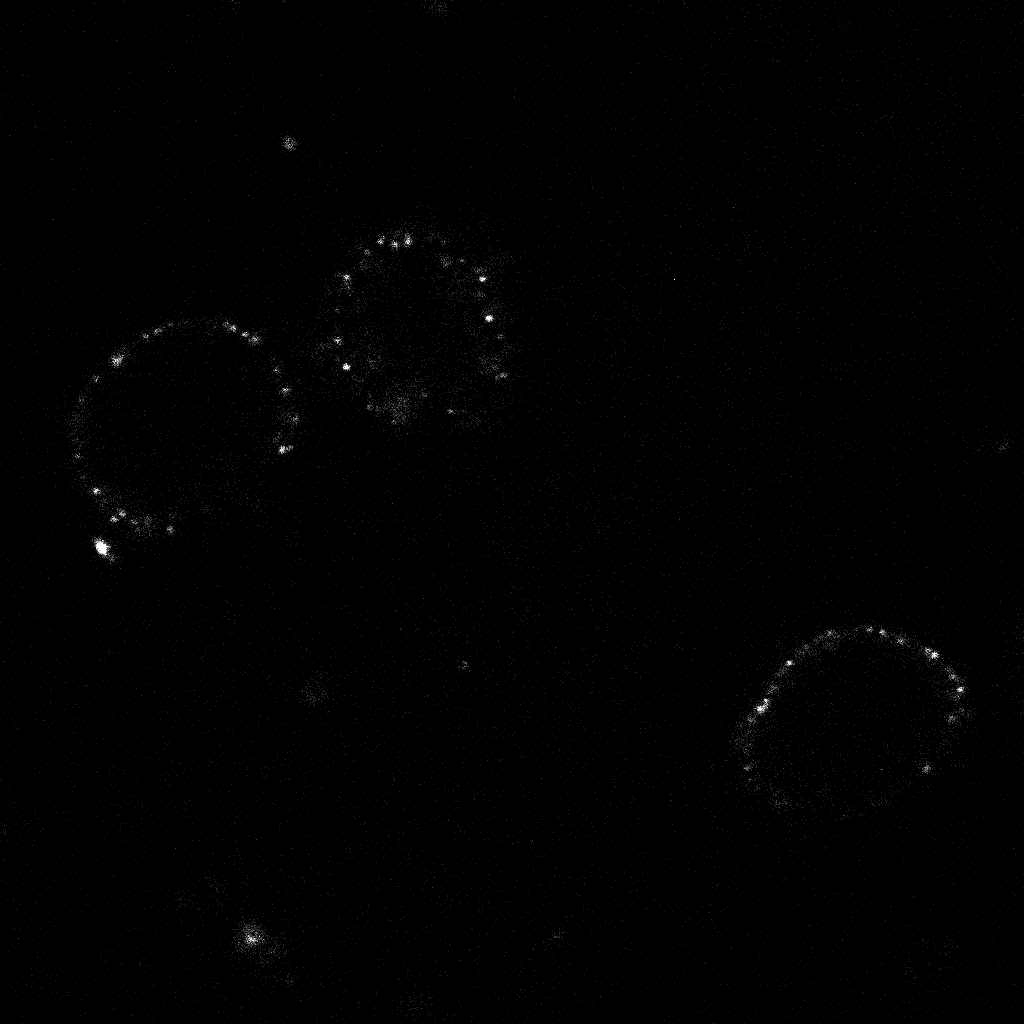

Supplement: Supplementary file 8 — Source data Fig. 1 [file 44319_2025_567_MOESM8_ESM.zip › Fig1/1D/Counted_nuclei/Mechanical/Nuc_15+16+17/Nuc_15+16+17_z22_RAW_ch00.tif]

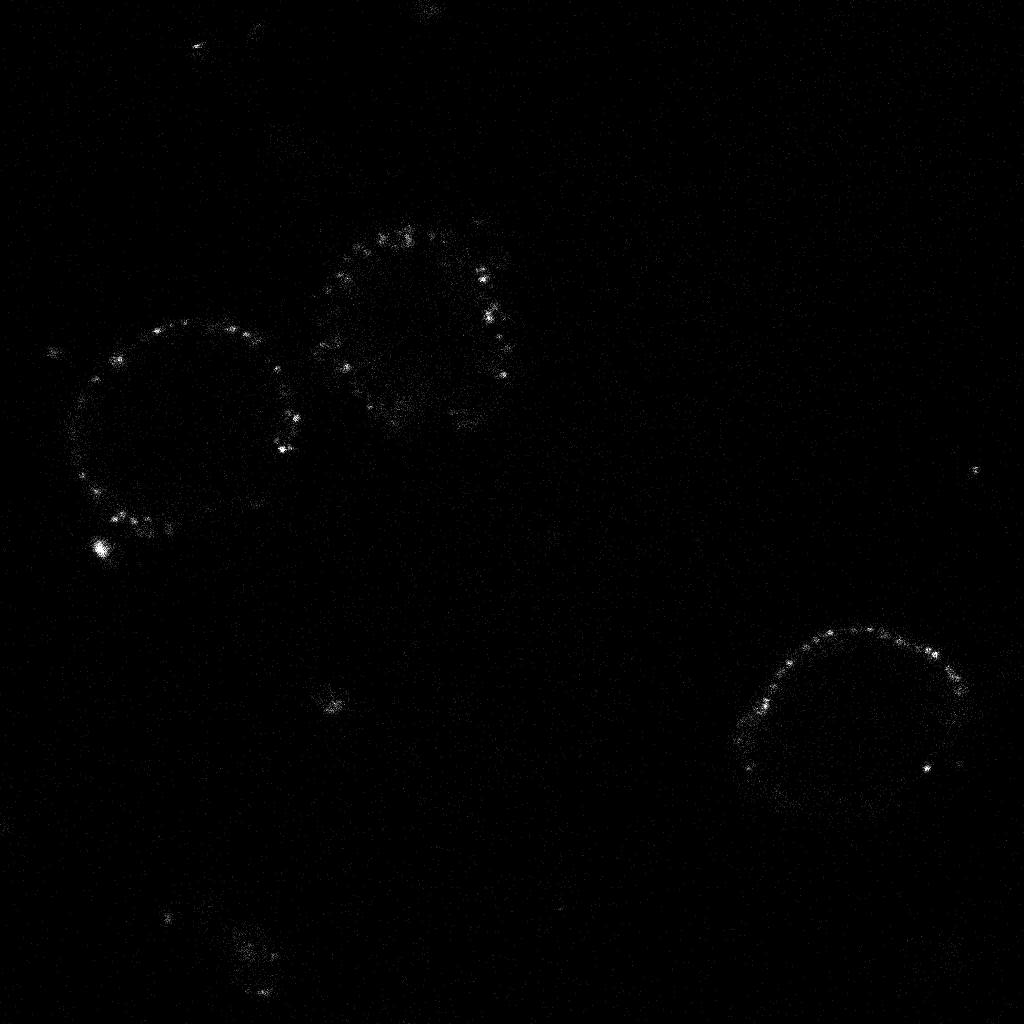

Supplement: Supplementary file 8 — Source data Fig. 1 [file 44319_2025_567_MOESM8_ESM.zip › Fig1/1D/Counted_nuclei/Mechanical/Nuc_15+16+17/Nuc_15+16+17_z23_RAW_ch00.tif]

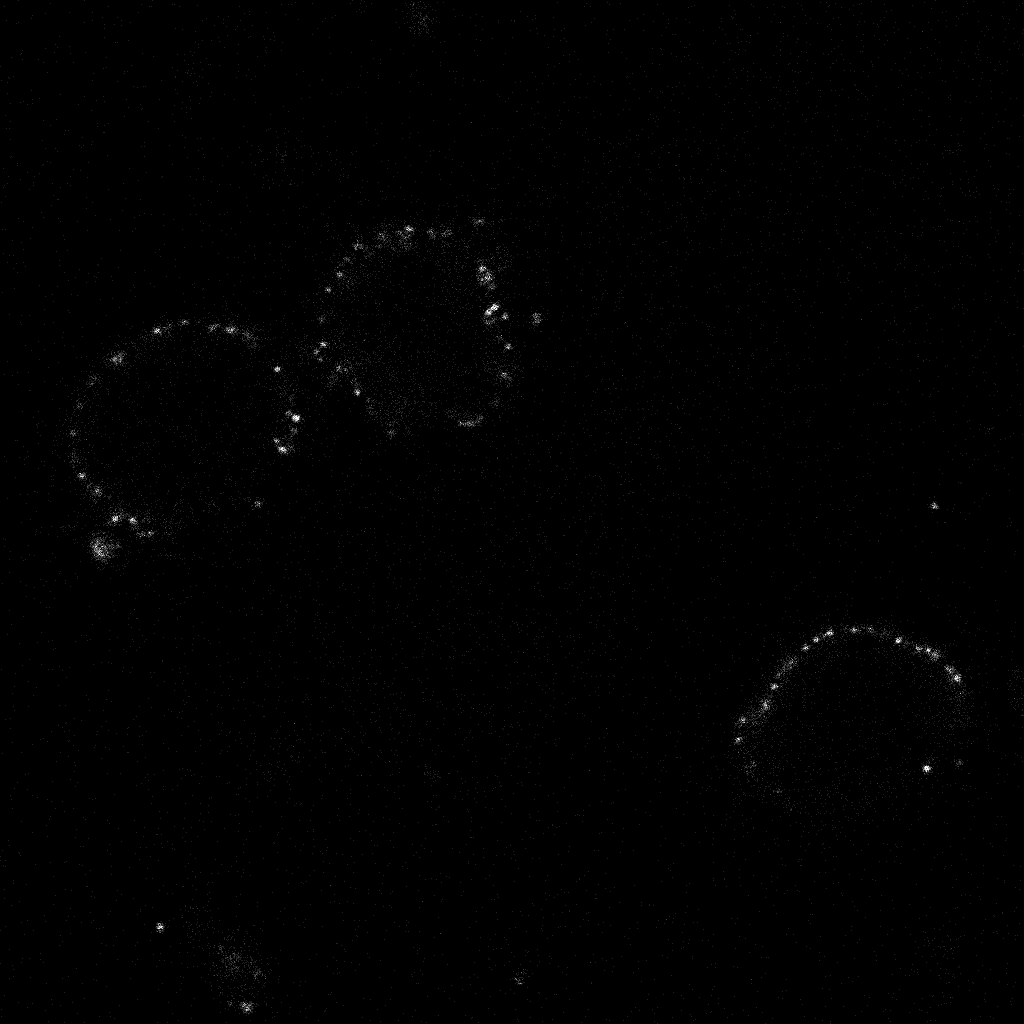

Supplement: Supplementary file 8 — Source data Fig. 1 [file 44319_2025_567_MOESM8_ESM.zip › Fig1/1D/Counted_nuclei/Mechanical/Nuc_15+16+17/Nuc_15+16+17_z24_RAW_ch00.tif]

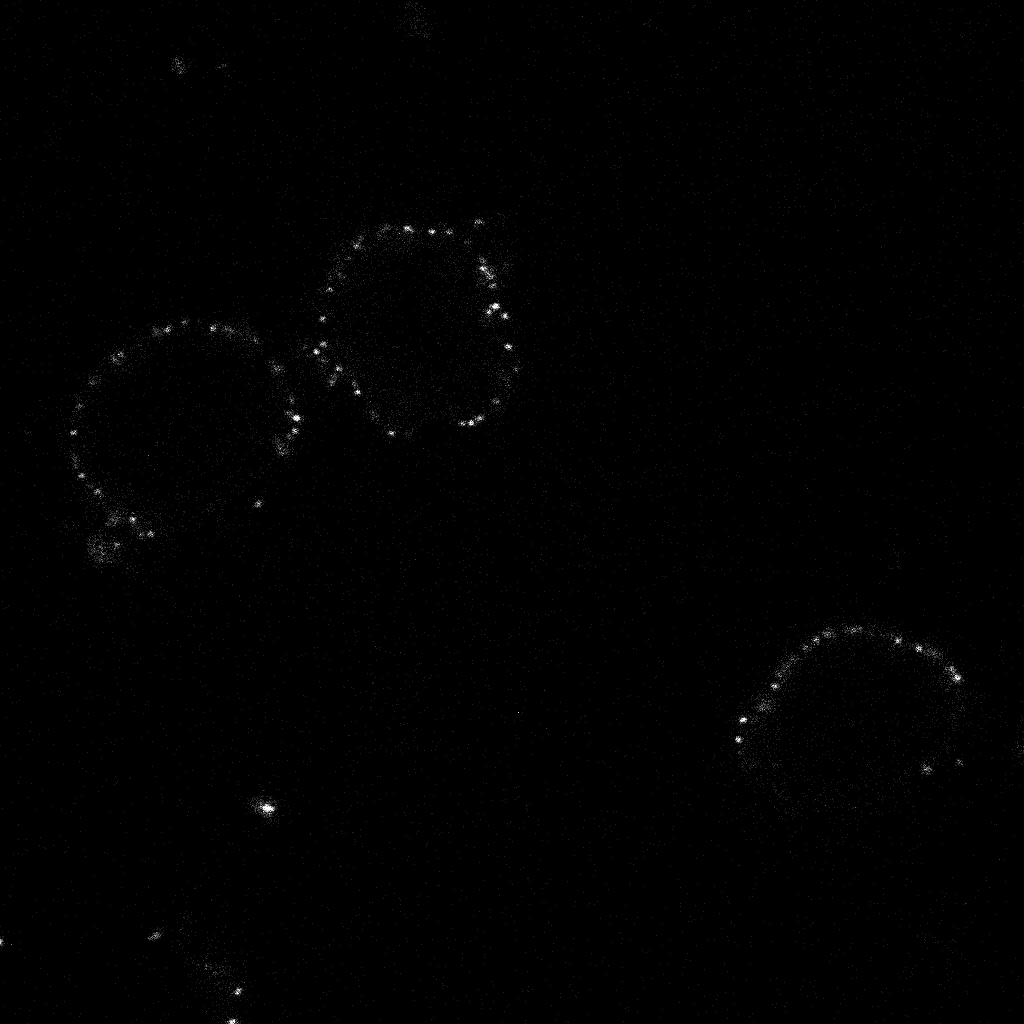

Supplement: Supplementary file 8 — Source data Fig. 1 [file 44319_2025_567_MOESM8_ESM.zip › Fig1/1D/Counted_nuclei/Mechanical/Nuc_15+16+17/Nuc_15+16+17_z25_RAW_ch00.tif]

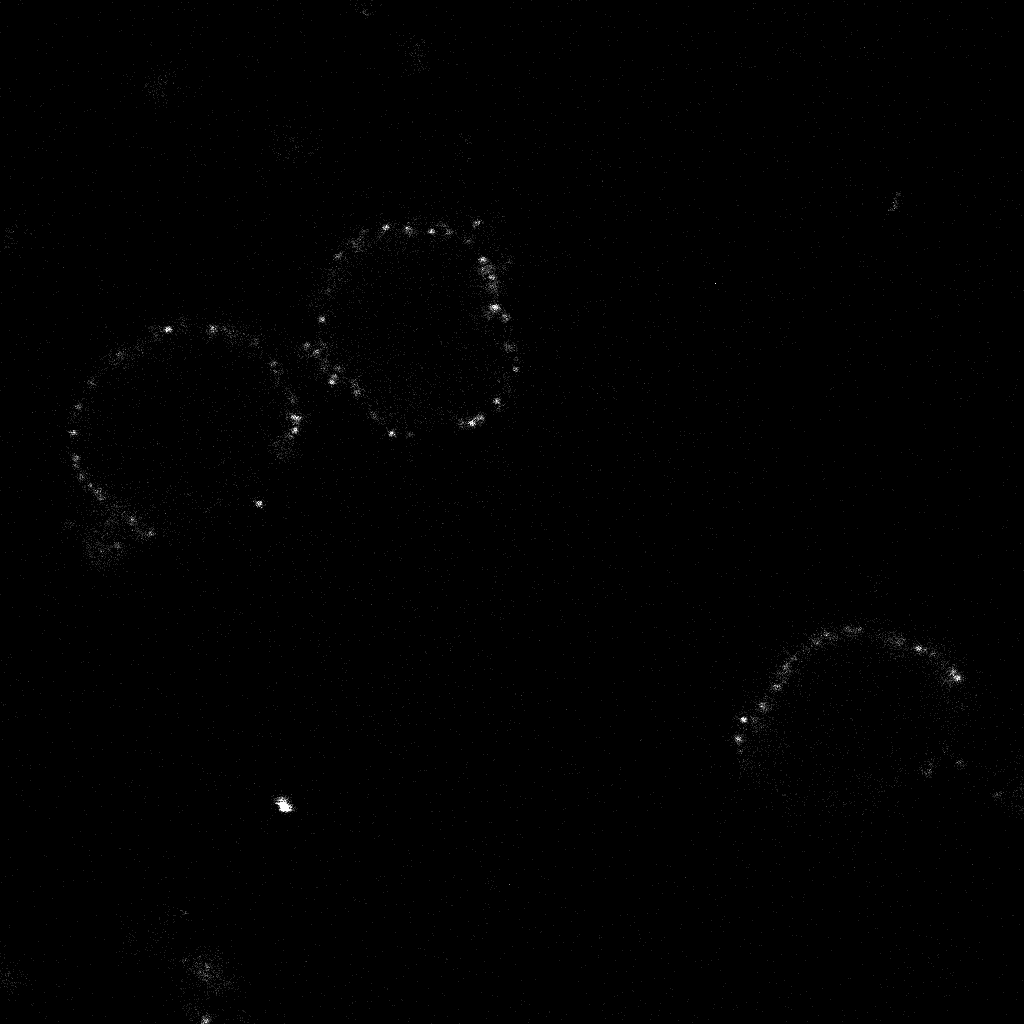

Supplement: Supplementary file 8 — Source data Fig. 1 [file 44319_2025_567_MOESM8_ESM.zip › Fig1/1D/Counted_nuclei/Mechanical/Nuc_15+16+17/Nuc_15+16+17_z26_RAW_ch00.tif]

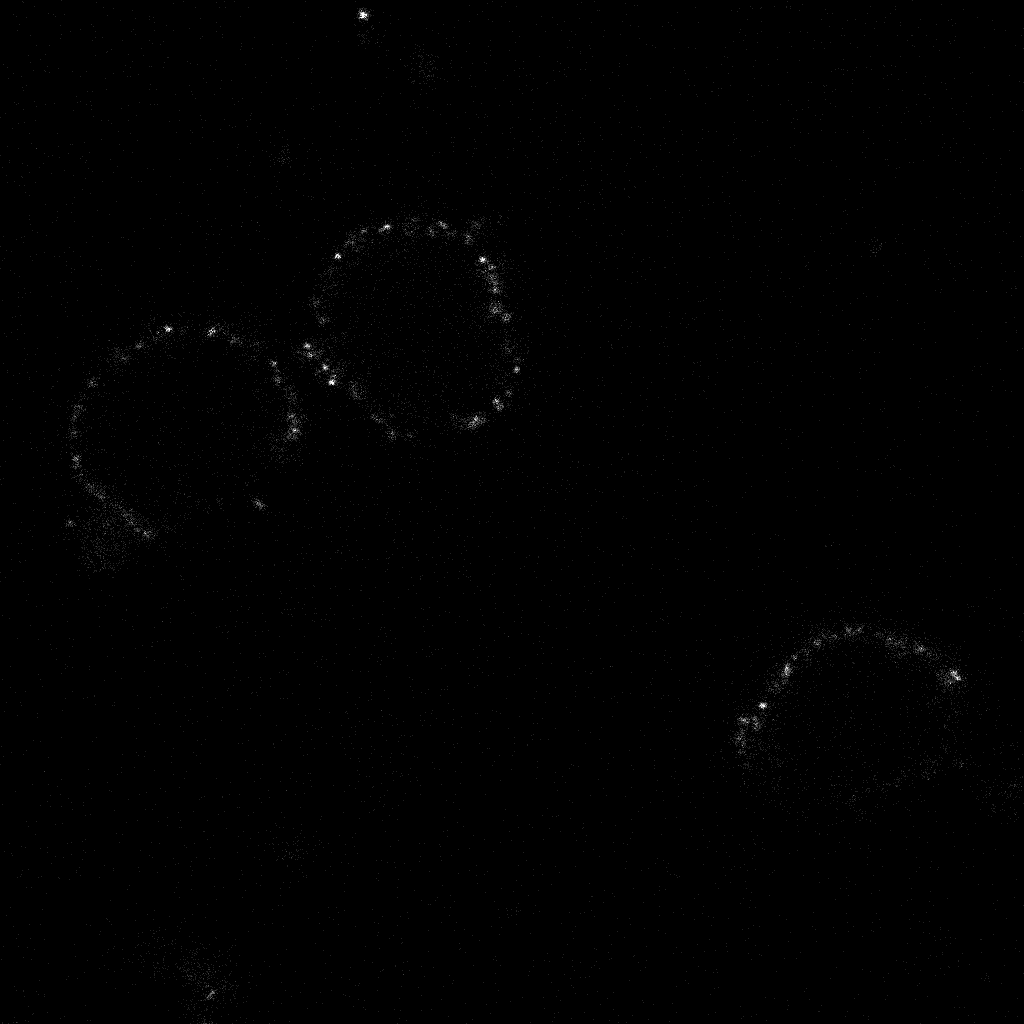

Supplement: Supplementary file 8 — Source data Fig. 1 [file 44319_2025_567_MOESM8_ESM.zip › Fig1/1D/Counted_nuclei/Mechanical/Nuc_15+16+17/Nuc_15+16+17_z27_RAW_ch00.tif]

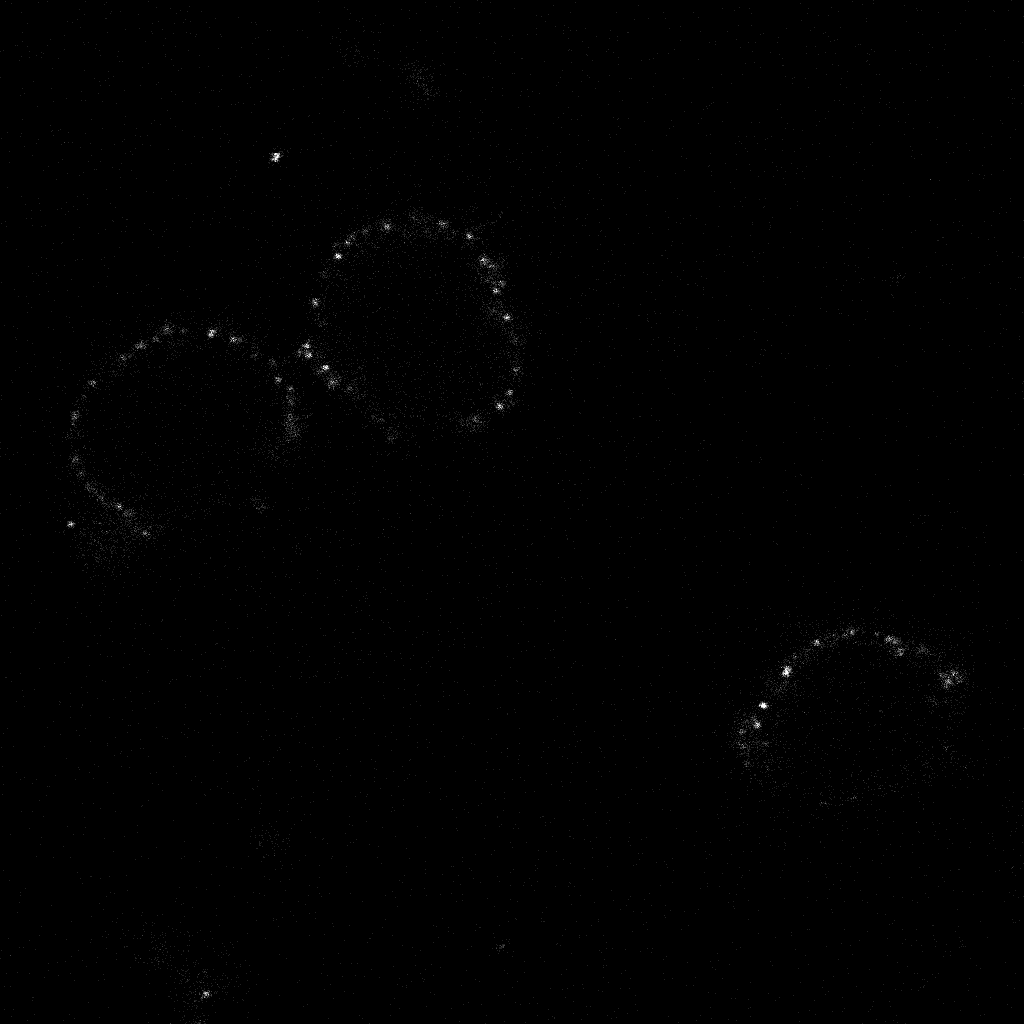

Supplement: Supplementary file 8 — Source data Fig. 1 [file 44319_2025_567_MOESM8_ESM.zip › Fig1/1D/Counted_nuclei/Mechanical/Nuc_15+16+17/Nuc_15+16+17_z28_RAW_ch00.tif]

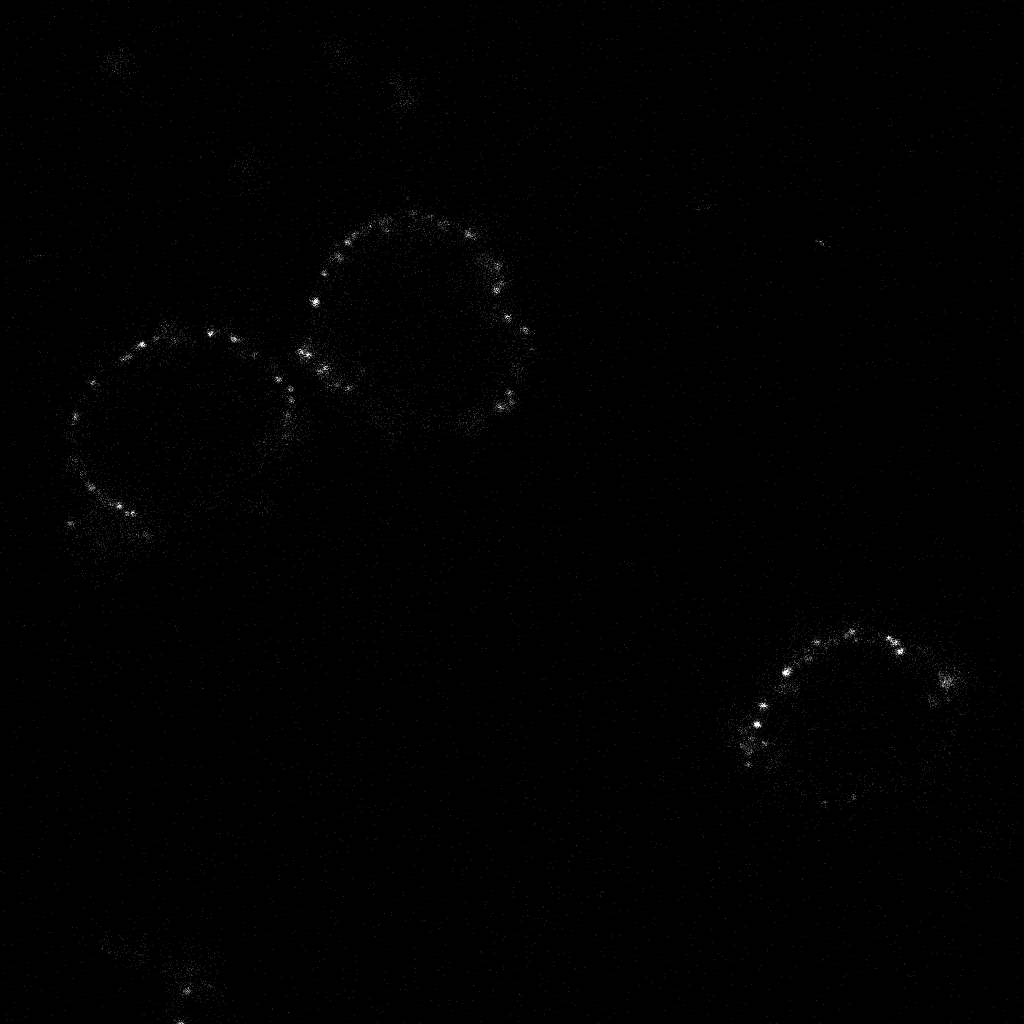

Supplement: Supplementary file 8 — Source data Fig. 1 [file 44319_2025_567_MOESM8_ESM.zip › Fig1/1D/Counted_nuclei/Mechanical/Nuc_15+16+17/Nuc_15+16+17_z29_RAW_ch00.tif]

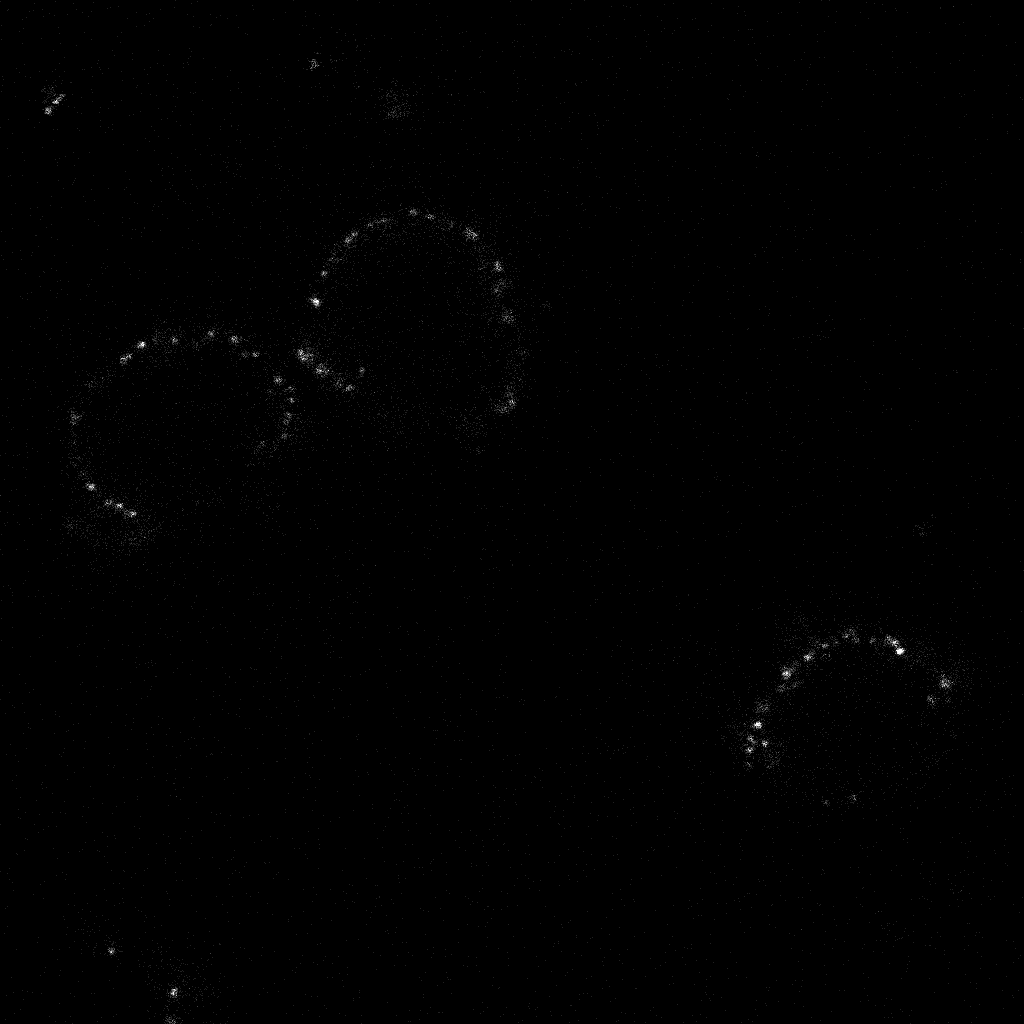

Supplement: Supplementary file 8 — Source data Fig. 1 [file 44319_2025_567_MOESM8_ESM.zip › Fig1/1D/Counted_nuclei/Mechanical/Nuc_15+16+17/Nuc_15+16+17_z30_RAW_ch00.tif]

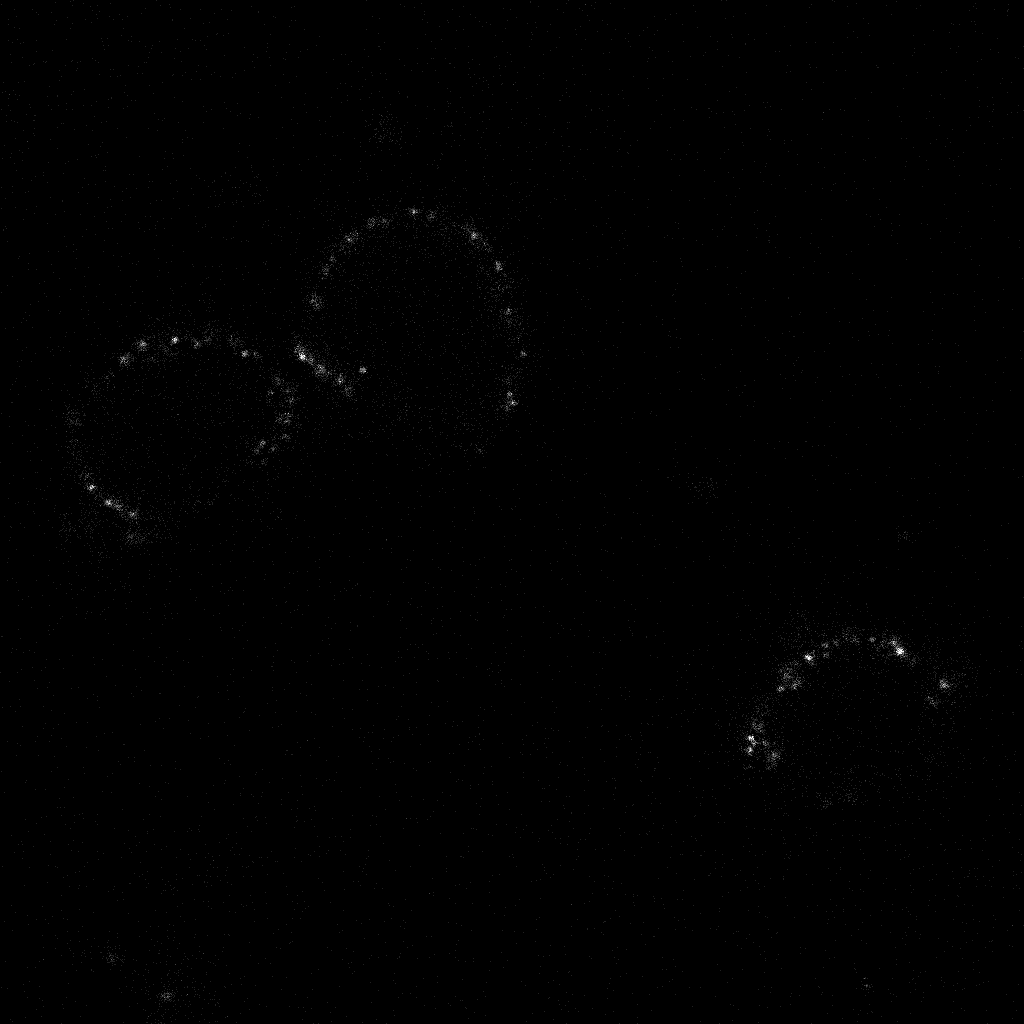

Supplement: Supplementary file 8 — Source data Fig. 1 [file 44319_2025_567_MOESM8_ESM.zip › Fig1/1D/Counted_nuclei/Mechanical/Nuc_15+16+17/Nuc_15+16+17_z31_RAW_ch00.tif]

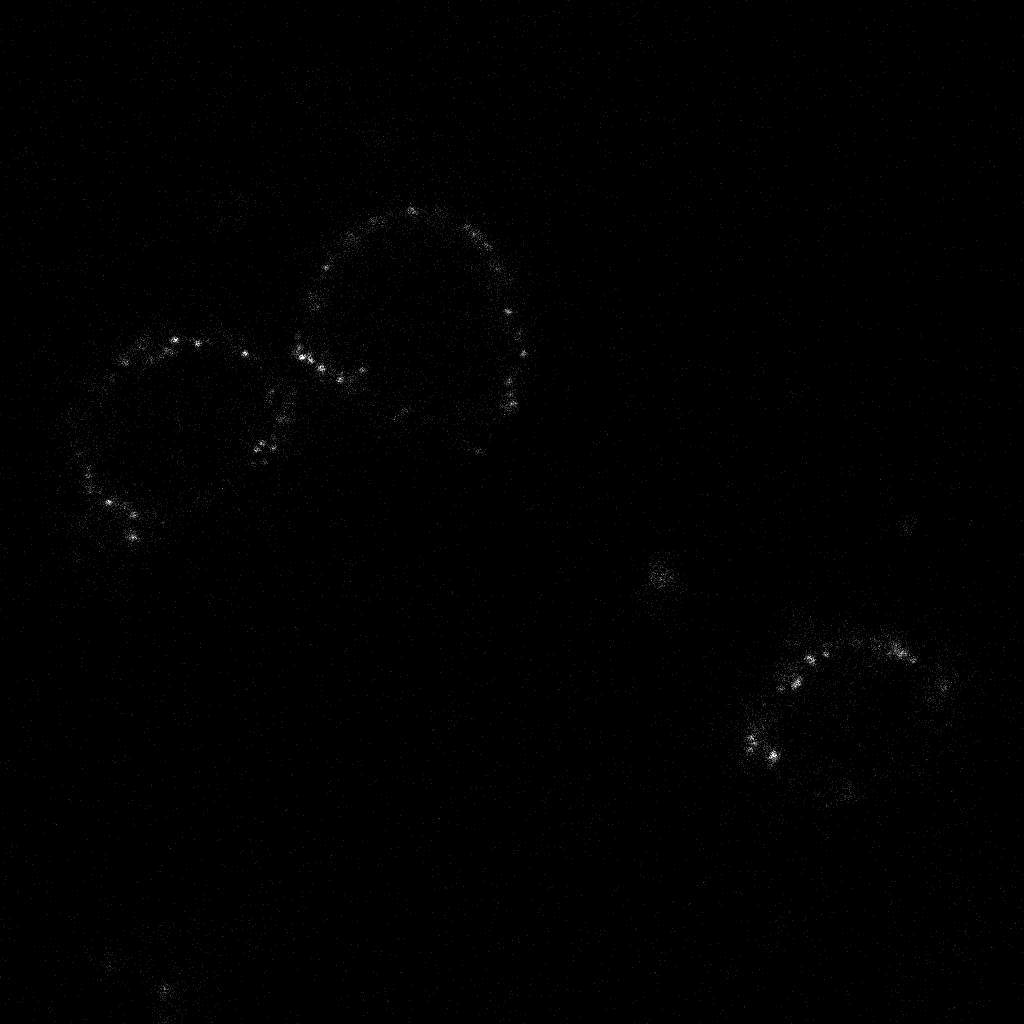

Supplement: Supplementary file 8 — Source data Fig. 1 [file 44319_2025_567_MOESM8_ESM.zip › Fig1/1D/Counted_nuclei/Mechanical/Nuc_15+16+17/Nuc_15+16+17_z32_RAW_ch00.tif]

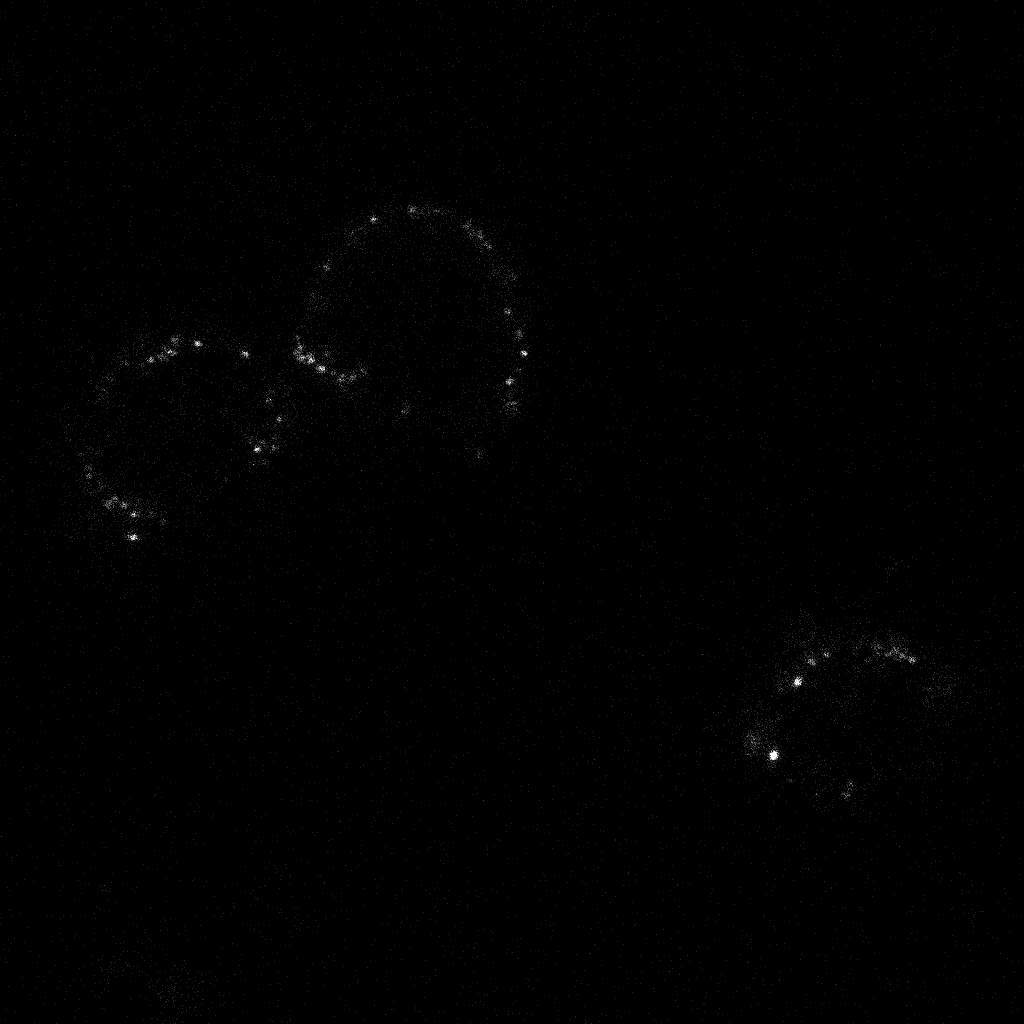

Supplement: Supplementary file 8 — Source data Fig. 1 [file 44319_2025_567_MOESM8_ESM.zip › Fig1/1D/Counted_nuclei/Mechanical/Nuc_15+16+17/Nuc_15+16+17_z33_RAW_ch00.tif]

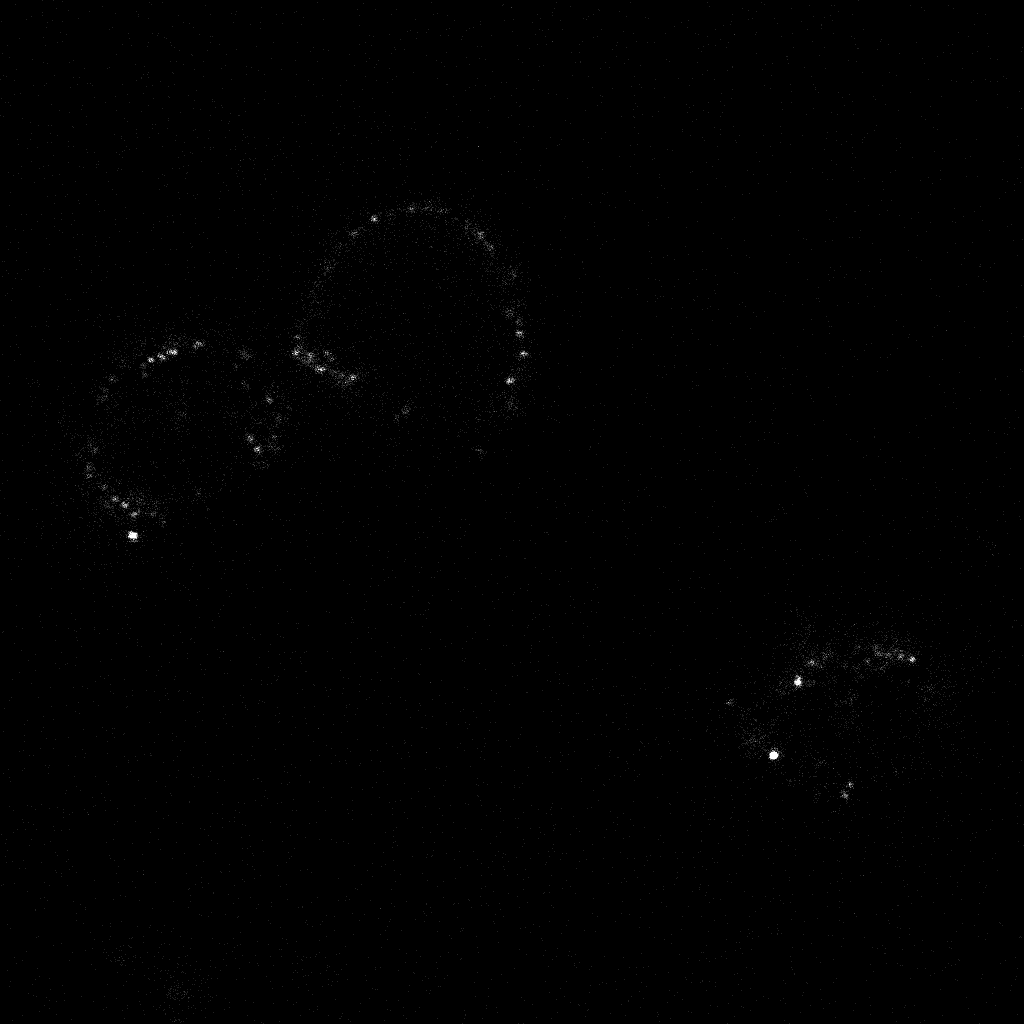

Supplement: Supplementary file 8 — Source data Fig. 1 [file 44319_2025_567_MOESM8_ESM.zip › Fig1/1D/Counted_nuclei/Mechanical/Nuc_15+16+17/Nuc_15+16+17_z34_RAW_ch00.tif]

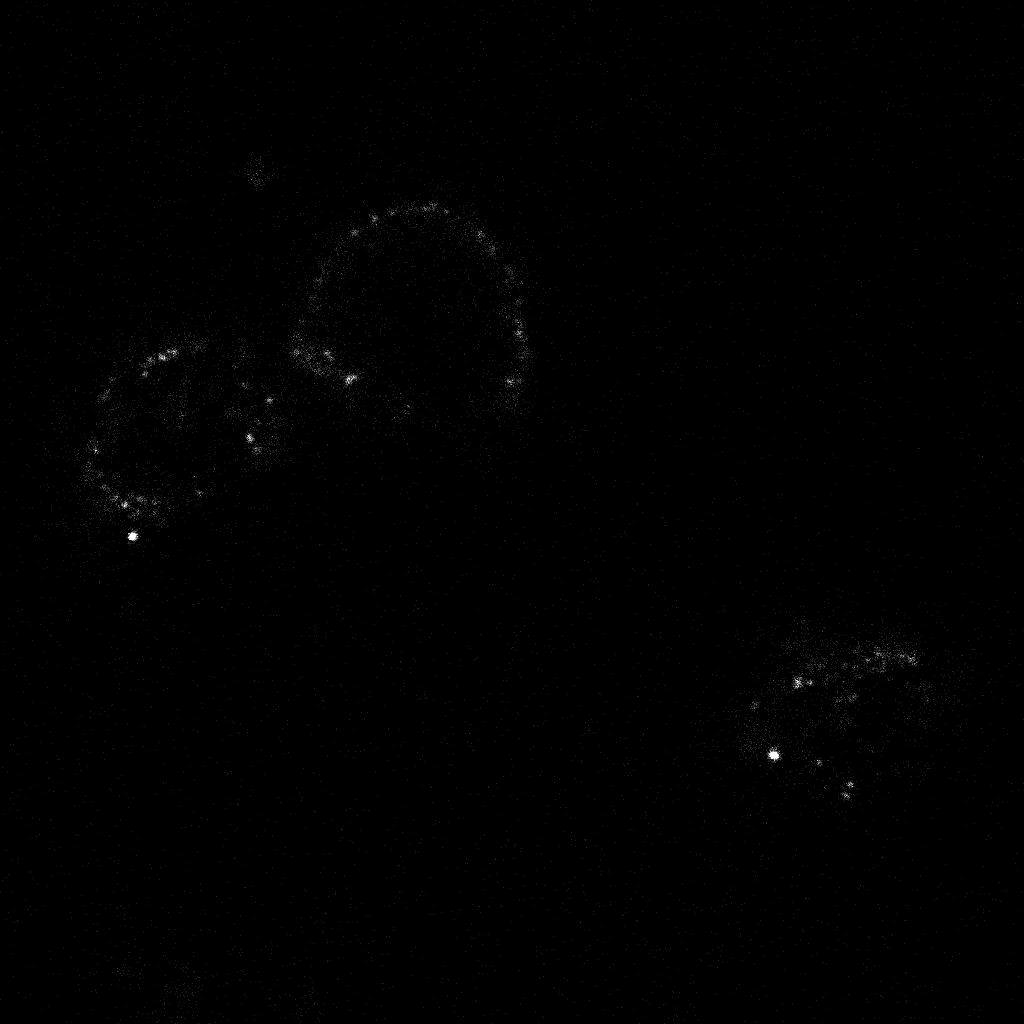

Supplement: Supplementary file 8 — Source data Fig. 1 [file 44319_2025_567_MOESM8_ESM.zip › Fig1/1D/Counted_nuclei/Mechanical/Nuc_15+16+17/Nuc_15+16+17_z35_RAW_ch00.tif]

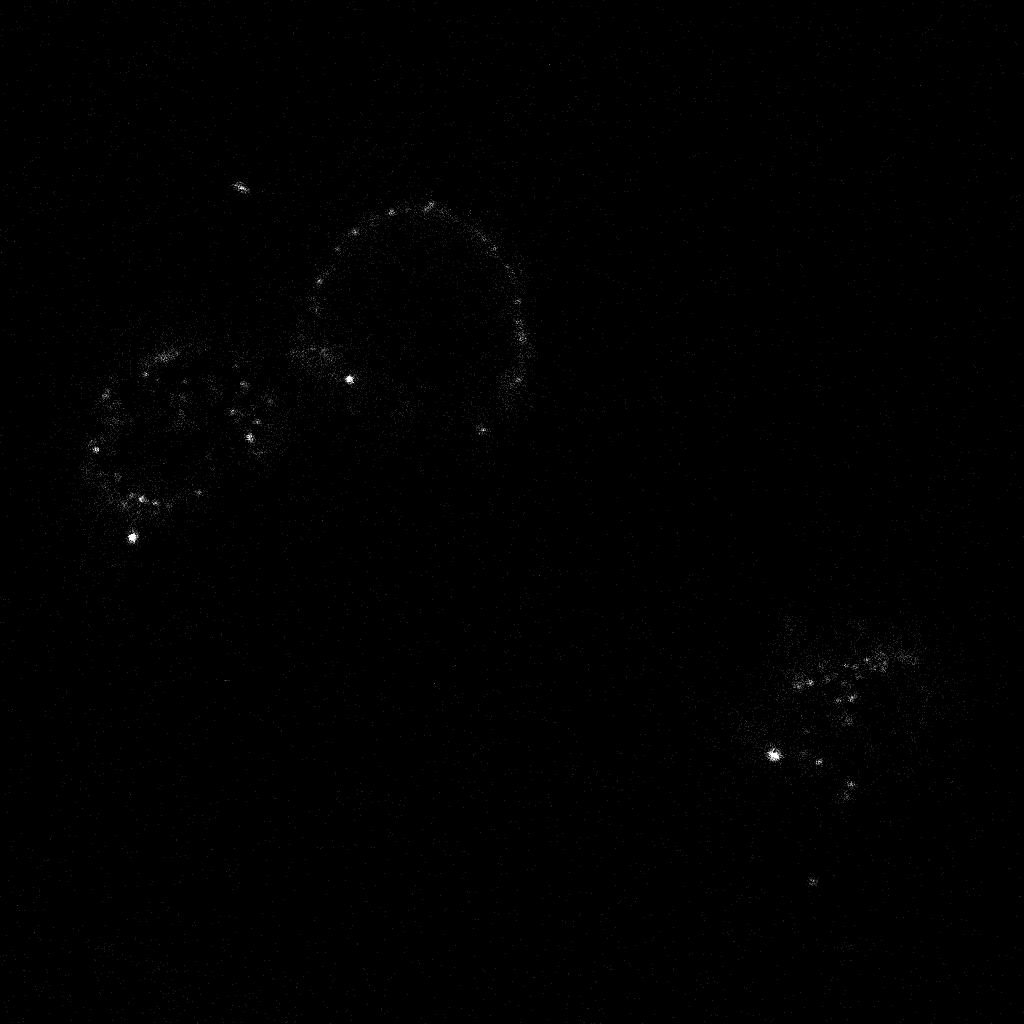

Supplement: Supplementary file 8 — Source data Fig. 1 [file 44319_2025_567_MOESM8_ESM.zip › Fig1/1D/Counted_nuclei/Mechanical/Nuc_15+16+17/Nuc_15+16+17_z36_RAW_ch00.tif]

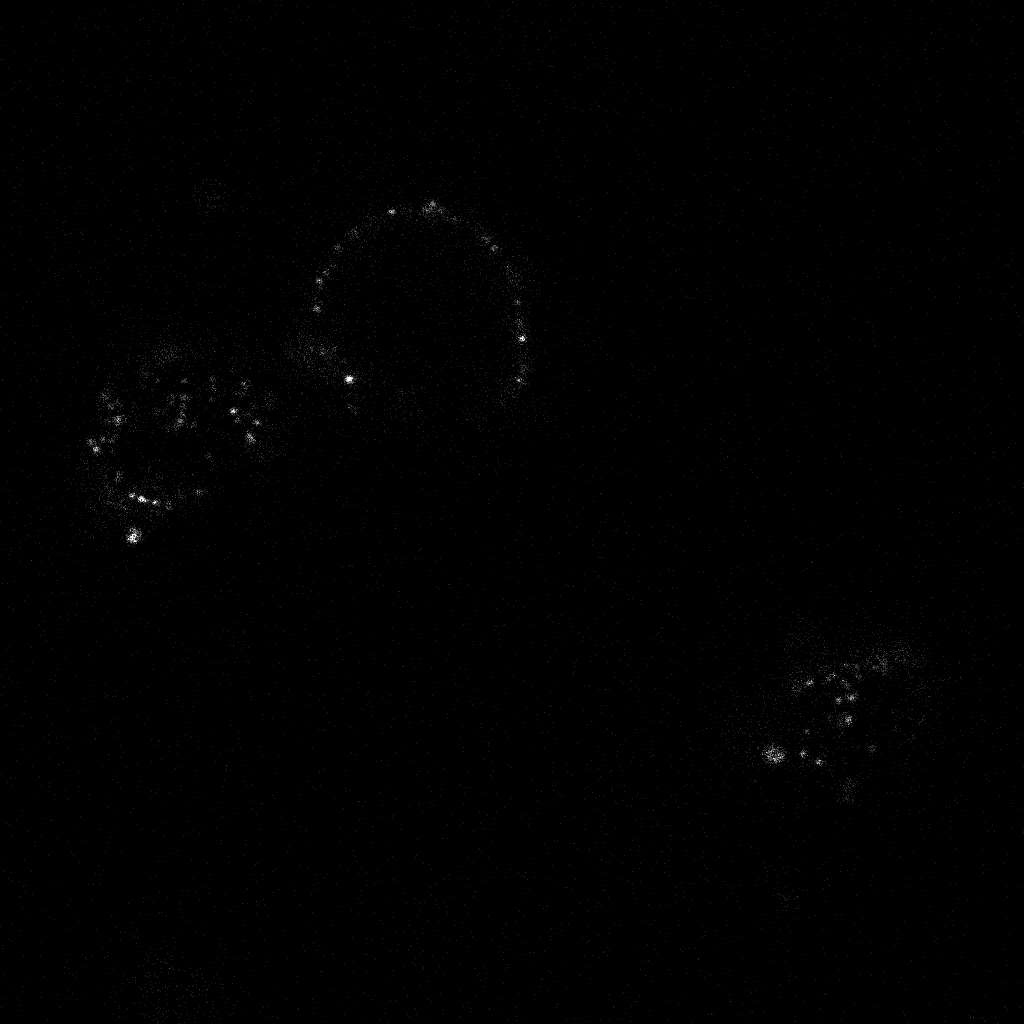

Supplement: Supplementary file 8 — Source data Fig. 1 [file 44319_2025_567_MOESM8_ESM.zip › Fig1/1D/Counted_nuclei/Mechanical/Nuc_15+16+17/Nuc_15+16+17_z37_RAW_ch00.tif]

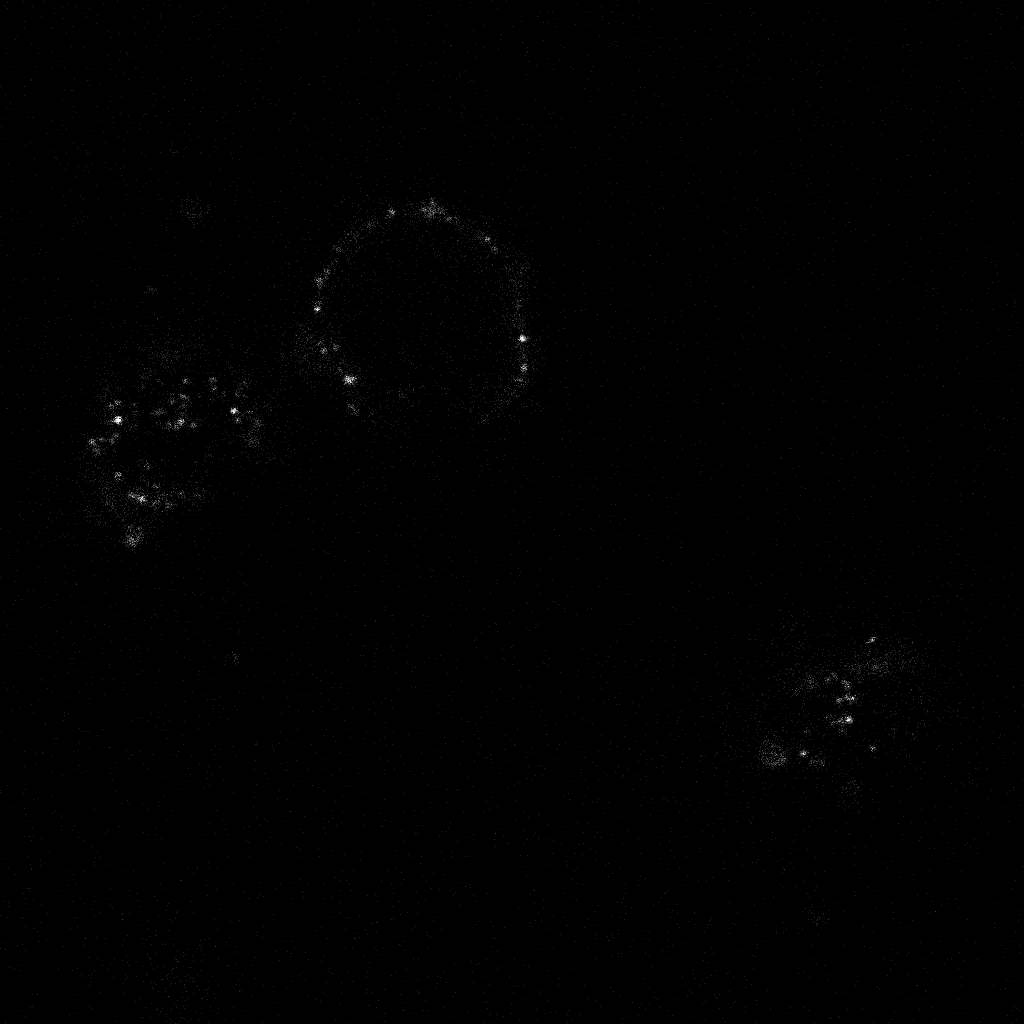

Supplement: Supplementary file 8 — Source data Fig. 1 [file 44319_2025_567_MOESM8_ESM.zip › Fig1/1D/Counted_nuclei/Mechanical/Nuc_15+16+17/Nuc_15+16+17_z38_RAW_ch00.tif]

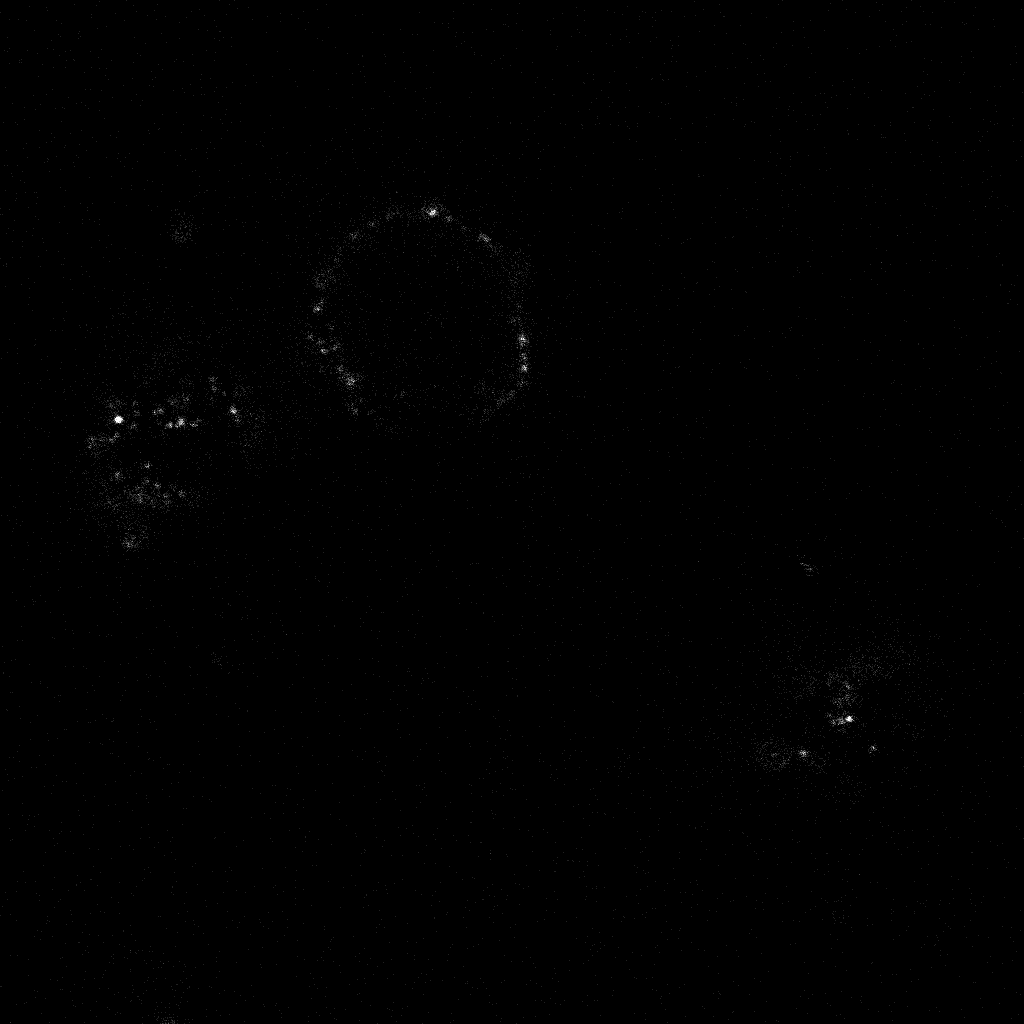

Supplement: Supplementary file 8 — Source data Fig. 1 [file 44319_2025_567_MOESM8_ESM.zip › Fig1/1D/Counted_nuclei/Mechanical/Nuc_15+16+17/Nuc_15+16+17_z39_RAW_ch00.tif]

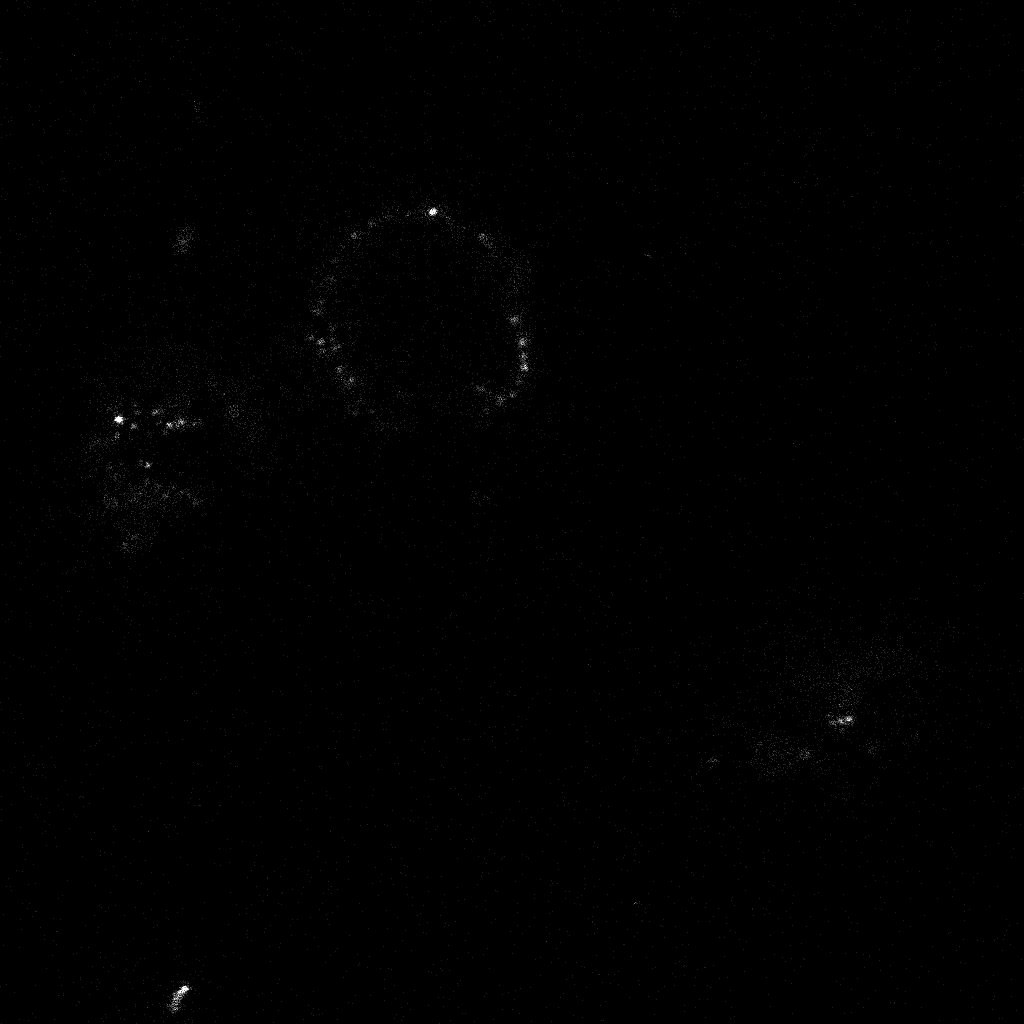

Supplement: Supplementary file 8 — Source data Fig. 1 [file 44319_2025_567_MOESM8_ESM.zip › Fig1/1D/Counted_nuclei/Mechanical/Nuc_15+16+17/Nuc_15+16+17_z40_RAW_ch00.tif]

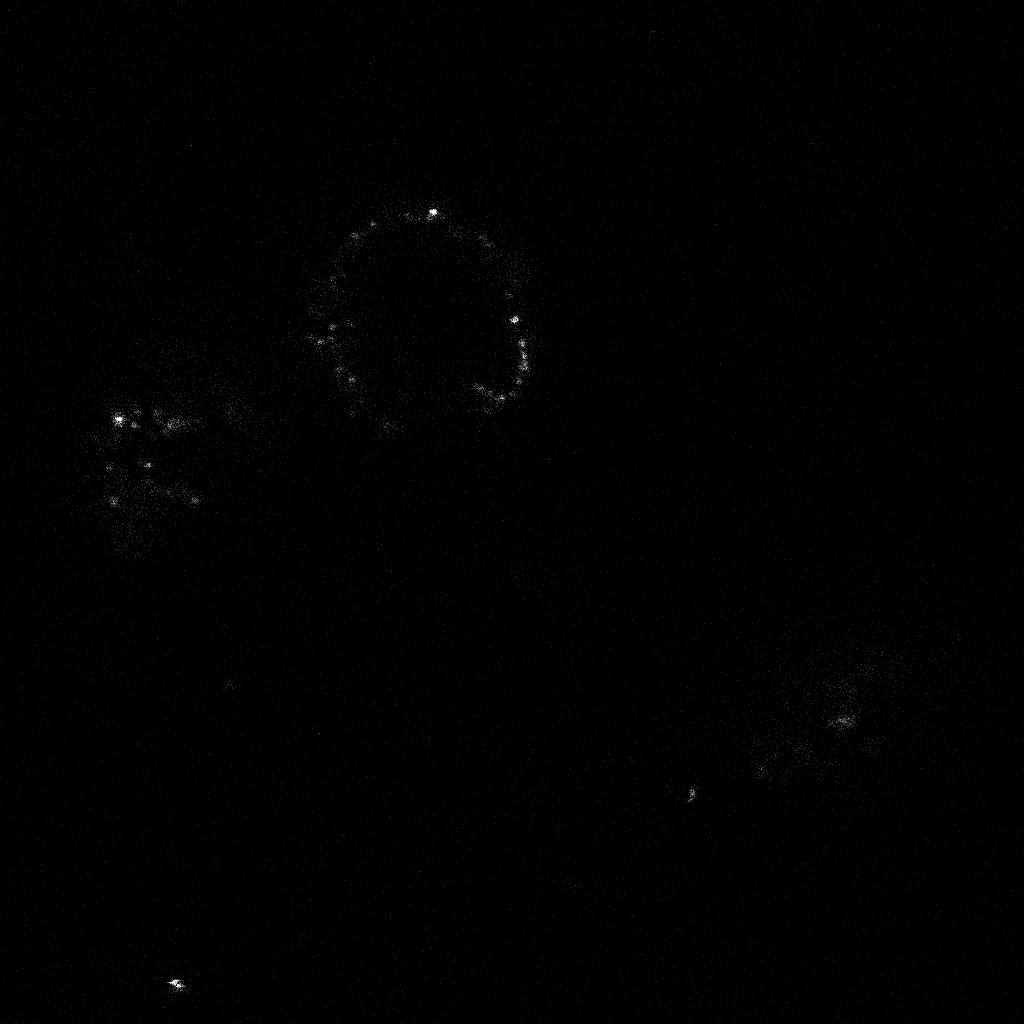

Supplement: Supplementary file 8 — Source data Fig. 1 [file 44319_2025_567_MOESM8_ESM.zip › Fig1/1D/Counted_nuclei/Mechanical/Nuc_15+16+17/Nuc_15+16+17_z41_RAW_ch00.tif]

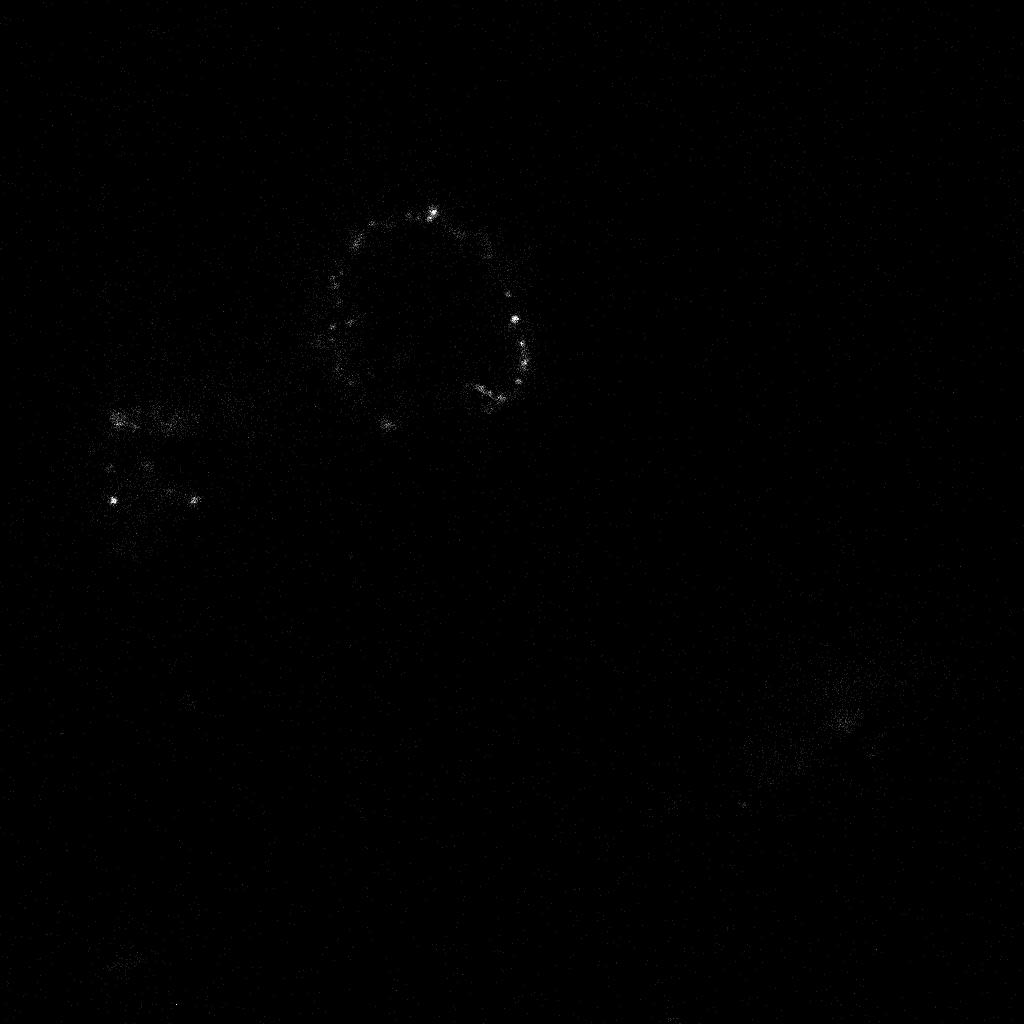

Supplement: Supplementary file 8 — Source data Fig. 1 [file 44319_2025_567_MOESM8_ESM.zip › Fig1/1D/Counted_nuclei/Mechanical/Nuc_15+16+17/Nuc_15+16+17_z42_RAW_ch00.tif]

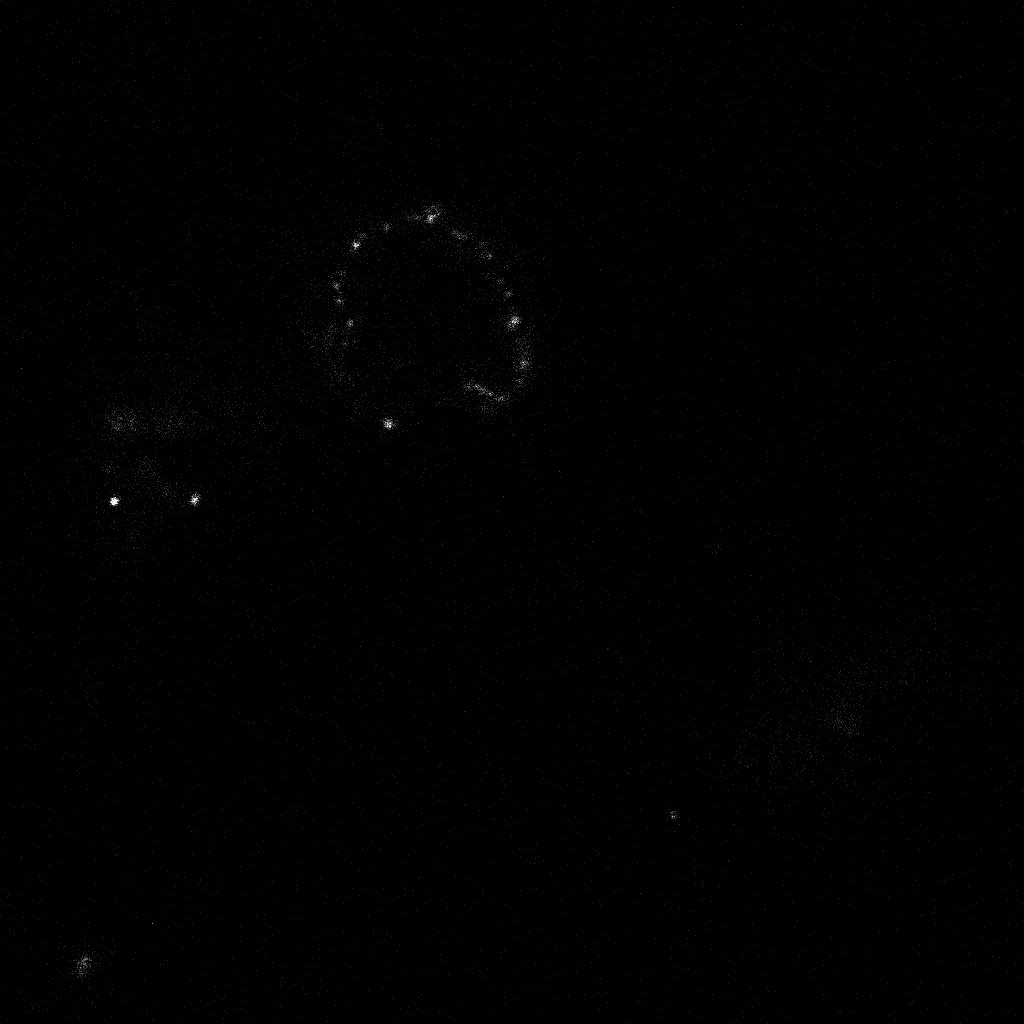

Supplement: Supplementary file 8 — Source data Fig. 1 [file 44319_2025_567_MOESM8_ESM.zip › Fig1/1D/Counted_nuclei/Mechanical/Nuc_15+16+17/Nuc_15+16+17_z43_RAW_ch00.tif]

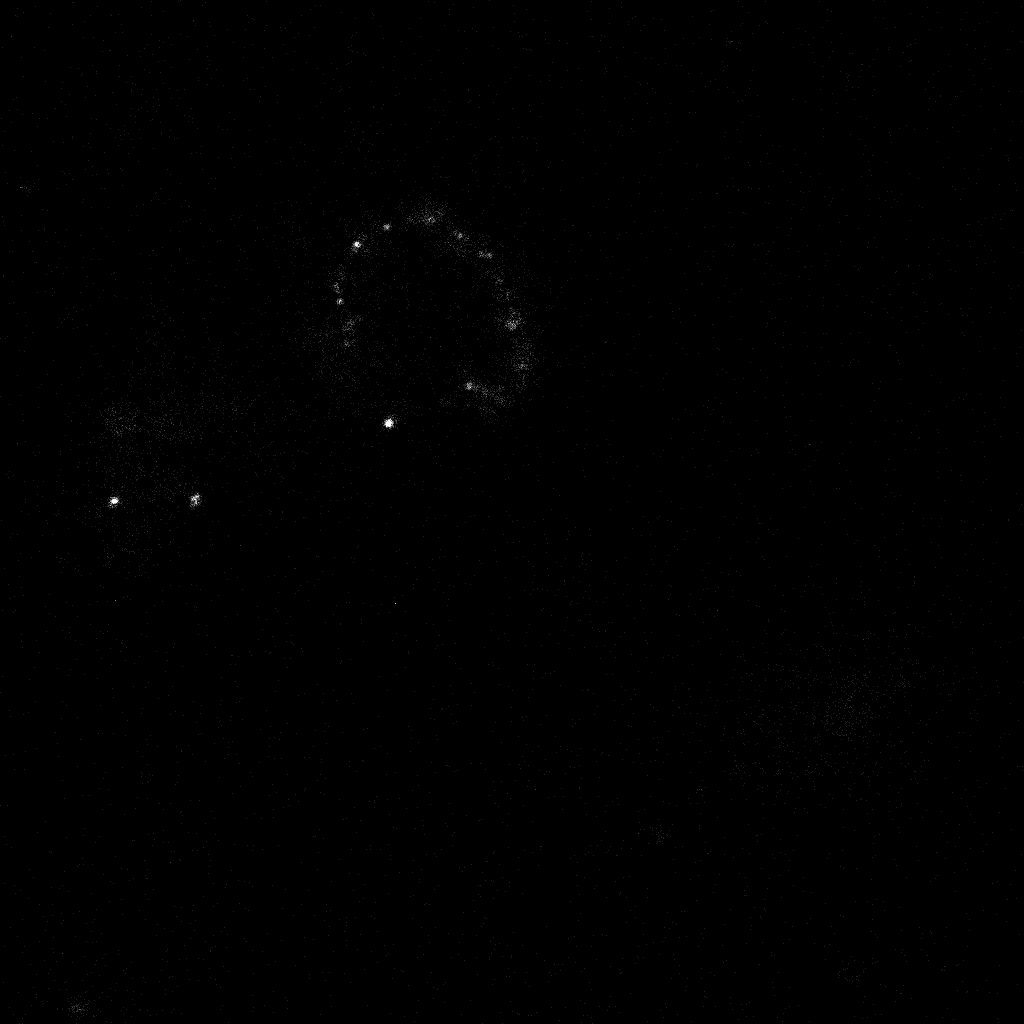

Supplement: Supplementary file 8 — Source data Fig. 1 [file 44319_2025_567_MOESM8_ESM.zip › Fig1/1D/Counted_nuclei/Mechanical/Nuc_15+16+17/Nuc_15+16+17_z44_RAW_ch00.tif]

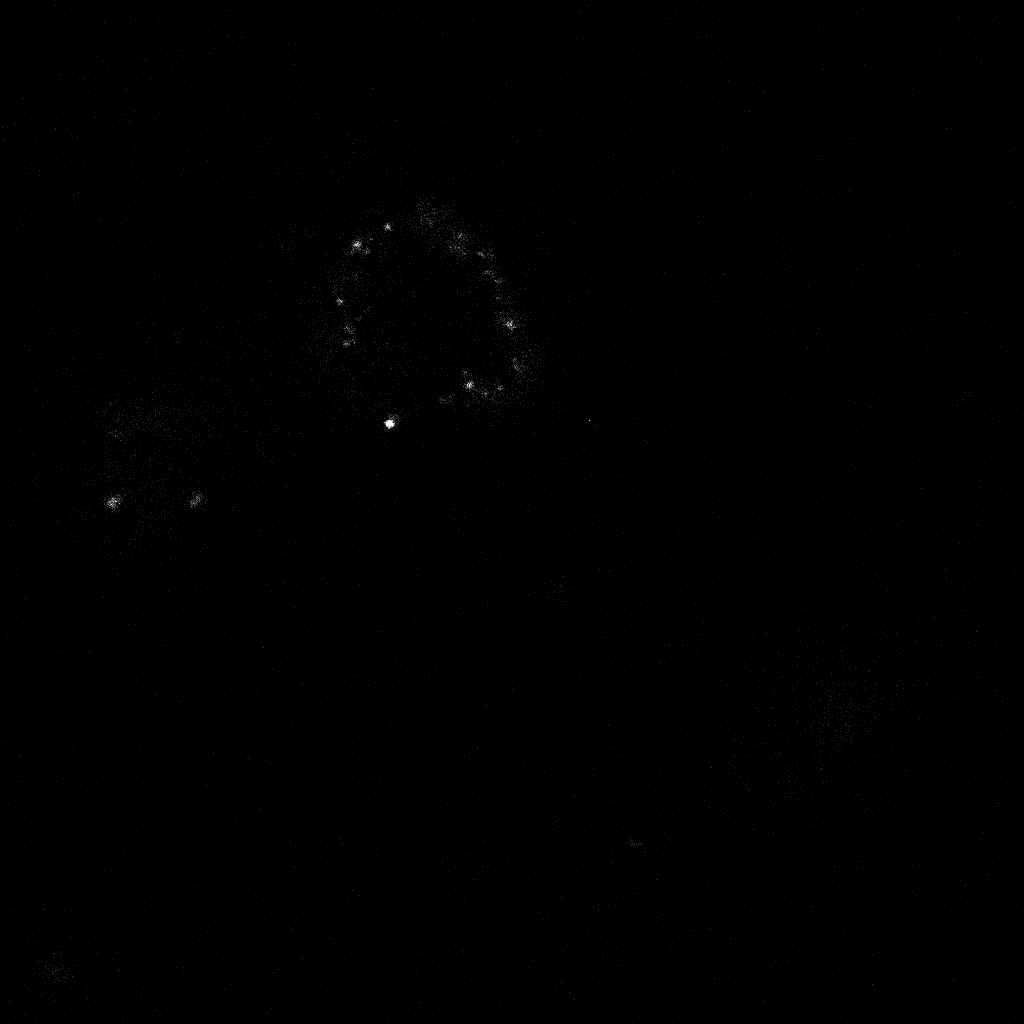

Supplement: Supplementary file 8 — Source data Fig. 1 [file 44319_2025_567_MOESM8_ESM.zip › Fig1/1D/Counted_nuclei/Mechanical/Nuc_15+16+17/Nuc_15+16+17_z45_RAW_ch00.tif]

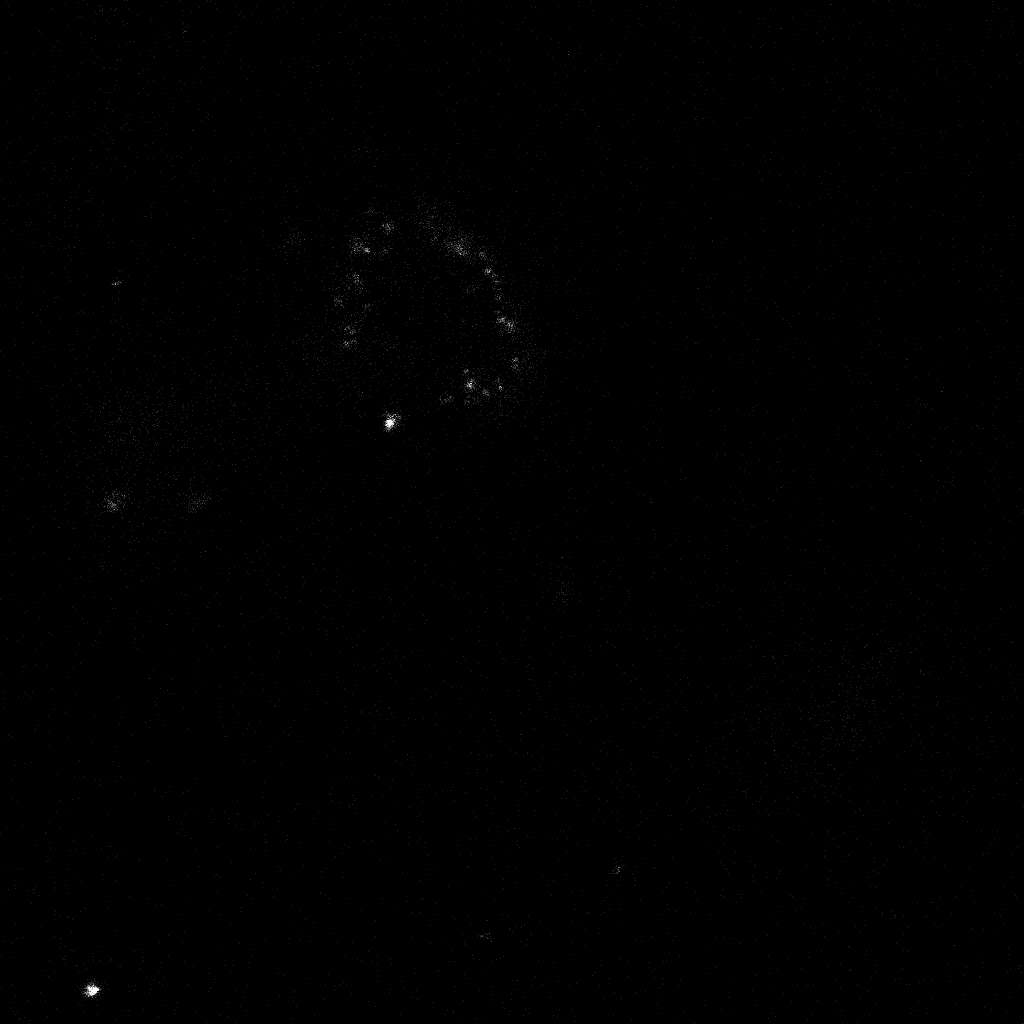

Supplement: Supplementary file 8 — Source data Fig. 1 [file 44319_2025_567_MOESM8_ESM.zip › Fig1/1D/Counted_nuclei/Mechanical/Nuc_15+16+17/Nuc_15+16+17_z46_RAW_ch00.tif]

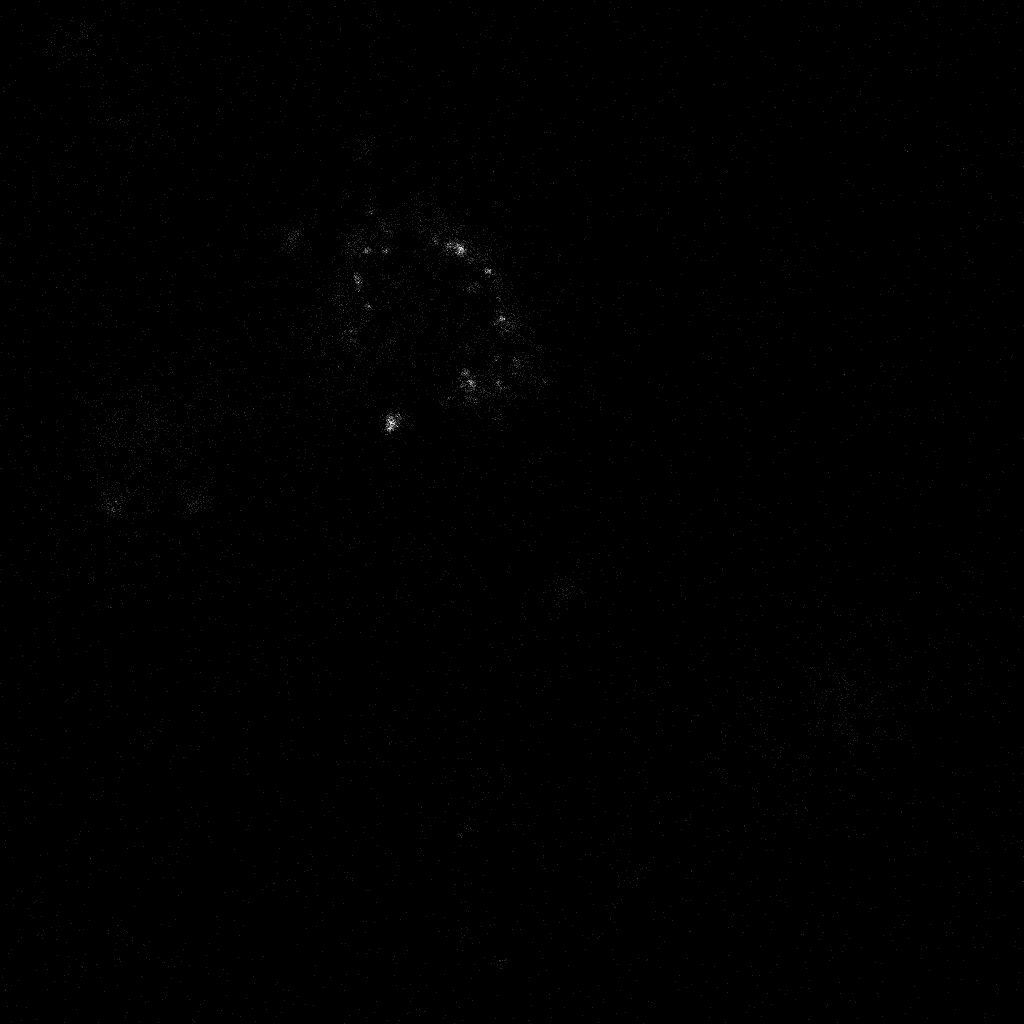

Supplement: Supplementary file 8 — Source data Fig. 1 [file 44319_2025_567_MOESM8_ESM.zip › Fig1/1D/Counted_nuclei/Mechanical/Nuc_15+16+17/Nuc_15+16+17_z47_RAW_ch00.tif]

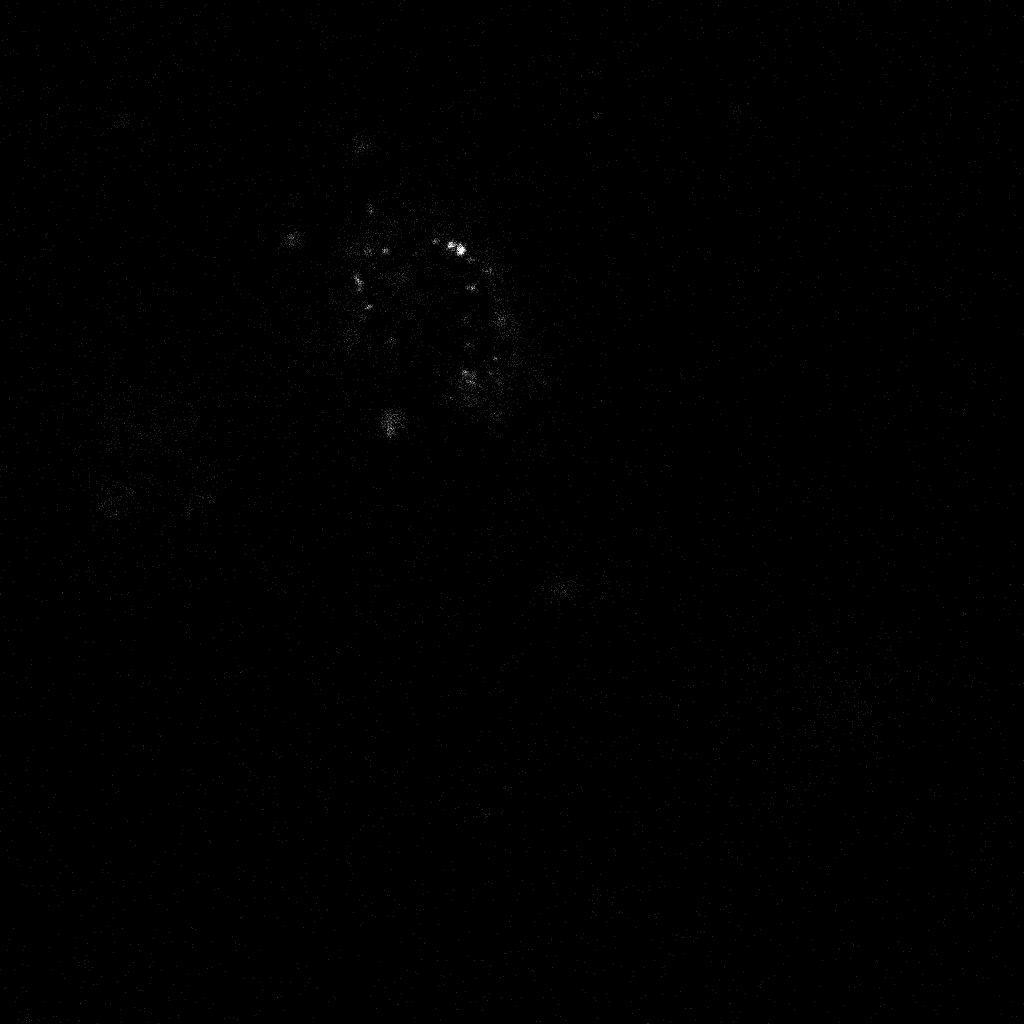

Supplement: Supplementary file 8 — Source data Fig. 1 [file 44319_2025_567_MOESM8_ESM.zip › Fig1/1D/Counted_nuclei/Mechanical/Nuc_15+16+17/Nuc_15+16+17_z48_RAW_ch00.tif]

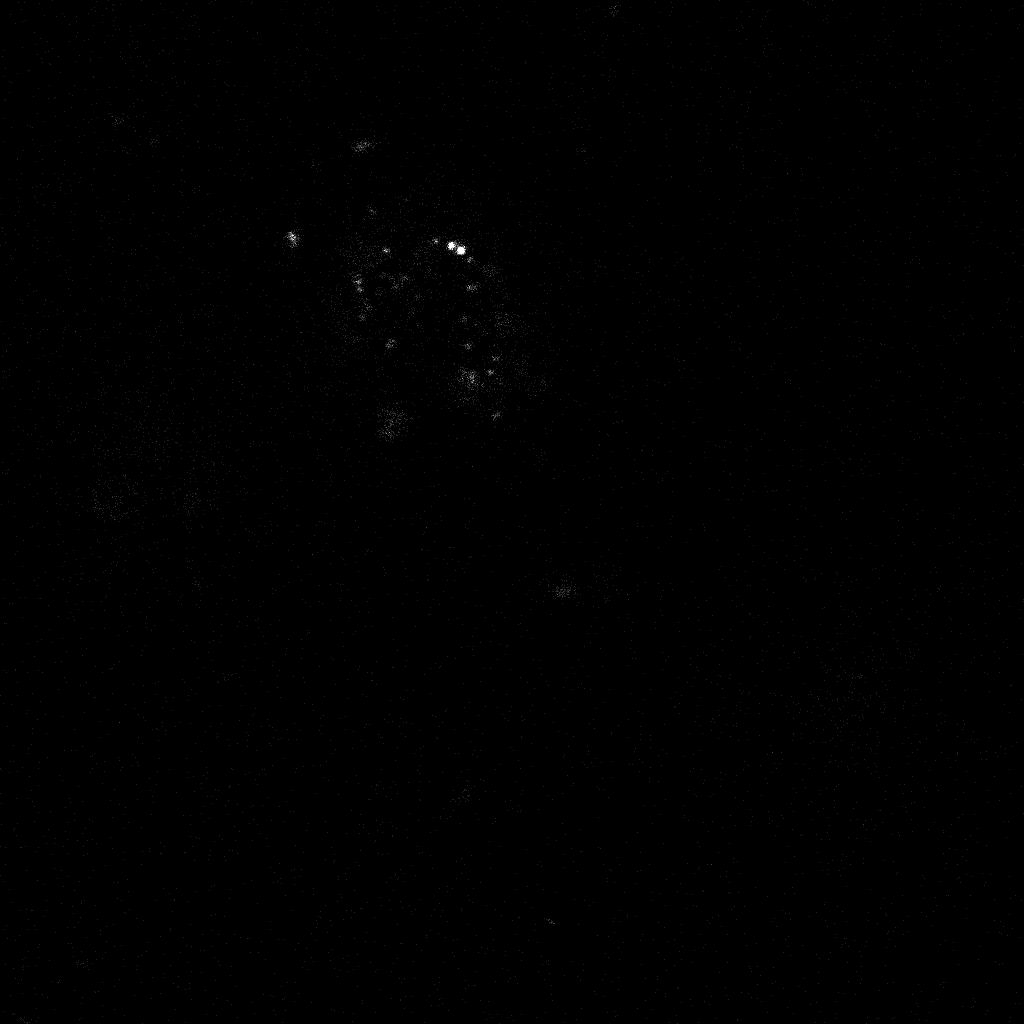

Supplement: Supplementary file 8 — Source data Fig. 1 [file 44319_2025_567_MOESM8_ESM.zip › Fig1/1D/Counted_nuclei/Mechanical/Nuc_15+16+17/Nuc_15+16+17_z49_RAW_ch00.tif]

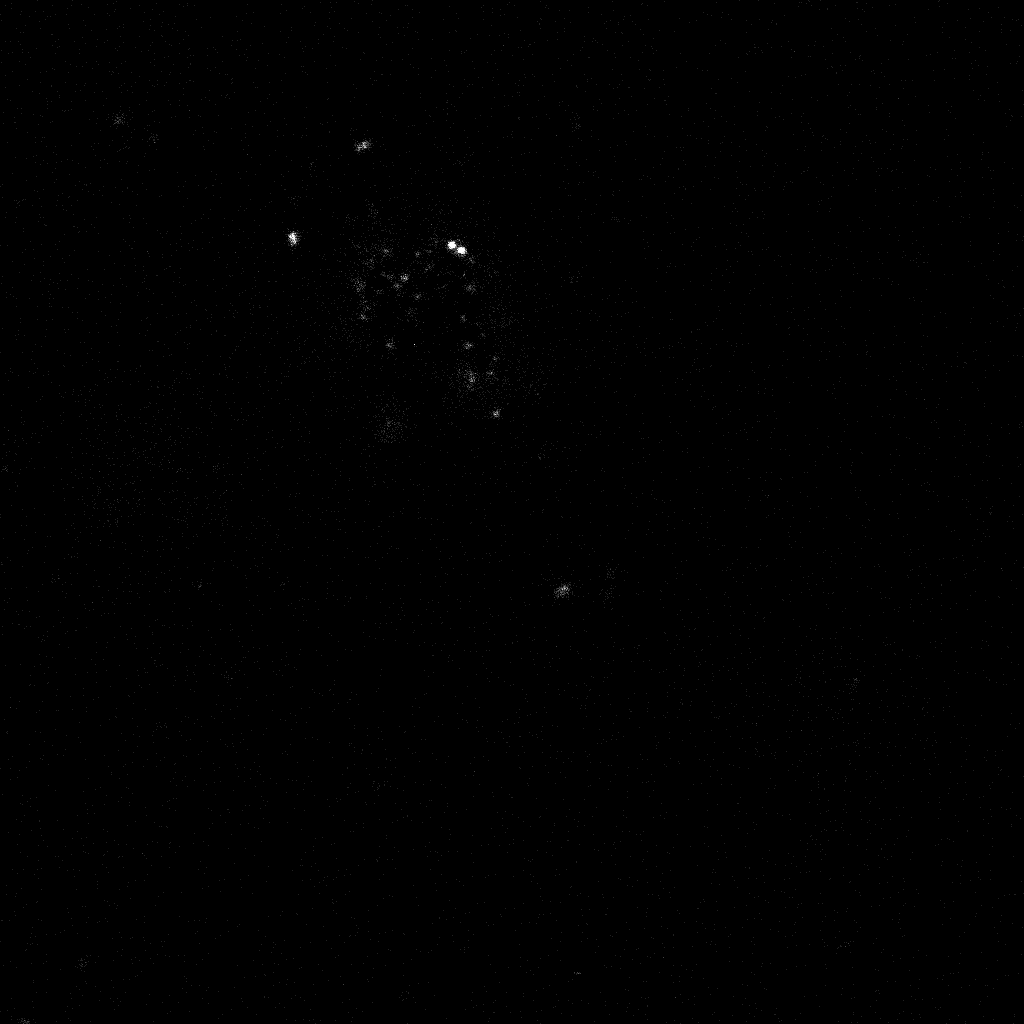

Supplement: Supplementary file 8 — Source data Fig. 1 [file 44319_2025_567_MOESM8_ESM.zip › Fig1/1D/Counted_nuclei/Mechanical/Nuc_15+16+17/Nuc_15+16+17_z50_RAW_ch00.tif]

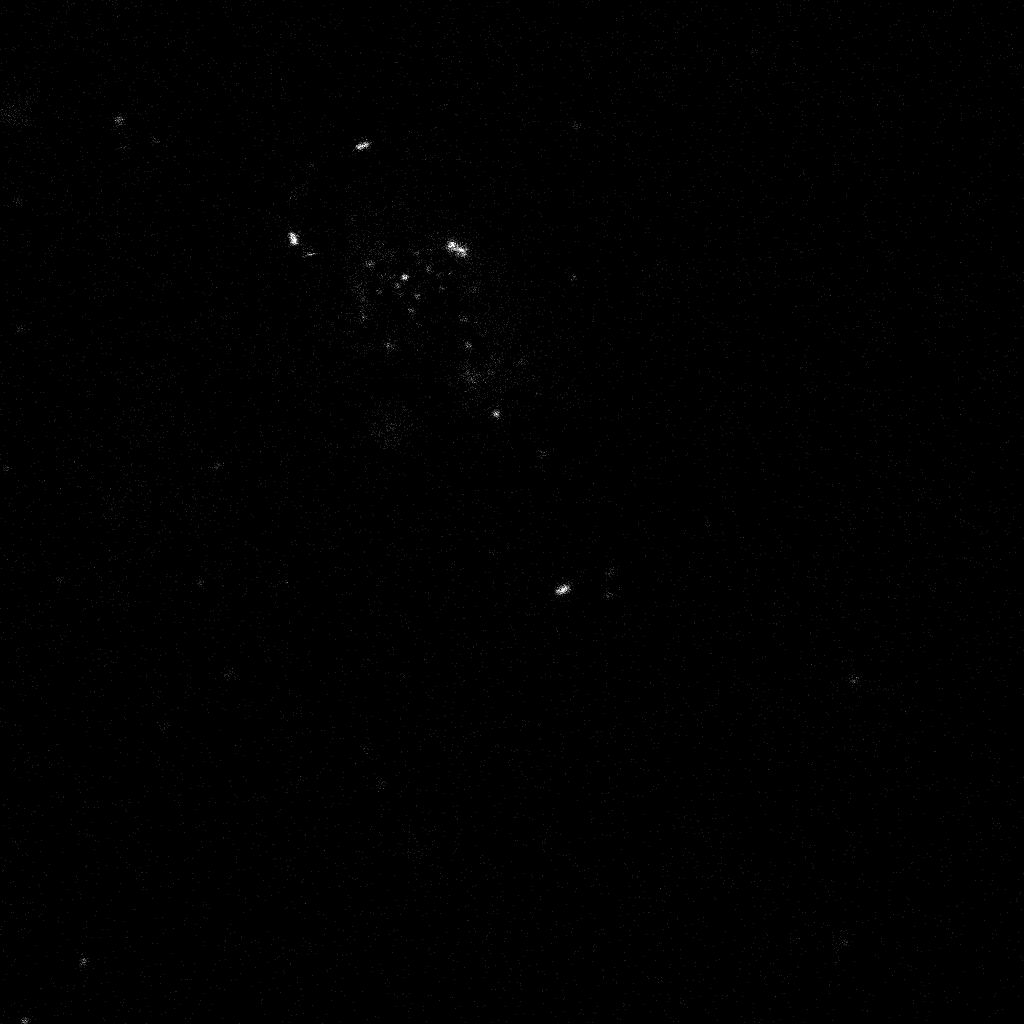

Supplement: Supplementary file 8 — Source data Fig. 1 [file 44319_2025_567_MOESM8_ESM.zip › Fig1/1D/Counted_nuclei/Mechanical/Nuc_15+16+17/Nuc_15+16+17_z51_RAW_ch00.tif]

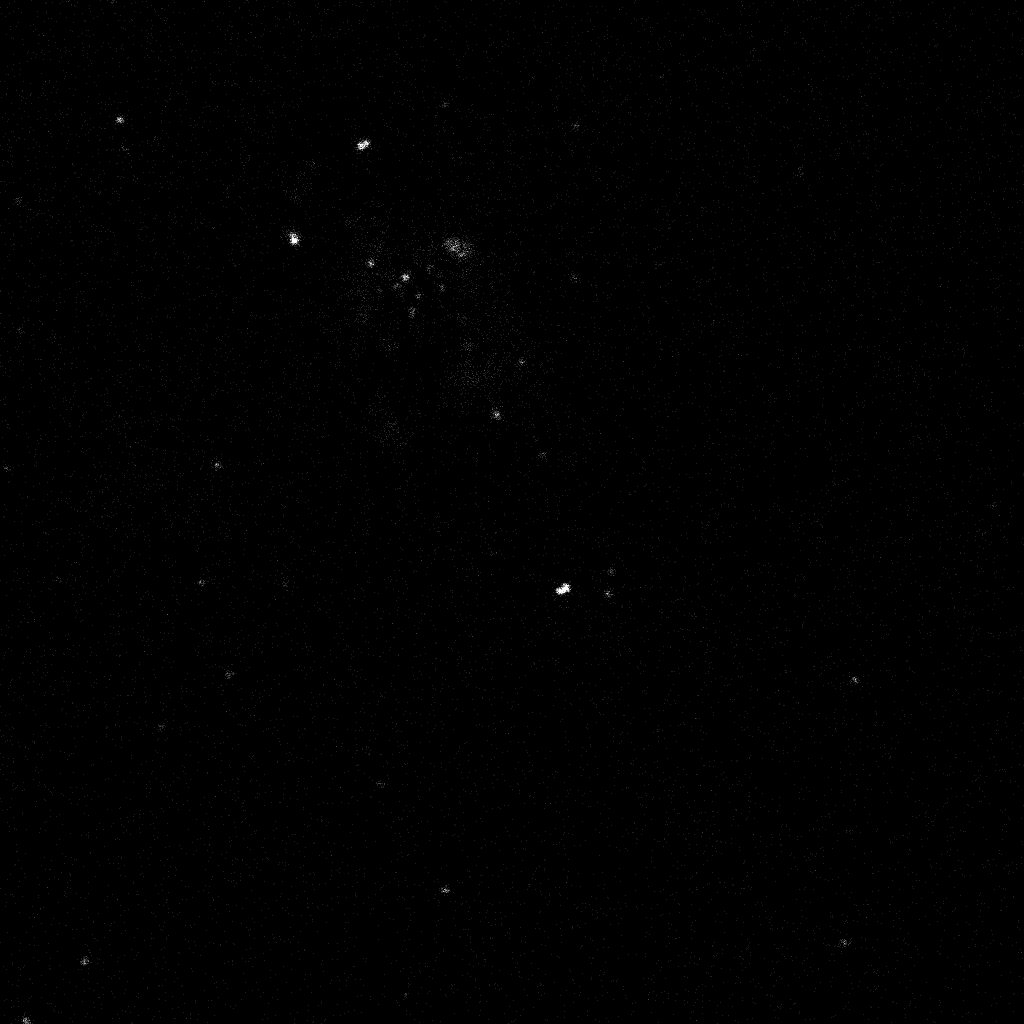

Supplement: Supplementary file 8 — Source data Fig. 1 [file 44319_2025_567_MOESM8_ESM.zip › Fig1/1D/Counted_nuclei/Mechanical/Nuc_15+16+17/Nuc_15+16+17_z52_RAW_ch00.tif]

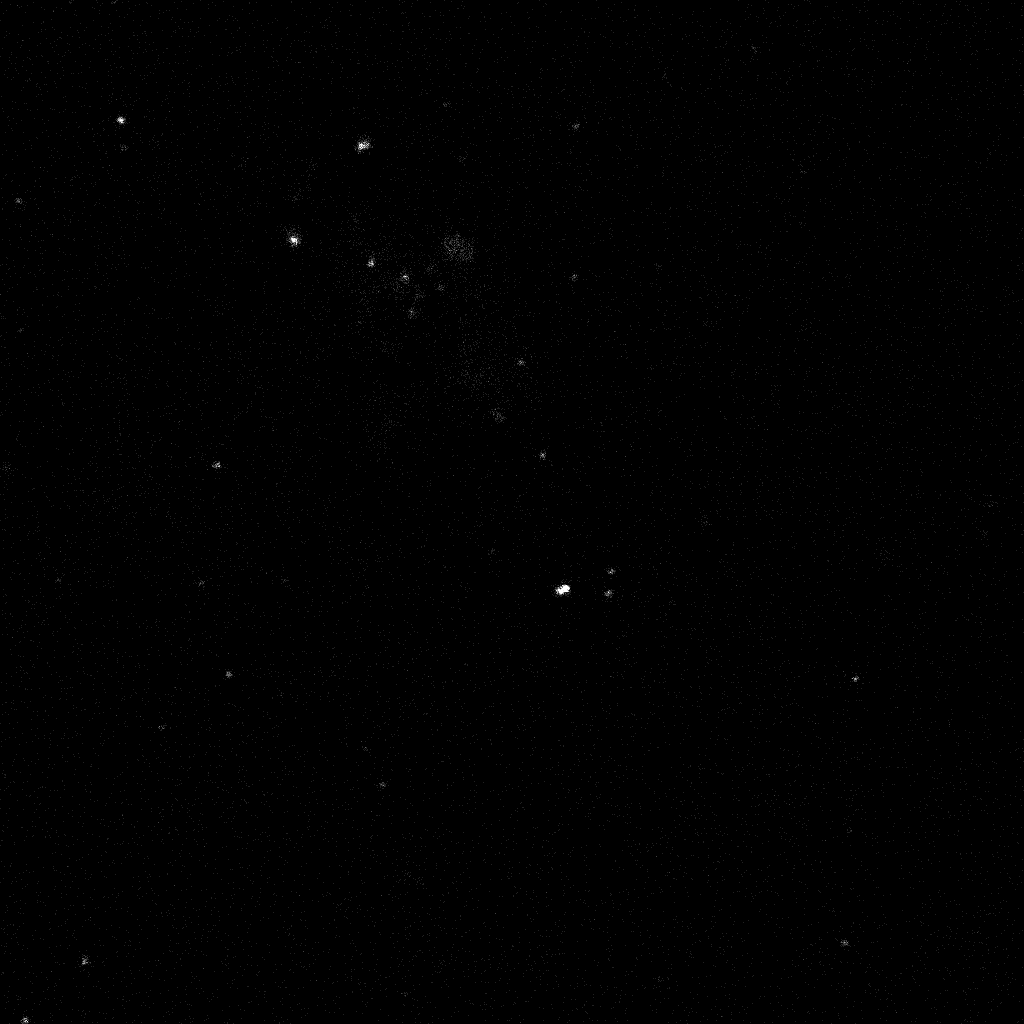

Supplement: Supplementary file 8 — Source data Fig. 1 [file 44319_2025_567_MOESM8_ESM.zip › Fig1/1D/Counted_nuclei/Mechanical/Nuc_15+16+17/Nuc_15+16+17_z53_RAW_ch00.tif]

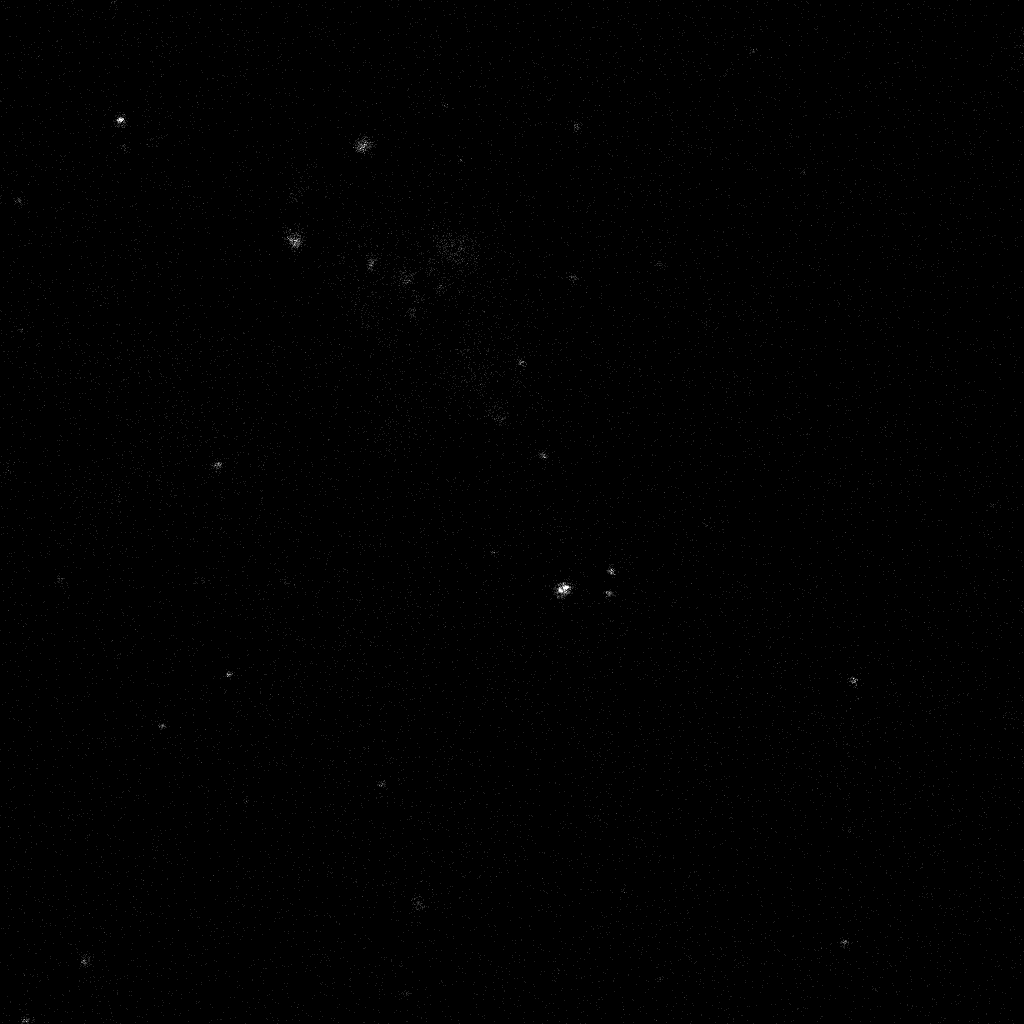

Supplement: Supplementary file 8 — Source data Fig. 1 [file 44319_2025_567_MOESM8_ESM.zip › Fig1/1D/Counted_nuclei/Mechanical/Nuc_15+16+17/Nuc_15+16+17_z54_RAW_ch00.tif]

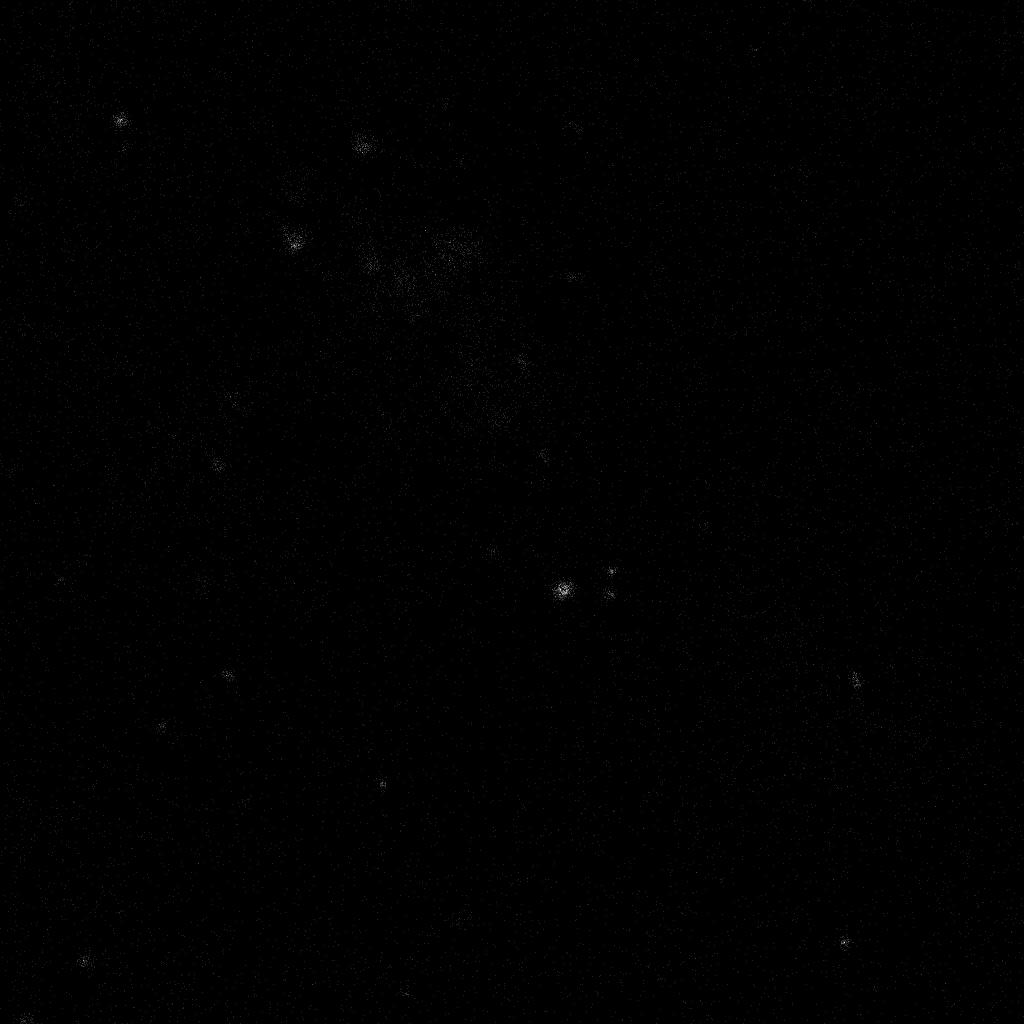

Supplement: Supplementary file 8 — Source data Fig. 1 [file 44319_2025_567_MOESM8_ESM.zip › Fig1/1D/Counted_nuclei/Mechanical/Nuc_15+16+17/Nuc_15+16+17_z55_RAW_ch00.tif]

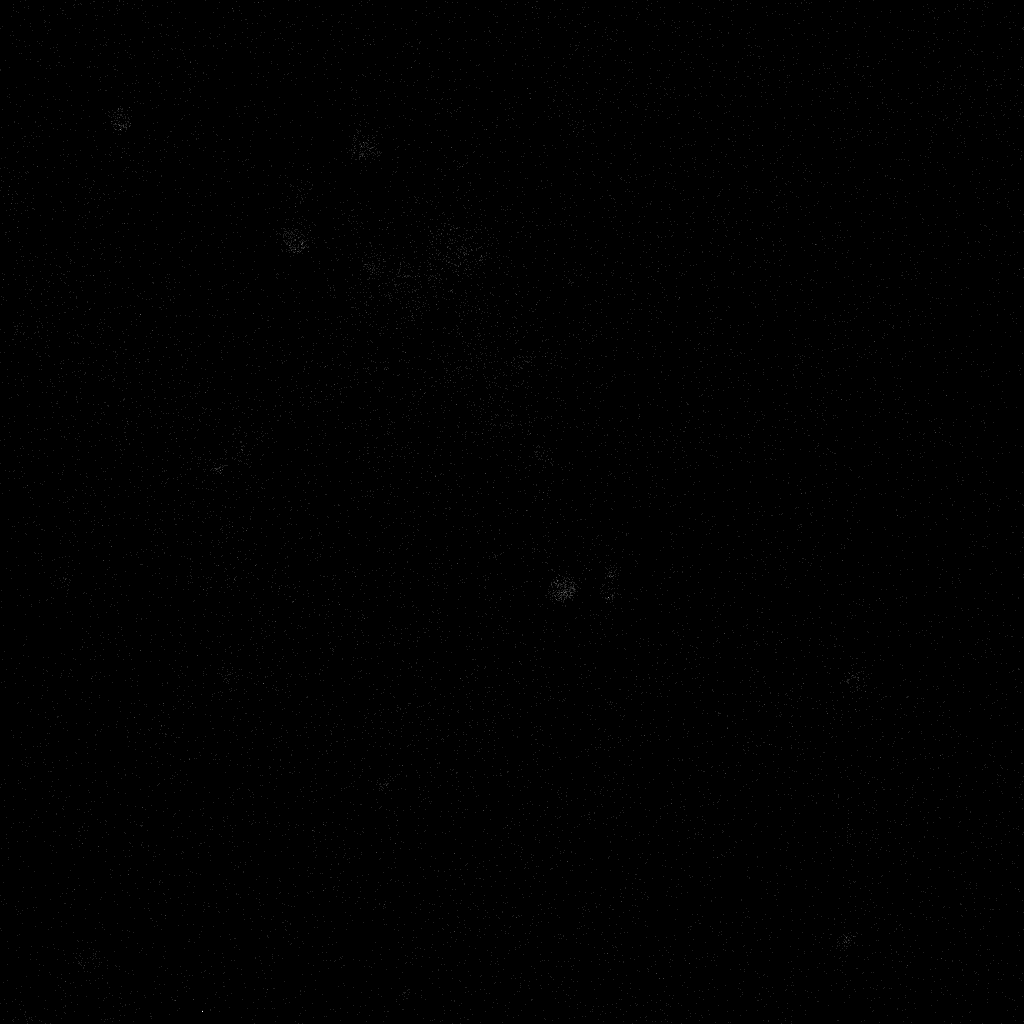

Supplement: Supplementary file 8 — Source data Fig. 1 [file 44319_2025_567_MOESM8_ESM.zip › Fig1/1D/Counted_nuclei/Mechanical/Nuc_15+16+17/Nuc_15+16+17_z56_RAW_ch00.tif]

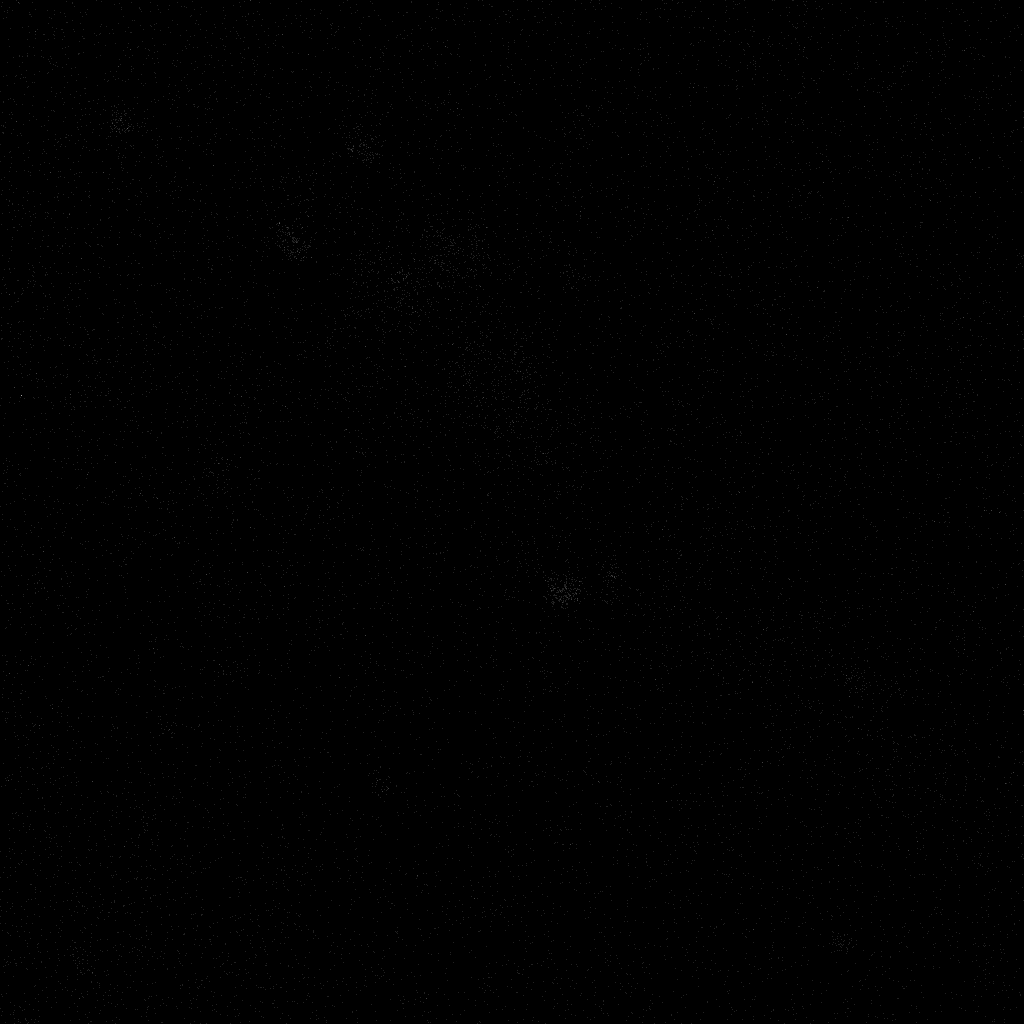

Supplement: Supplementary file 8 — Source data Fig. 1 [file 44319_2025_567_MOESM8_ESM.zip › Fig1/1D/Counted_nuclei/Mechanical/Nuc_15+16+17/Nuc_15+16+17_z57_RAW_ch00.tif]

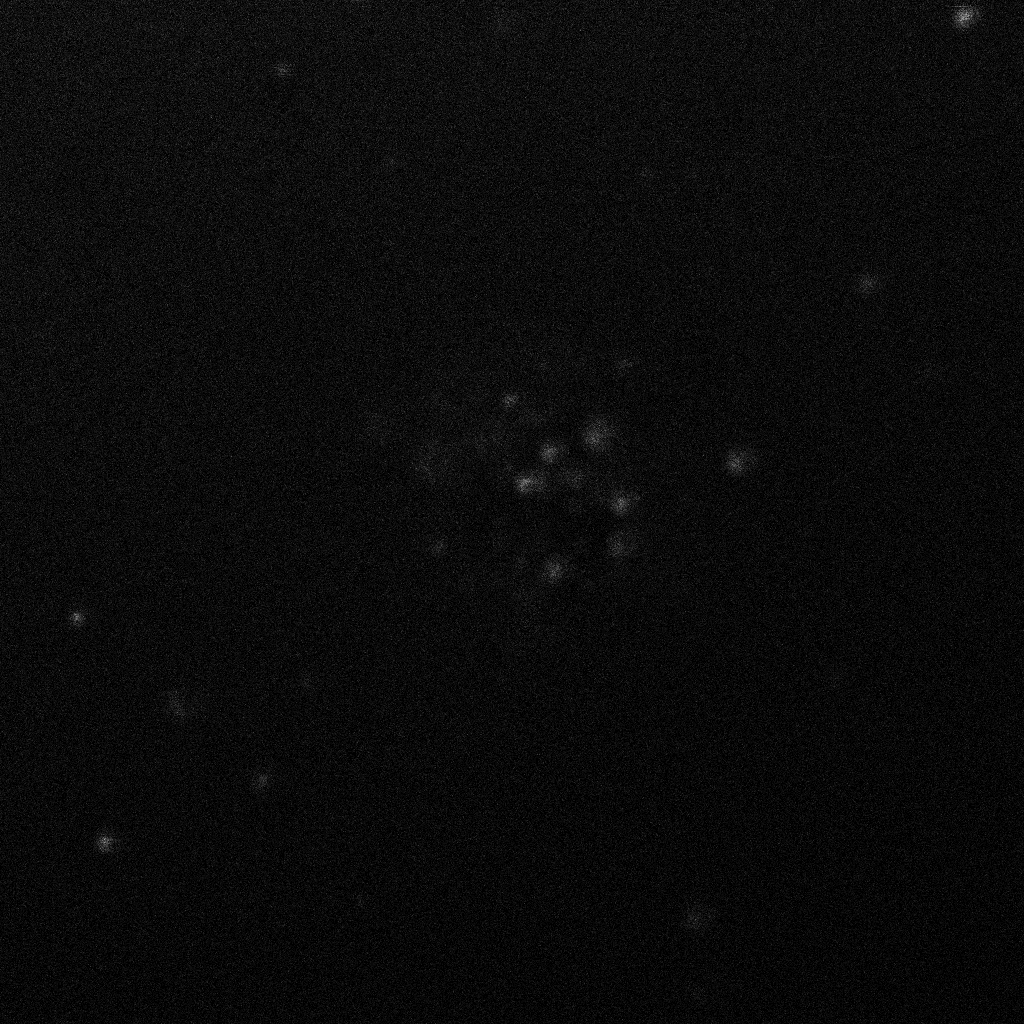

Supplement: Supplementary file 8 — Source data Fig. 1 [file 44319_2025_567_MOESM8_ESM.zip › Fig1/1D/Counted_nuclei/Mechanical/Nuc_18/Nuc_18_z00_RAW_ch00.tif]

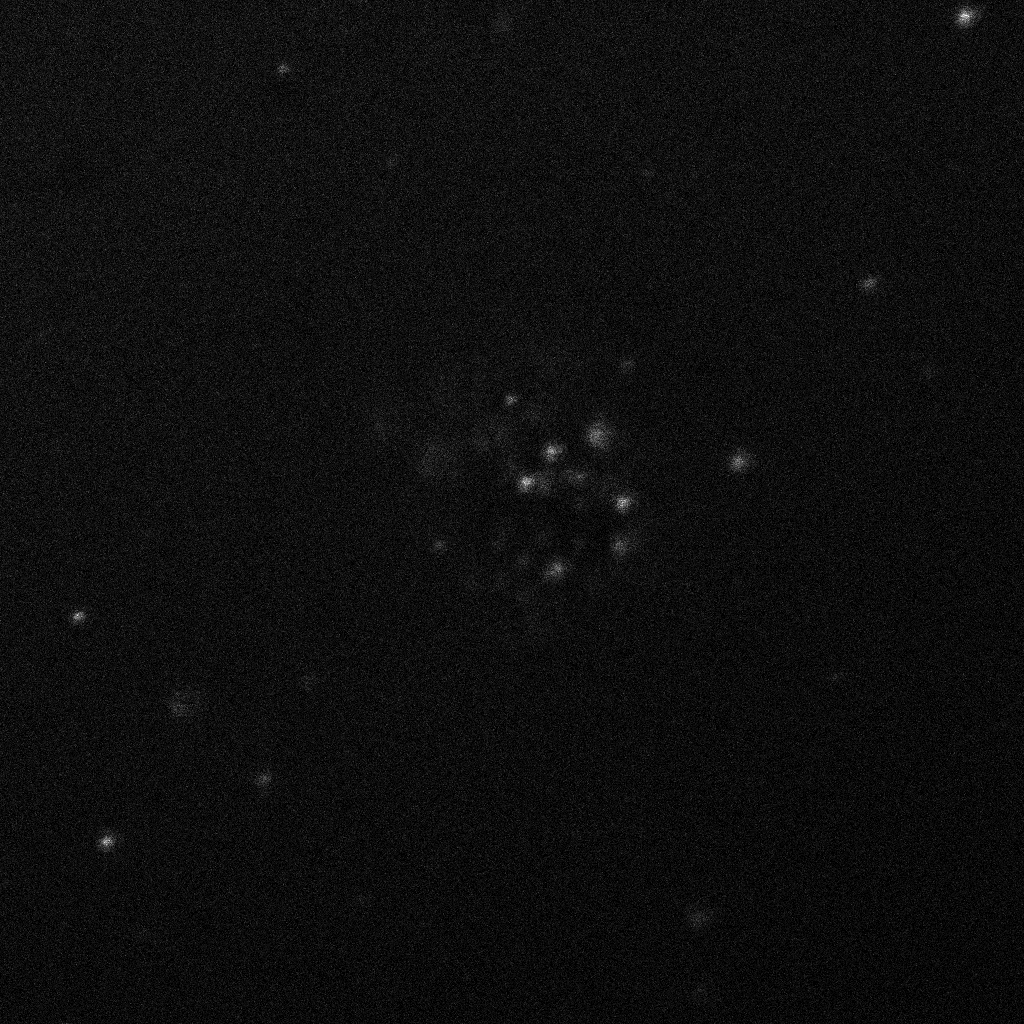

Supplement: Supplementary file 8 — Source data Fig. 1 [file 44319_2025_567_MOESM8_ESM.zip › Fig1/1D/Counted_nuclei/Mechanical/Nuc_18/Nuc_18_z01_RAW_ch00.tif]

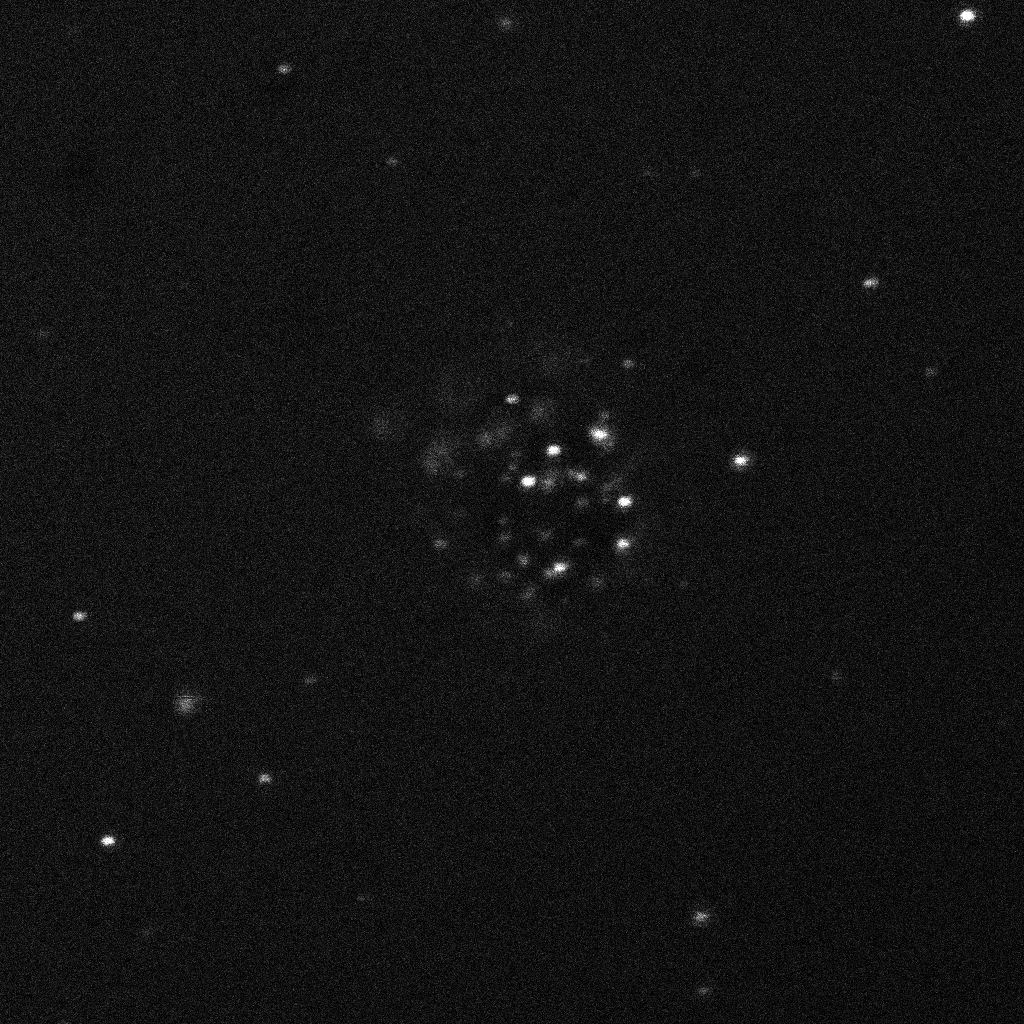

Supplement: Supplementary file 8 — Source data Fig. 1 [file 44319_2025_567_MOESM8_ESM.zip › Fig1/1D/Counted_nuclei/Mechanical/Nuc_18/Nuc_18_z02_RAW_ch00.tif]

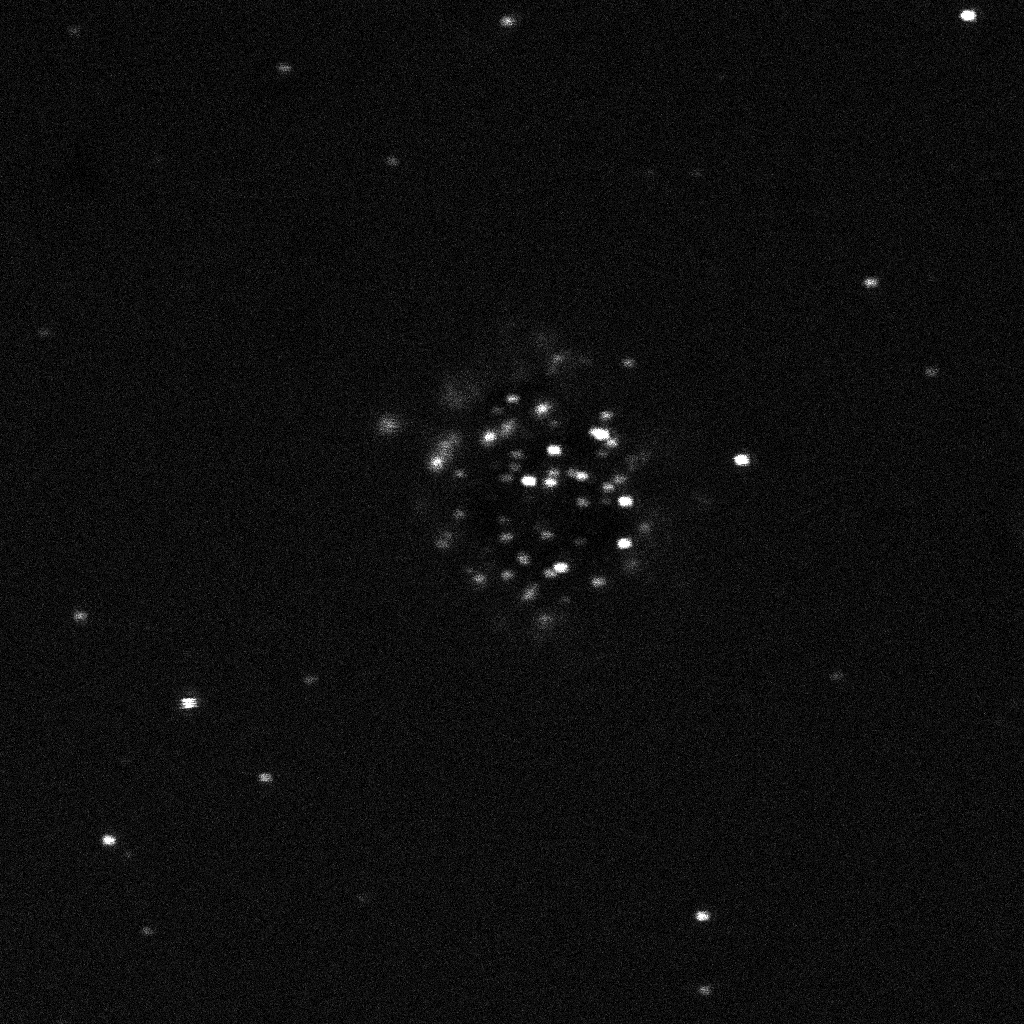

Supplement: Supplementary file 8 — Source data Fig. 1 [file 44319_2025_567_MOESM8_ESM.zip › Fig1/1D/Counted_nuclei/Mechanical/Nuc_18/Nuc_18_z03_RAW_ch00.tif]

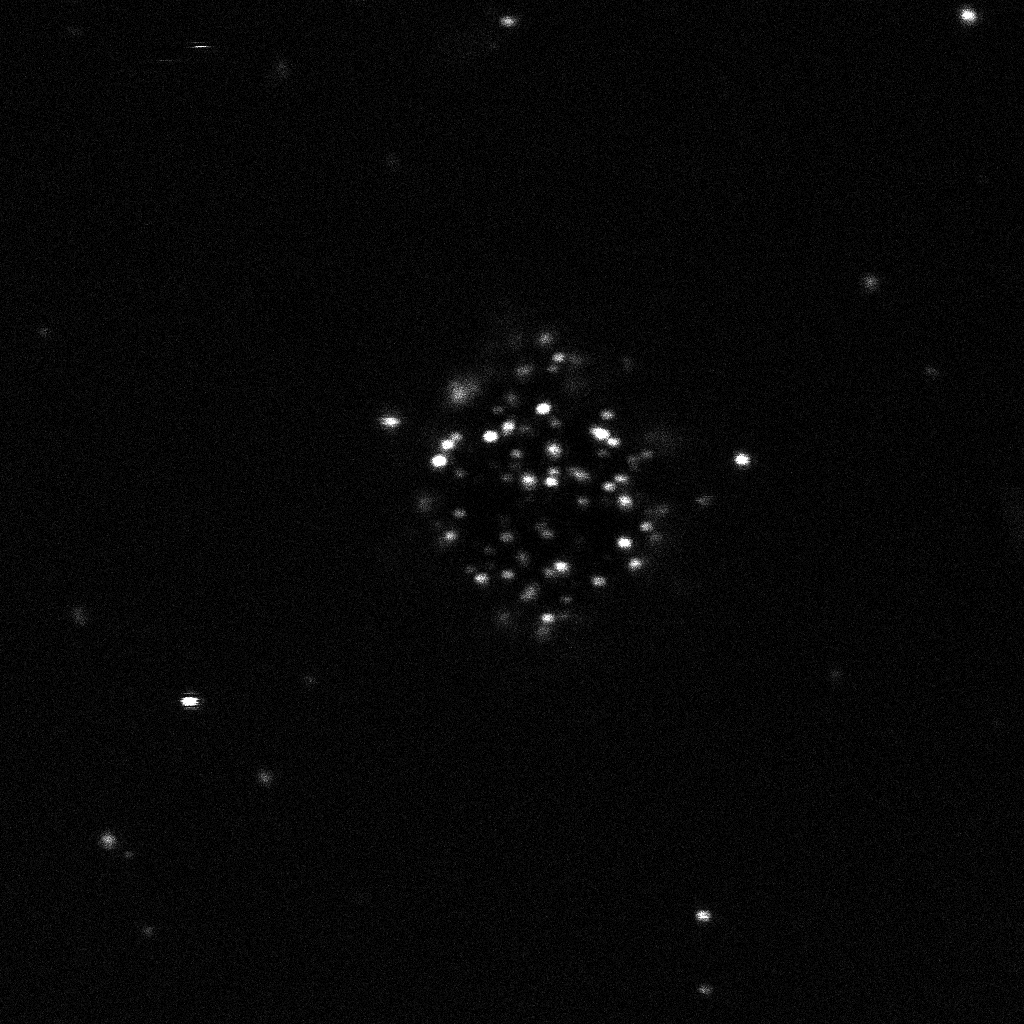

Supplement: Supplementary file 8 — Source data Fig. 1 [file 44319_2025_567_MOESM8_ESM.zip › Fig1/1D/Counted_nuclei/Mechanical/Nuc_18/Nuc_18_z04_RAW_ch00.tif]

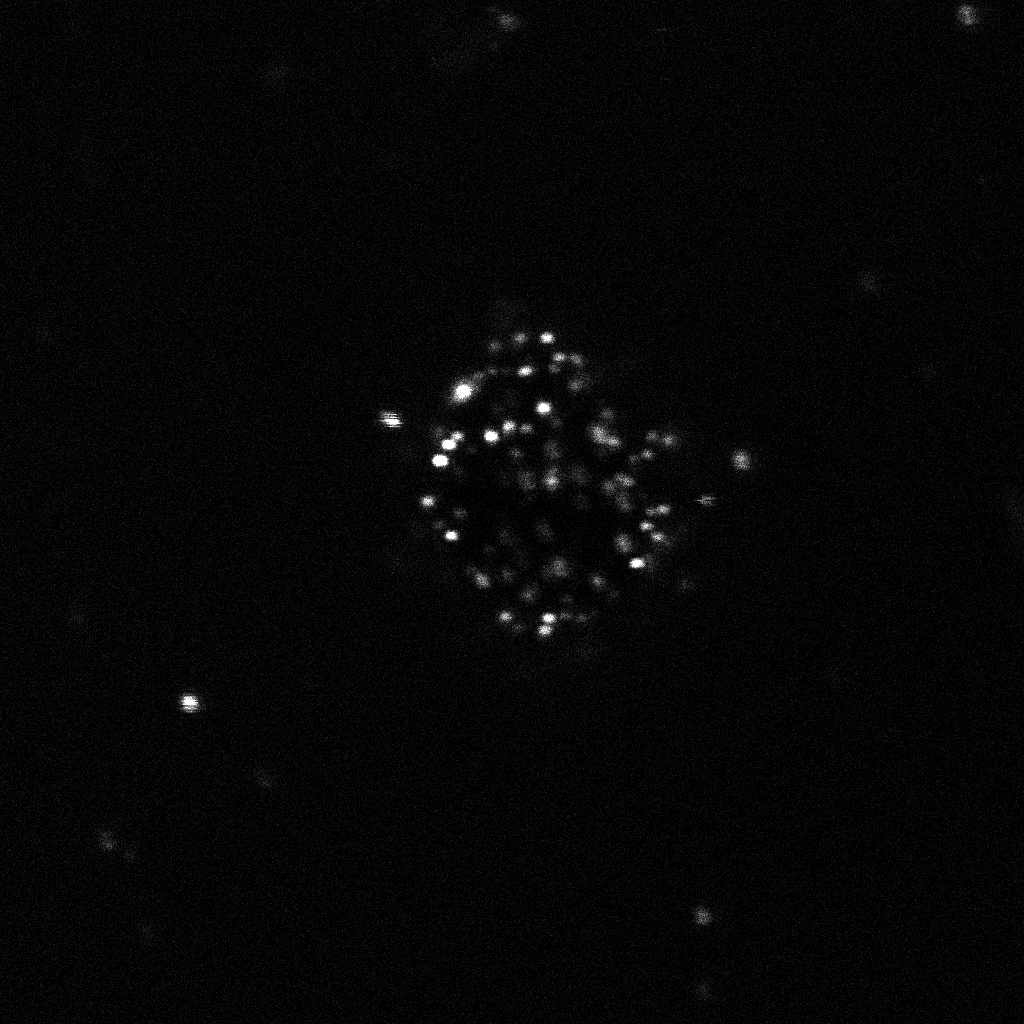

Supplement: Supplementary file 8 — Source data Fig. 1 [file 44319_2025_567_MOESM8_ESM.zip › Fig1/1D/Counted_nuclei/Mechanical/Nuc_18/Nuc_18_z05_RAW_ch00.tif]

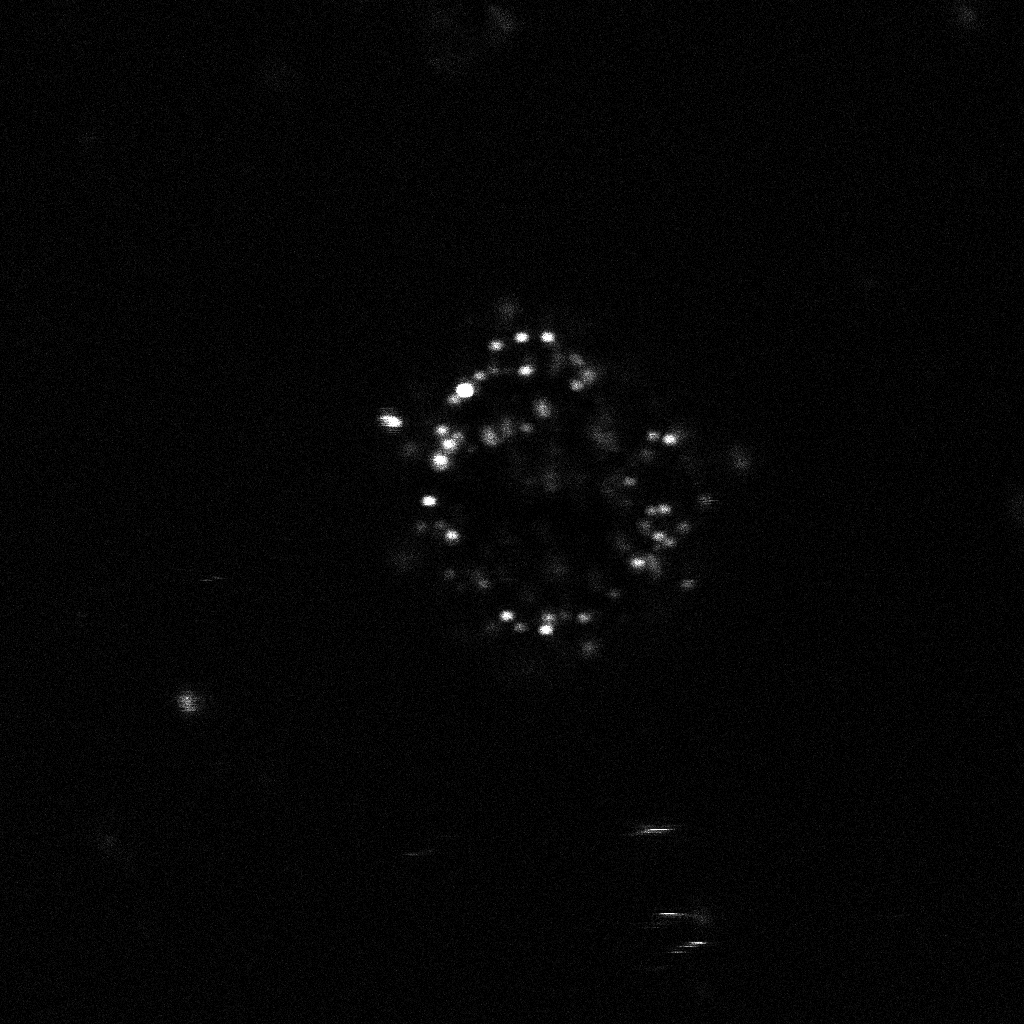

Supplement: Supplementary file 8 — Source data Fig. 1 [file 44319_2025_567_MOESM8_ESM.zip › Fig1/1D/Counted_nuclei/Mechanical/Nuc_18/Nuc_18_z06_RAW_ch00.tif]

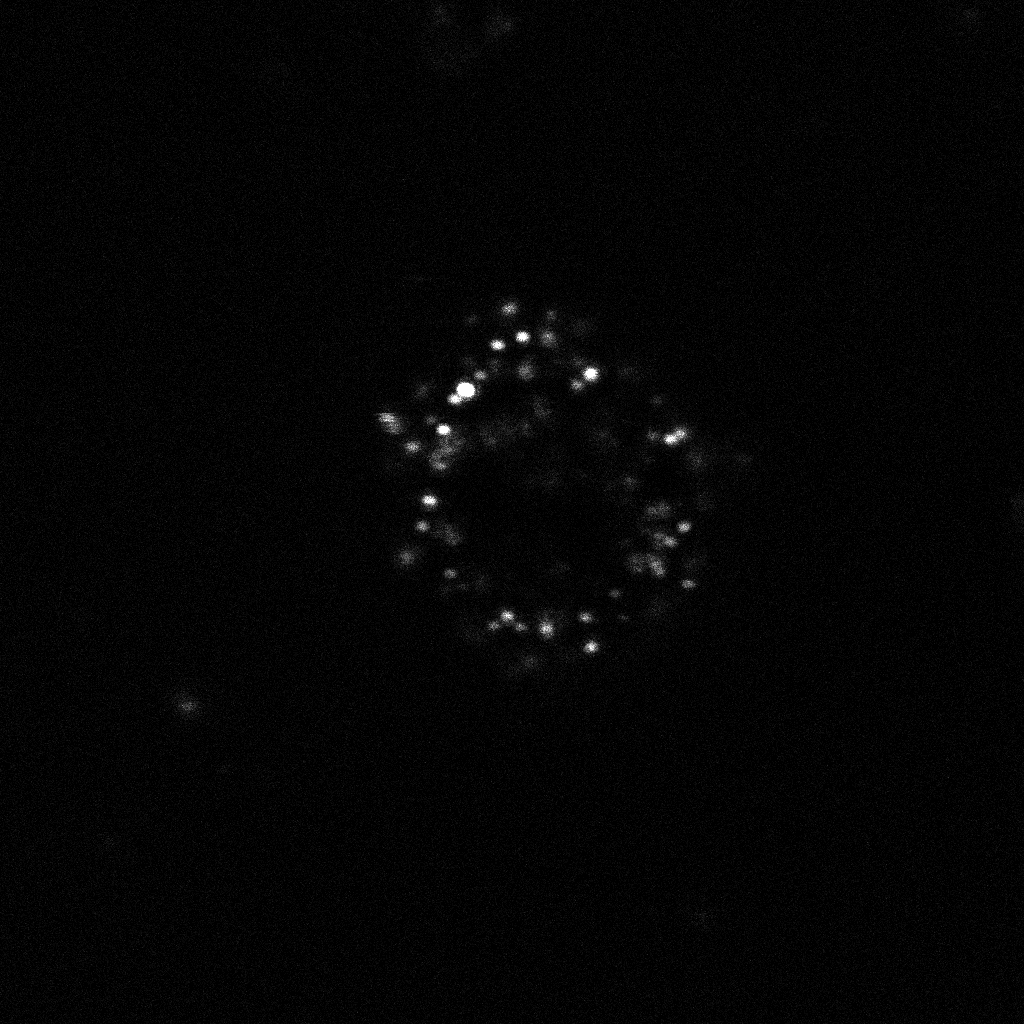

Supplement: Supplementary file 8 — Source data Fig. 1 [file 44319_2025_567_MOESM8_ESM.zip › Fig1/1D/Counted_nuclei/Mechanical/Nuc_18/Nuc_18_z07_RAW_ch00.tif]

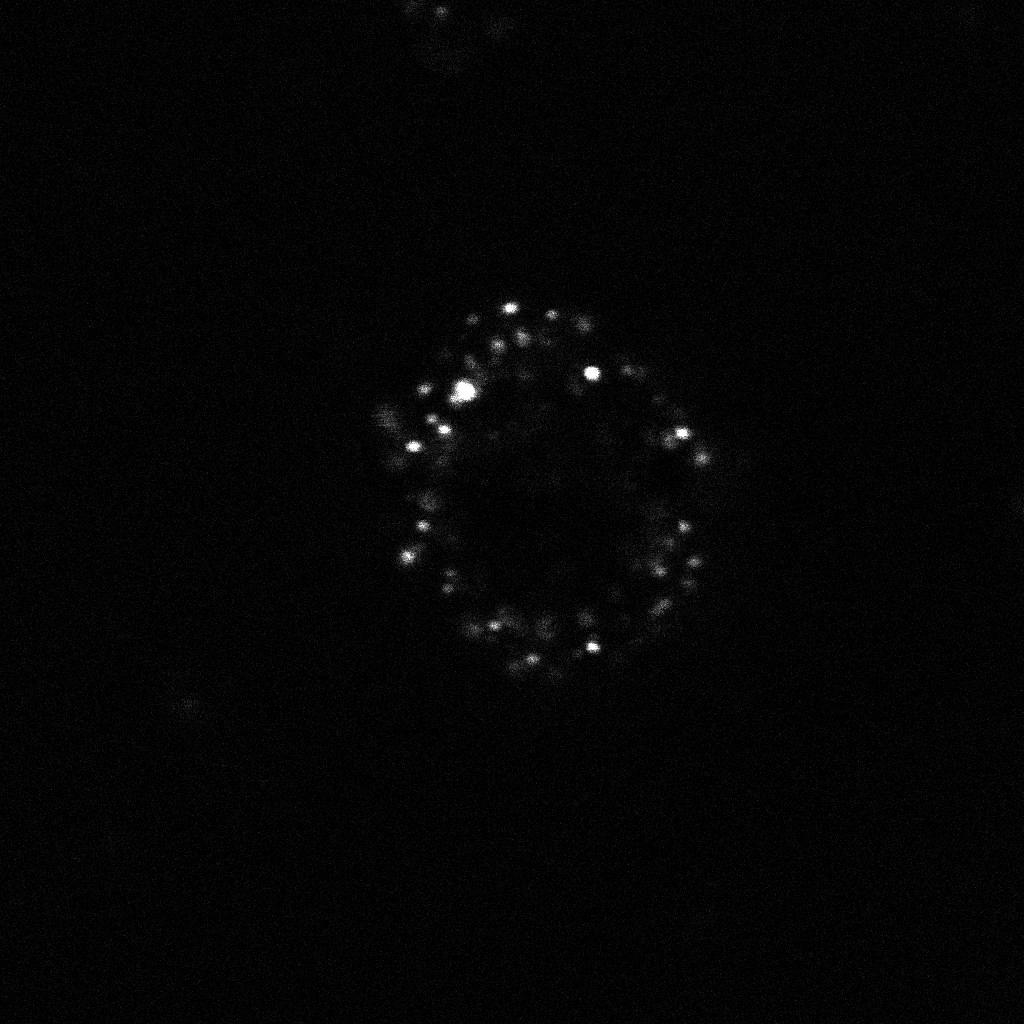

Supplement: Supplementary file 8 — Source data Fig. 1 [file 44319_2025_567_MOESM8_ESM.zip › Fig1/1D/Counted_nuclei/Mechanical/Nuc_18/Nuc_18_z08_RAW_ch00.tif]

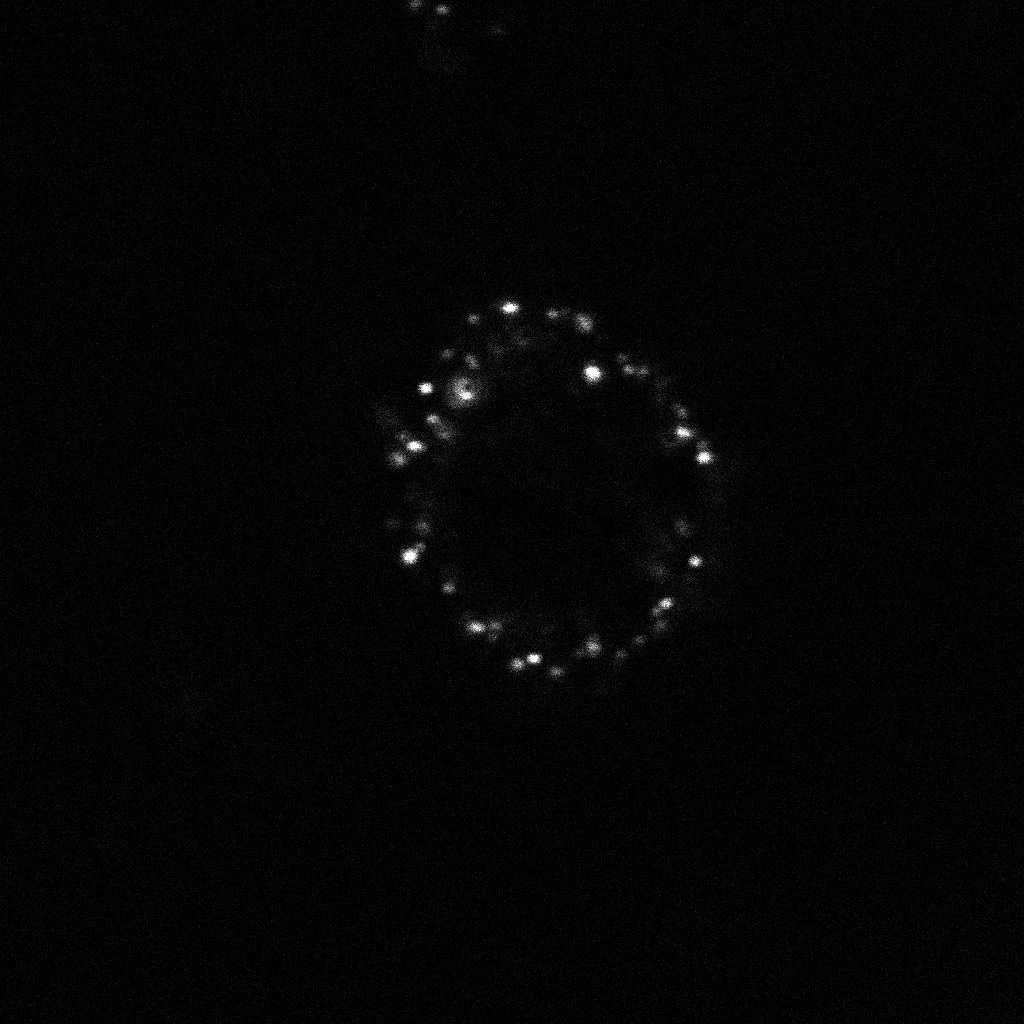

Supplement: Supplementary file 8 — Source data Fig. 1 [file 44319_2025_567_MOESM8_ESM.zip › Fig1/1D/Counted_nuclei/Mechanical/Nuc_18/Nuc_18_z09_RAW_ch00.tif]

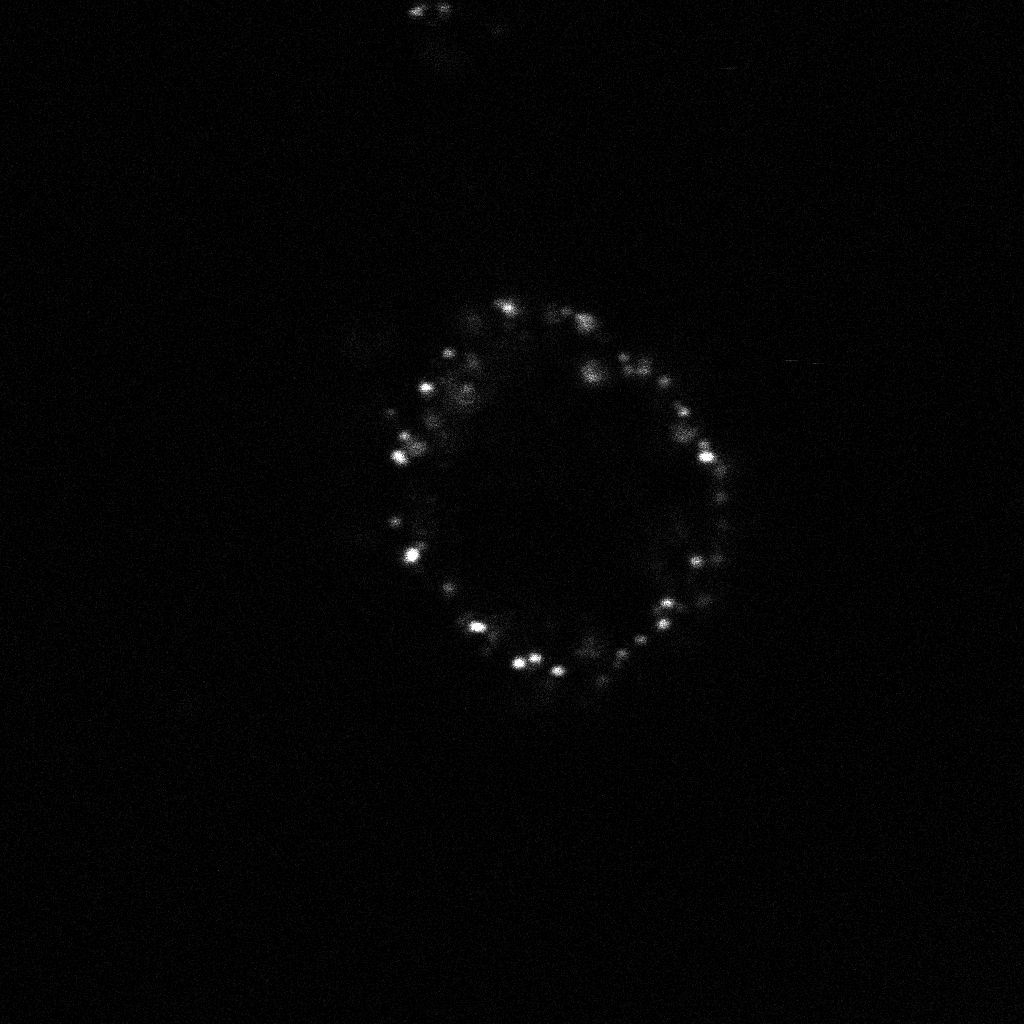

Supplement: Supplementary file 8 — Source data Fig. 1 [file 44319_2025_567_MOESM8_ESM.zip › Fig1/1D/Counted_nuclei/Mechanical/Nuc_18/Nuc_18_z10_RAW_ch00.tif]

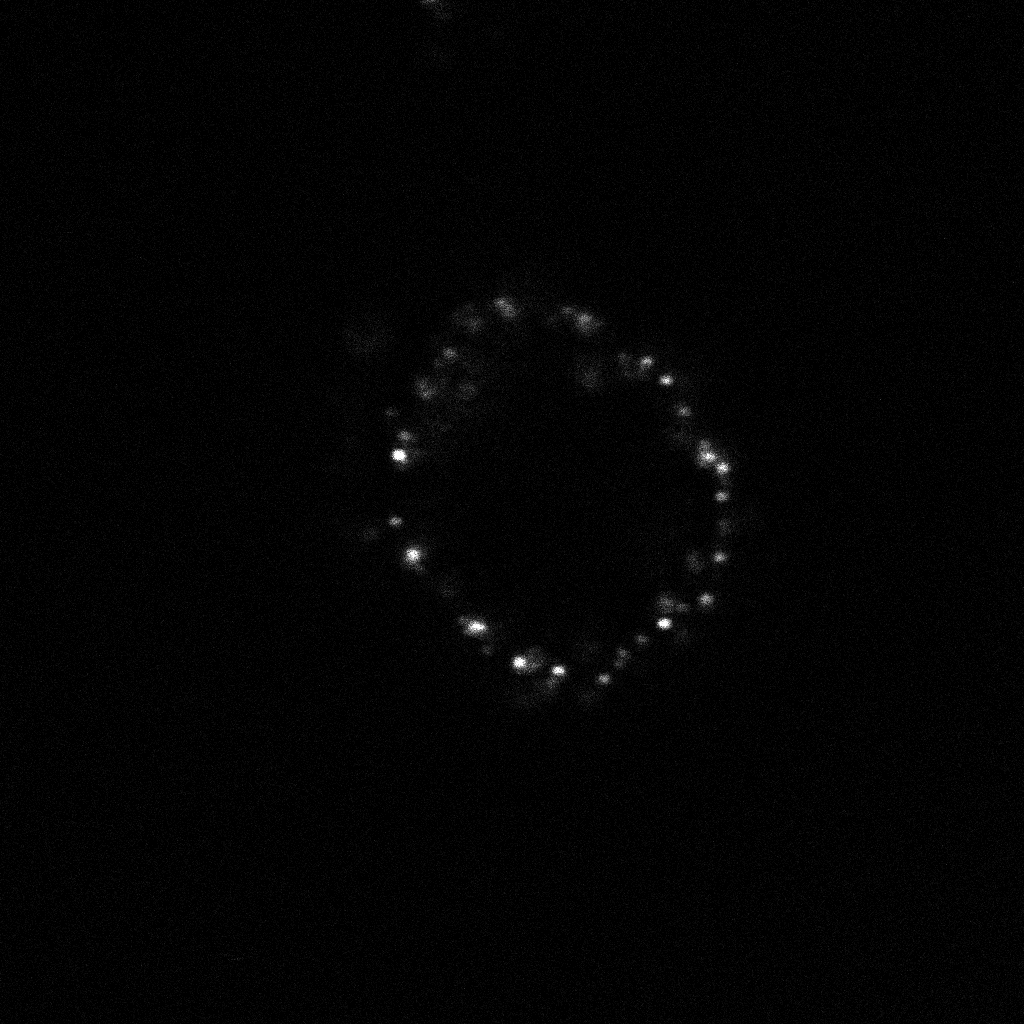

Supplement: Supplementary file 8 — Source data Fig. 1 [file 44319_2025_567_MOESM8_ESM.zip › Fig1/1D/Counted_nuclei/Mechanical/Nuc_18/Nuc_18_z11_RAW_ch00.tif]

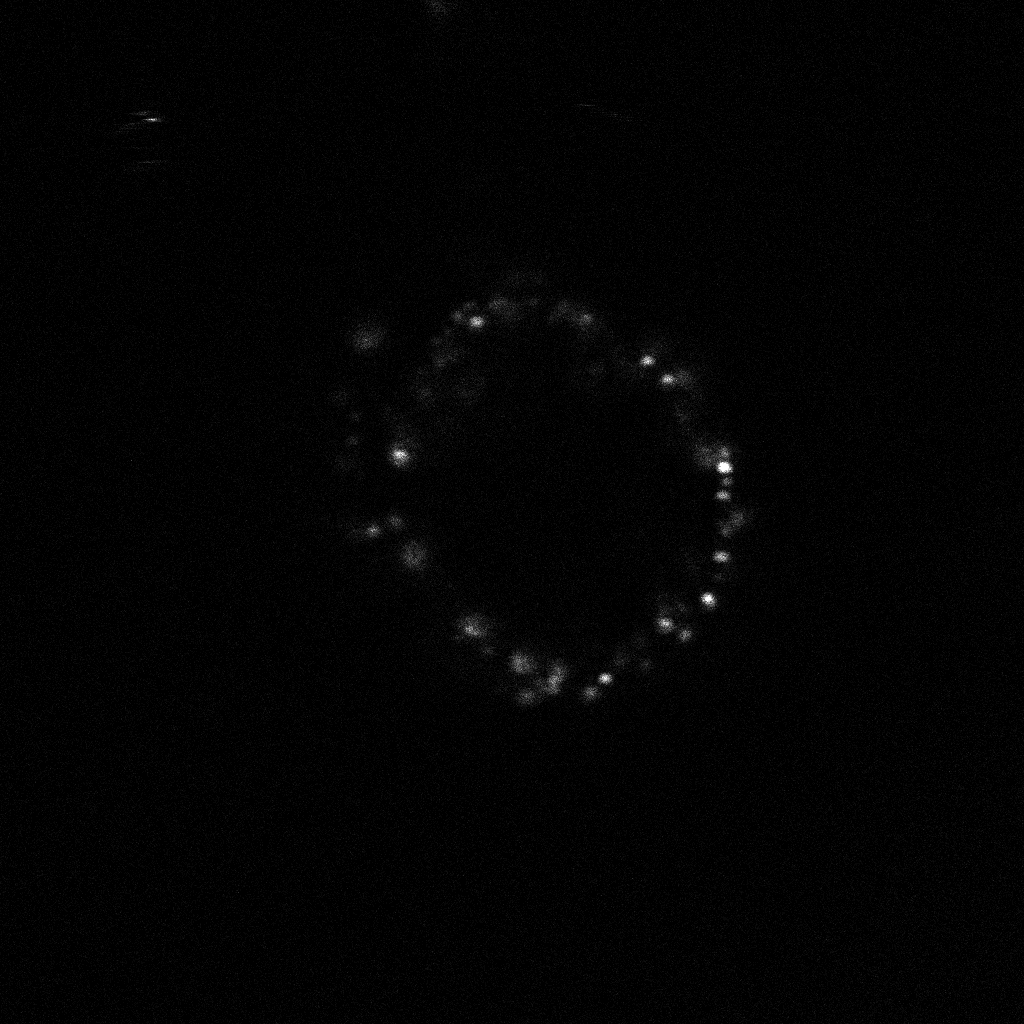

Supplement: Supplementary file 8 — Source data Fig. 1 [file 44319_2025_567_MOESM8_ESM.zip › Fig1/1D/Counted_nuclei/Mechanical/Nuc_18/Nuc_18_z12_RAW_ch00.tif]

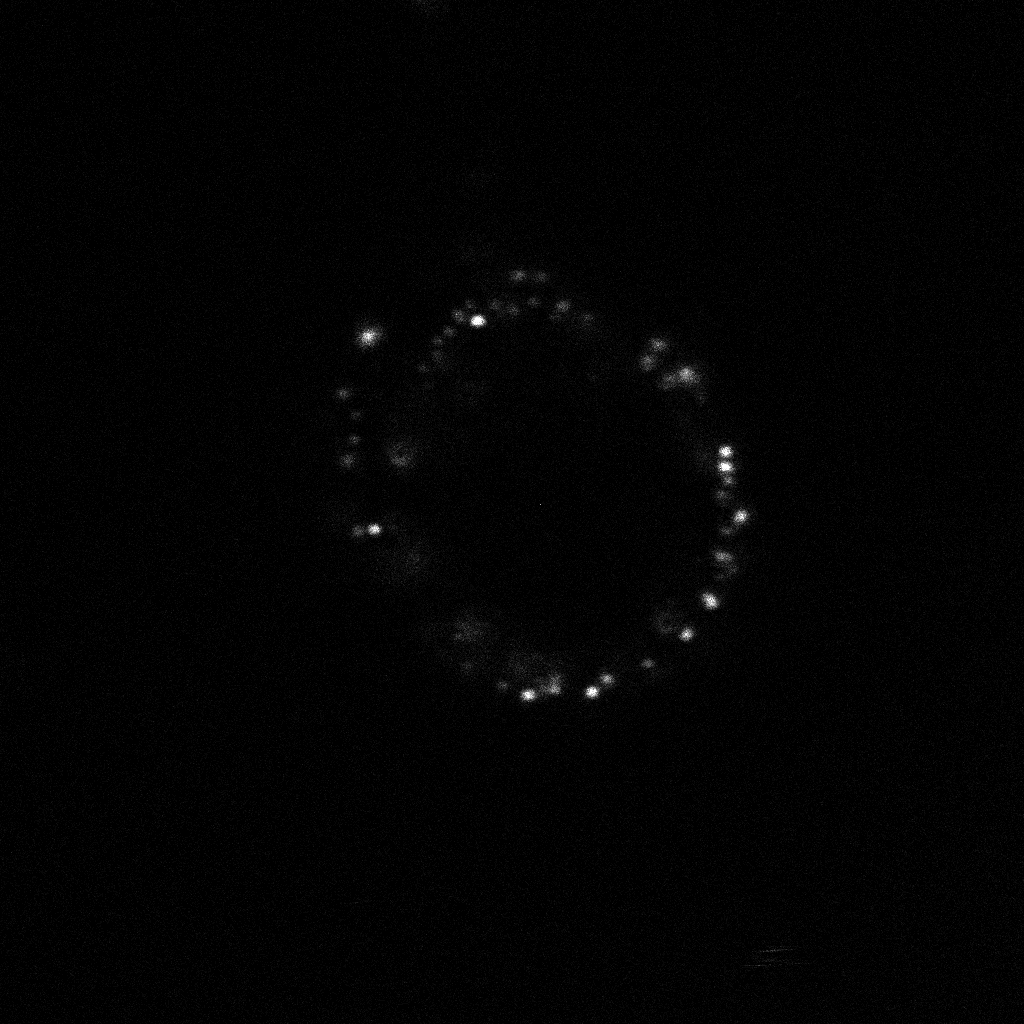

Supplement: Supplementary file 8 — Source data Fig. 1 [file 44319_2025_567_MOESM8_ESM.zip › Fig1/1D/Counted_nuclei/Mechanical/Nuc_18/Nuc_18_z13_RAW_ch00.tif]

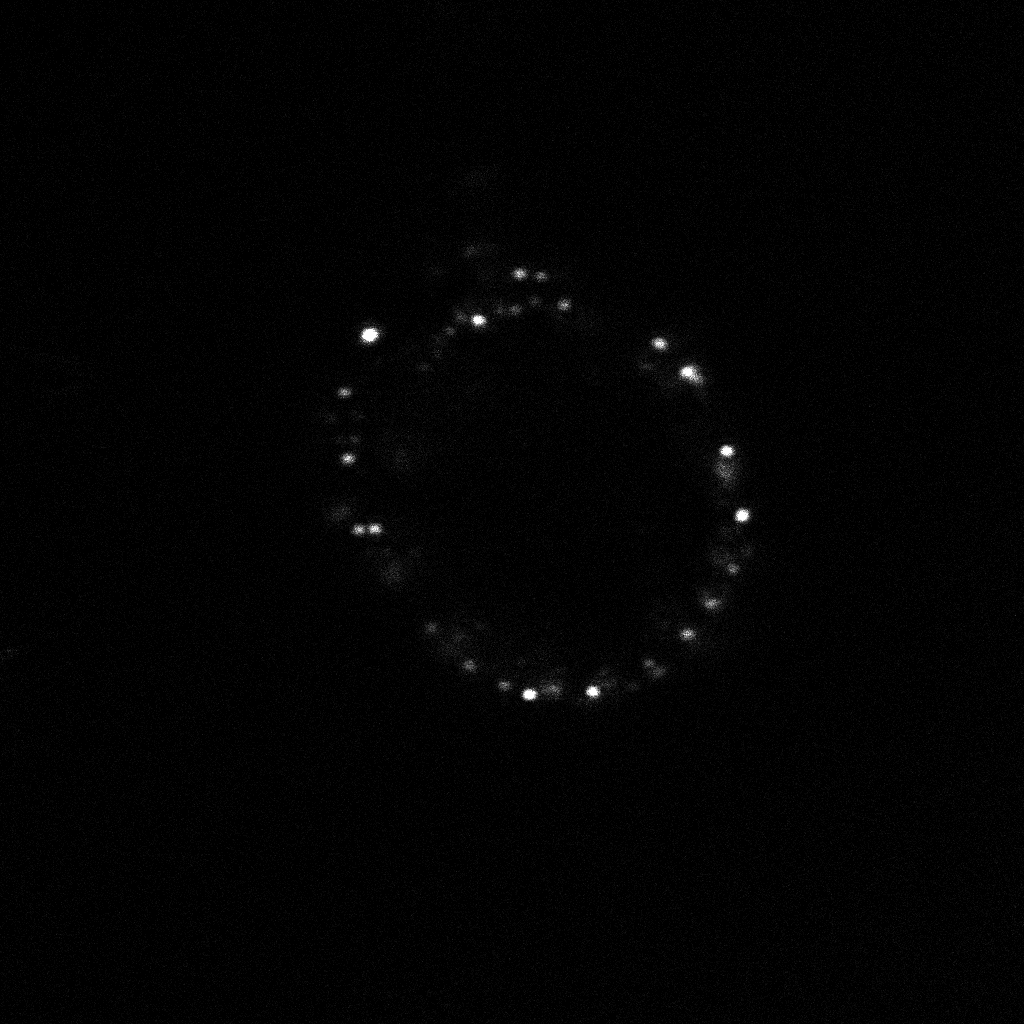

Supplement: Supplementary file 8 — Source data Fig. 1 [file 44319_2025_567_MOESM8_ESM.zip › Fig1/1D/Counted_nuclei/Mechanical/Nuc_18/Nuc_18_z14_RAW_ch00.tif]

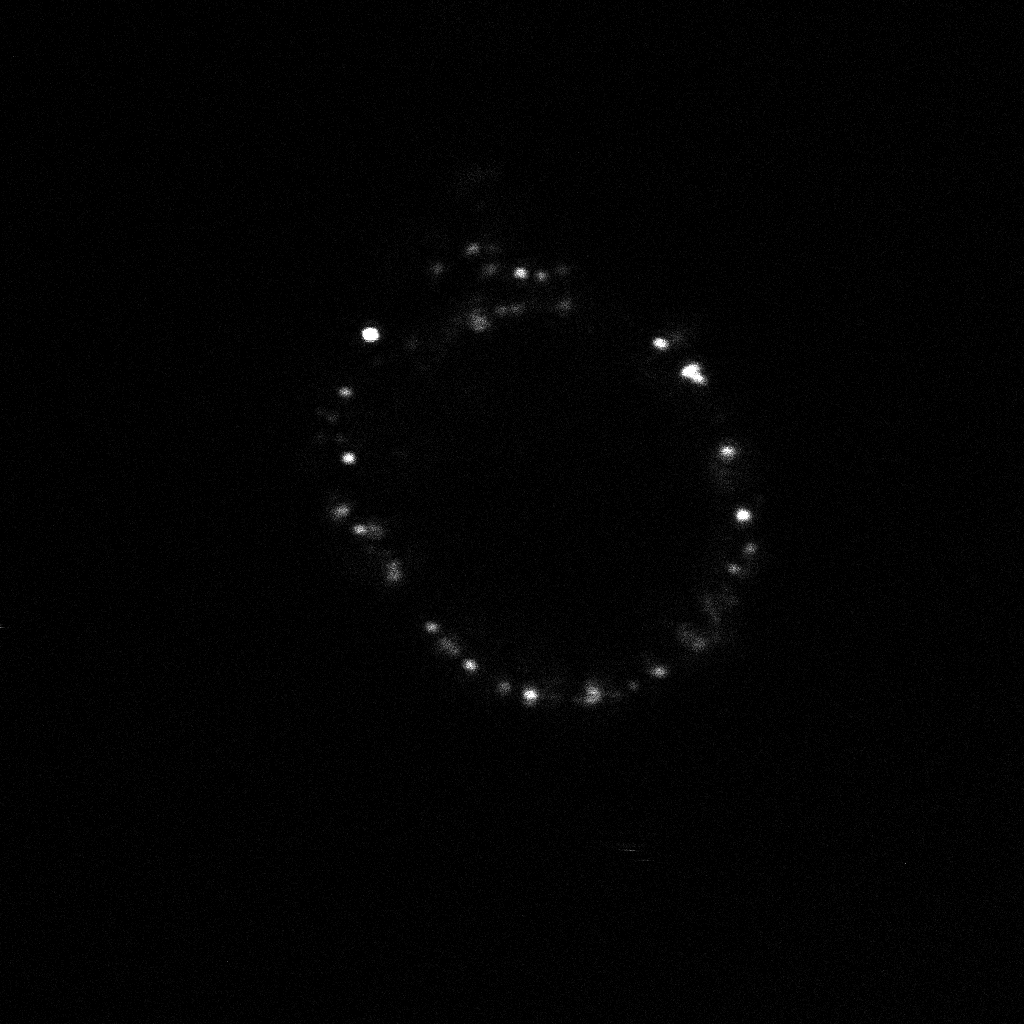

Supplement: Supplementary file 8 — Source data Fig. 1 [file 44319_2025_567_MOESM8_ESM.zip › Fig1/1D/Counted_nuclei/Mechanical/Nuc_18/Nuc_18_z15_RAW_ch00.tif]

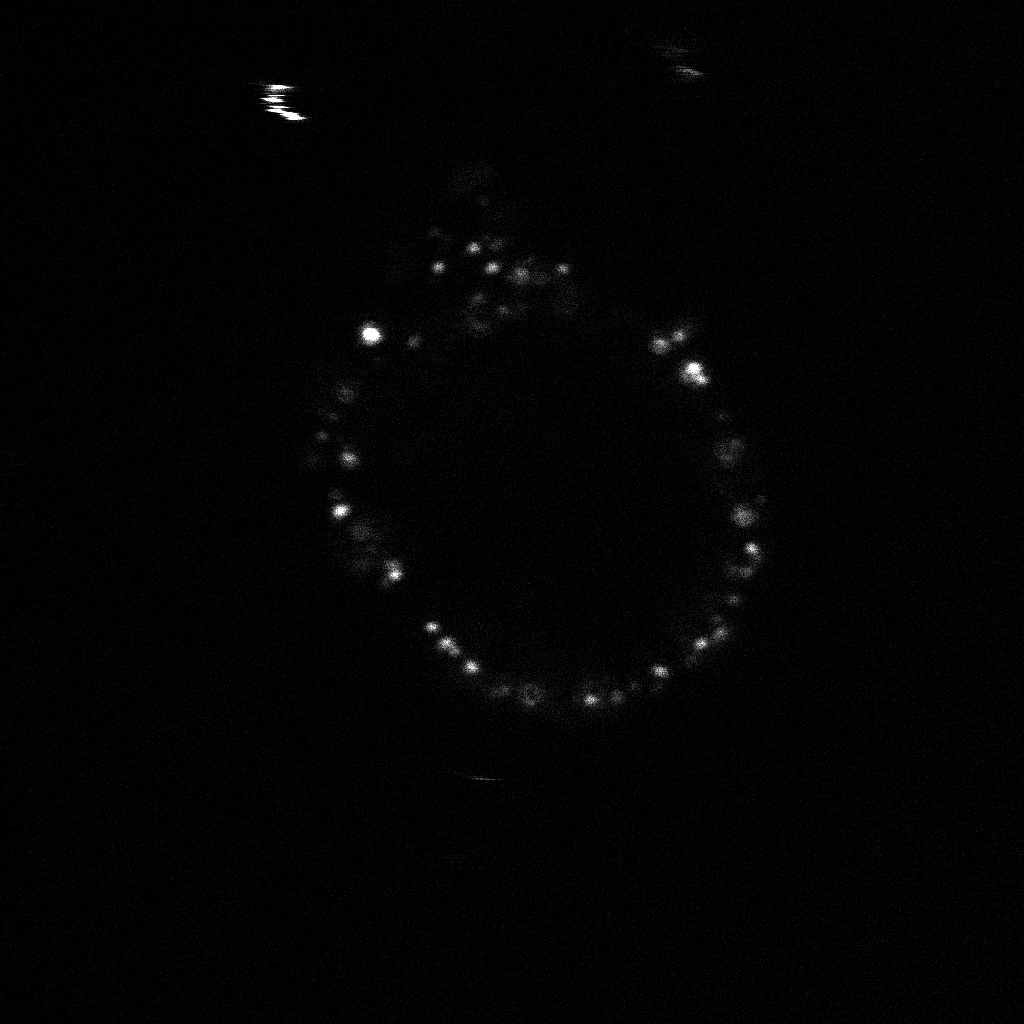

Supplement: Supplementary file 8 — Source data Fig. 1 [file 44319_2025_567_MOESM8_ESM.zip › Fig1/1D/Counted_nuclei/Mechanical/Nuc_18/Nuc_18_z16_RAW_ch00.tif]

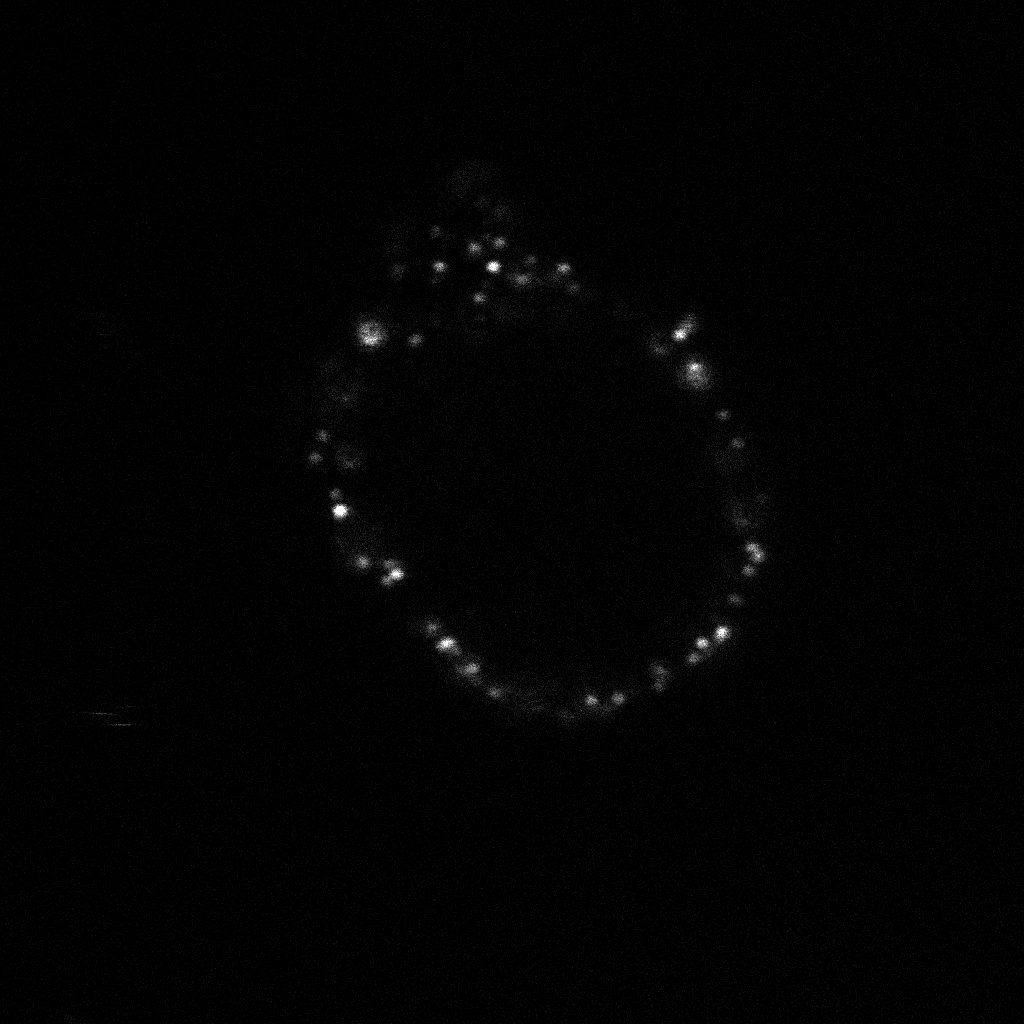

Supplement: Supplementary file 8 — Source data Fig. 1 [file 44319_2025_567_MOESM8_ESM.zip › Fig1/1D/Counted_nuclei/Mechanical/Nuc_18/Nuc_18_z17_RAW_ch00.tif]

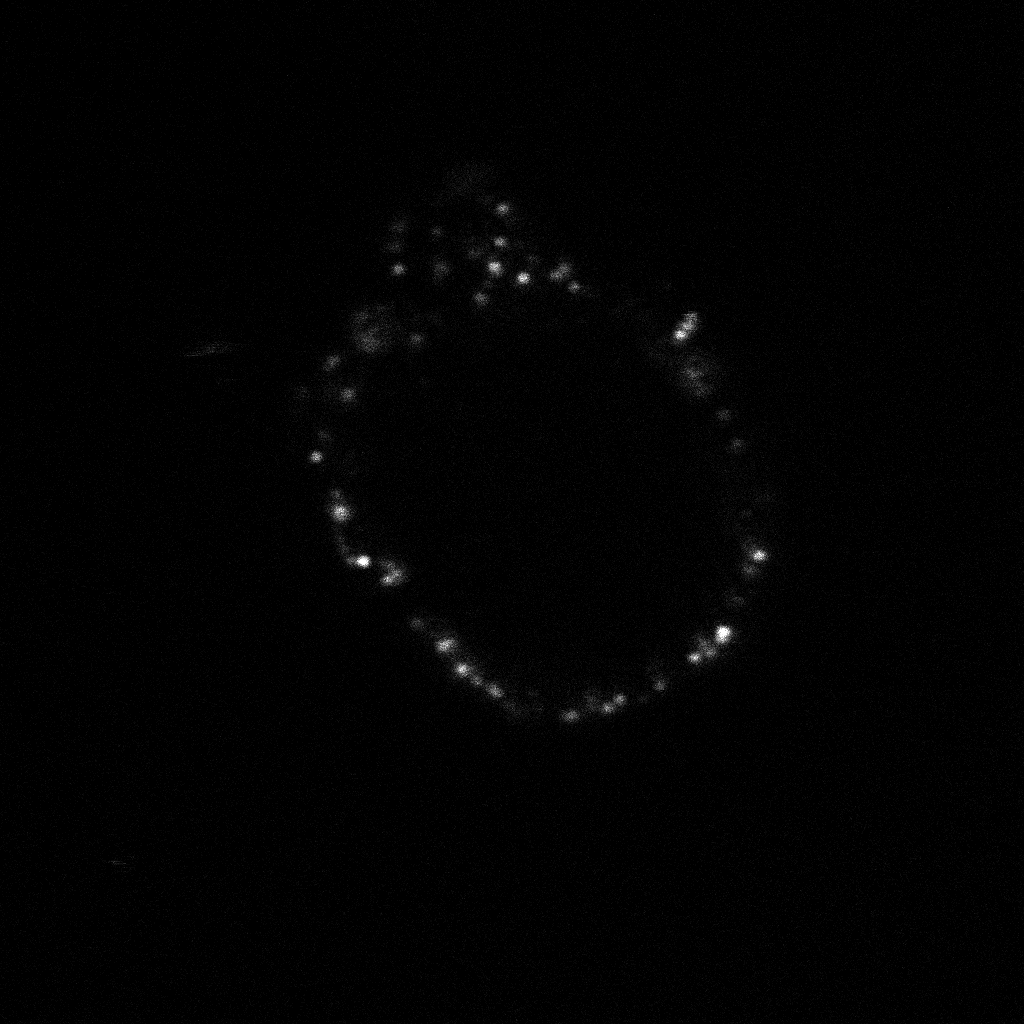

Supplement: Supplementary file 8 — Source data Fig. 1 [file 44319_2025_567_MOESM8_ESM.zip › Fig1/1D/Counted_nuclei/Mechanical/Nuc_18/Nuc_18_z18_RAW_ch00.tif]

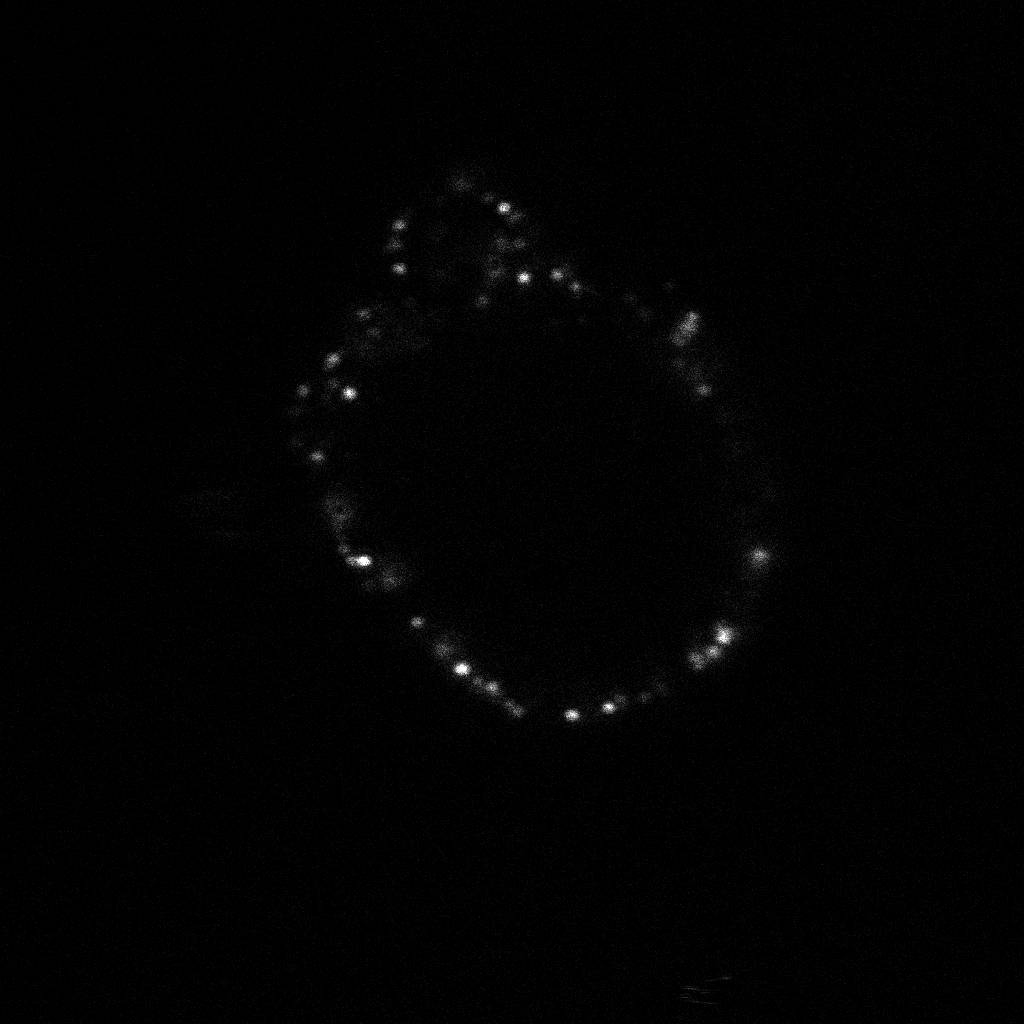

Supplement: Supplementary file 8 — Source data Fig. 1 [file 44319_2025_567_MOESM8_ESM.zip › Fig1/1D/Counted_nuclei/Mechanical/Nuc_18/Nuc_18_z19_RAW_ch00.tif]

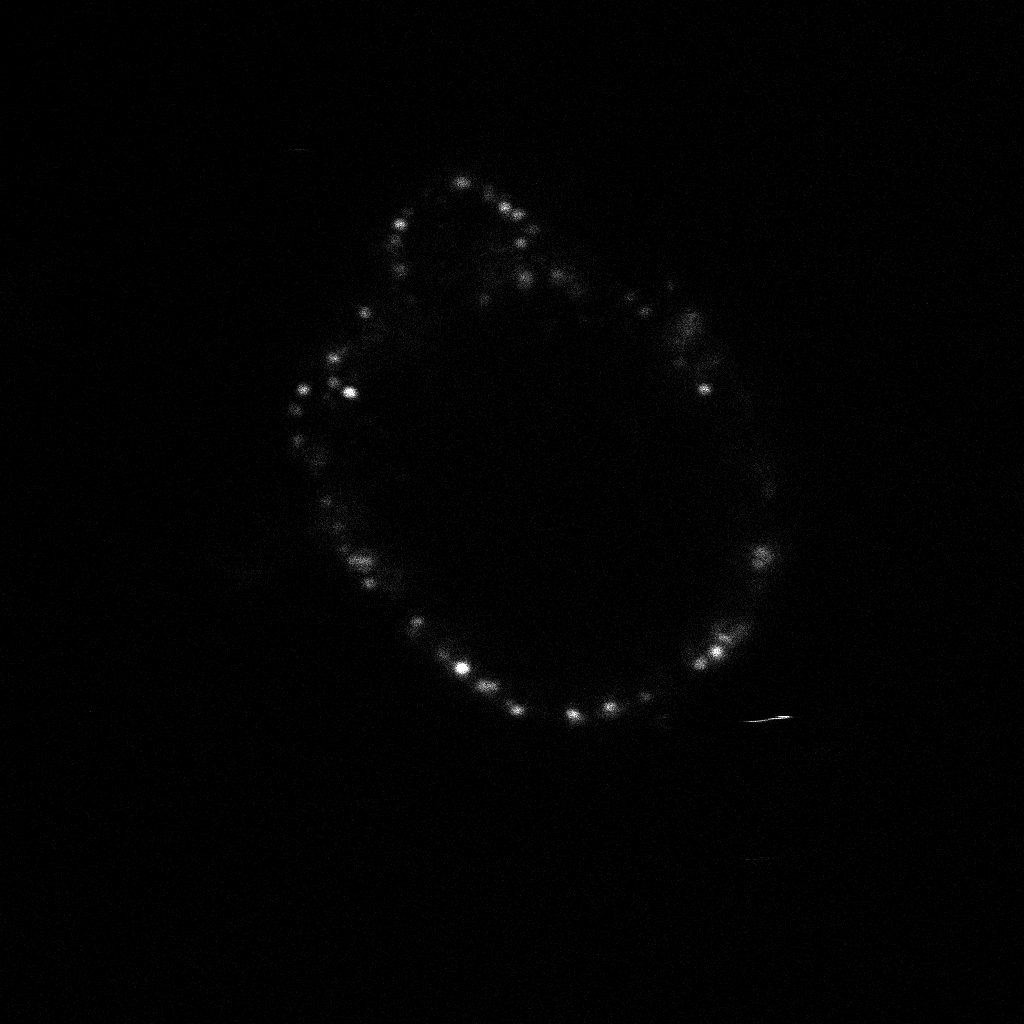

Supplement: Supplementary file 8 — Source data Fig. 1 [file 44319_2025_567_MOESM8_ESM.zip › Fig1/1D/Counted_nuclei/Mechanical/Nuc_18/Nuc_18_z20_RAW_ch00.tif]

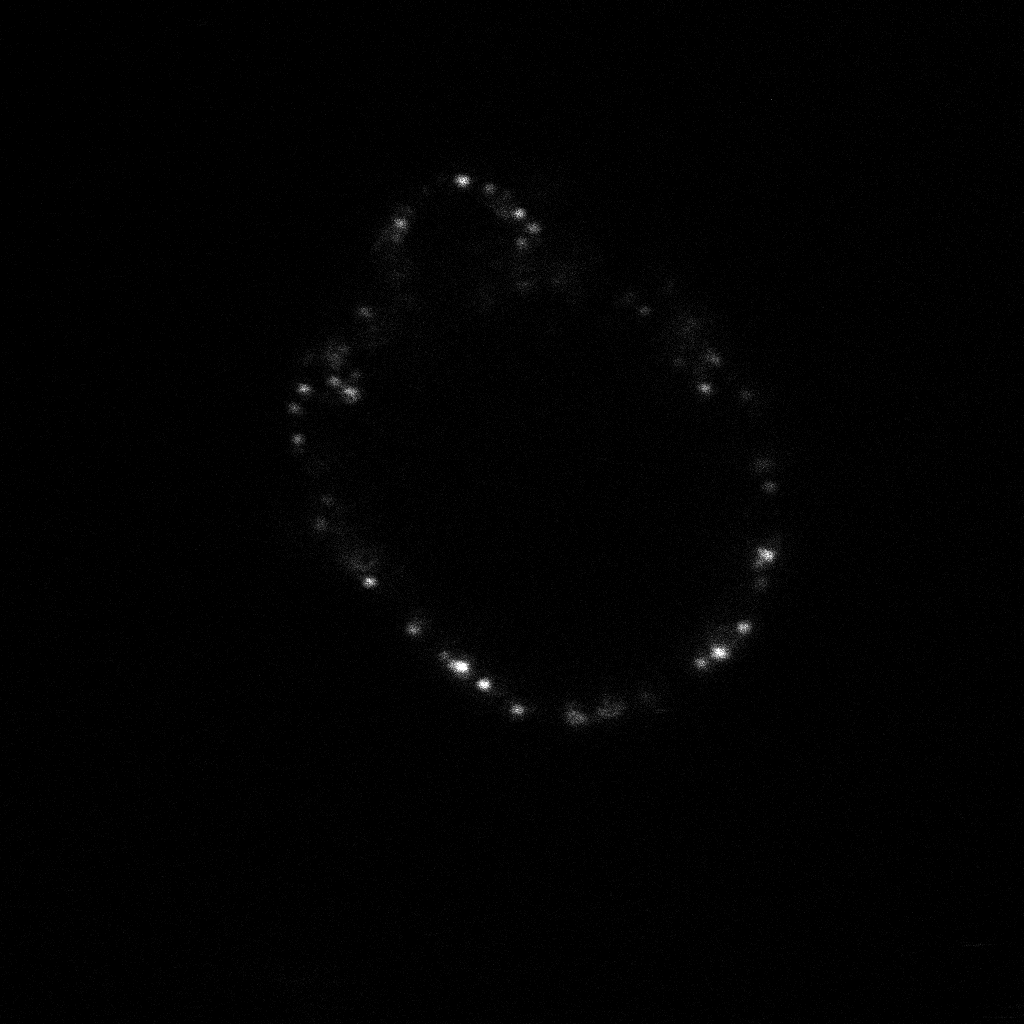

Supplement: Supplementary file 8 — Source data Fig. 1 [file 44319_2025_567_MOESM8_ESM.zip › Fig1/1D/Counted_nuclei/Mechanical/Nuc_18/Nuc_18_z21_RAW_ch00.tif]

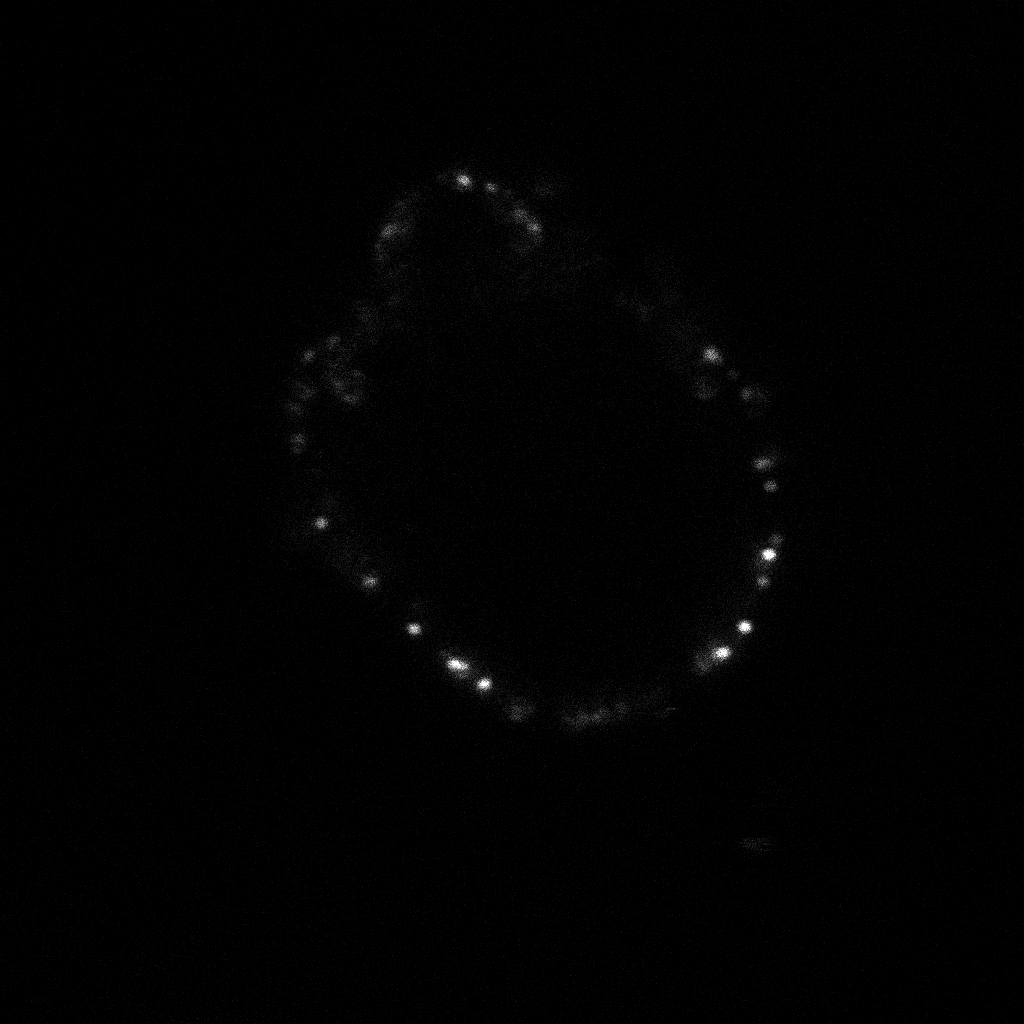

Supplement: Supplementary file 8 — Source data Fig. 1 [file 44319_2025_567_MOESM8_ESM.zip › Fig1/1D/Counted_nuclei/Mechanical/Nuc_18/Nuc_18_z22_RAW_ch00.tif]

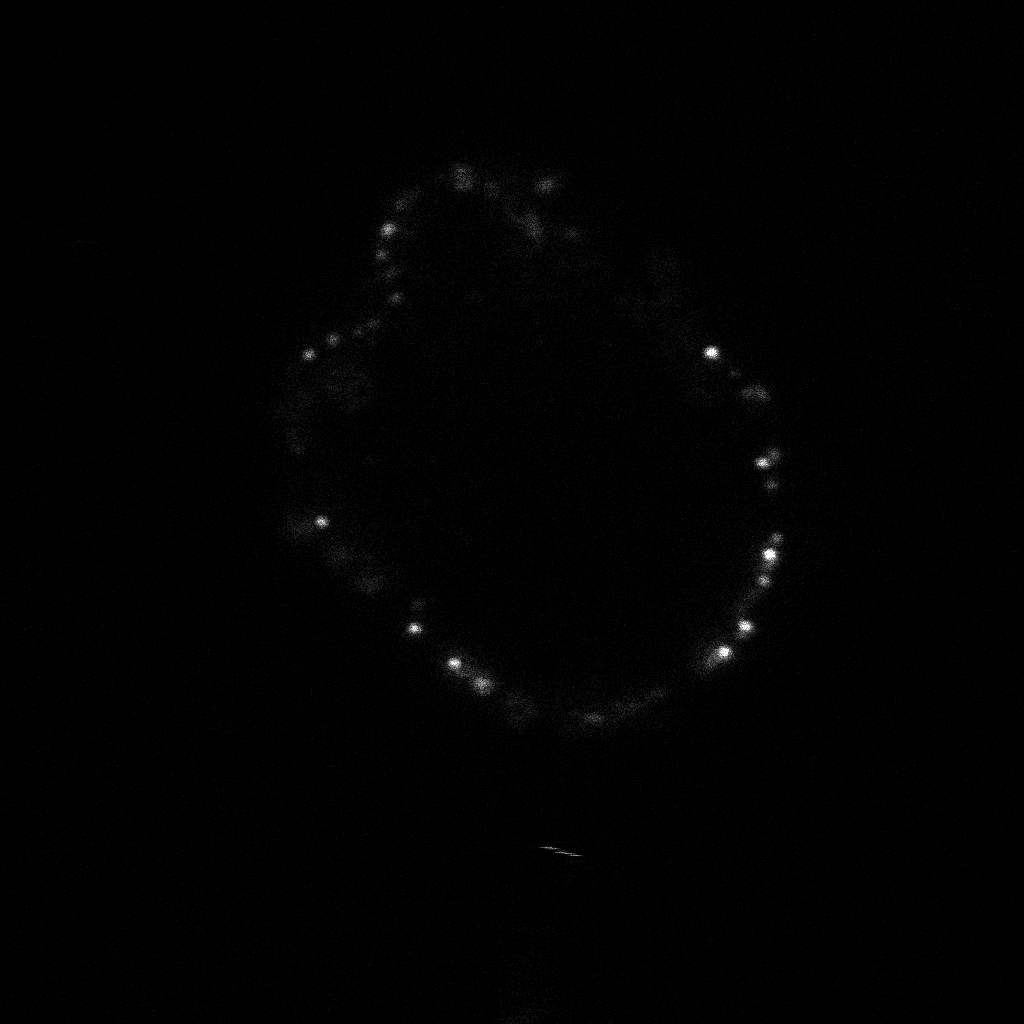

Supplement: Supplementary file 8 — Source data Fig. 1 [file 44319_2025_567_MOESM8_ESM.zip › Fig1/1D/Counted_nuclei/Mechanical/Nuc_18/Nuc_18_z23_RAW_ch00.tif]

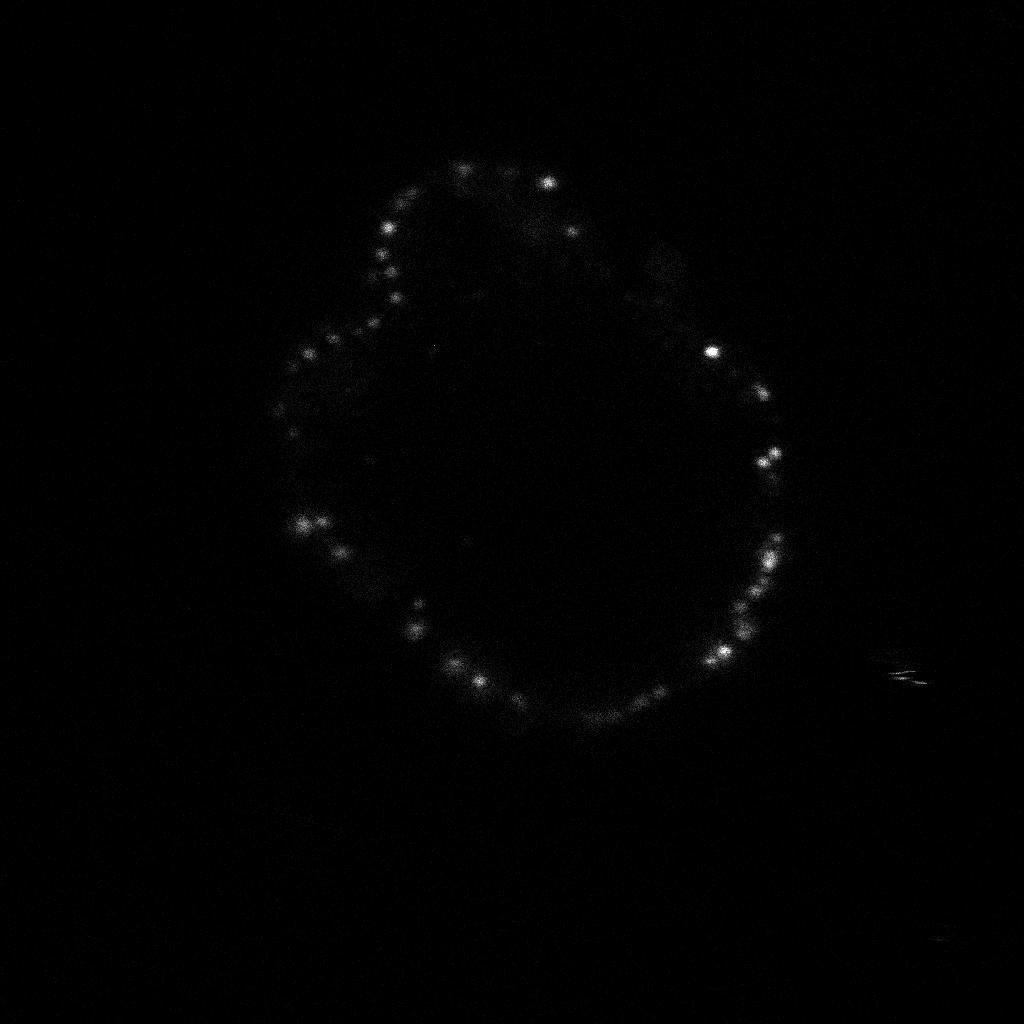

Supplement: Supplementary file 8 — Source data Fig. 1 [file 44319_2025_567_MOESM8_ESM.zip › Fig1/1D/Counted_nuclei/Mechanical/Nuc_18/Nuc_18_z24_RAW_ch00.tif]

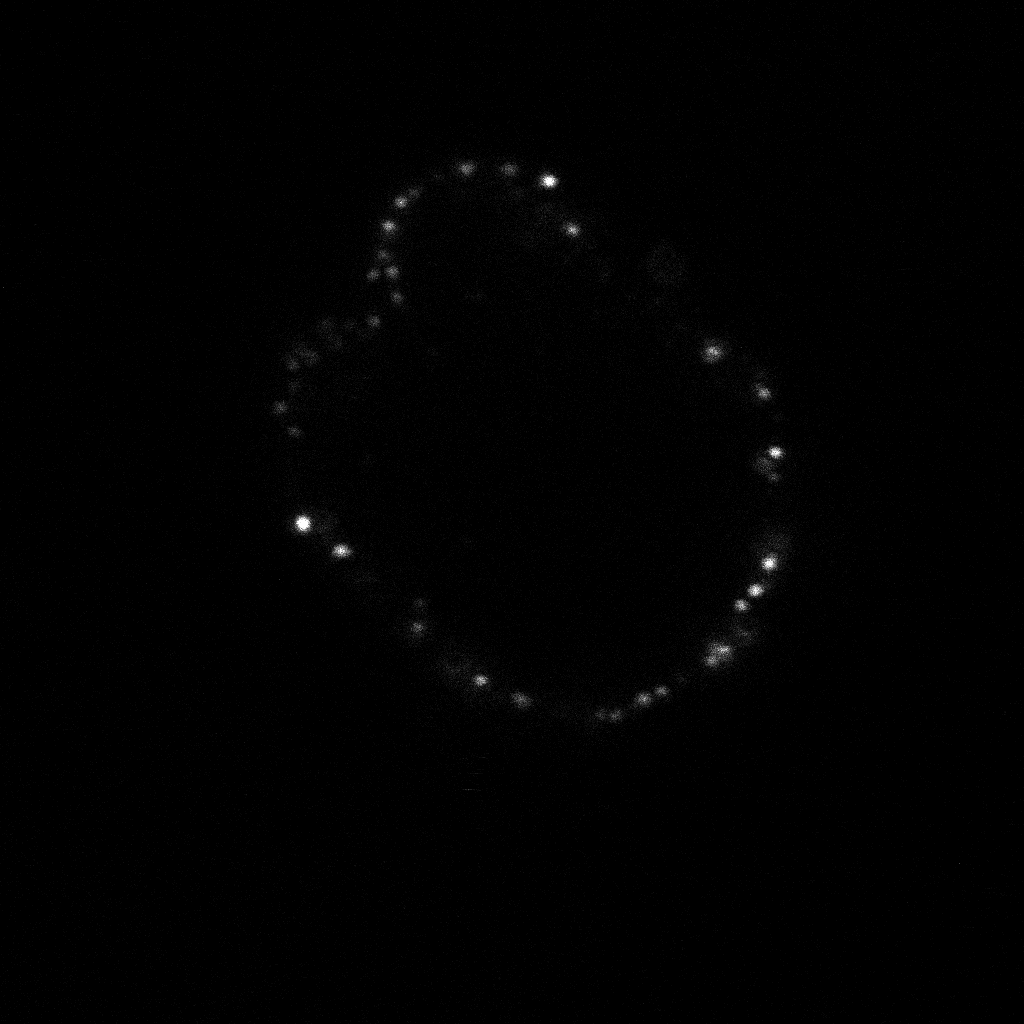

Supplement: Supplementary file 8 — Source data Fig. 1 [file 44319_2025_567_MOESM8_ESM.zip › Fig1/1D/Counted_nuclei/Mechanical/Nuc_18/Nuc_18_z25_RAW_ch00.tif]

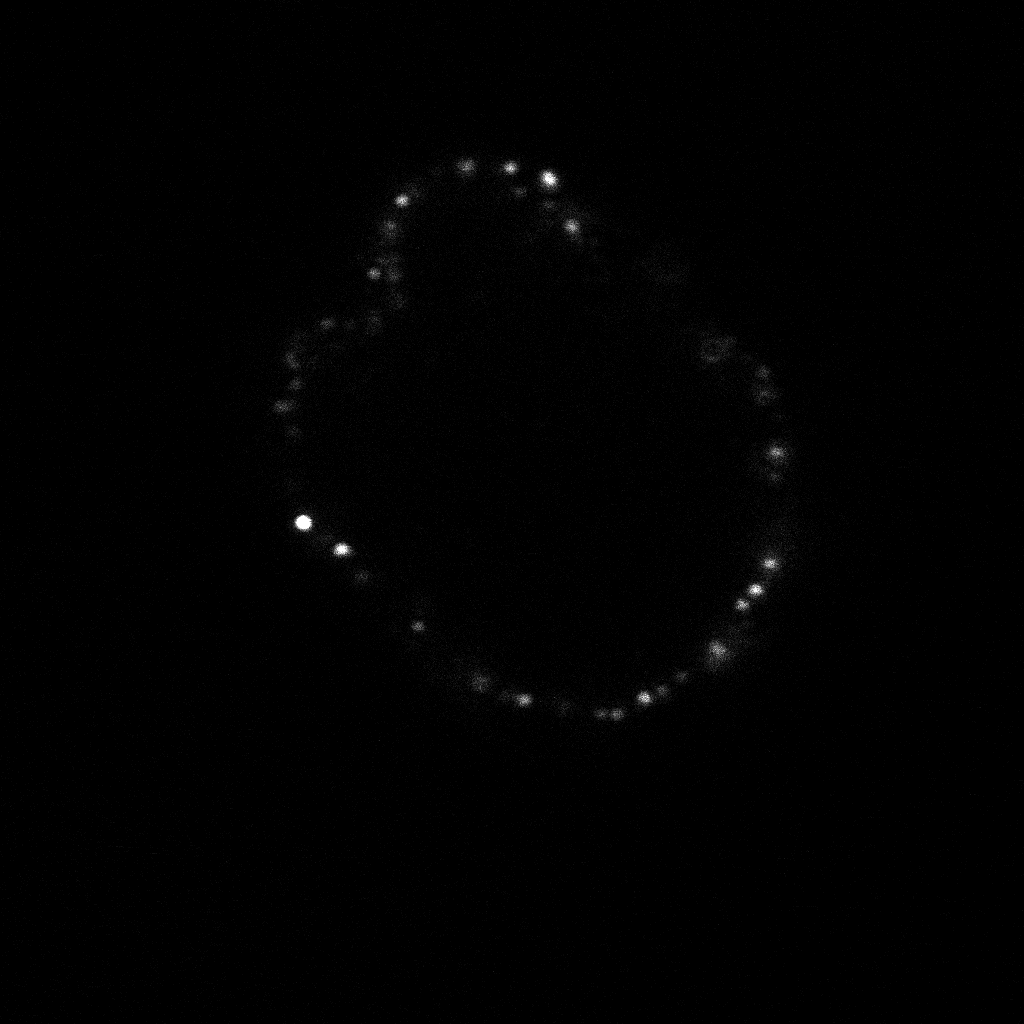

Supplement: Supplementary file 8 — Source data Fig. 1 [file 44319_2025_567_MOESM8_ESM.zip › Fig1/1D/Counted_nuclei/Mechanical/Nuc_18/Nuc_18_z26_RAW_ch00.tif]

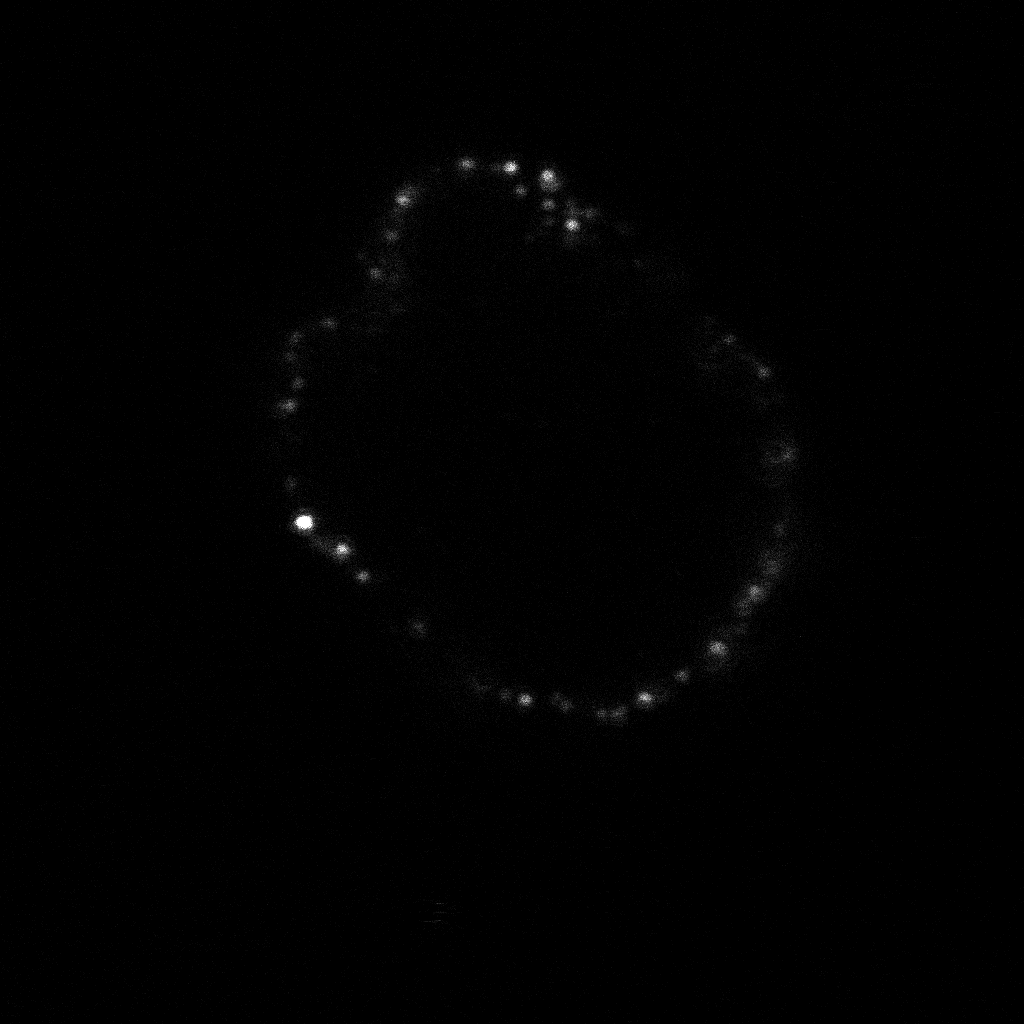

Supplement: Supplementary file 8 — Source data Fig. 1 [file 44319_2025_567_MOESM8_ESM.zip › Fig1/1D/Counted_nuclei/Mechanical/Nuc_18/Nuc_18_z27_RAW_ch00.tif]

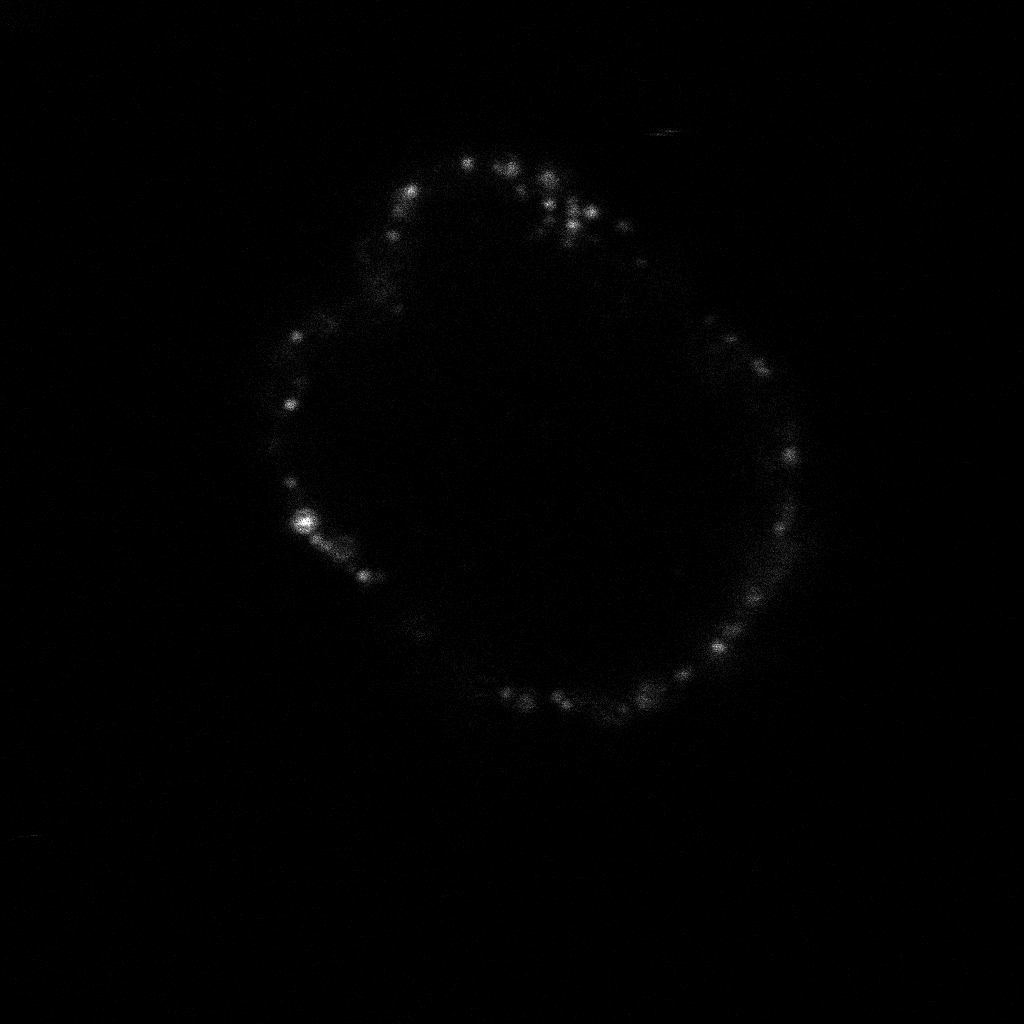

Supplement: Supplementary file 8 — Source data Fig. 1 [file 44319_2025_567_MOESM8_ESM.zip › Fig1/1D/Counted_nuclei/Mechanical/Nuc_18/Nuc_18_z28_RAW_ch00.tif]

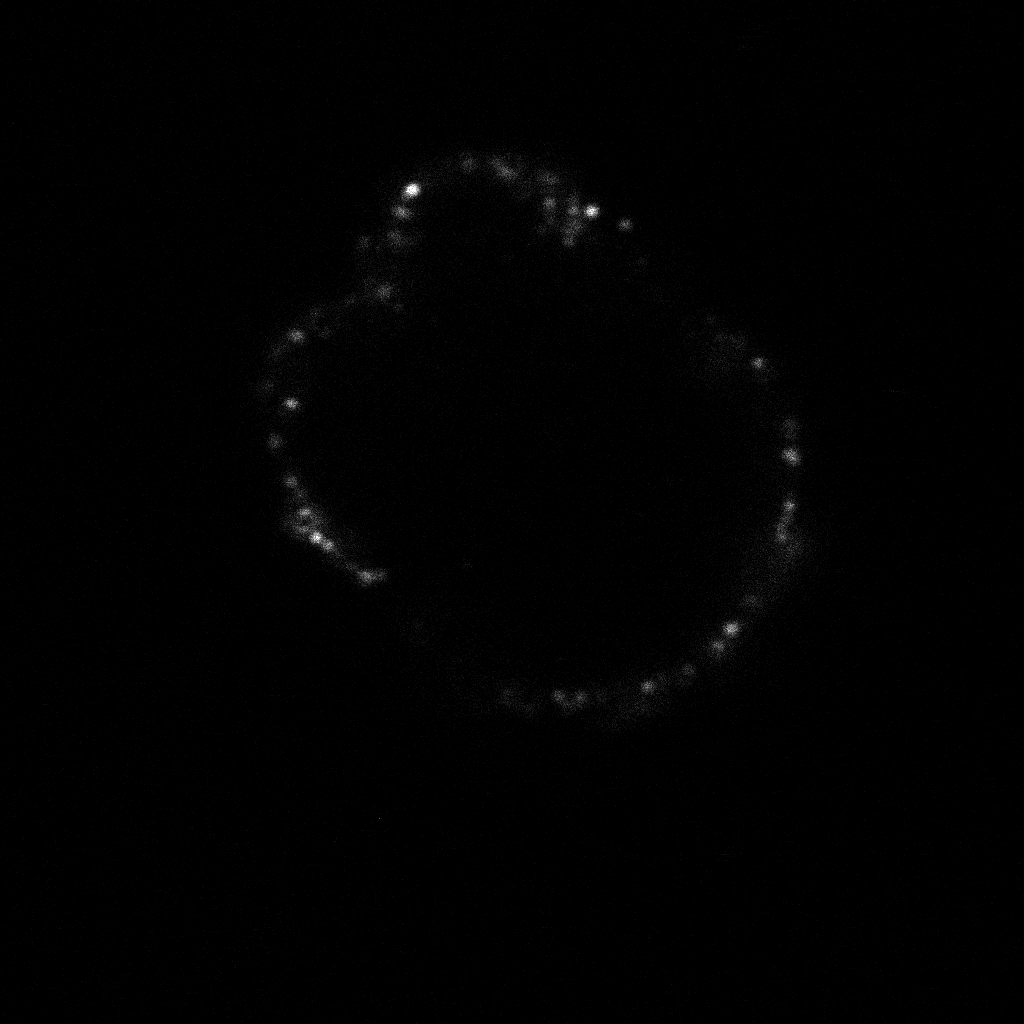

Supplement: Supplementary file 8 — Source data Fig. 1 [file 44319_2025_567_MOESM8_ESM.zip › Fig1/1D/Counted_nuclei/Mechanical/Nuc_18/Nuc_18_z29_RAW_ch00.tif]

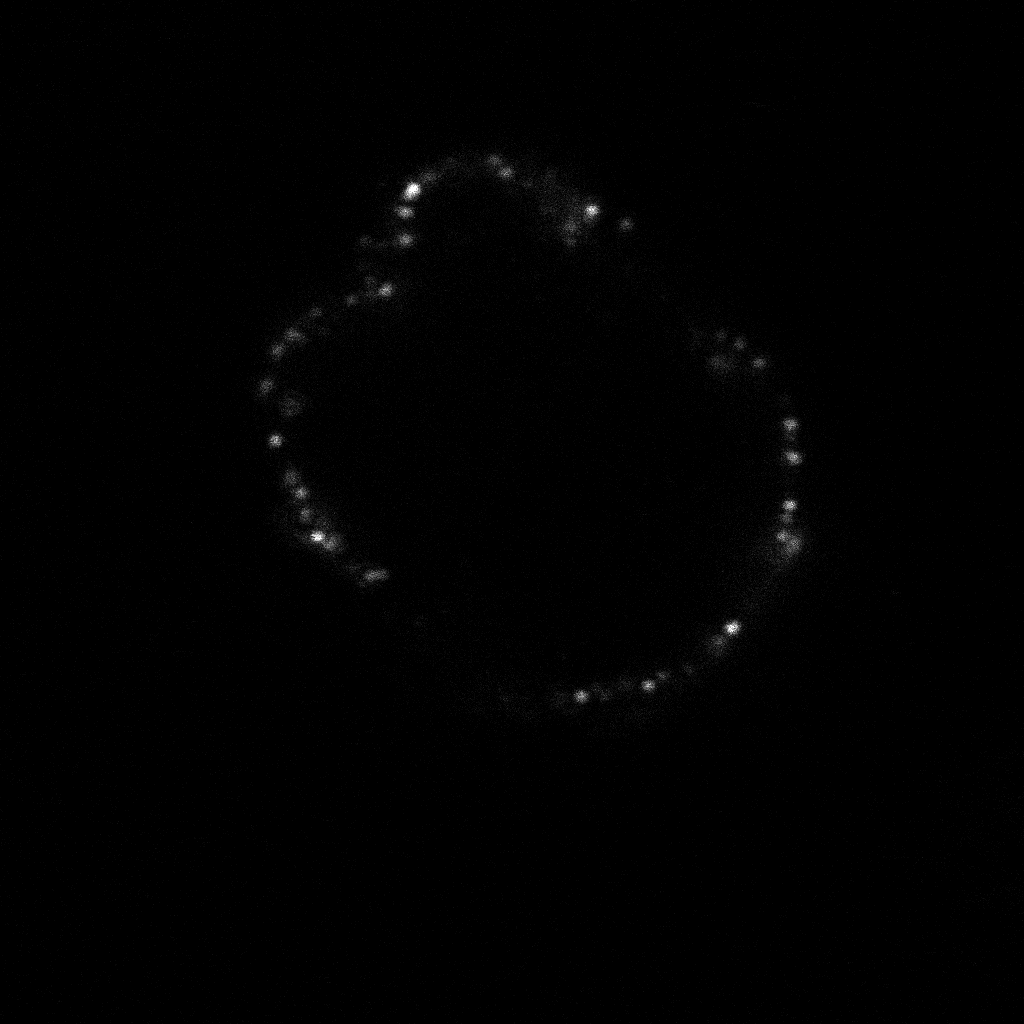

Supplement: Supplementary file 8 — Source data Fig. 1 [file 44319_2025_567_MOESM8_ESM.zip › Fig1/1D/Counted_nuclei/Mechanical/Nuc_18/Nuc_18_z30_RAW_ch00.tif]

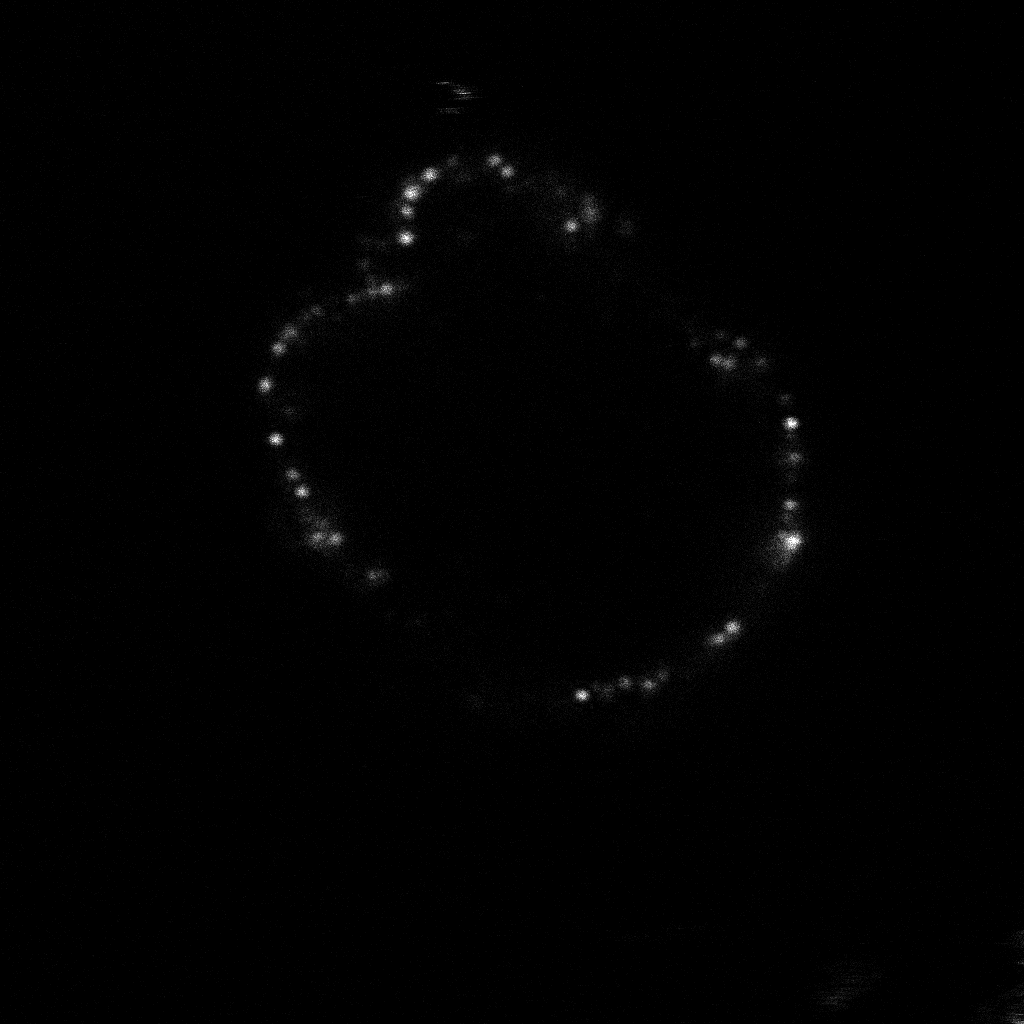

Supplement: Supplementary file 8 — Source data Fig. 1 [file 44319_2025_567_MOESM8_ESM.zip › Fig1/1D/Counted_nuclei/Mechanical/Nuc_18/Nuc_18_z31_RAW_ch00.tif]

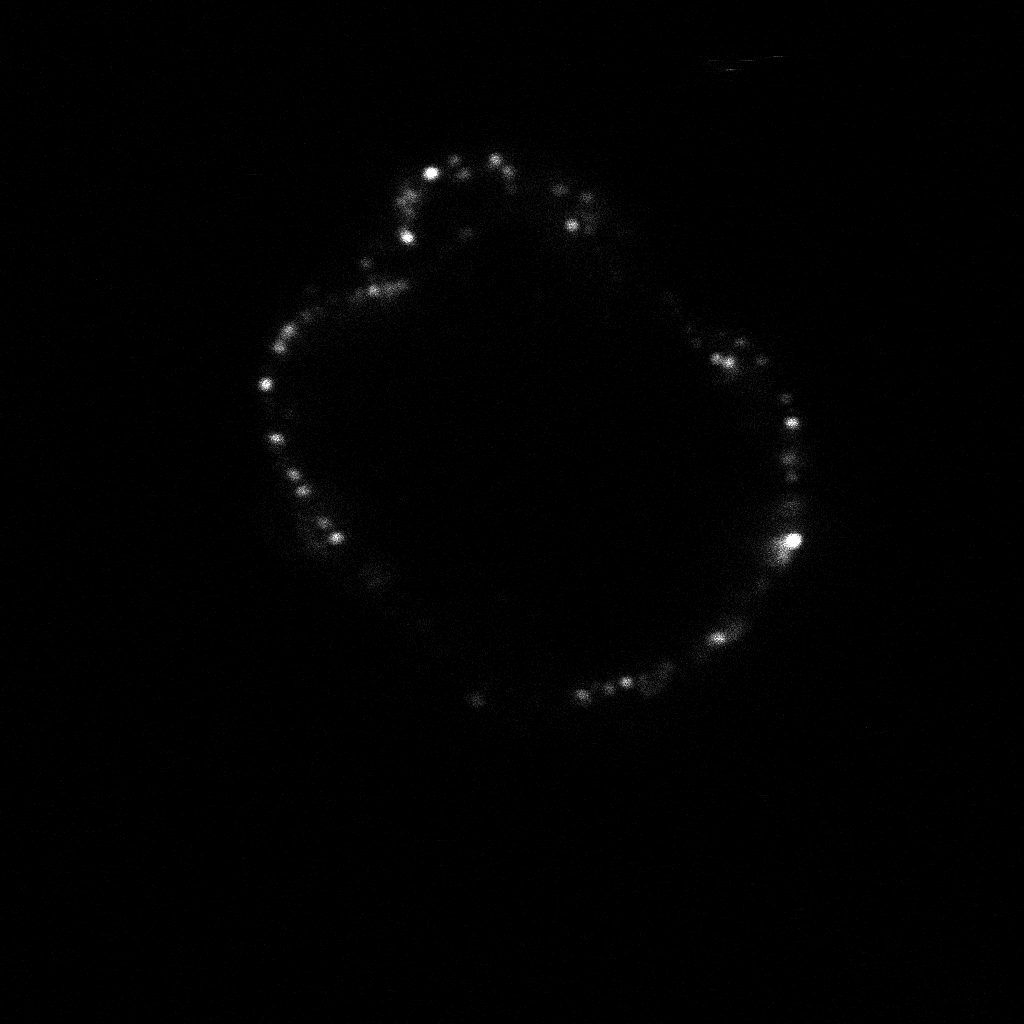

Supplement: Supplementary file 8 — Source data Fig. 1 [file 44319_2025_567_MOESM8_ESM.zip › Fig1/1D/Counted_nuclei/Mechanical/Nuc_18/Nuc_18_z32_RAW_ch00.tif]

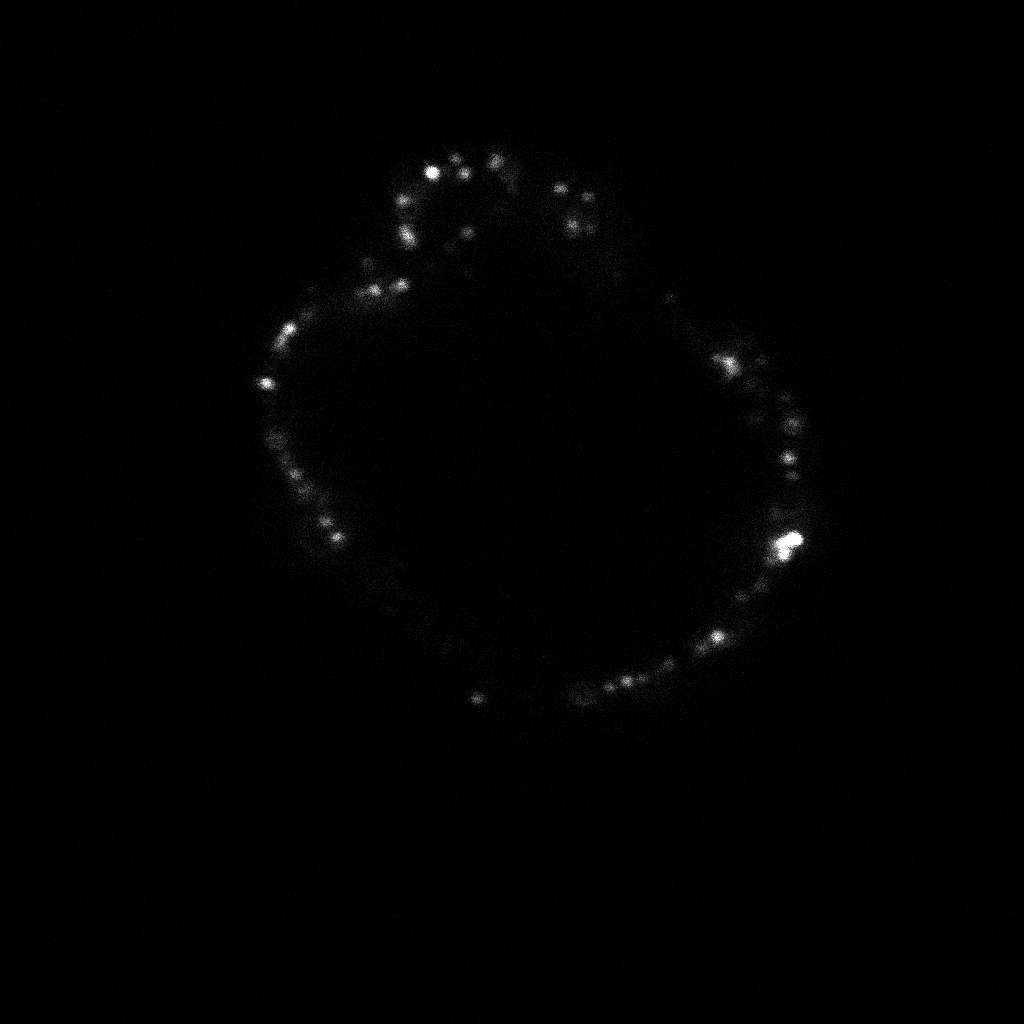

Supplement: Supplementary file 8 — Source data Fig. 1 [file 44319_2025_567_MOESM8_ESM.zip › Fig1/1D/Counted_nuclei/Mechanical/Nuc_18/Nuc_18_z33_RAW_ch00.tif]

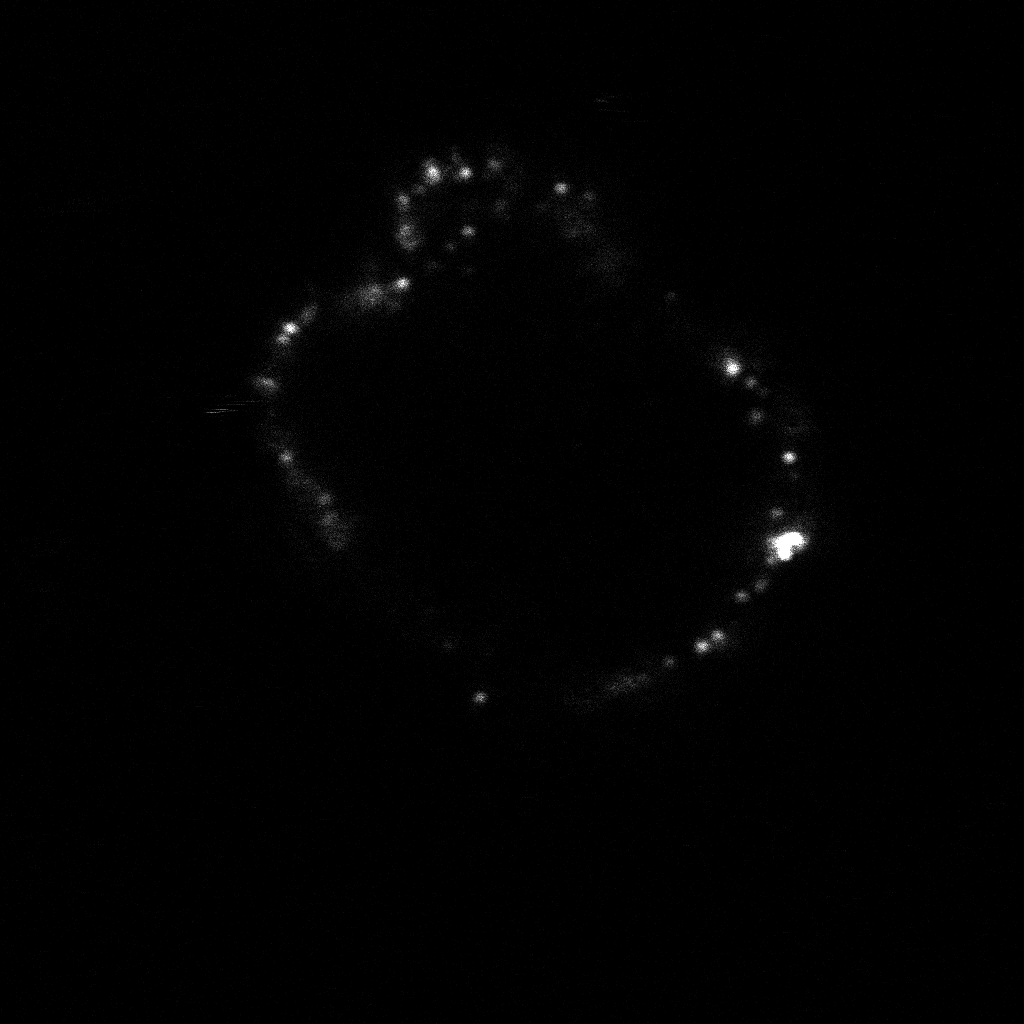

Supplement: Supplementary file 8 — Source data Fig. 1 [file 44319_2025_567_MOESM8_ESM.zip › Fig1/1D/Counted_nuclei/Mechanical/Nuc_18/Nuc_18_z34_RAW_ch00.tif]

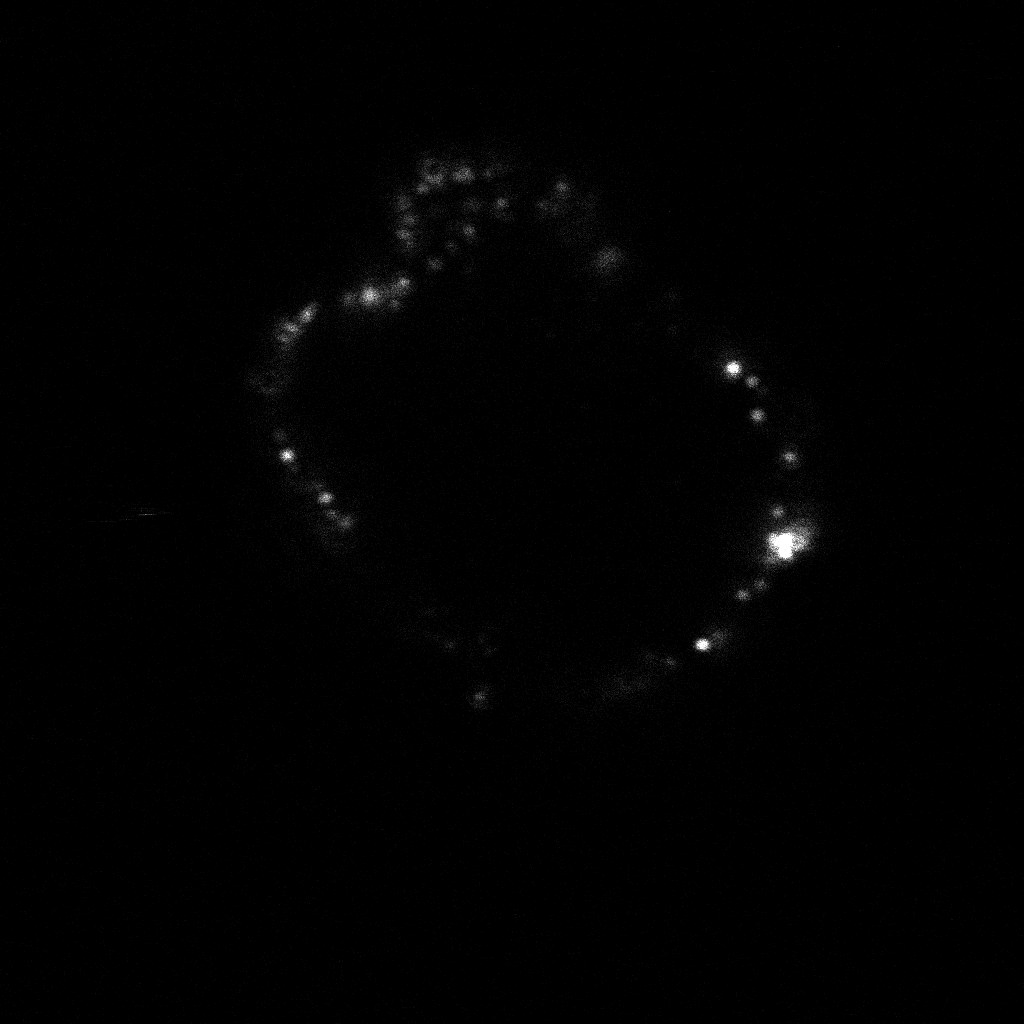

Supplement: Supplementary file 8 — Source data Fig. 1 [file 44319_2025_567_MOESM8_ESM.zip › Fig1/1D/Counted_nuclei/Mechanical/Nuc_18/Nuc_18_z35_RAW_ch00.tif]

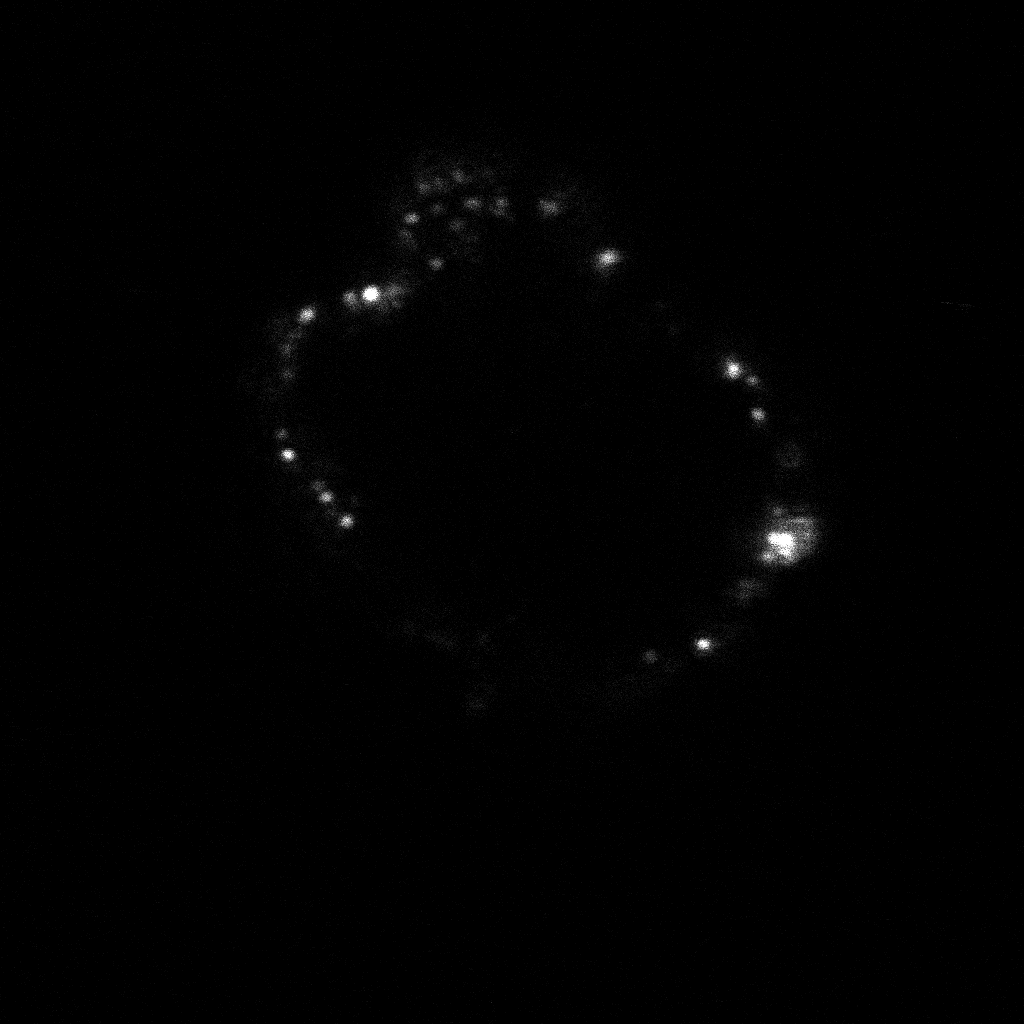

Supplement: Supplementary file 8 — Source data Fig. 1 [file 44319_2025_567_MOESM8_ESM.zip › Fig1/1D/Counted_nuclei/Mechanical/Nuc_18/Nuc_18_z36_RAW_ch00.tif]

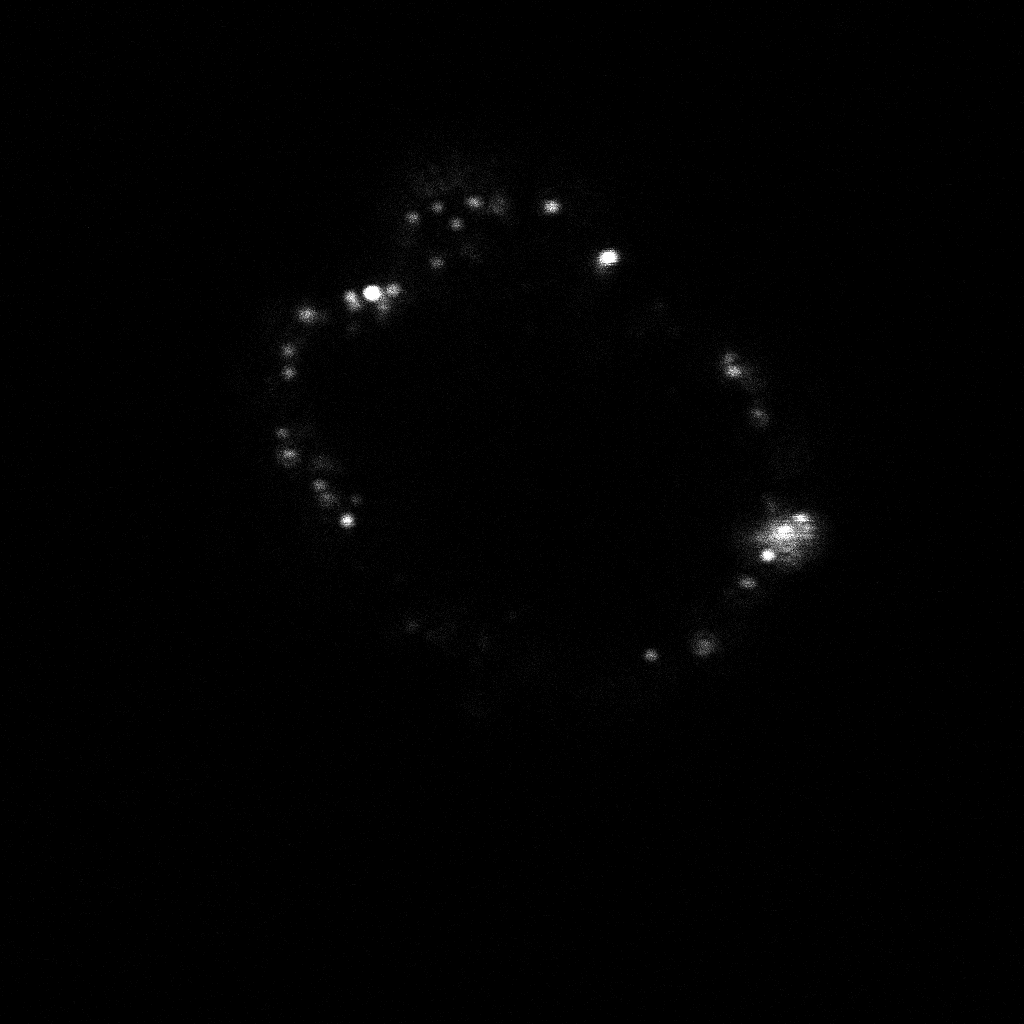

Supplement: Supplementary file 8 — Source data Fig. 1 [file 44319_2025_567_MOESM8_ESM.zip › Fig1/1D/Counted_nuclei/Mechanical/Nuc_18/Nuc_18_z37_RAW_ch00.tif]

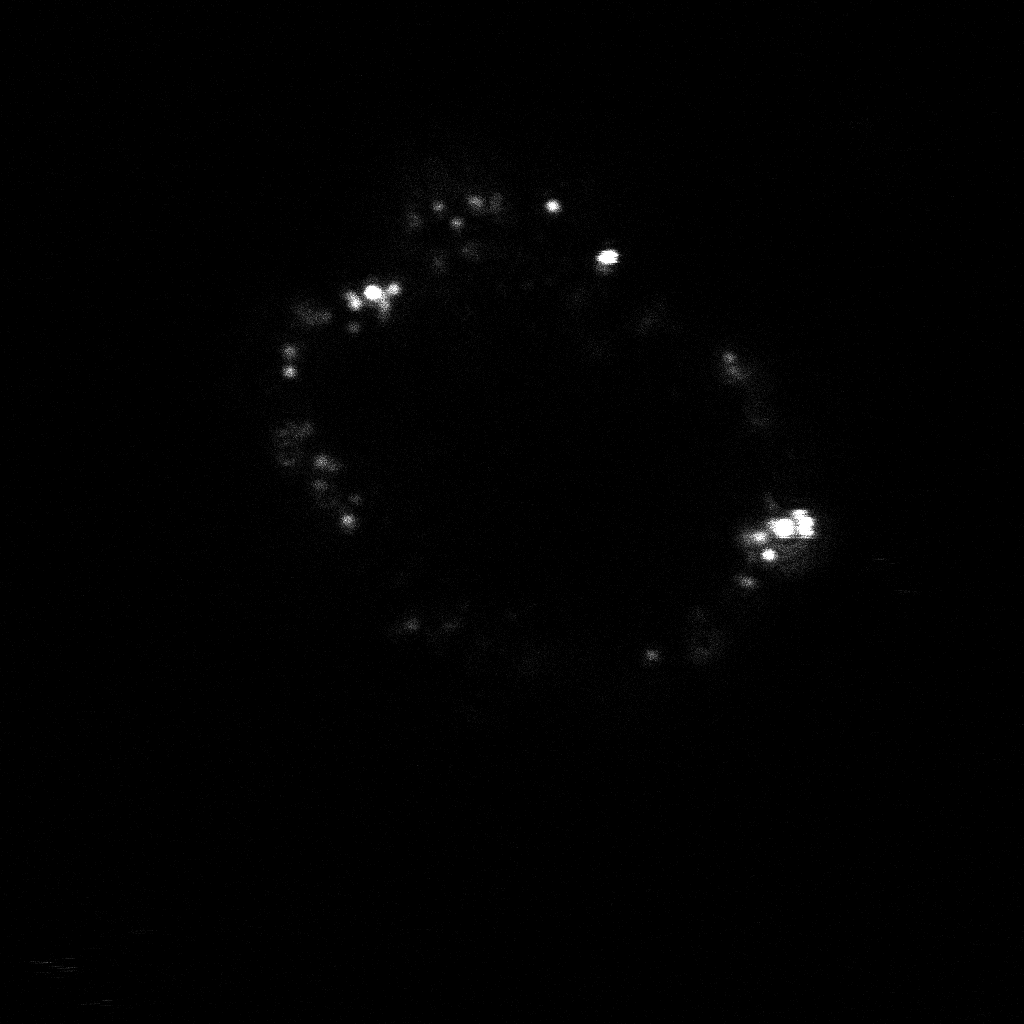

Supplement: Supplementary file 8 — Source data Fig. 1 [file 44319_2025_567_MOESM8_ESM.zip › Fig1/1D/Counted_nuclei/Mechanical/Nuc_18/Nuc_18_z38_RAW_ch00.tif]

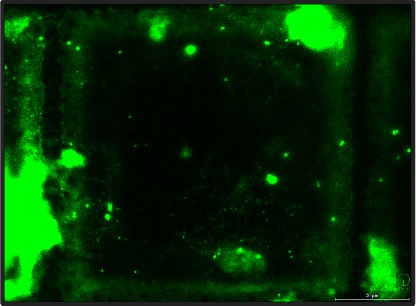

Supplement: Supplementary file 9 — Source data Fig. 2 [file 44319_2025_567_MOESM9_ESM.zip › Fig2/A/i.jpg]

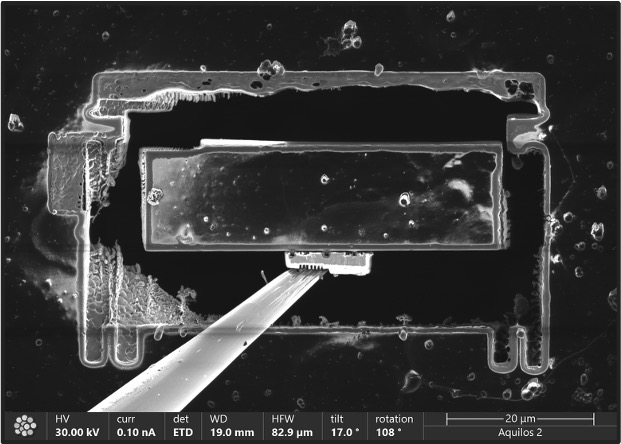

Supplement: Supplementary file 9 — Source data Fig. 2 [file 44319_2025_567_MOESM9_ESM.zip › Fig2/A/ii.jpg]

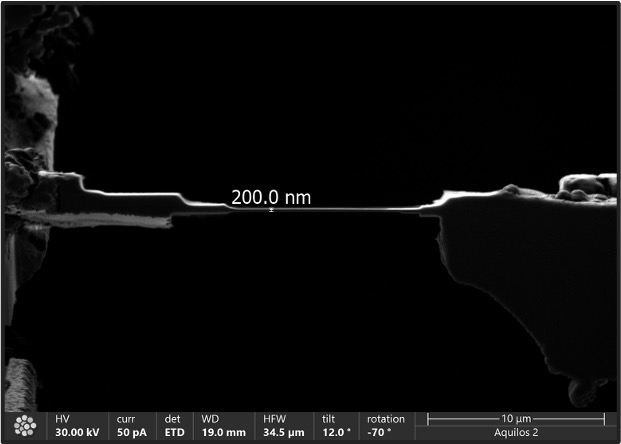

Supplement: Supplementary file 9 — Source data Fig. 2 [file 44319_2025_567_MOESM9_ESM.zip › Fig2/A/iii.jpg]
