# Supplementary figures and images for: Direct visualization of HIV-1 core nuclear import and its interplay with the nuclear pore (part 1 of 3)
Source: EMBO Rep. 2025 Aug 29;26(21):5133–53. doi: 10.1038/s44319-025-00567-6 (PMC12592377; doi:10.1038/s44319-025-00567-6)

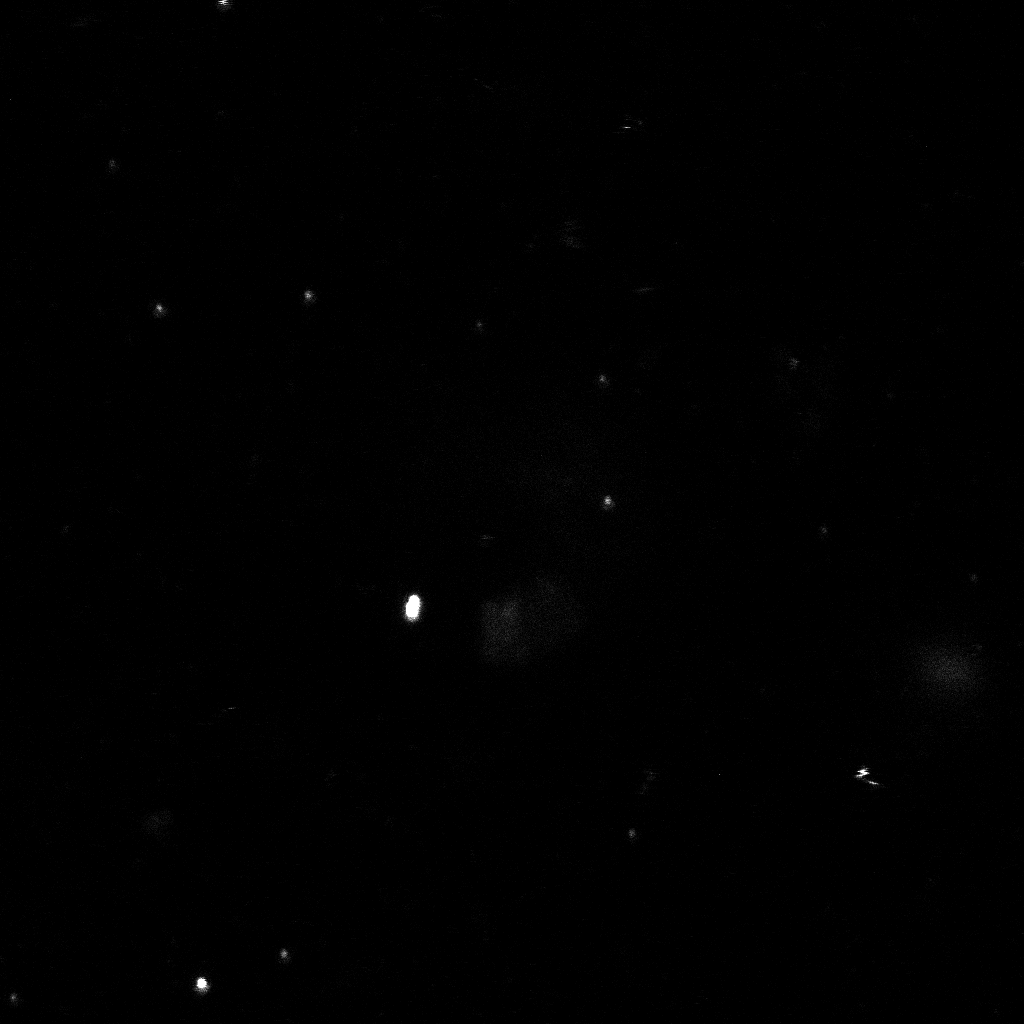

Supplement: Supplementary file 8 — Source data Fig. 1 [file 44319_2025_567_MOESM8_ESM.zip › Fig1/1A/CEM-cores_mechanical_RAW_ch00.tif]

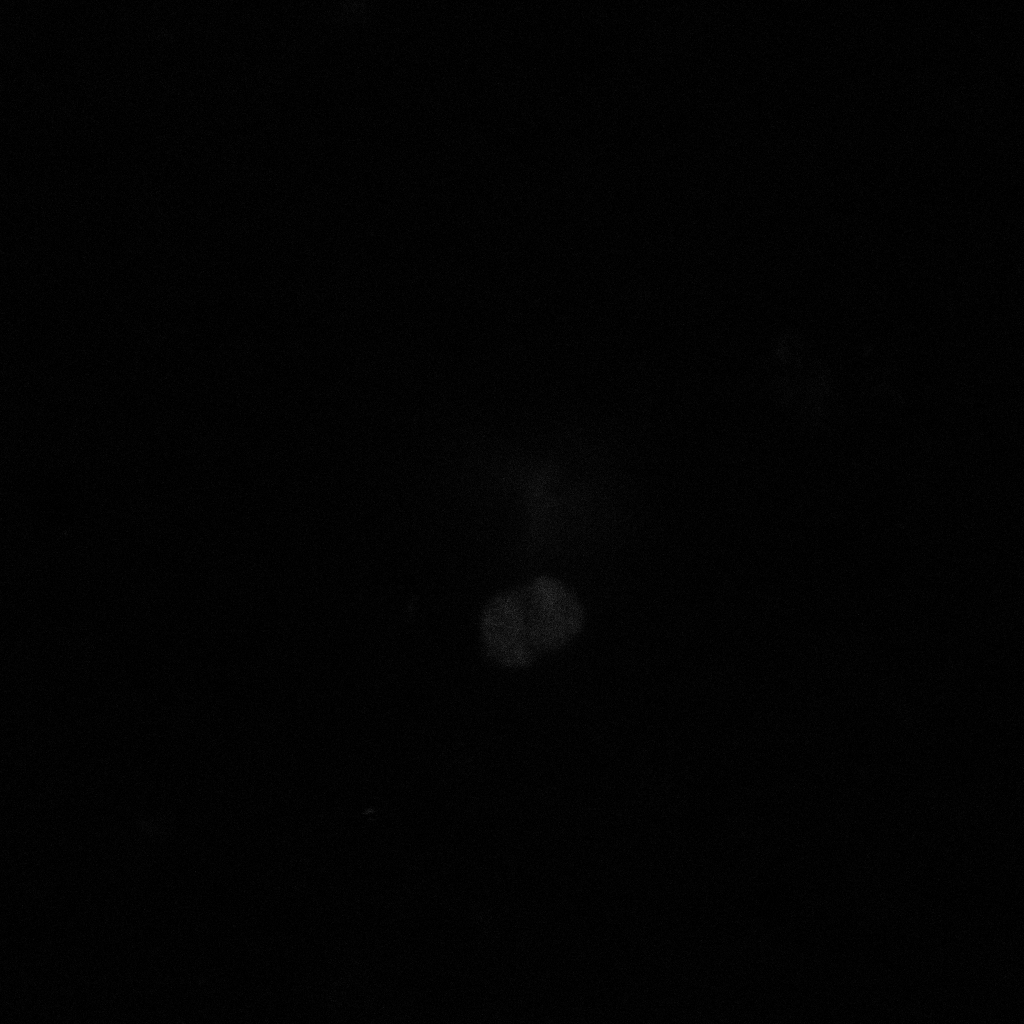

Supplement: Supplementary file 8 — Source data Fig. 1 [file 44319_2025_567_MOESM8_ESM.zip › Fig1/1A/CEM-cores_mechanical_RAW_ch01.tif]

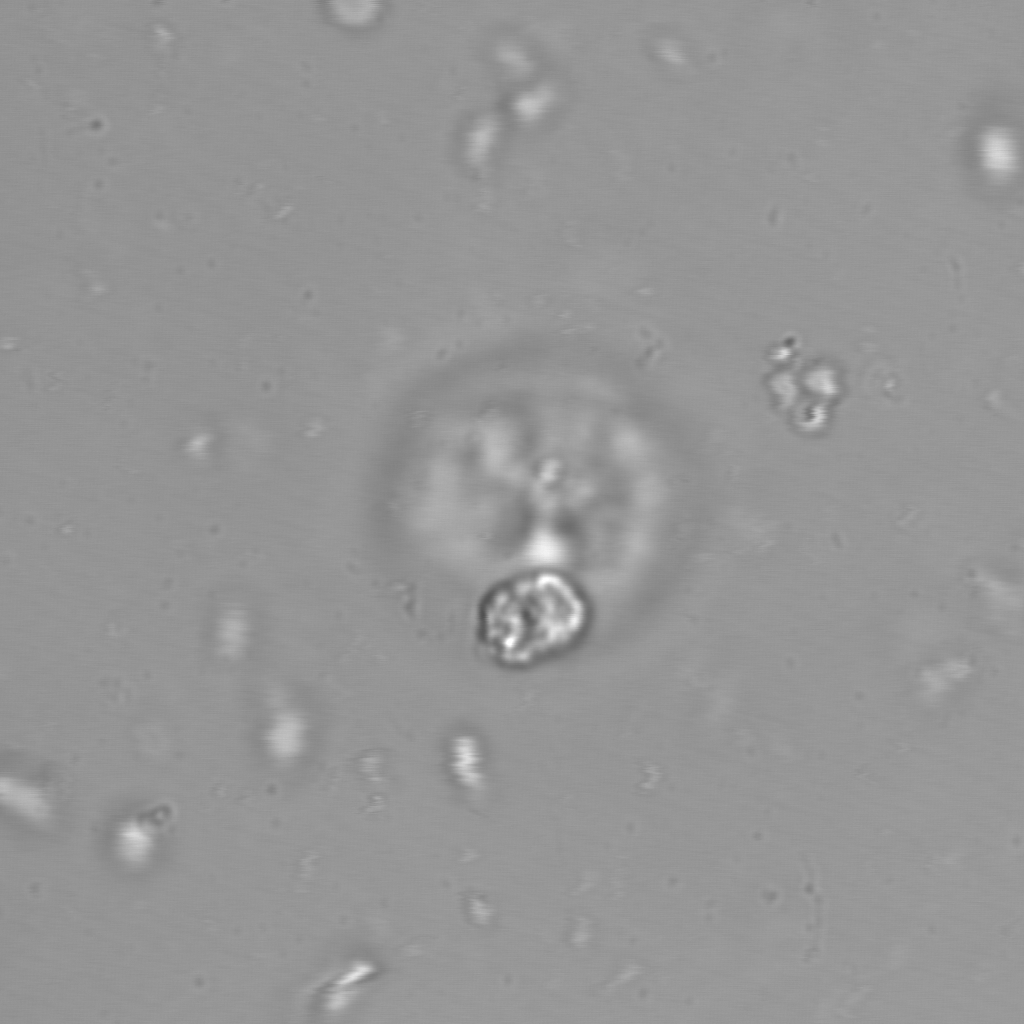

Supplement: Supplementary file 8 — Source data Fig. 1 [file 44319_2025_567_MOESM8_ESM.zip › Fig1/1A/CEM-cores_mechanical_RAW_ch02.tif]

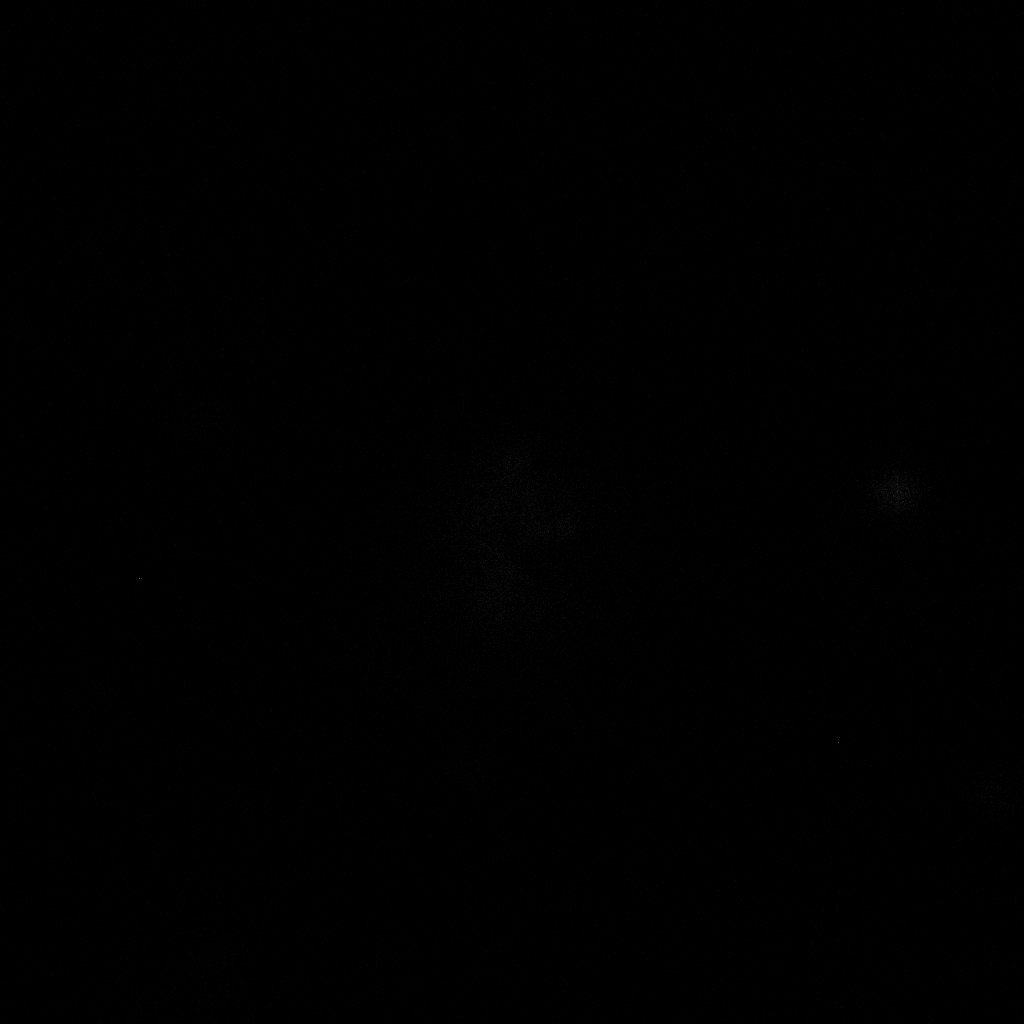

Supplement: Supplementary file 8 — Source data Fig. 1 [file 44319_2025_567_MOESM8_ESM.zip › Fig1/1B/CEM-NUP358-cores_RAW_ch00.tif]

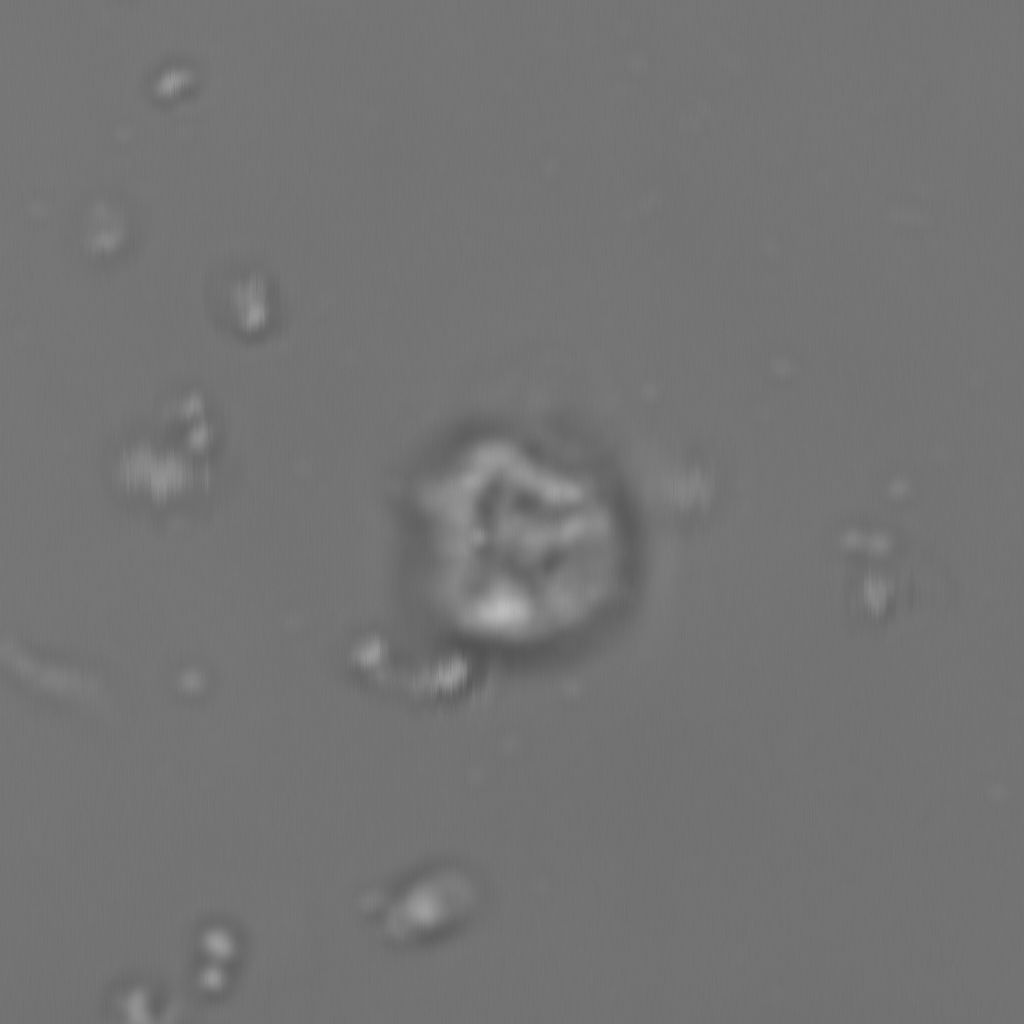

Supplement: Supplementary file 8 — Source data Fig. 1 [file 44319_2025_567_MOESM8_ESM.zip › Fig1/1B/CEM-NUP358-cores_RAW_ch01.tif]

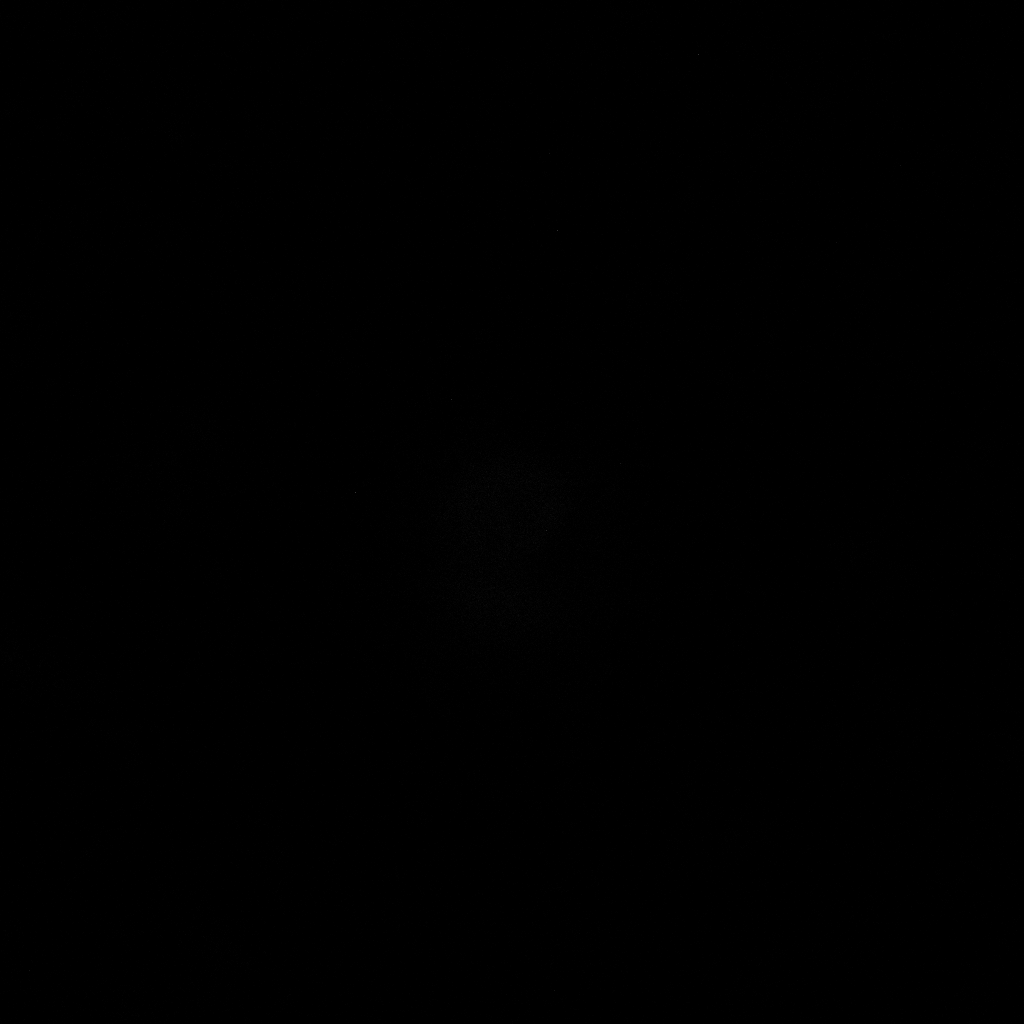

Supplement: Supplementary file 8 — Source data Fig. 1 [file 44319_2025_567_MOESM8_ESM.zip › Fig1/1B/CEM-NUP358-cores_RAW_ch02.tif]

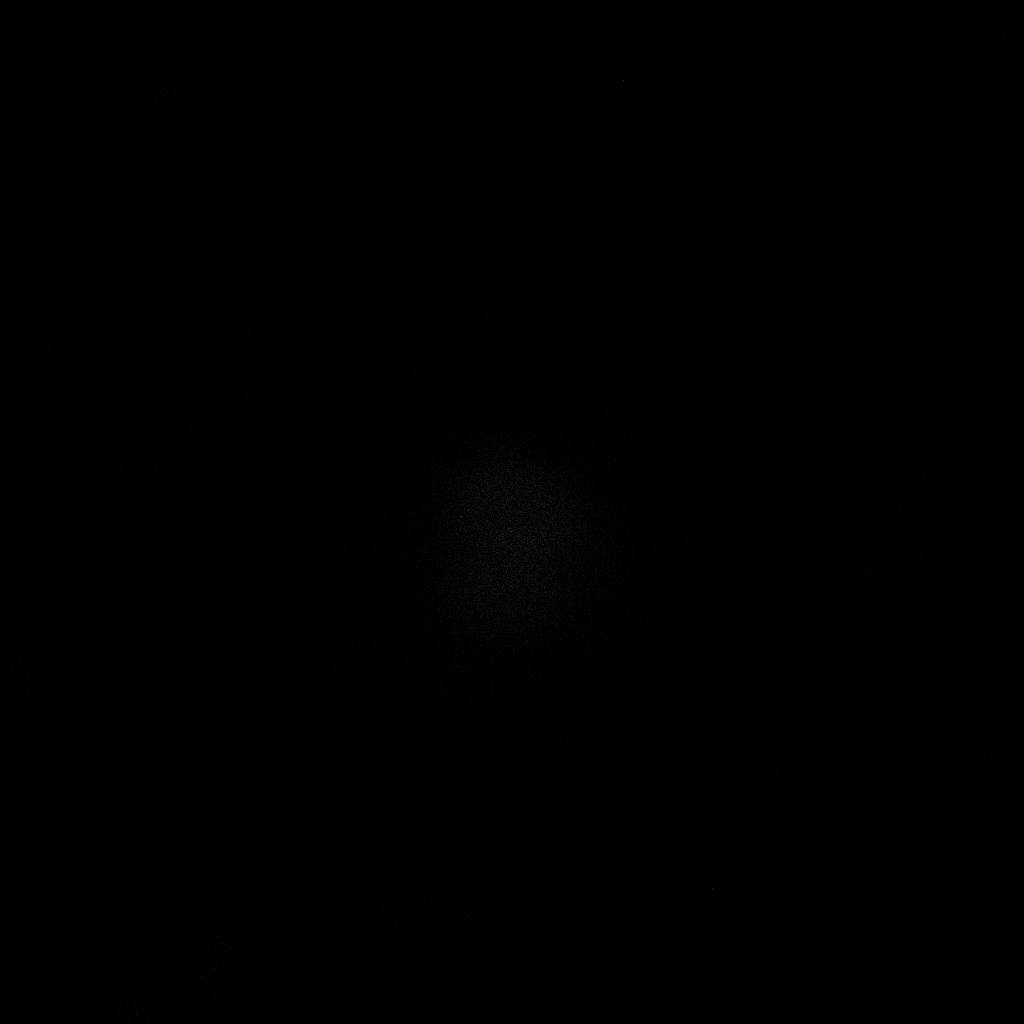

Supplement: Supplementary file 8 — Source data Fig. 1 [file 44319_2025_567_MOESM8_ESM.zip › Fig1/1B/CEM-NUP358-cores_RAW_ch03.tif]

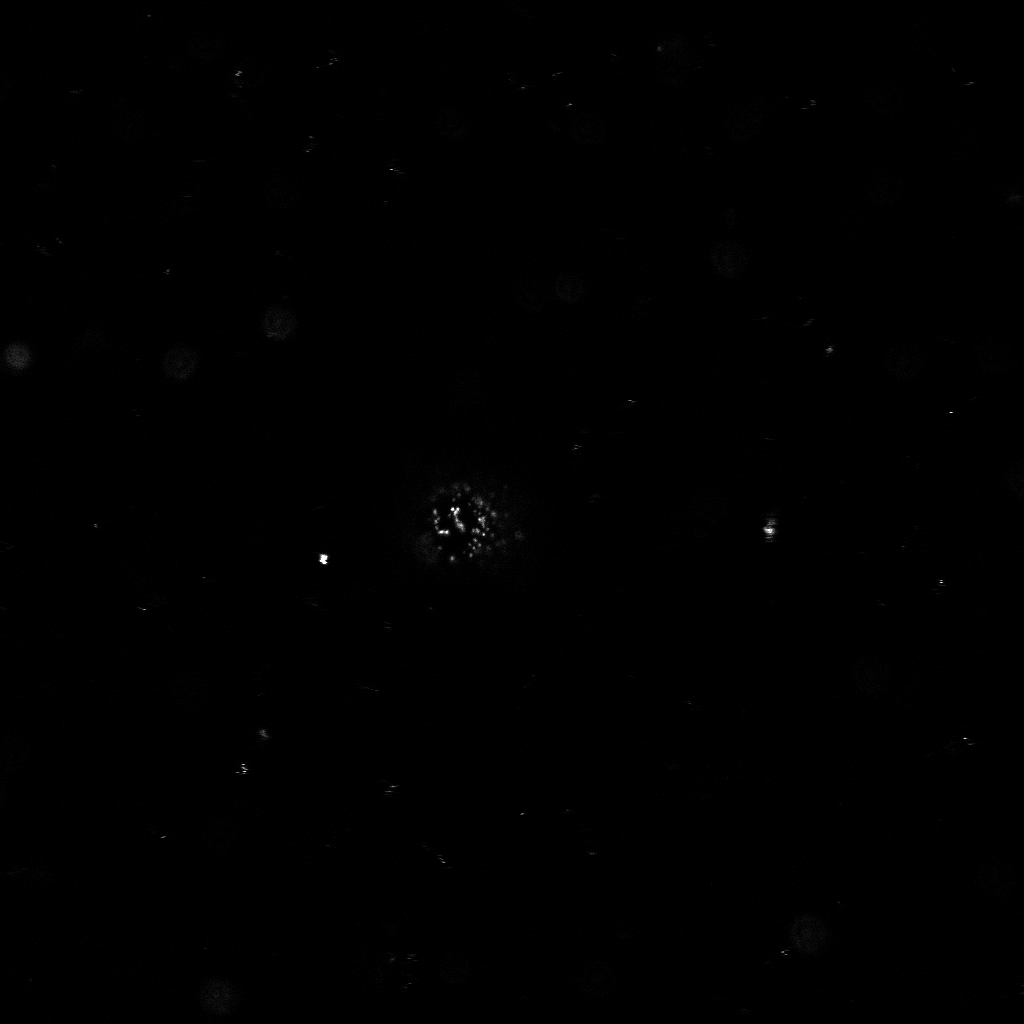

Supplement: Supplementary file 8 — Source data Fig. 1 [file 44319_2025_567_MOESM8_ESM.zip › Fig1/1C/CEM-cores_permeabilized_RAW_ch00.tif]

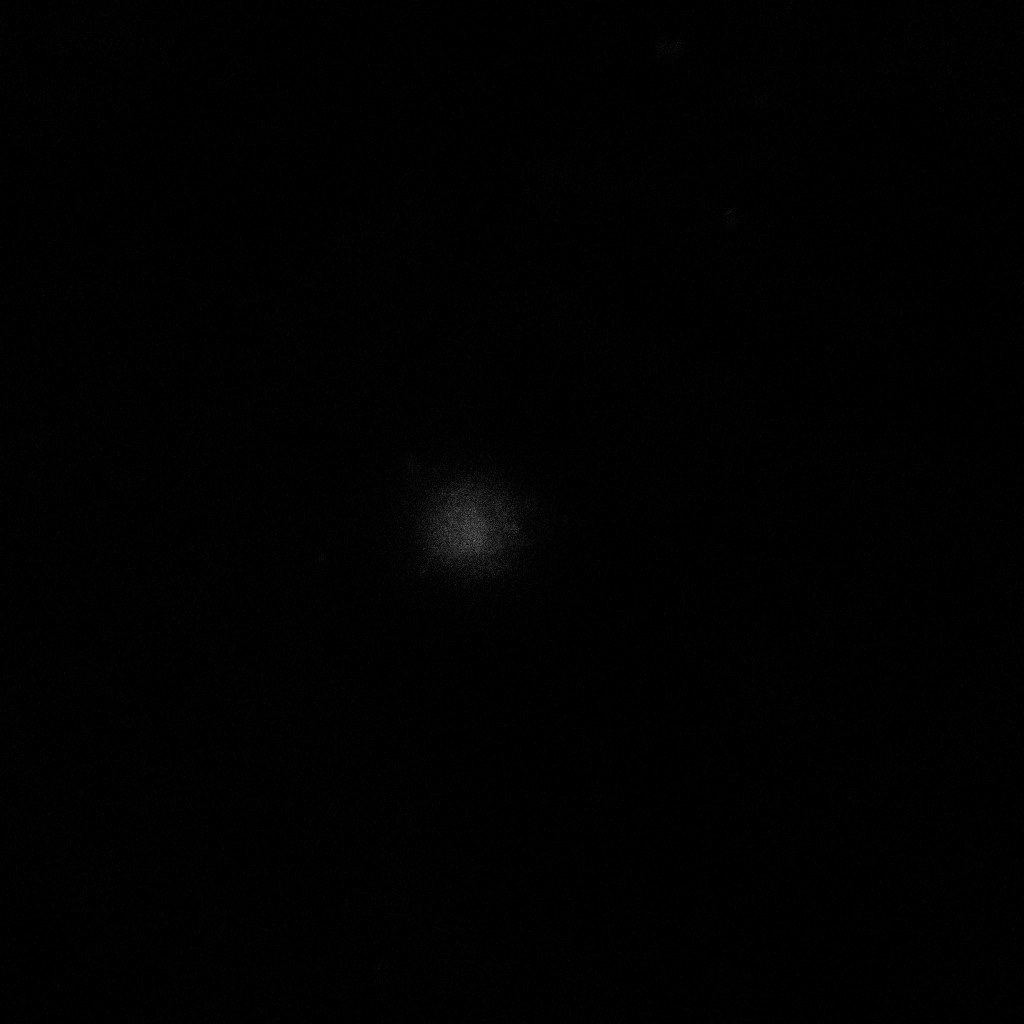

Supplement: Supplementary file 8 — Source data Fig. 1 [file 44319_2025_567_MOESM8_ESM.zip › Fig1/1C/CEM-cores_permeabilized_RAW_ch01.tif]

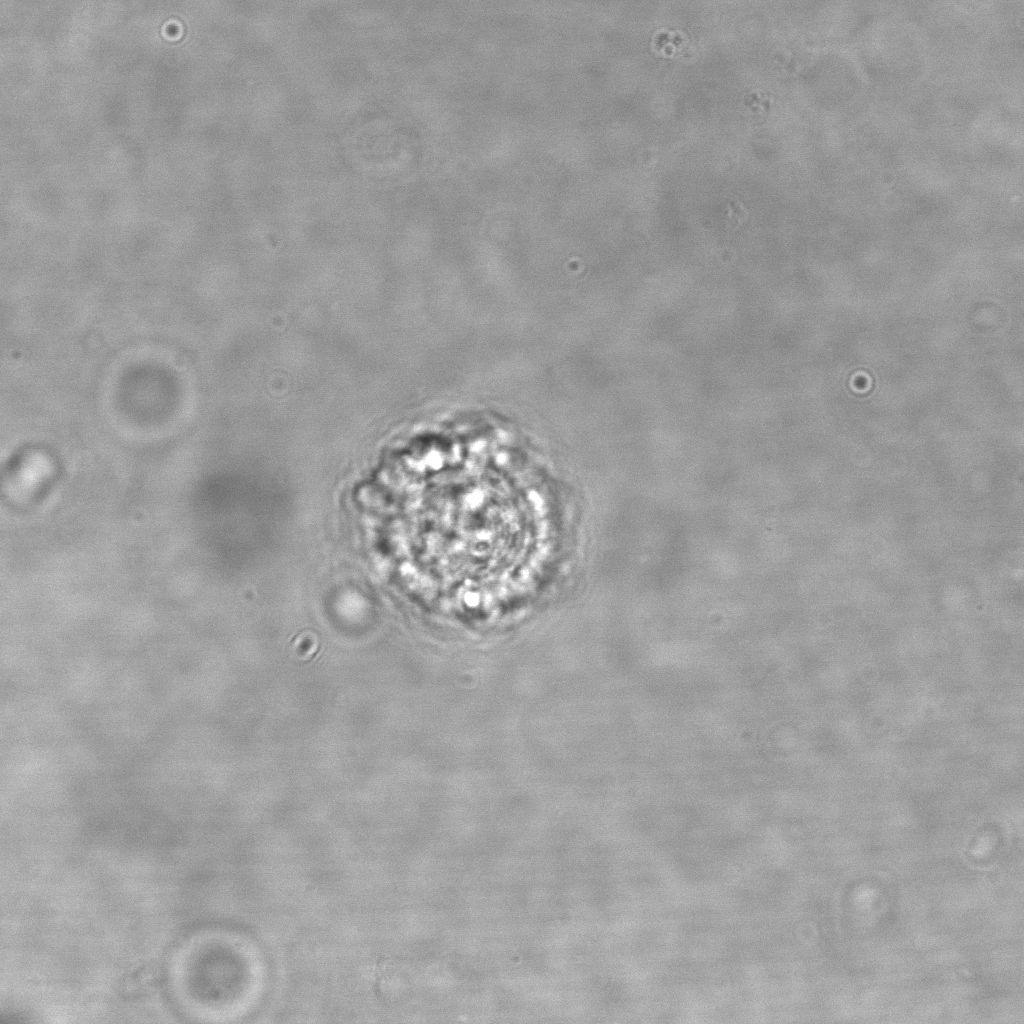

Supplement: Supplementary file 8 — Source data Fig. 1 [file 44319_2025_567_MOESM8_ESM.zip › Fig1/1C/CEM-cores_permeabilized_RAW_ch02.tif]

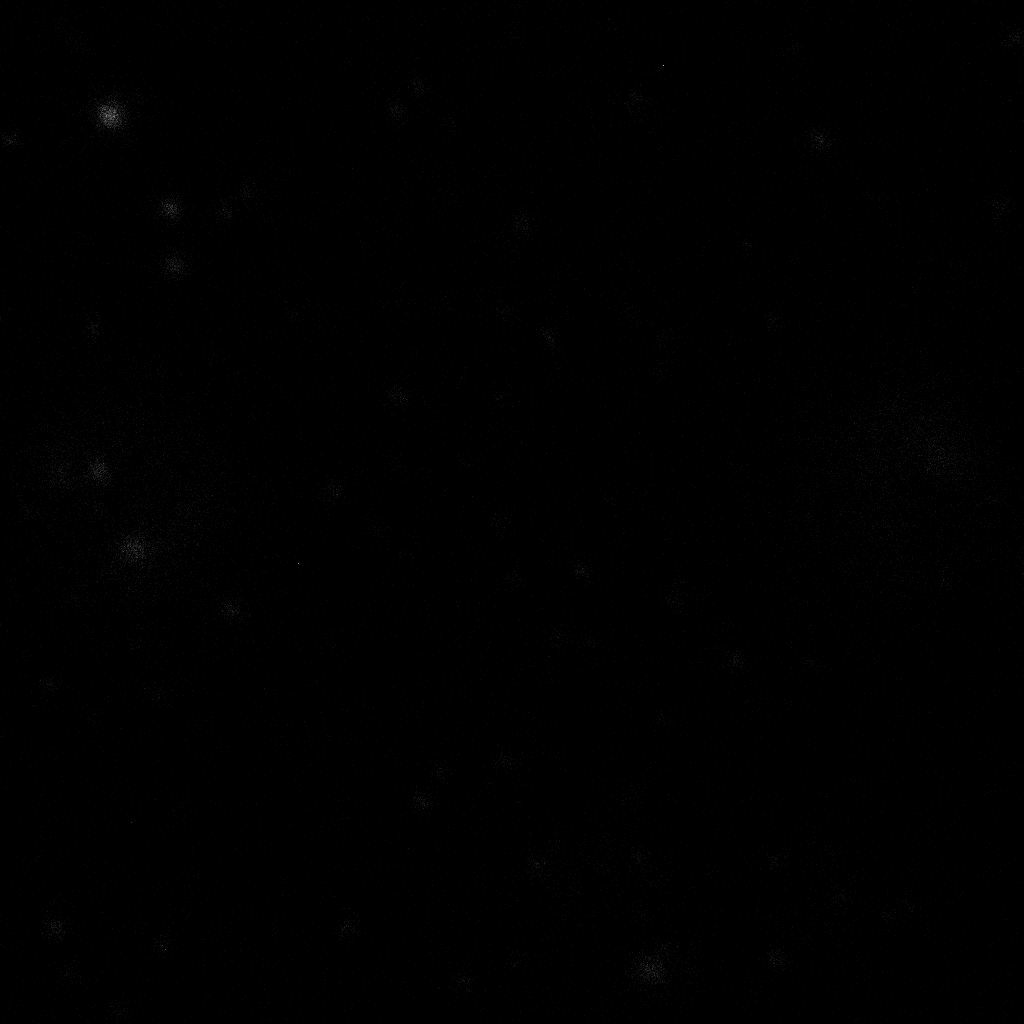

Supplement: Supplementary file 8 — Source data Fig. 1 [file 44319_2025_567_MOESM8_ESM.zip › Fig1/1D/Counted_nuclei/Mechanical/Nuc_1+2/Nuc_1+2_z00_RAW_ch00.tif]

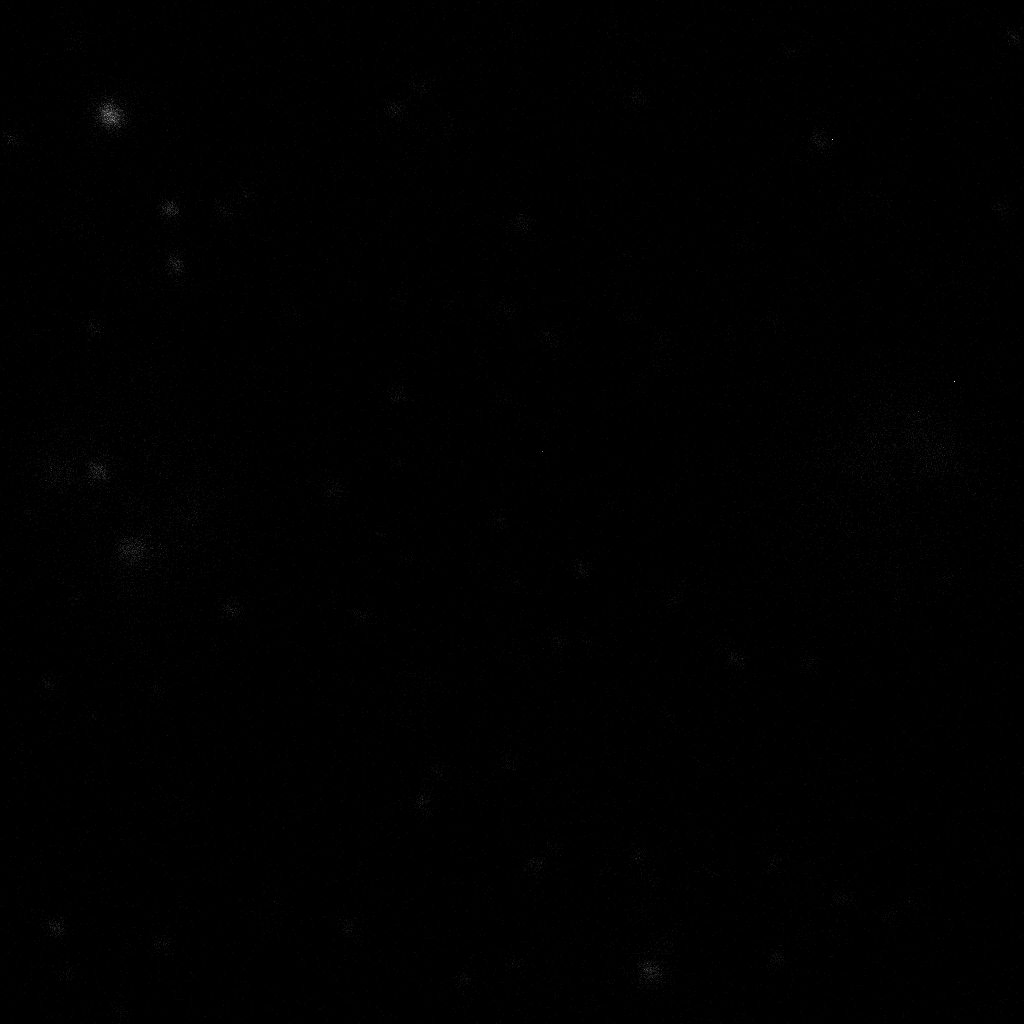

Supplement: Supplementary file 8 — Source data Fig. 1 [file 44319_2025_567_MOESM8_ESM.zip › Fig1/1D/Counted_nuclei/Mechanical/Nuc_1+2/Nuc_1+2_z01_RAW_ch00.tif]

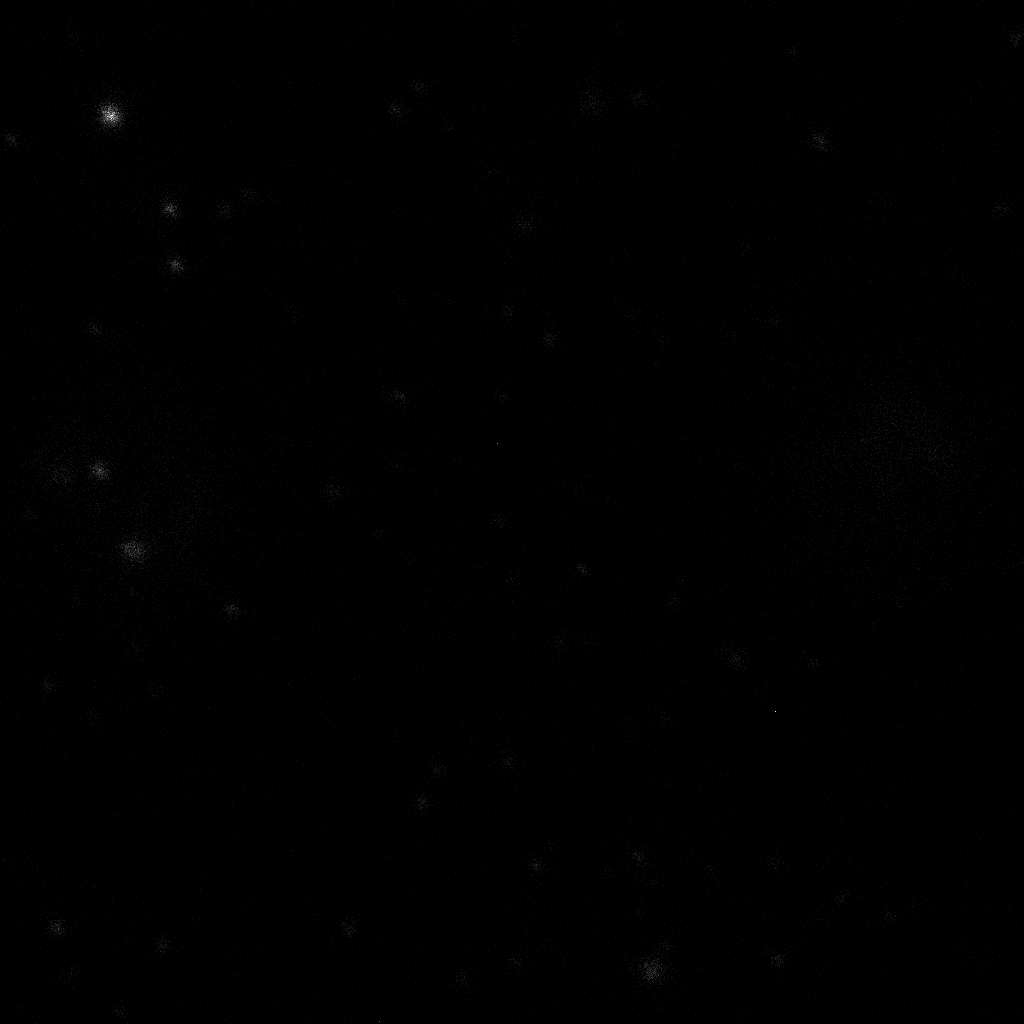

Supplement: Supplementary file 8 — Source data Fig. 1 [file 44319_2025_567_MOESM8_ESM.zip › Fig1/1D/Counted_nuclei/Mechanical/Nuc_1+2/Nuc_1+2_z02_RAW_ch00.tif]

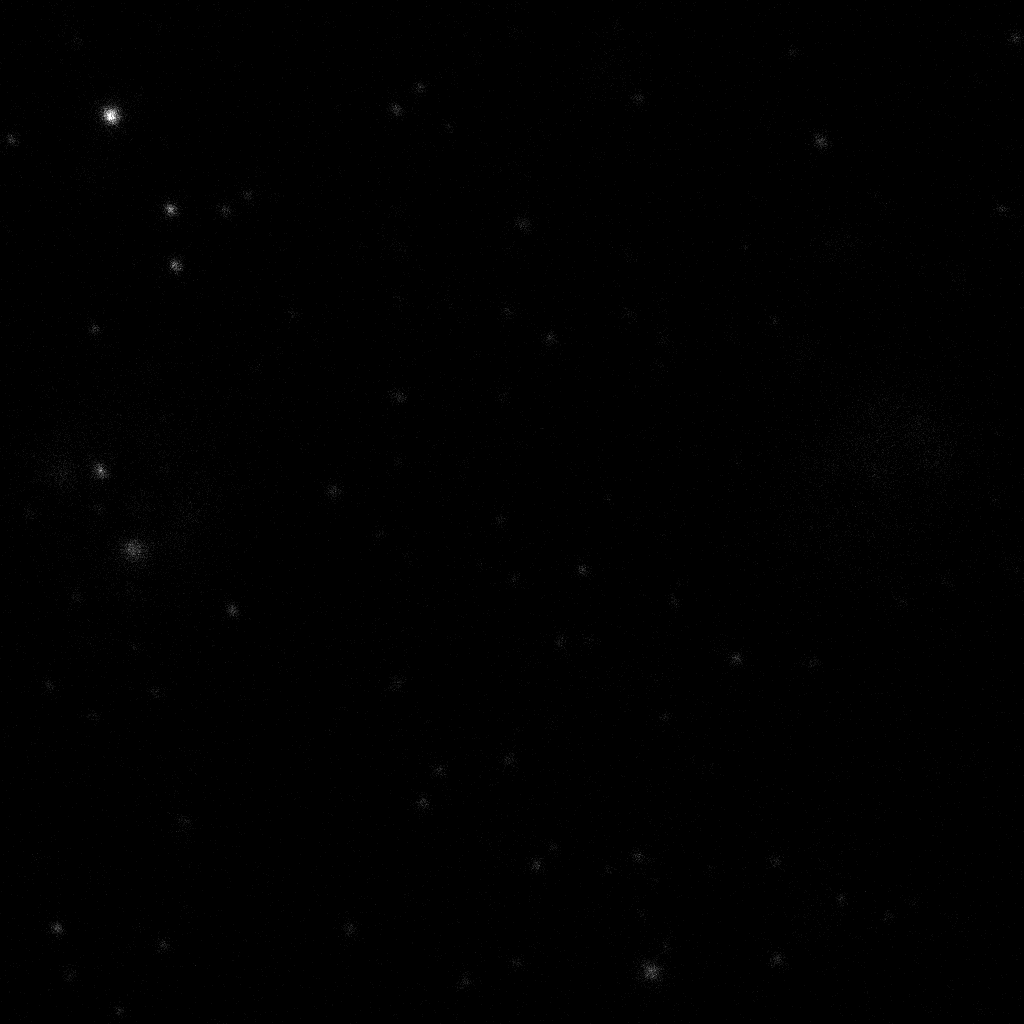

Supplement: Supplementary file 8 — Source data Fig. 1 [file 44319_2025_567_MOESM8_ESM.zip › Fig1/1D/Counted_nuclei/Mechanical/Nuc_1+2/Nuc_1+2_z03_RAW_ch00.tif]

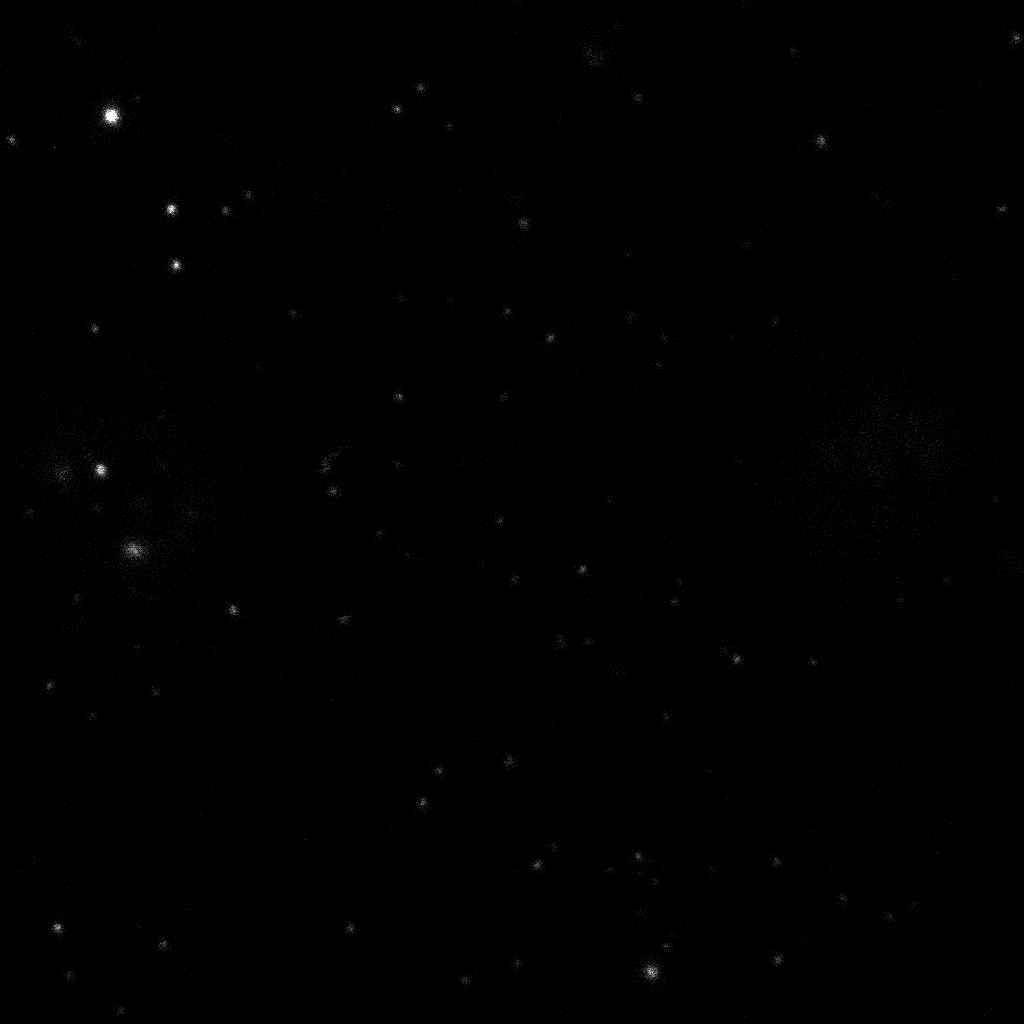

Supplement: Supplementary file 8 — Source data Fig. 1 [file 44319_2025_567_MOESM8_ESM.zip › Fig1/1D/Counted_nuclei/Mechanical/Nuc_1+2/Nuc_1+2_z04_RAW_ch00.tif]

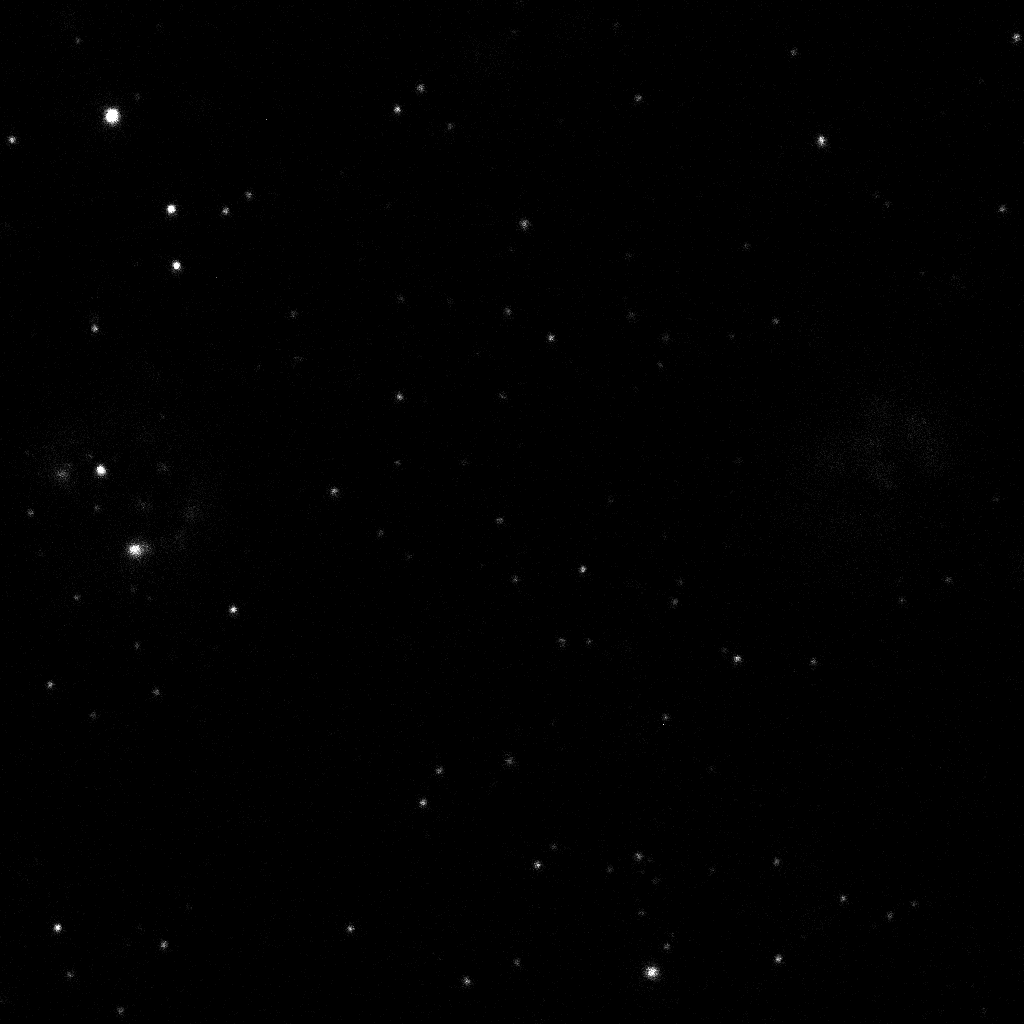

Supplement: Supplementary file 8 — Source data Fig. 1 [file 44319_2025_567_MOESM8_ESM.zip › Fig1/1D/Counted_nuclei/Mechanical/Nuc_1+2/Nuc_1+2_z05_RAW_ch00.tif]

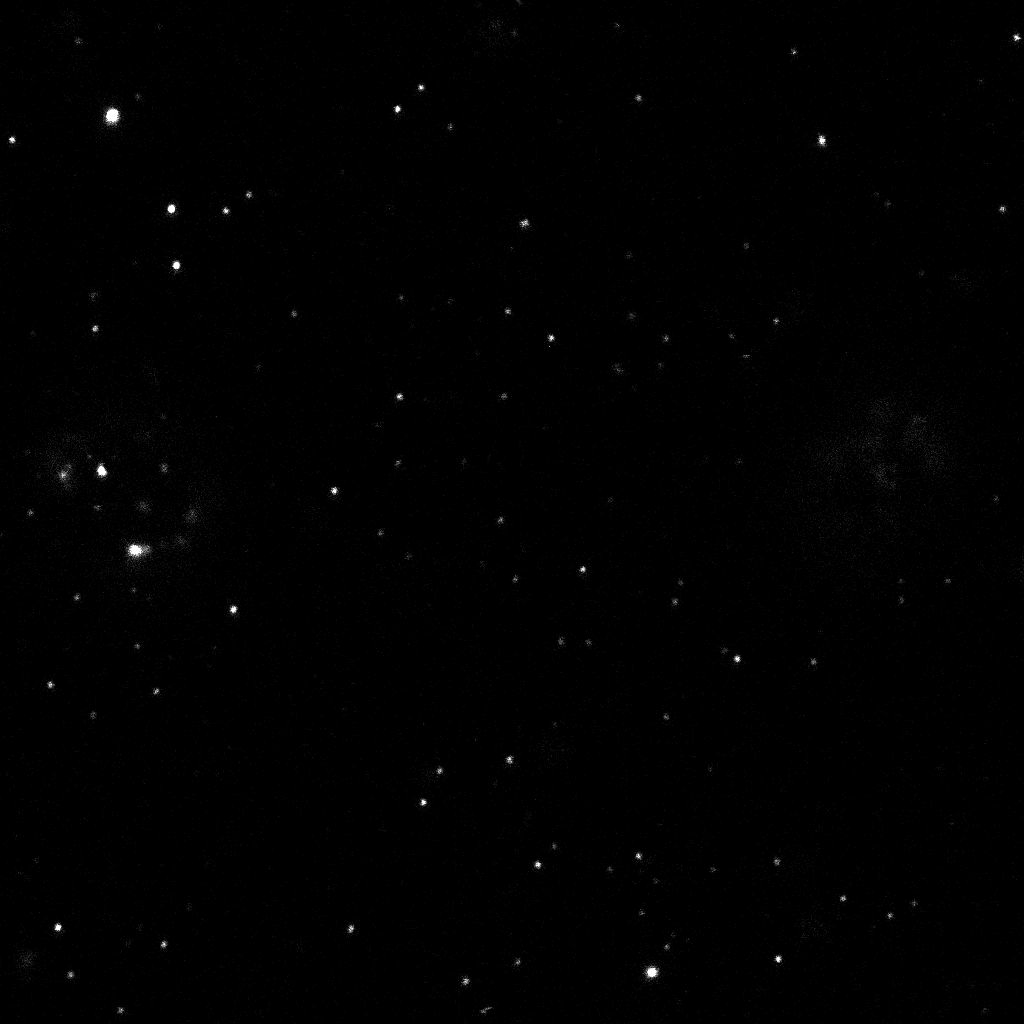

Supplement: Supplementary file 8 — Source data Fig. 1 [file 44319_2025_567_MOESM8_ESM.zip › Fig1/1D/Counted_nuclei/Mechanical/Nuc_1+2/Nuc_1+2_z06_RAW_ch00.tif]

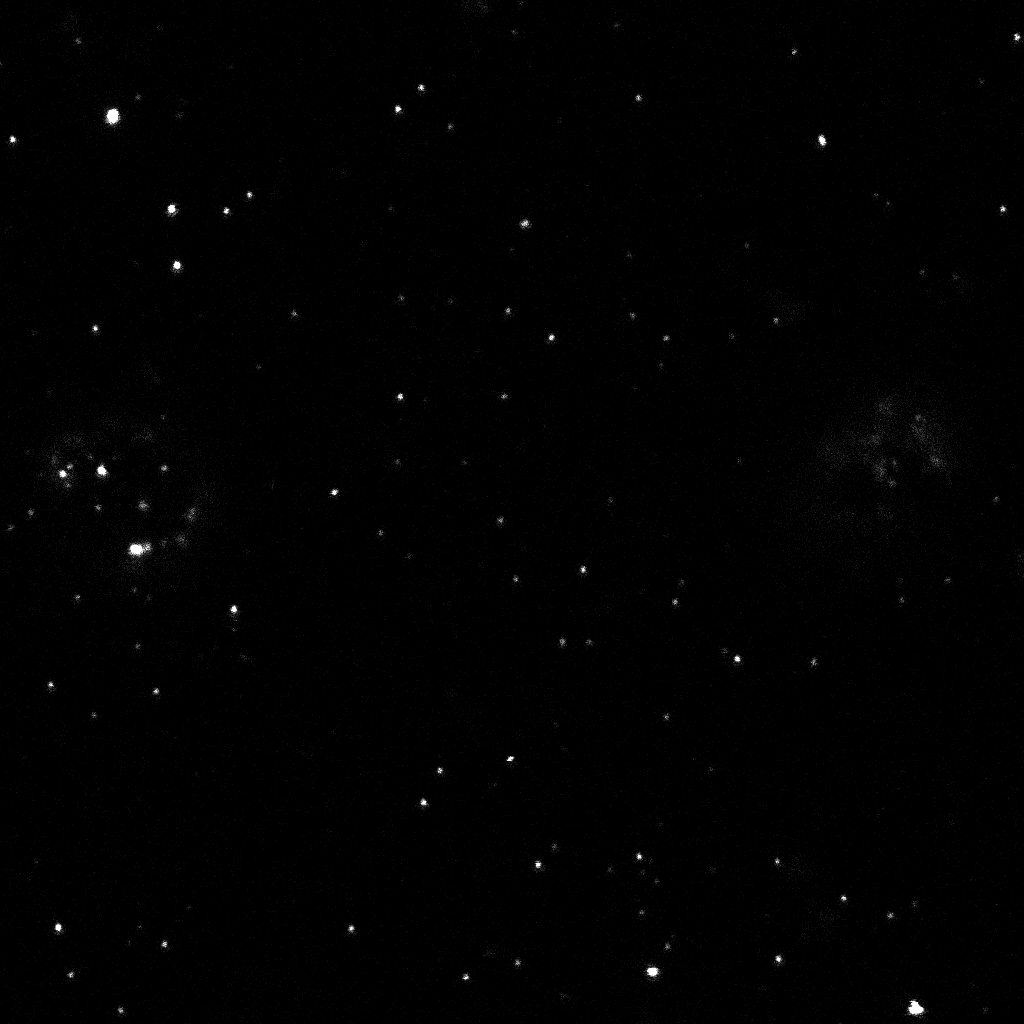

Supplement: Supplementary file 8 — Source data Fig. 1 [file 44319_2025_567_MOESM8_ESM.zip › Fig1/1D/Counted_nuclei/Mechanical/Nuc_1+2/Nuc_1+2_z07_RAW_ch00.tif]

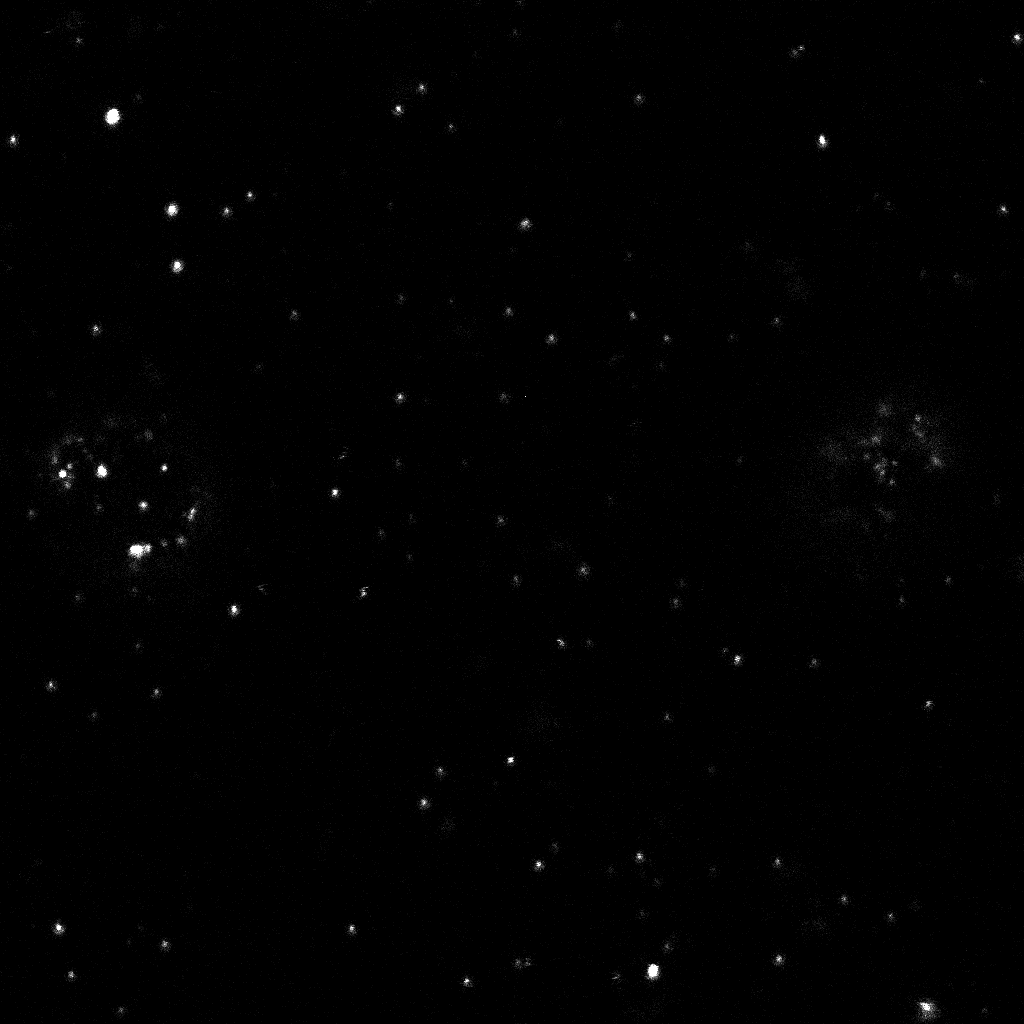

Supplement: Supplementary file 8 — Source data Fig. 1 [file 44319_2025_567_MOESM8_ESM.zip › Fig1/1D/Counted_nuclei/Mechanical/Nuc_1+2/Nuc_1+2_z08_RAW_ch00.tif]

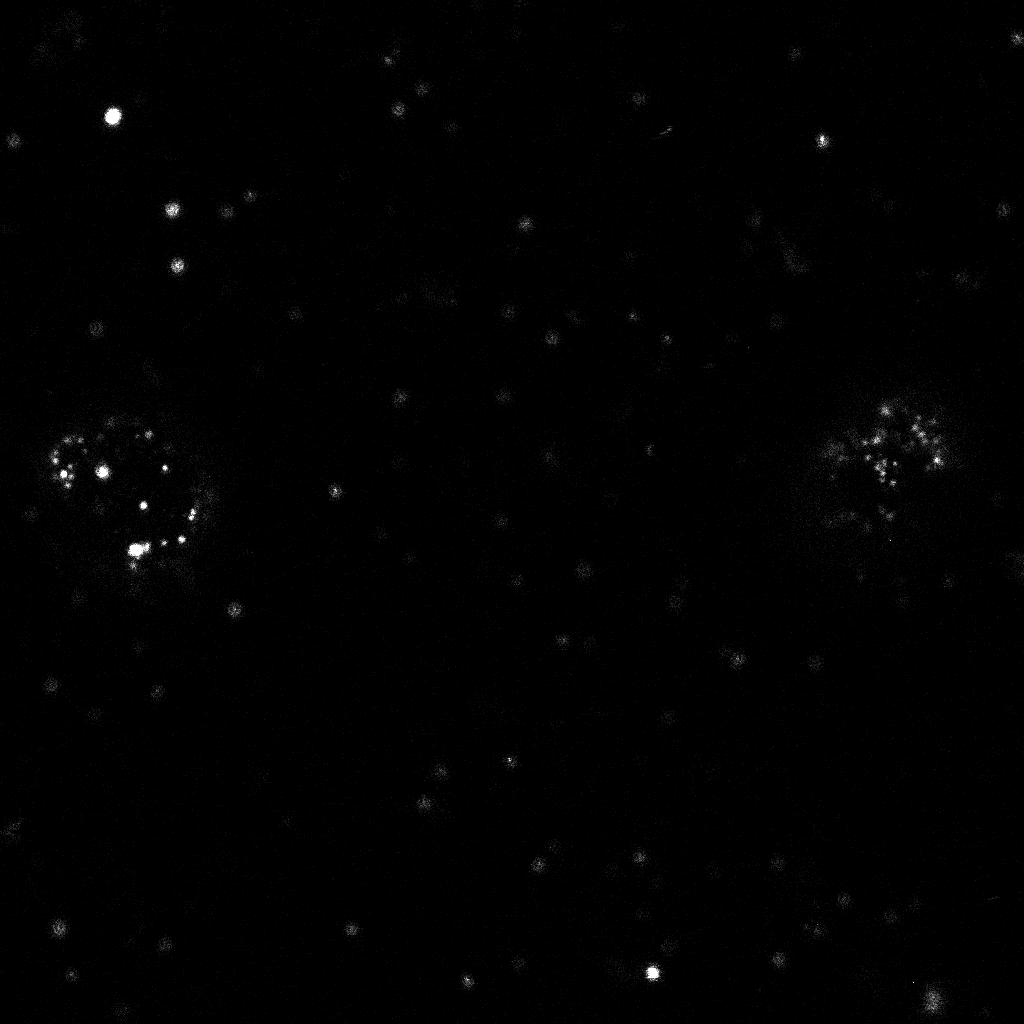

Supplement: Supplementary file 8 — Source data Fig. 1 [file 44319_2025_567_MOESM8_ESM.zip › Fig1/1D/Counted_nuclei/Mechanical/Nuc_1+2/Nuc_1+2_z09_RAW_ch00.tif]

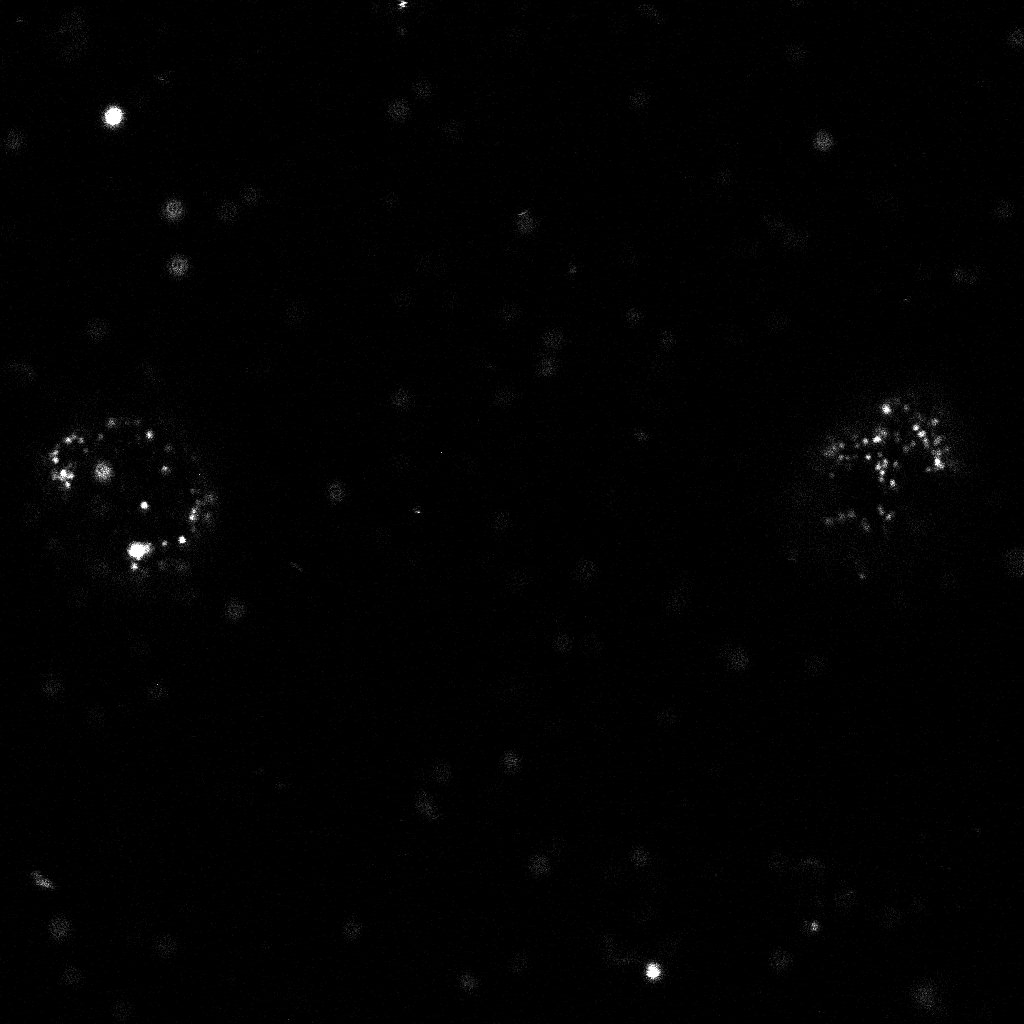

Supplement: Supplementary file 8 — Source data Fig. 1 [file 44319_2025_567_MOESM8_ESM.zip › Fig1/1D/Counted_nuclei/Mechanical/Nuc_1+2/Nuc_1+2_z10_RAW_ch00.tif]

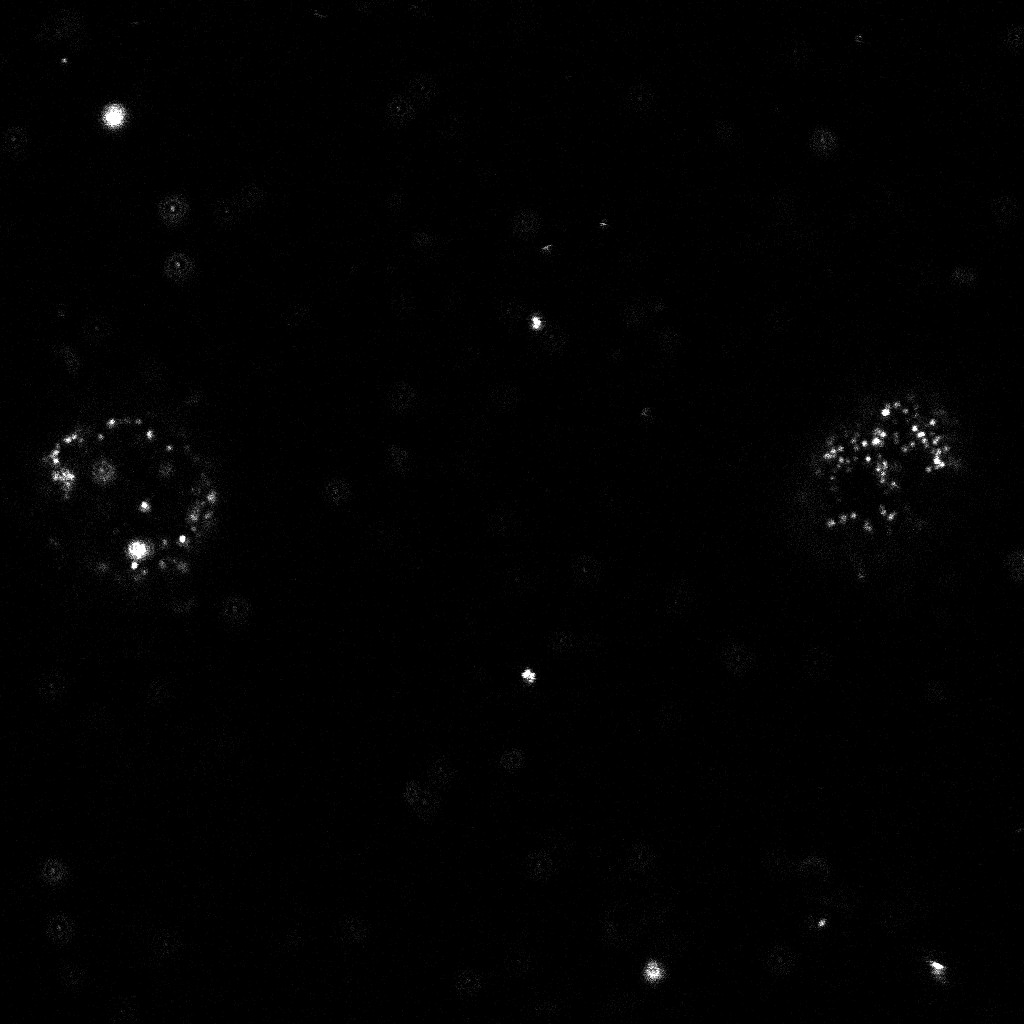

Supplement: Supplementary file 8 — Source data Fig. 1 [file 44319_2025_567_MOESM8_ESM.zip › Fig1/1D/Counted_nuclei/Mechanical/Nuc_1+2/Nuc_1+2_z11_RAW_ch00.tif]

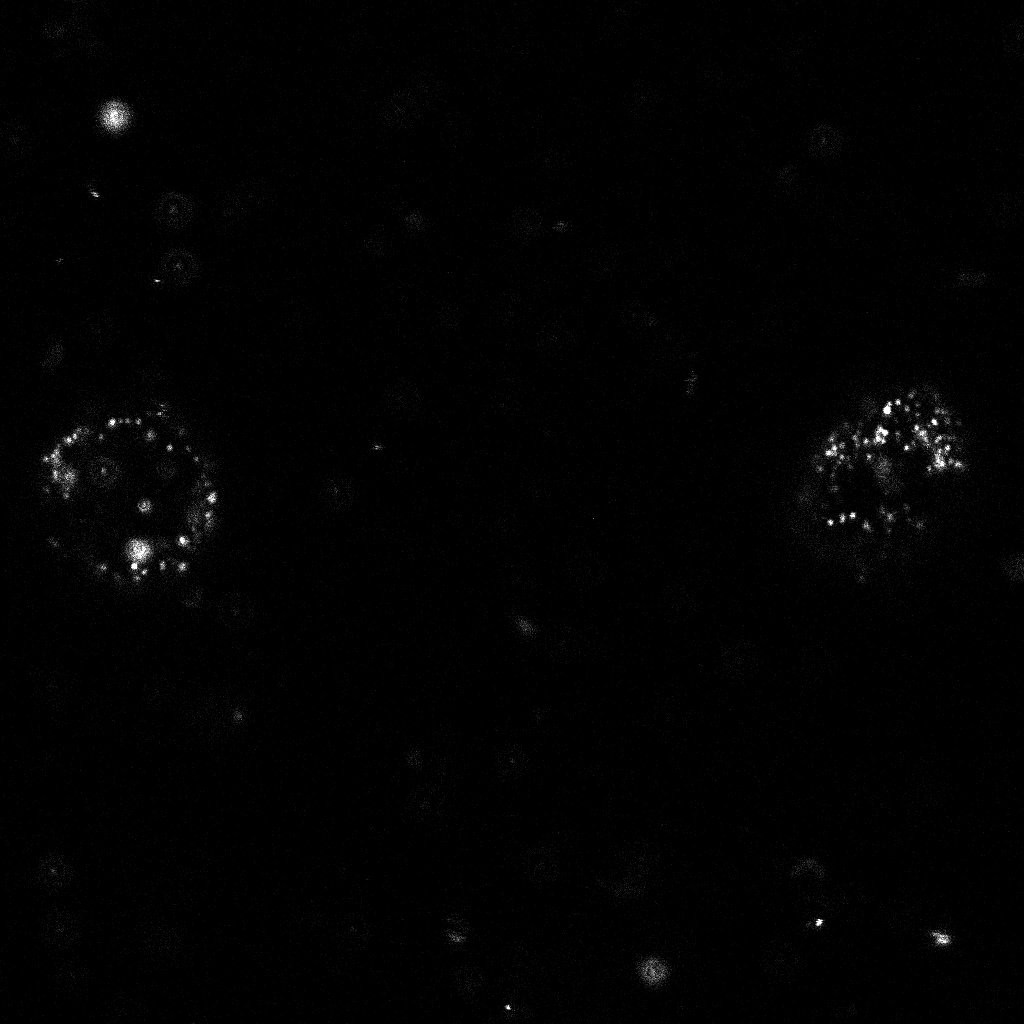

Supplement: Supplementary file 8 — Source data Fig. 1 [file 44319_2025_567_MOESM8_ESM.zip › Fig1/1D/Counted_nuclei/Mechanical/Nuc_1+2/Nuc_1+2_z12_RAW_ch00.tif]

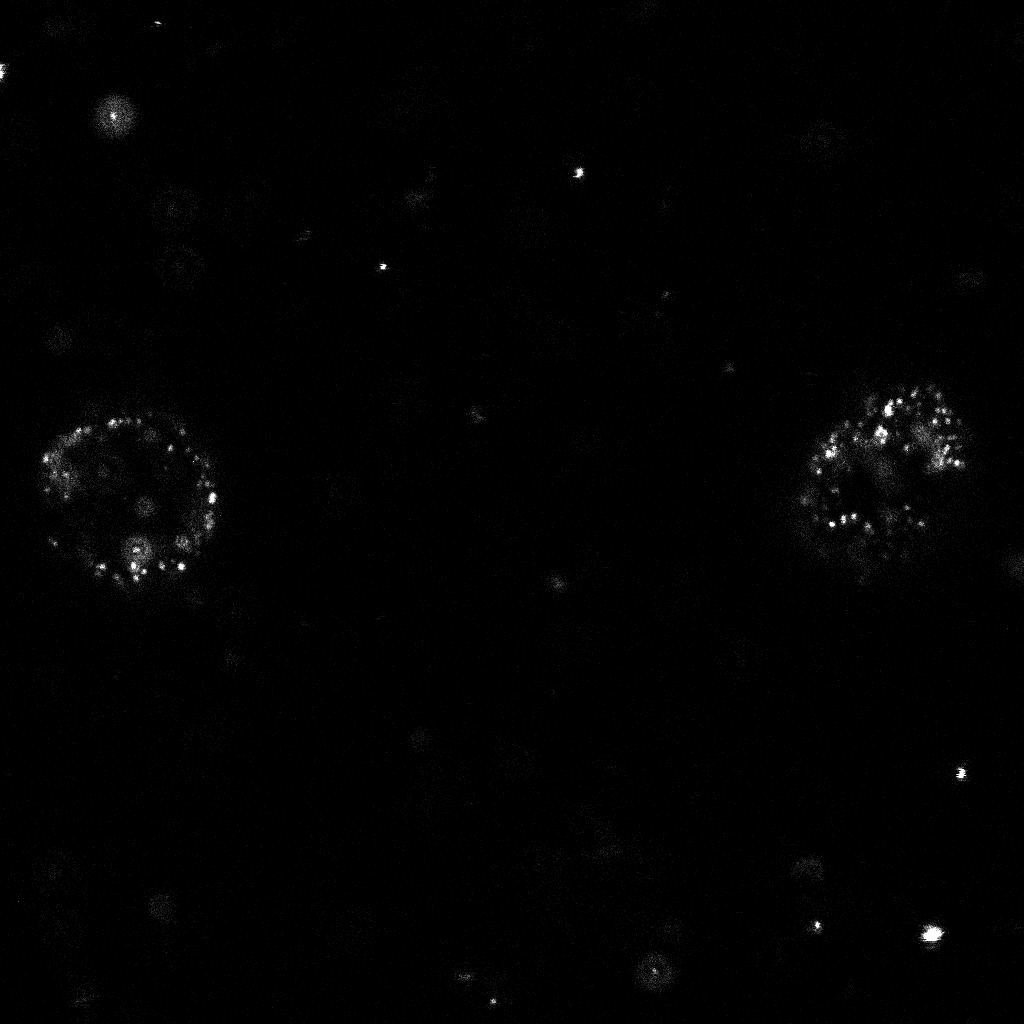

Supplement: Supplementary file 8 — Source data Fig. 1 [file 44319_2025_567_MOESM8_ESM.zip › Fig1/1D/Counted_nuclei/Mechanical/Nuc_1+2/Nuc_1+2_z13_RAW_ch00.tif]

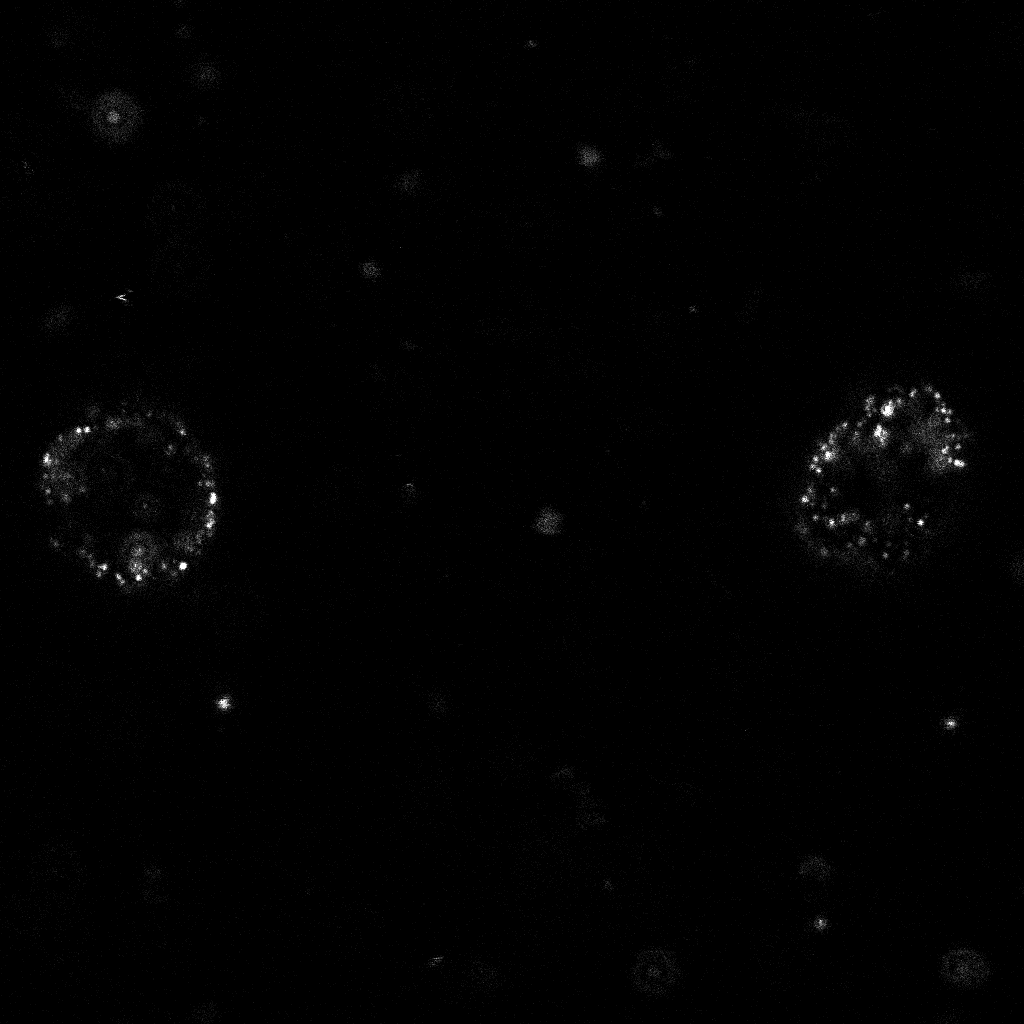

Supplement: Supplementary file 8 — Source data Fig. 1 [file 44319_2025_567_MOESM8_ESM.zip › Fig1/1D/Counted_nuclei/Mechanical/Nuc_1+2/Nuc_1+2_z14_RAW_ch00.tif]

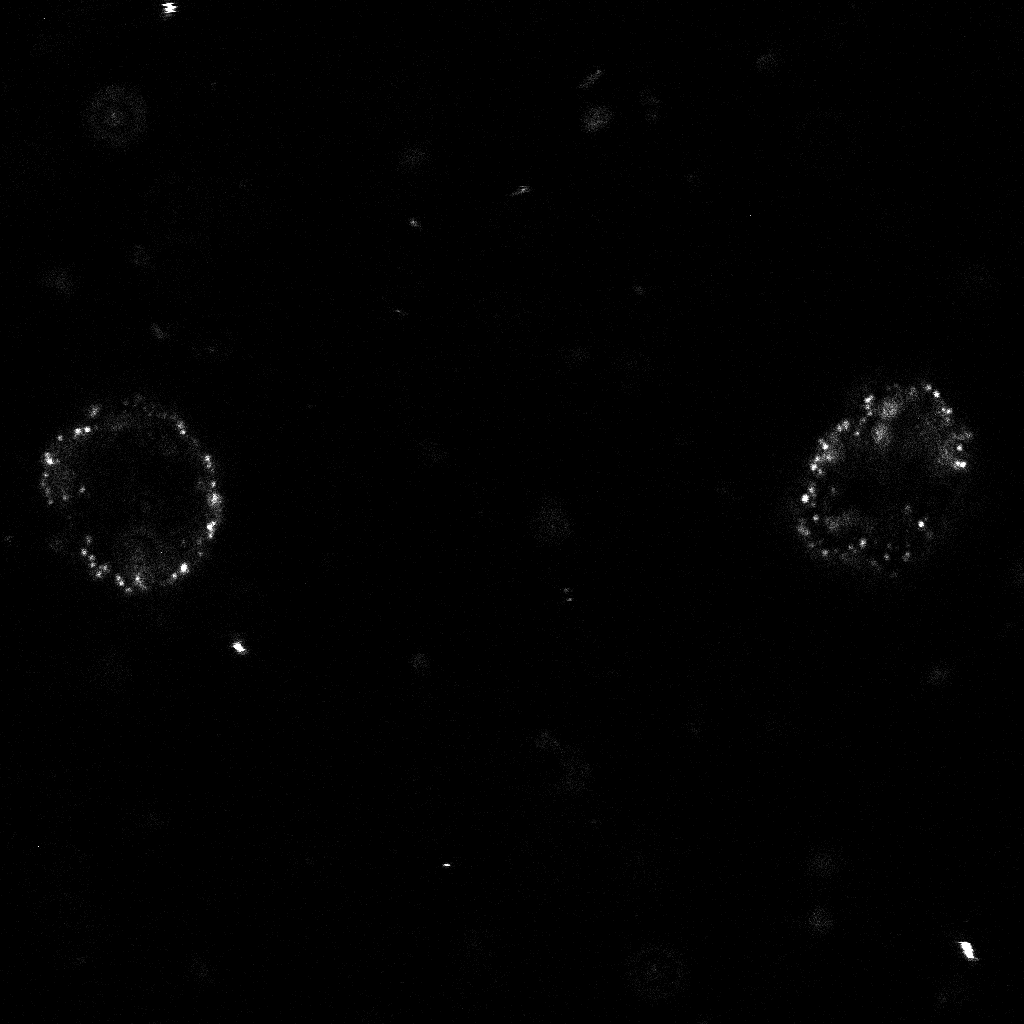

Supplement: Supplementary file 8 — Source data Fig. 1 [file 44319_2025_567_MOESM8_ESM.zip › Fig1/1D/Counted_nuclei/Mechanical/Nuc_1+2/Nuc_1+2_z15_RAW_ch00.tif]

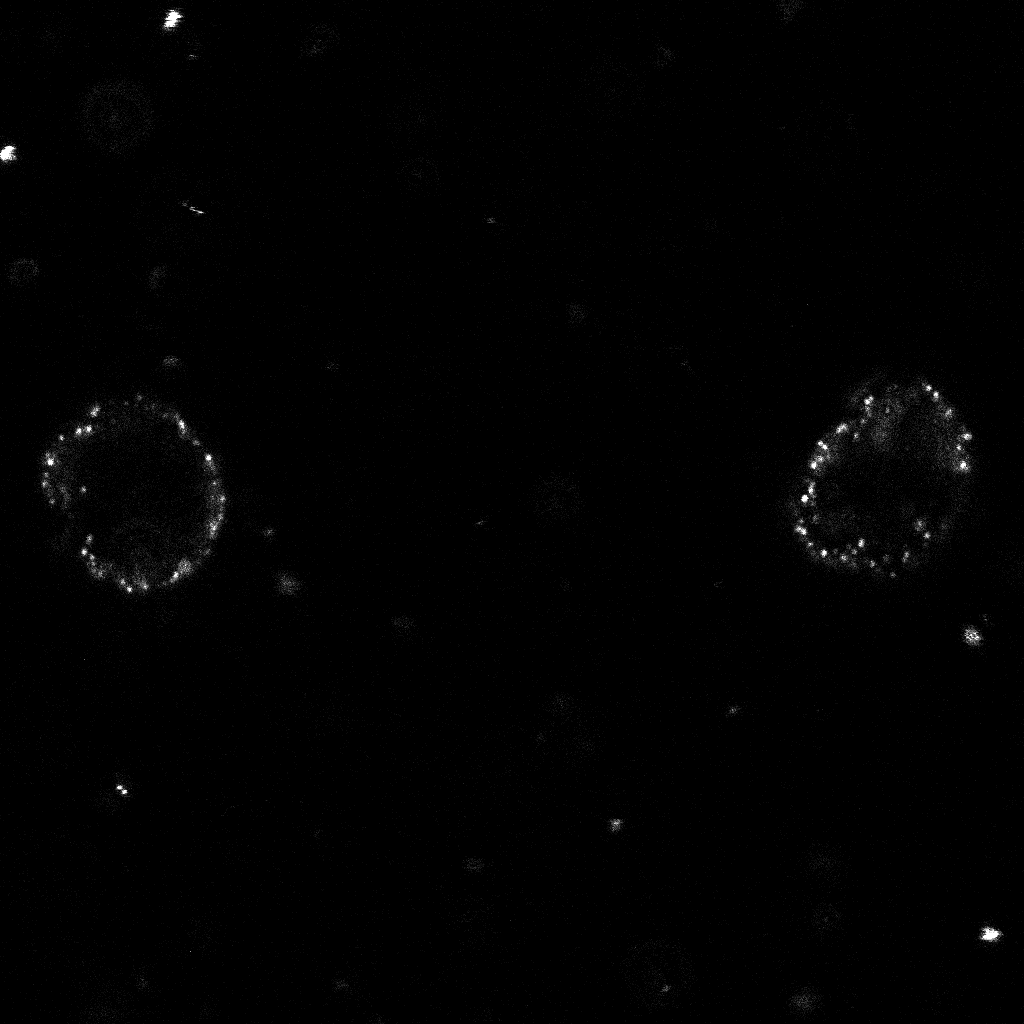

Supplement: Supplementary file 8 — Source data Fig. 1 [file 44319_2025_567_MOESM8_ESM.zip › Fig1/1D/Counted_nuclei/Mechanical/Nuc_1+2/Nuc_1+2_z16_RAW_ch00.tif]

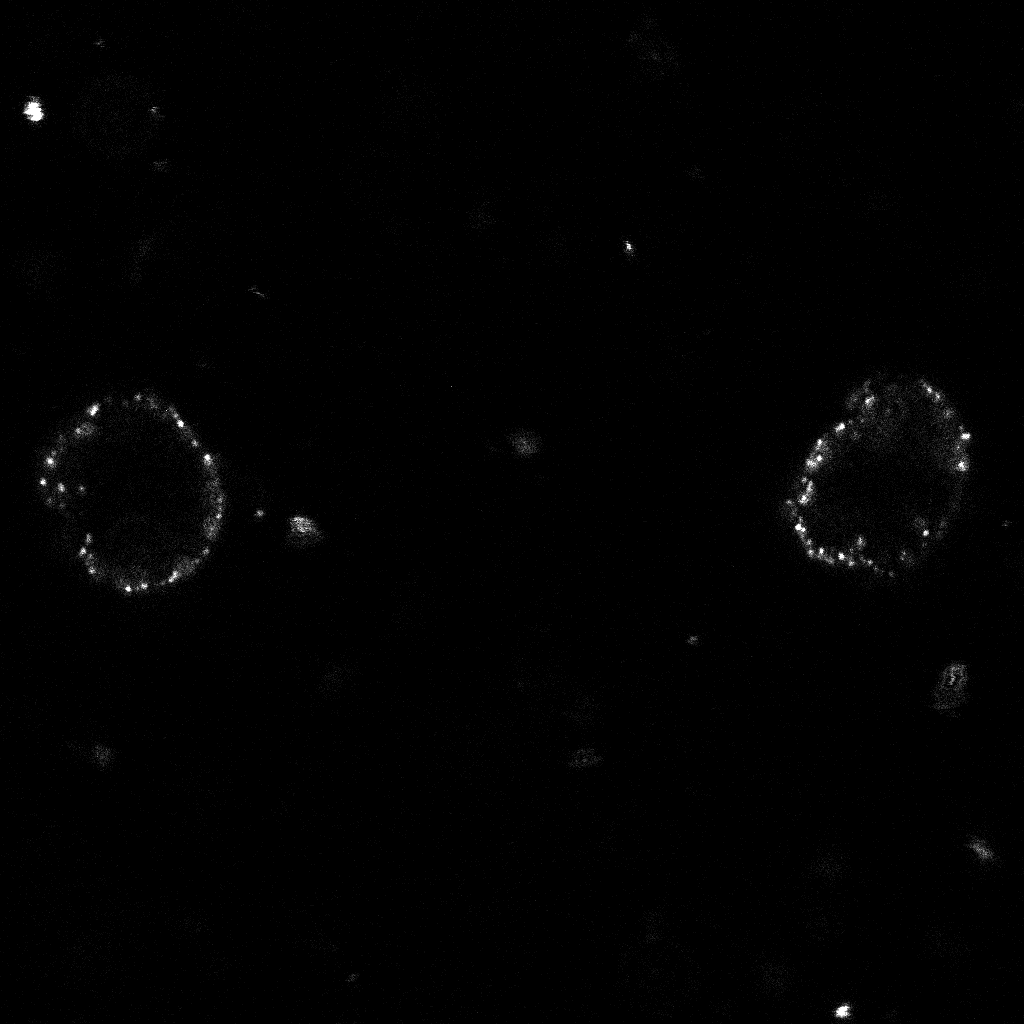

Supplement: Supplementary file 8 — Source data Fig. 1 [file 44319_2025_567_MOESM8_ESM.zip › Fig1/1D/Counted_nuclei/Mechanical/Nuc_1+2/Nuc_1+2_z17_RAW_ch00.tif]

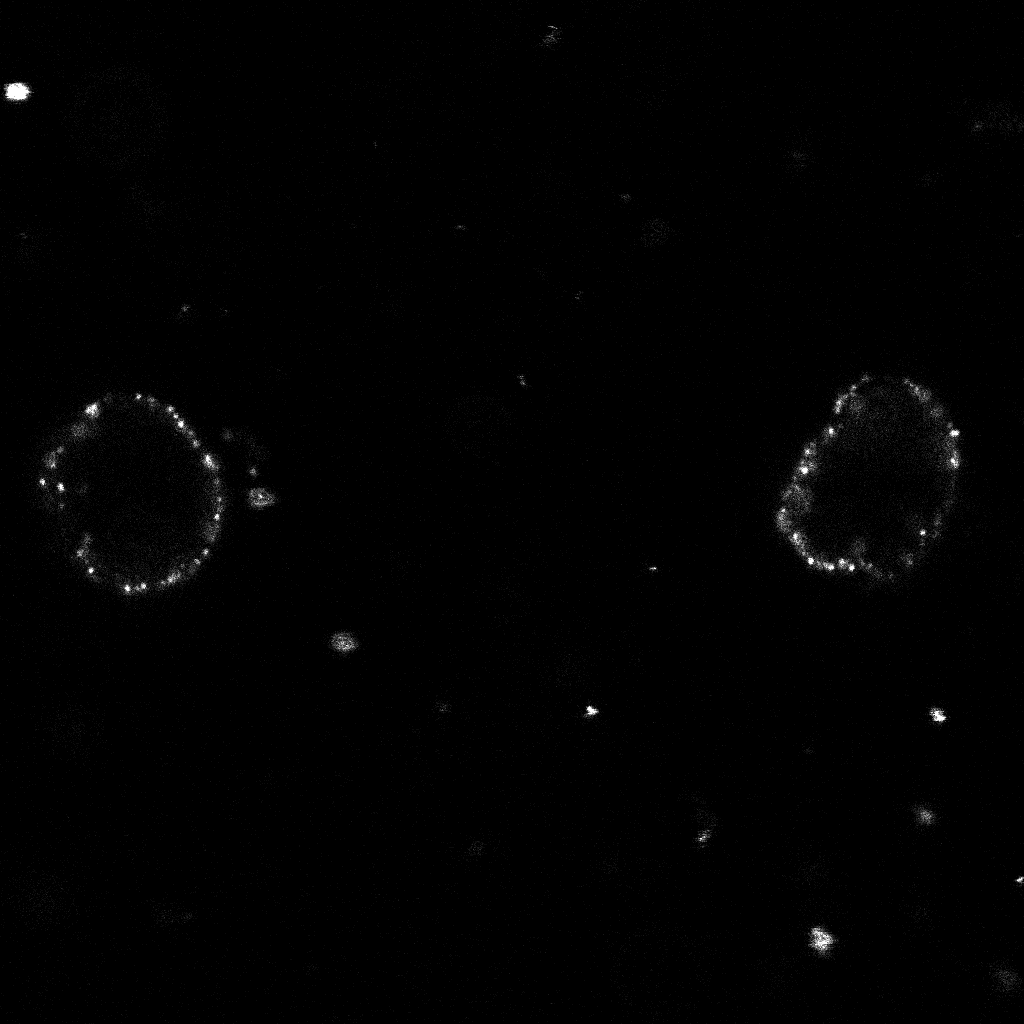

Supplement: Supplementary file 8 — Source data Fig. 1 [file 44319_2025_567_MOESM8_ESM.zip › Fig1/1D/Counted_nuclei/Mechanical/Nuc_1+2/Nuc_1+2_z18_RAW_ch00.tif]

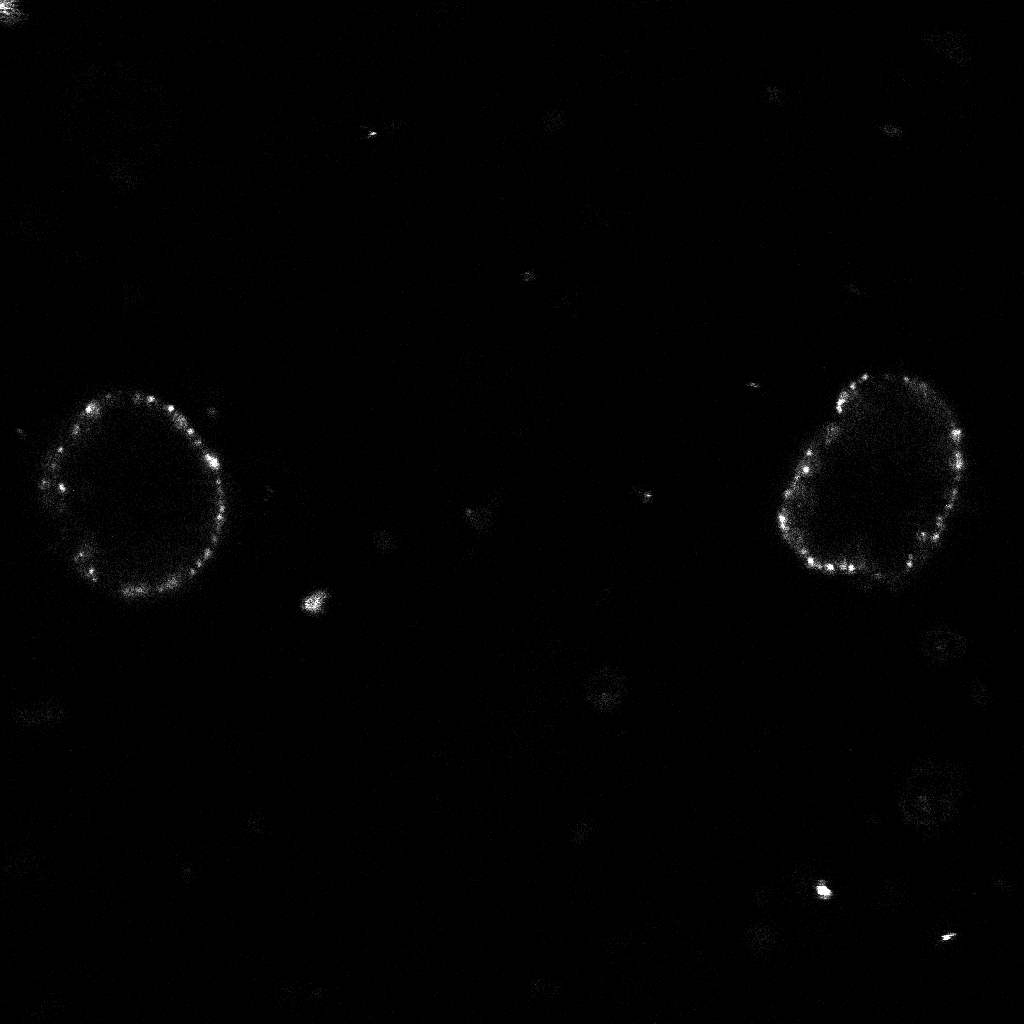

Supplement: Supplementary file 8 — Source data Fig. 1 [file 44319_2025_567_MOESM8_ESM.zip › Fig1/1D/Counted_nuclei/Mechanical/Nuc_1+2/Nuc_1+2_z19_RAW_ch00.tif]

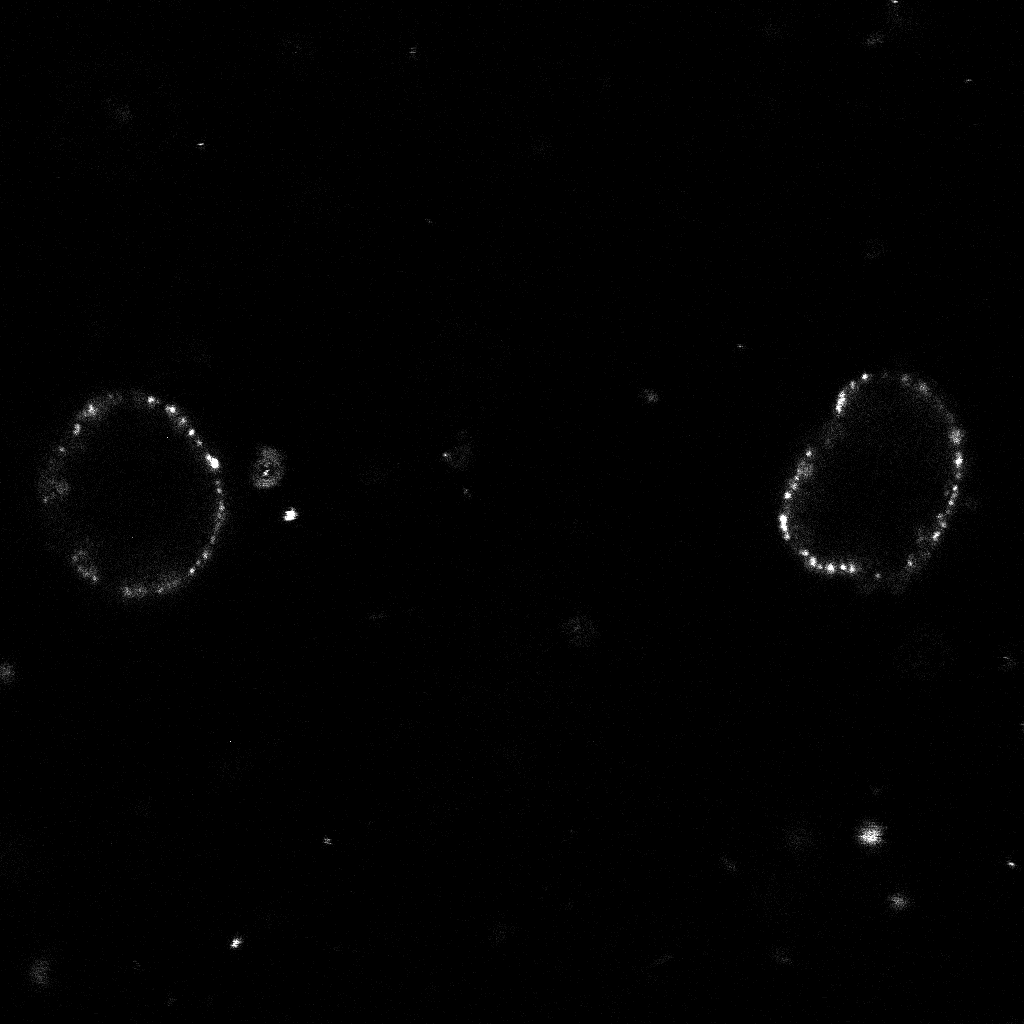

Supplement: Supplementary file 8 — Source data Fig. 1 [file 44319_2025_567_MOESM8_ESM.zip › Fig1/1D/Counted_nuclei/Mechanical/Nuc_1+2/Nuc_1+2_z20_RAW_ch00.tif]

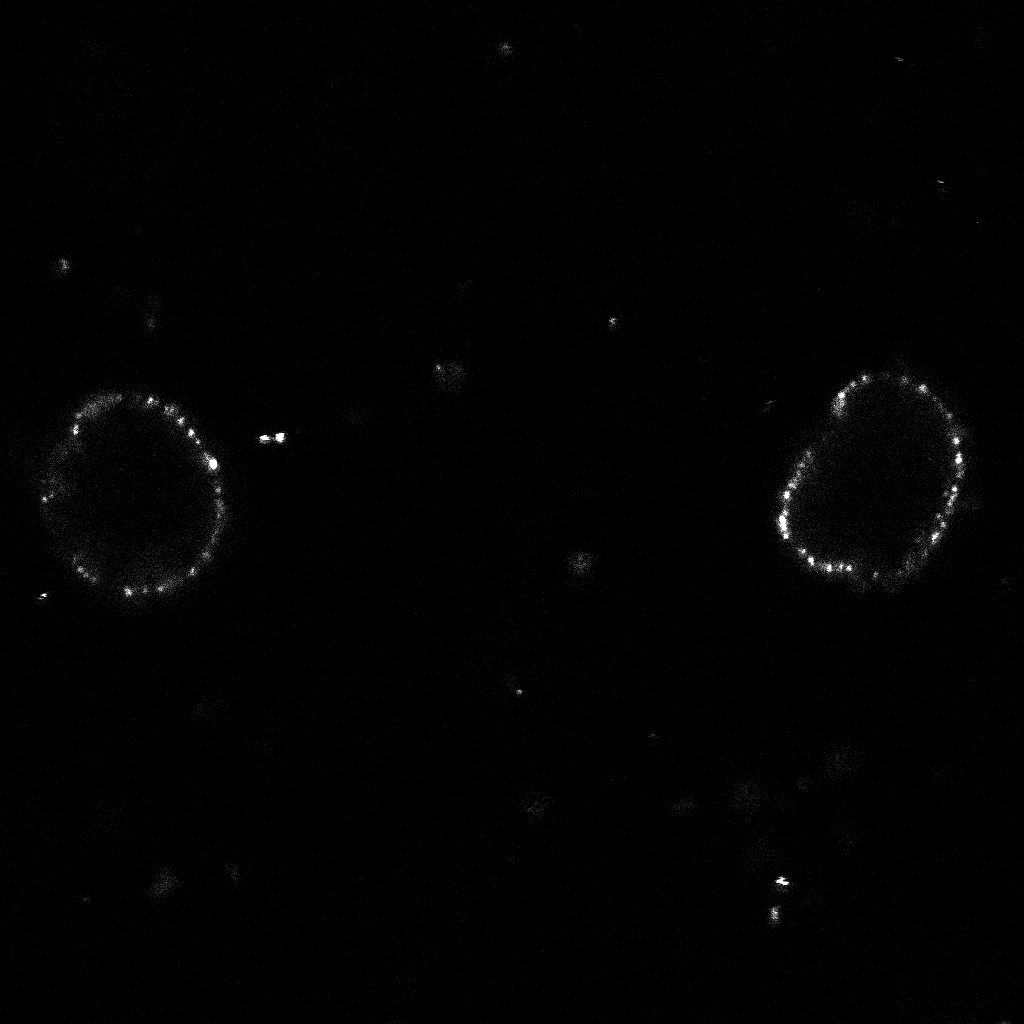

Supplement: Supplementary file 8 — Source data Fig. 1 [file 44319_2025_567_MOESM8_ESM.zip › Fig1/1D/Counted_nuclei/Mechanical/Nuc_1+2/Nuc_1+2_z21_RAW_ch00.tif]

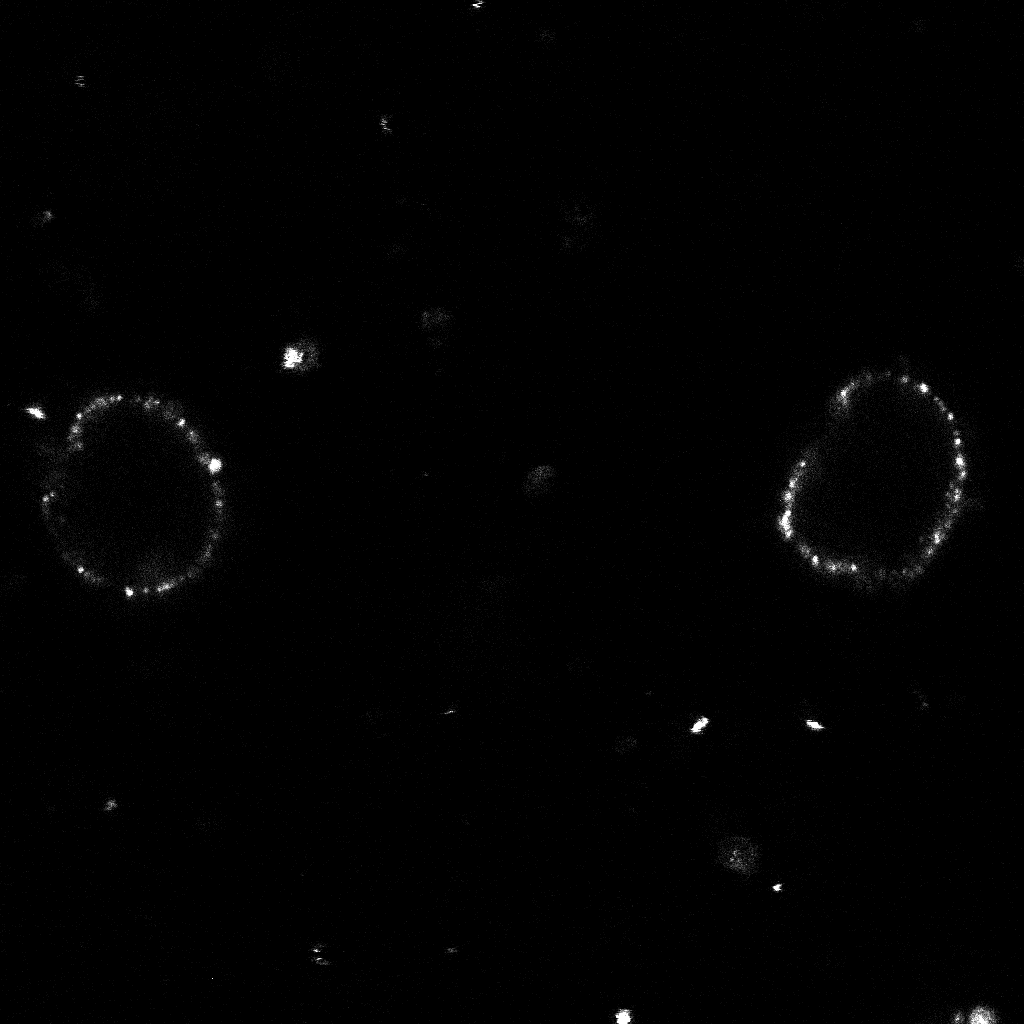

Supplement: Supplementary file 8 — Source data Fig. 1 [file 44319_2025_567_MOESM8_ESM.zip › Fig1/1D/Counted_nuclei/Mechanical/Nuc_1+2/Nuc_1+2_z22_RAW_ch00.tif]

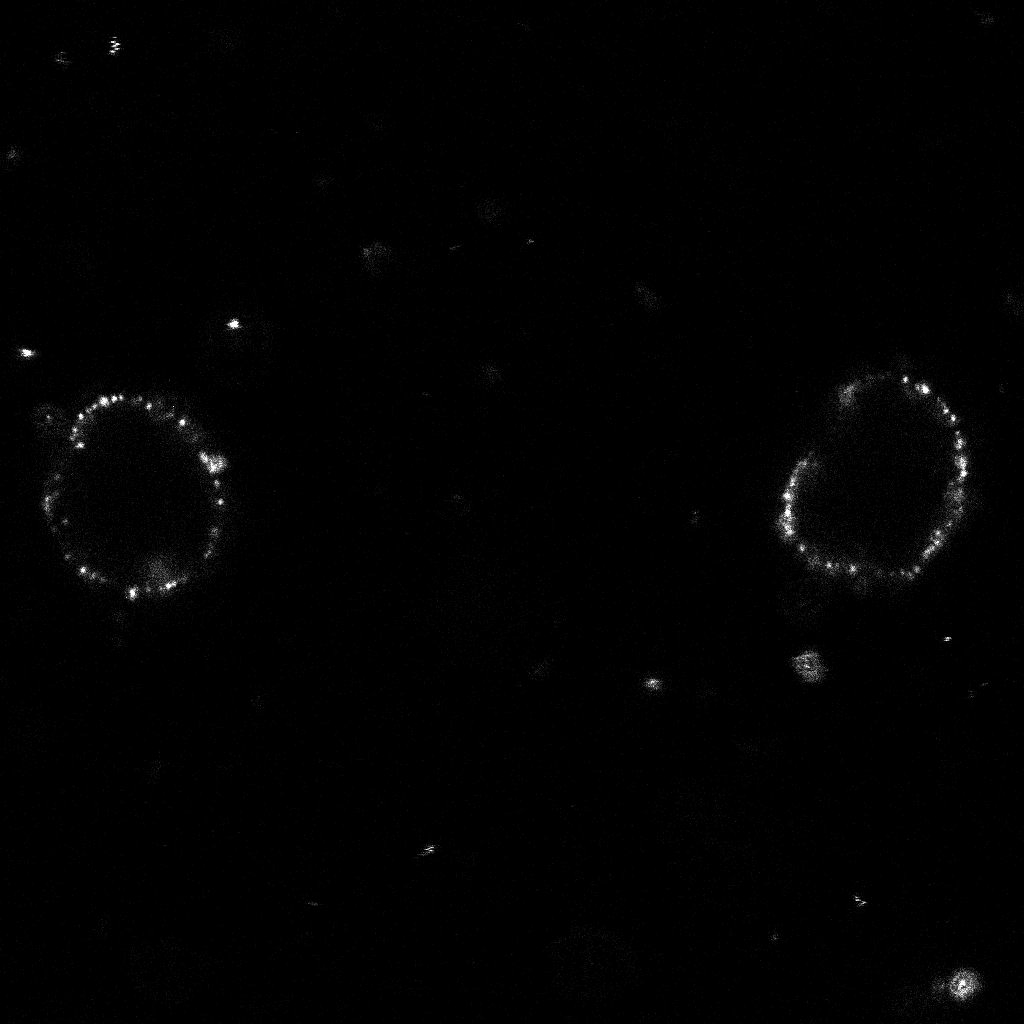

Supplement: Supplementary file 8 — Source data Fig. 1 [file 44319_2025_567_MOESM8_ESM.zip › Fig1/1D/Counted_nuclei/Mechanical/Nuc_1+2/Nuc_1+2_z23_RAW_ch00.tif]

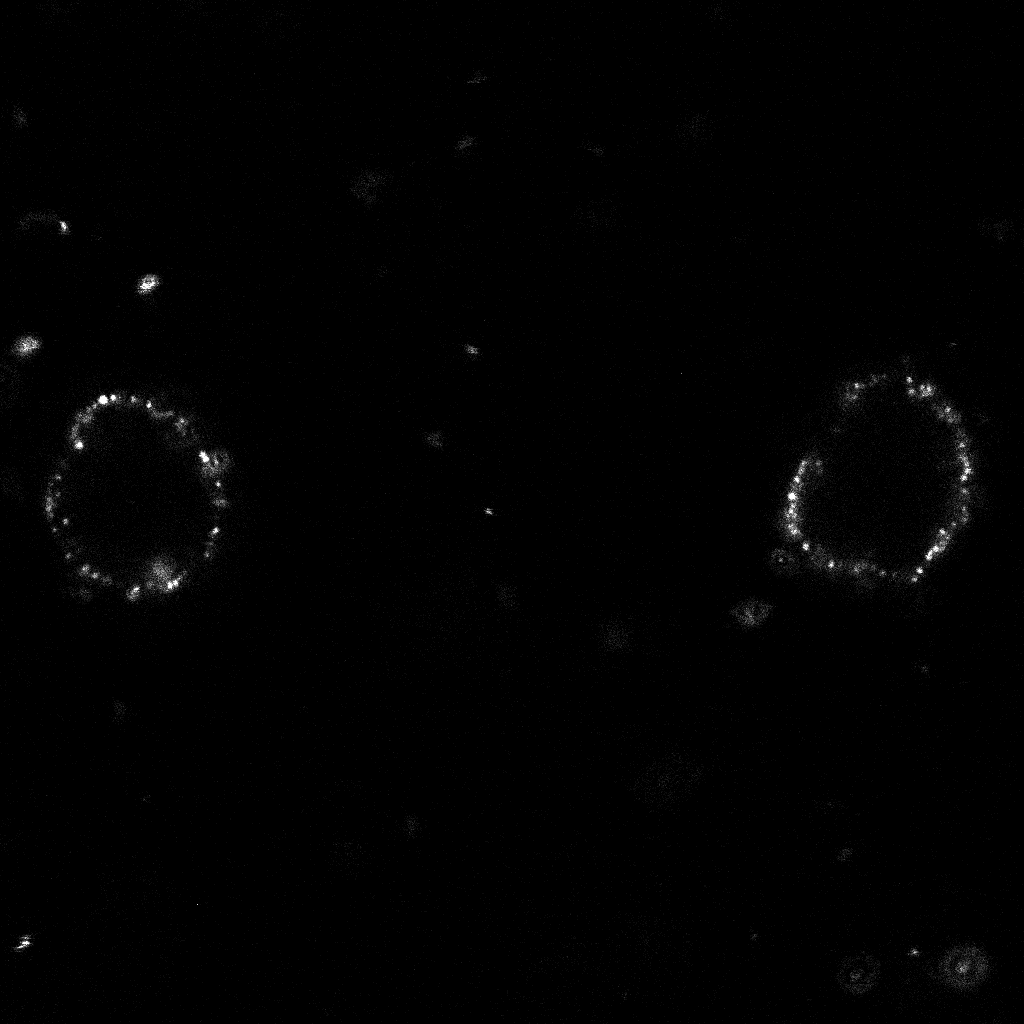

Supplement: Supplementary file 8 — Source data Fig. 1 [file 44319_2025_567_MOESM8_ESM.zip › Fig1/1D/Counted_nuclei/Mechanical/Nuc_1+2/Nuc_1+2_z24_RAW_ch00.tif]

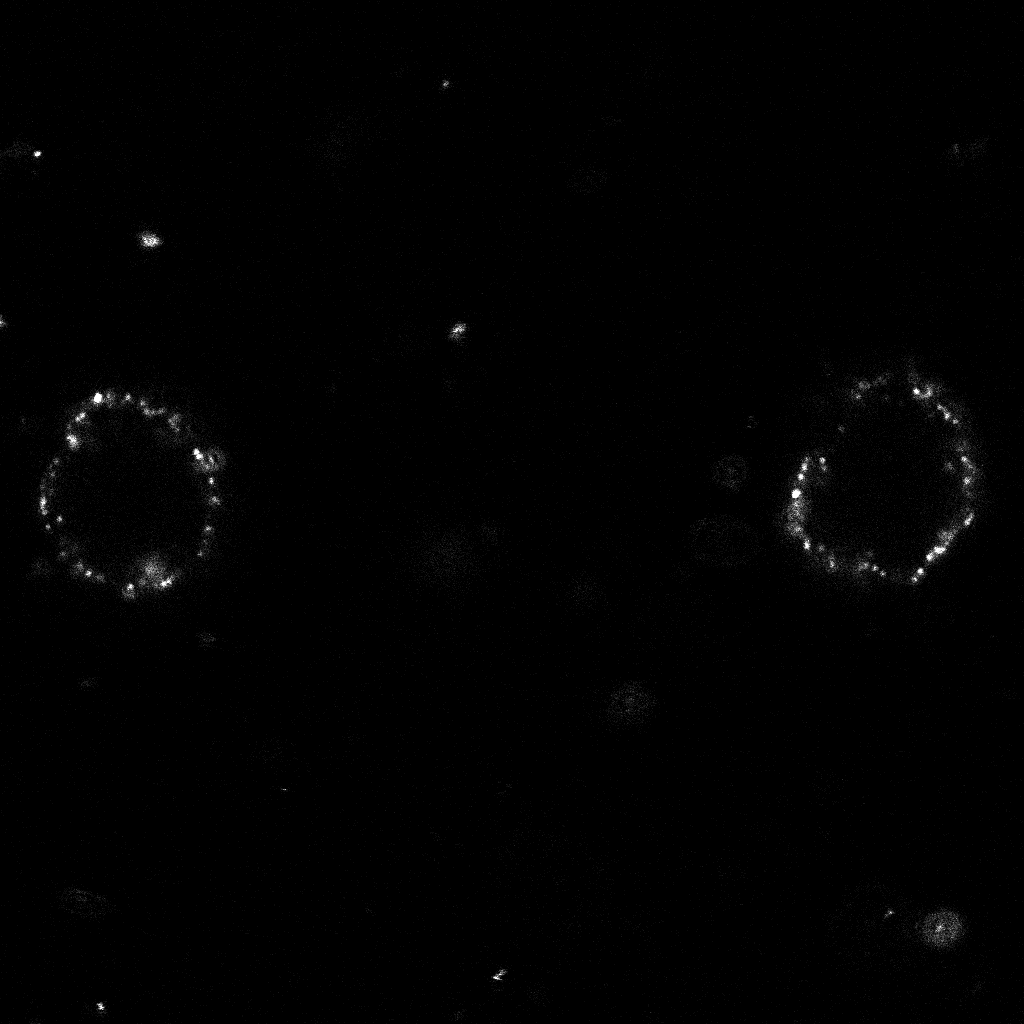

Supplement: Supplementary file 8 — Source data Fig. 1 [file 44319_2025_567_MOESM8_ESM.zip › Fig1/1D/Counted_nuclei/Mechanical/Nuc_1+2/Nuc_1+2_z25_RAW_ch00.tif]

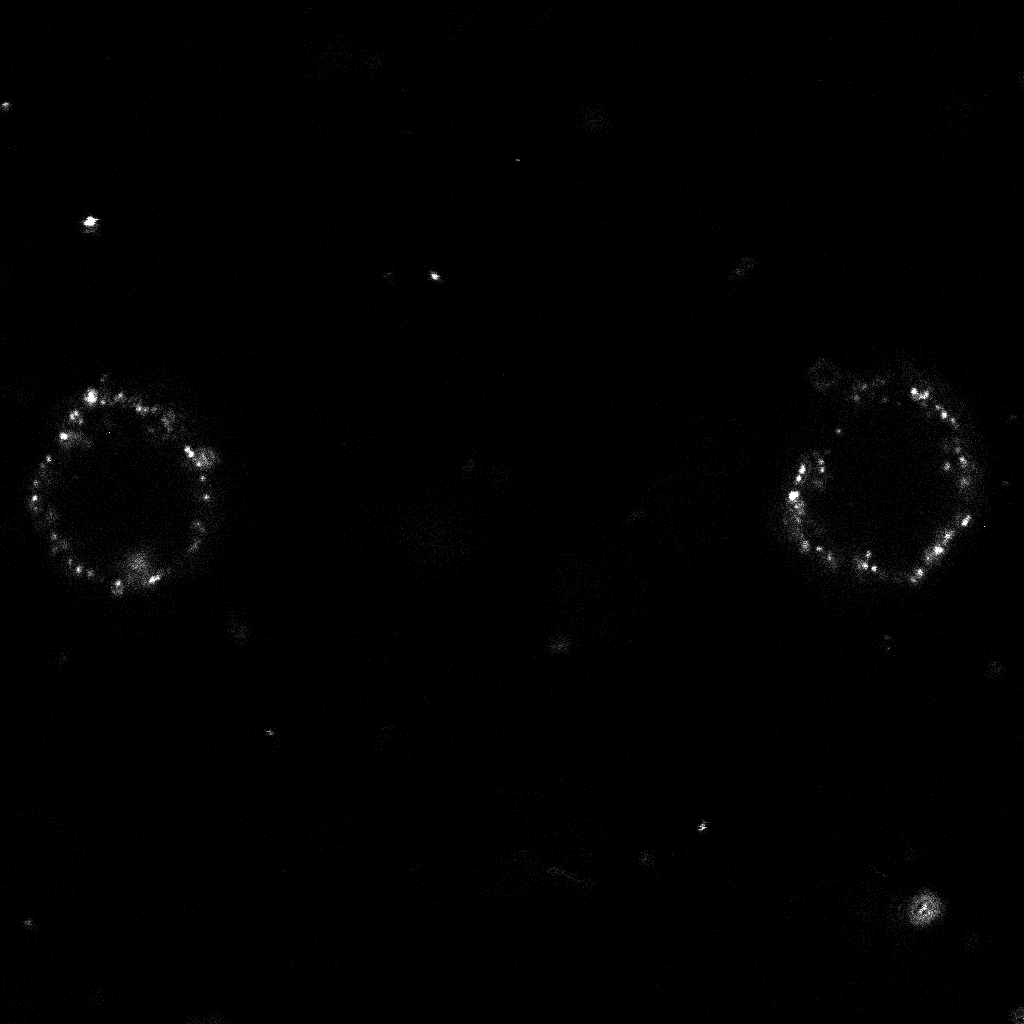

Supplement: Supplementary file 8 — Source data Fig. 1 [file 44319_2025_567_MOESM8_ESM.zip › Fig1/1D/Counted_nuclei/Mechanical/Nuc_1+2/Nuc_1+2_z26_RAW_ch00.tif]

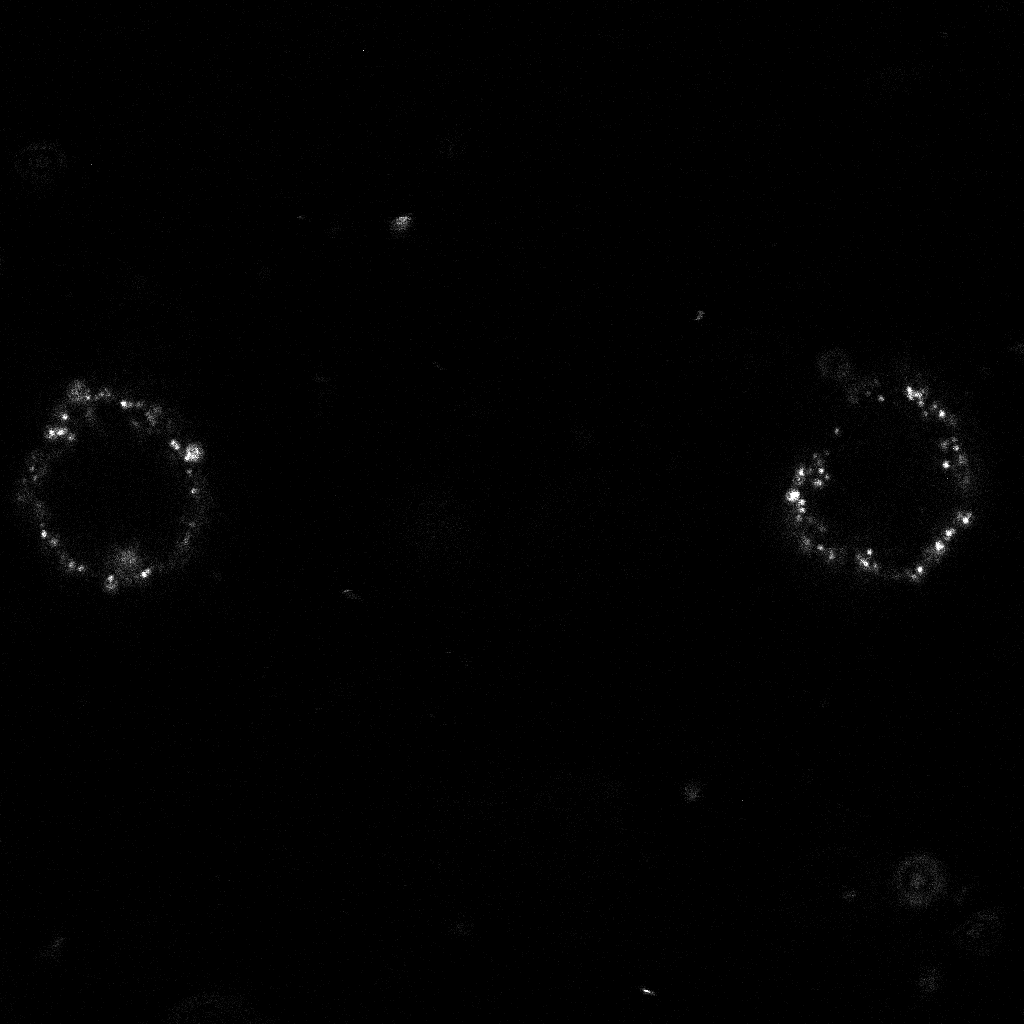

Supplement: Supplementary file 8 — Source data Fig. 1 [file 44319_2025_567_MOESM8_ESM.zip › Fig1/1D/Counted_nuclei/Mechanical/Nuc_1+2/Nuc_1+2_z27_RAW_ch00.tif]

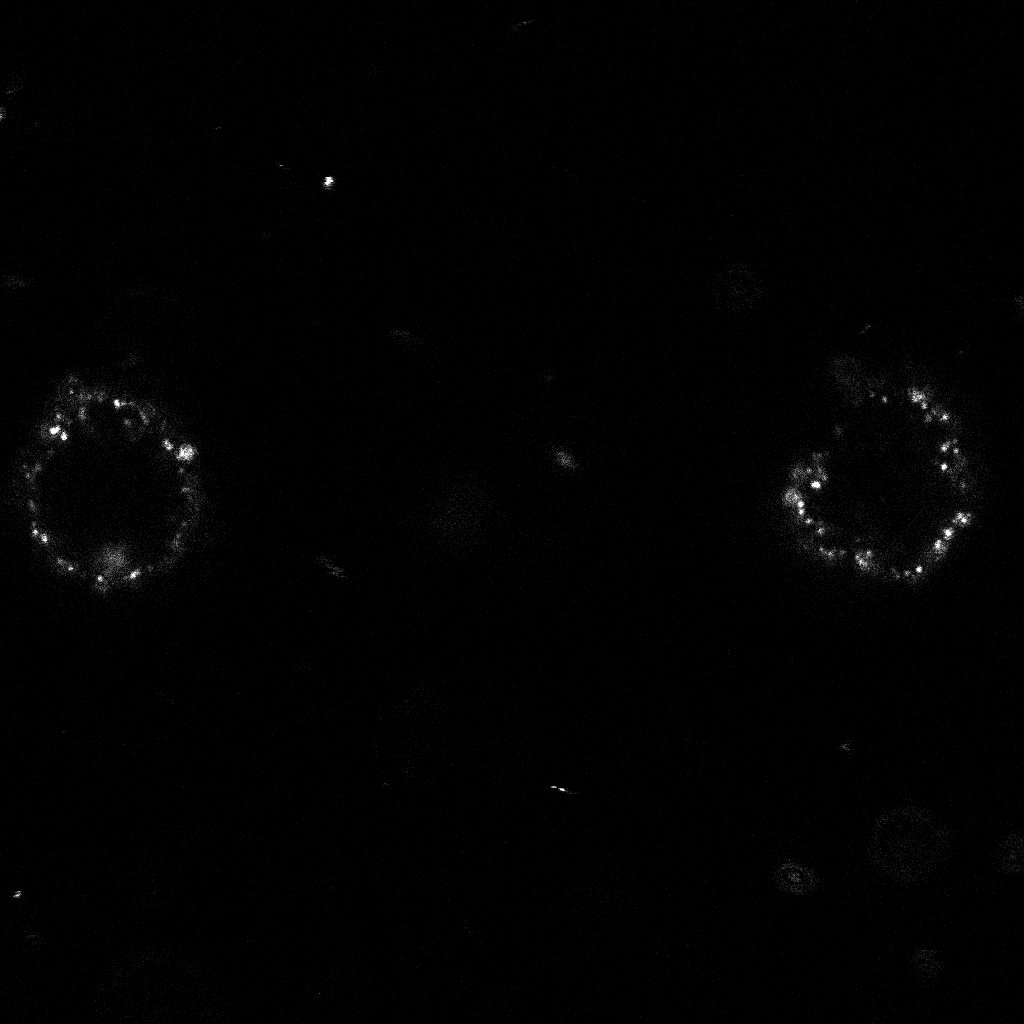

Supplement: Supplementary file 8 — Source data Fig. 1 [file 44319_2025_567_MOESM8_ESM.zip › Fig1/1D/Counted_nuclei/Mechanical/Nuc_1+2/Nuc_1+2_z28_RAW_ch00.tif]

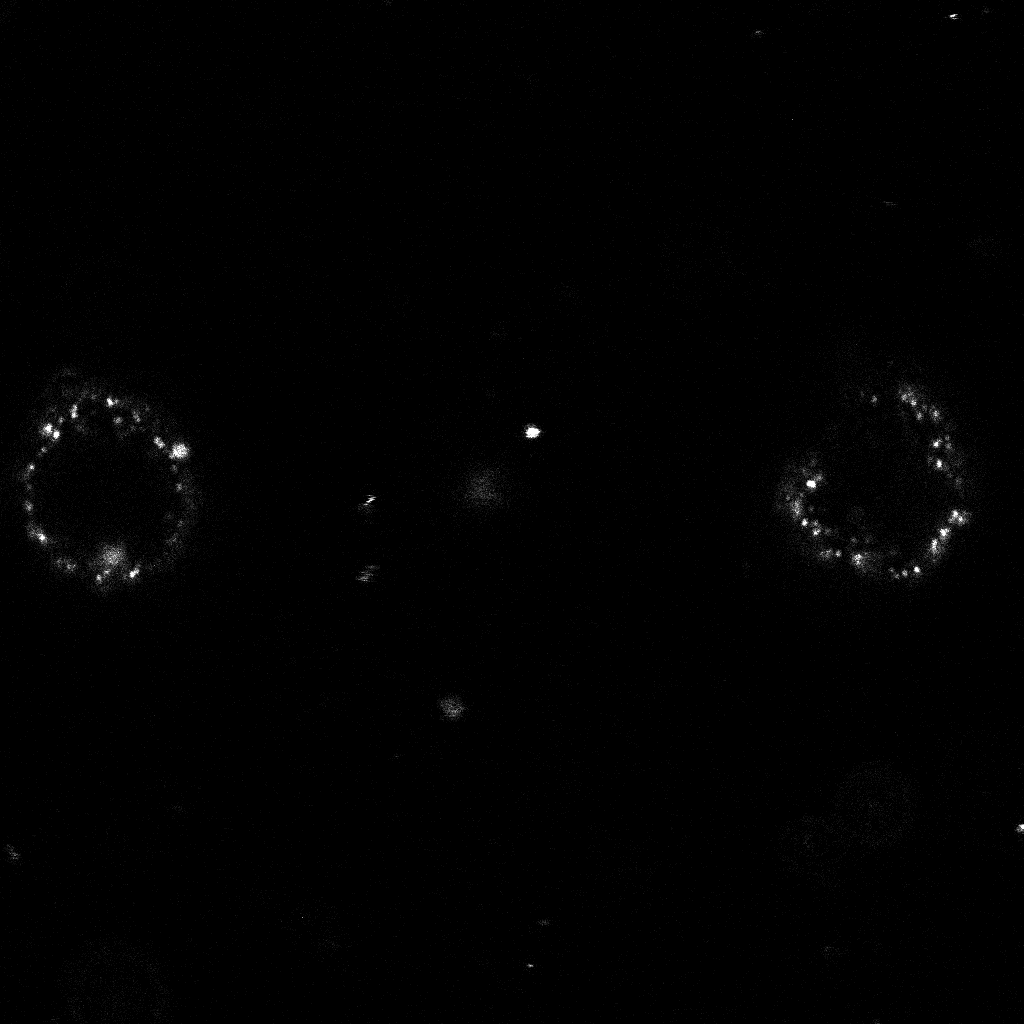

Supplement: Supplementary file 8 — Source data Fig. 1 [file 44319_2025_567_MOESM8_ESM.zip › Fig1/1D/Counted_nuclei/Mechanical/Nuc_1+2/Nuc_1+2_z29_RAW_ch00.tif]

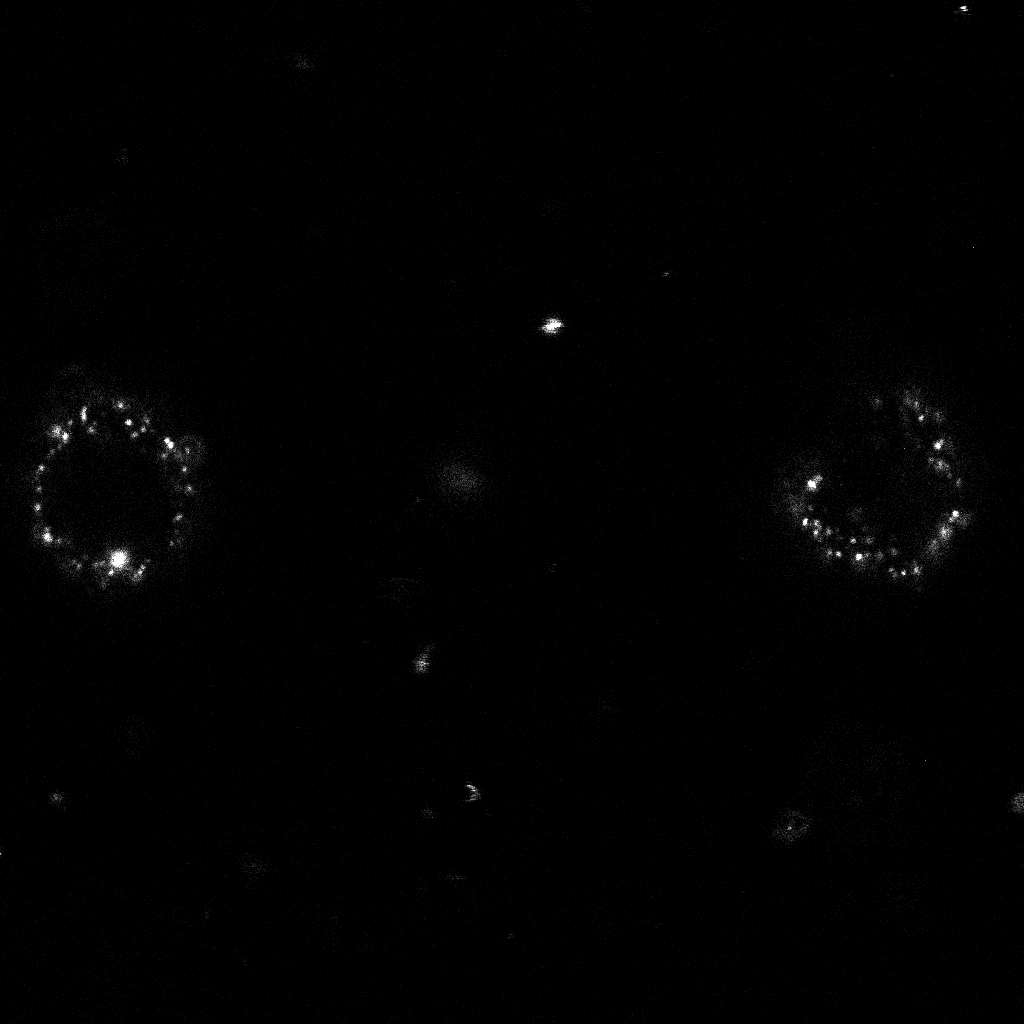

Supplement: Supplementary file 8 — Source data Fig. 1 [file 44319_2025_567_MOESM8_ESM.zip › Fig1/1D/Counted_nuclei/Mechanical/Nuc_1+2/Nuc_1+2_z30_RAW_ch00.tif]

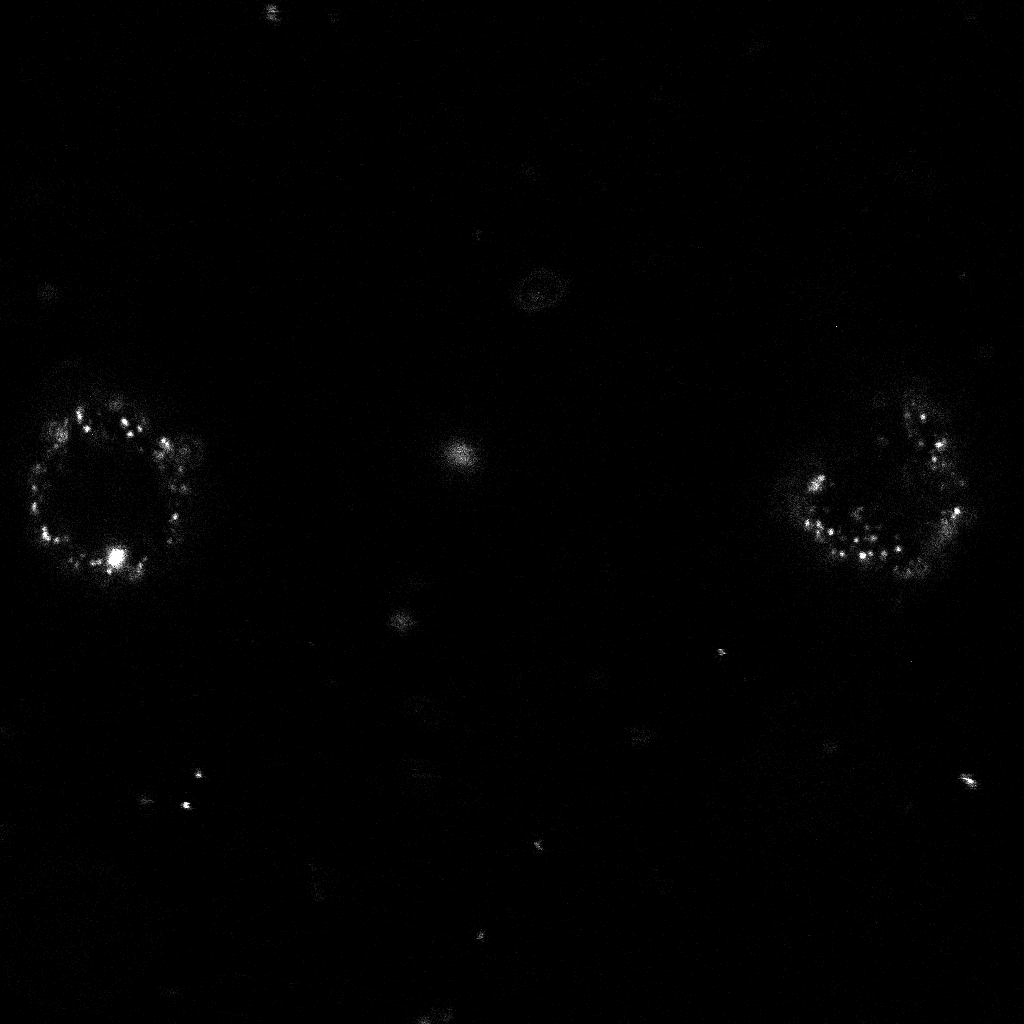

Supplement: Supplementary file 8 — Source data Fig. 1 [file 44319_2025_567_MOESM8_ESM.zip › Fig1/1D/Counted_nuclei/Mechanical/Nuc_1+2/Nuc_1+2_z31_RAW_ch00.tif]

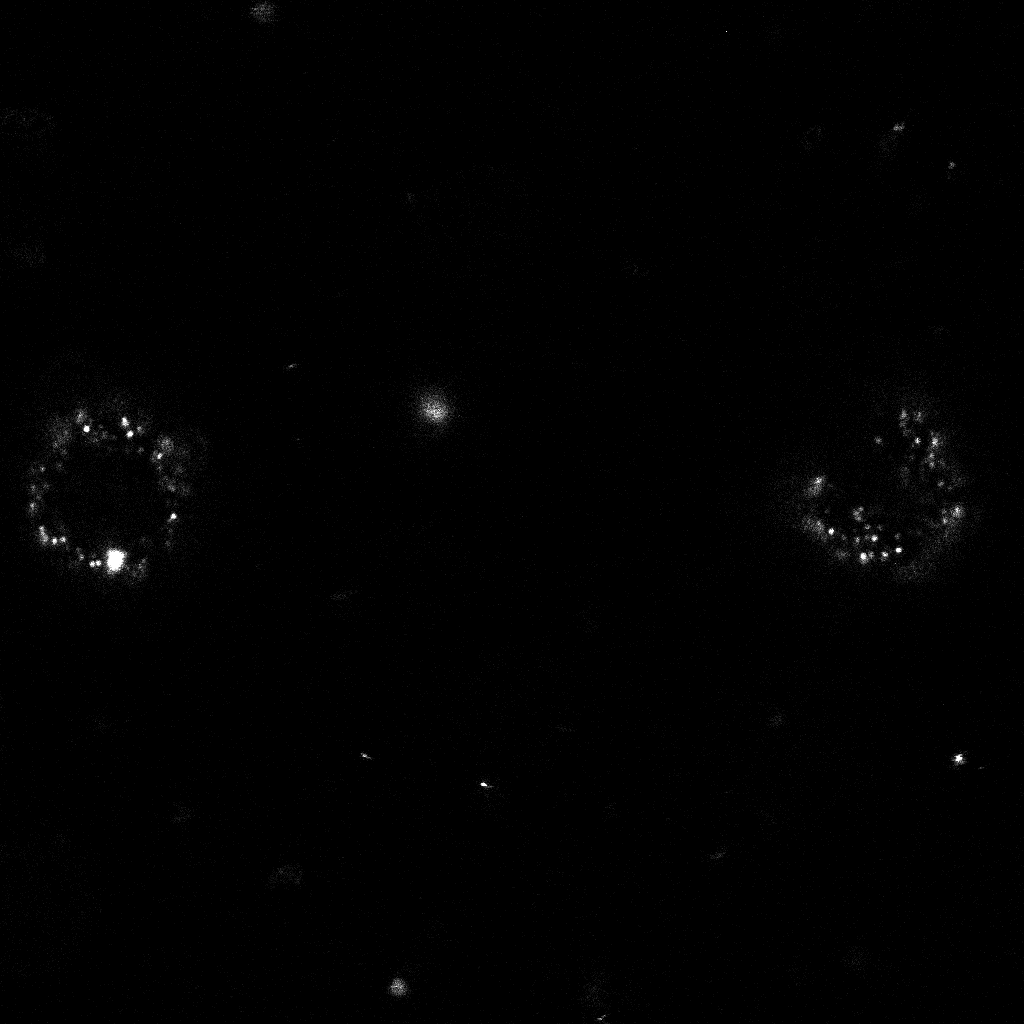

Supplement: Supplementary file 8 — Source data Fig. 1 [file 44319_2025_567_MOESM8_ESM.zip › Fig1/1D/Counted_nuclei/Mechanical/Nuc_1+2/Nuc_1+2_z32_RAW_ch00.tif]

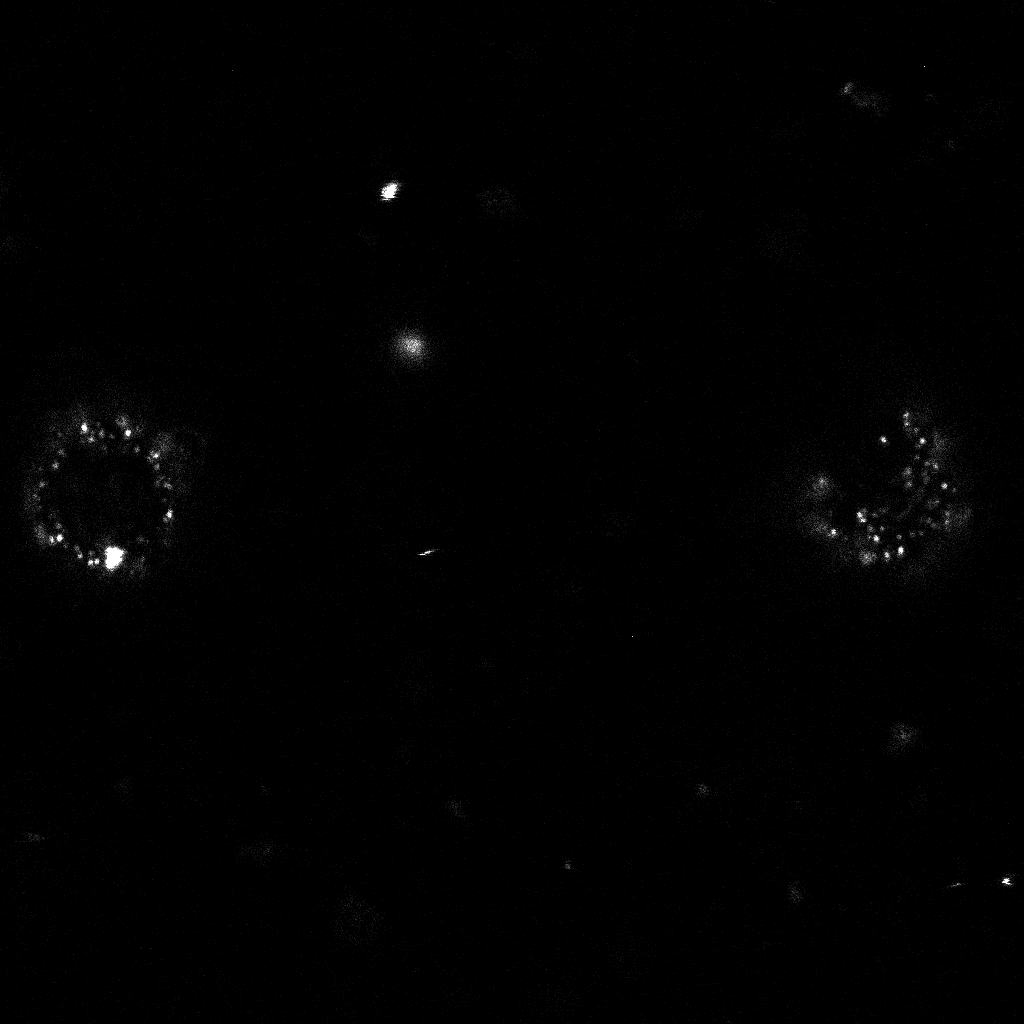

Supplement: Supplementary file 8 — Source data Fig. 1 [file 44319_2025_567_MOESM8_ESM.zip › Fig1/1D/Counted_nuclei/Mechanical/Nuc_1+2/Nuc_1+2_z33_RAW_ch00.tif]

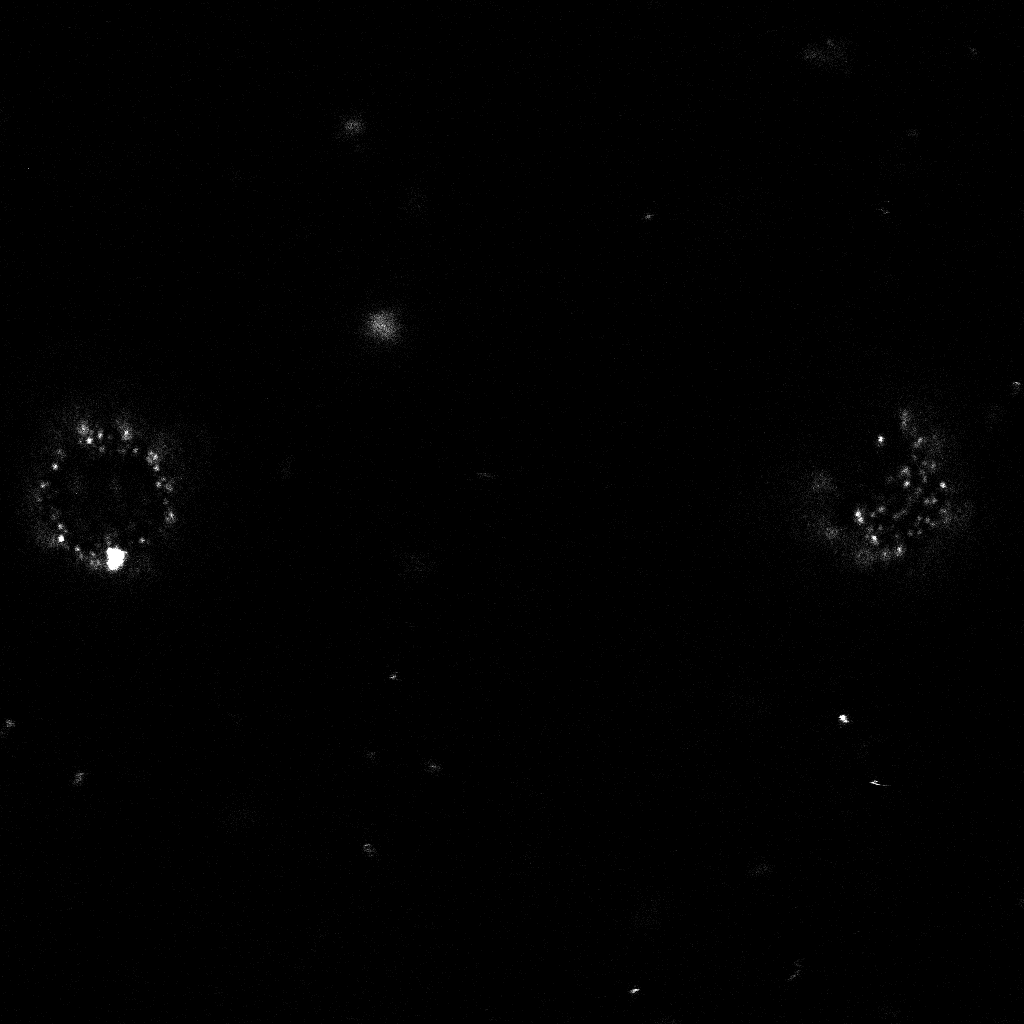

Supplement: Supplementary file 8 — Source data Fig. 1 [file 44319_2025_567_MOESM8_ESM.zip › Fig1/1D/Counted_nuclei/Mechanical/Nuc_1+2/Nuc_1+2_z34_RAW_ch00.tif]

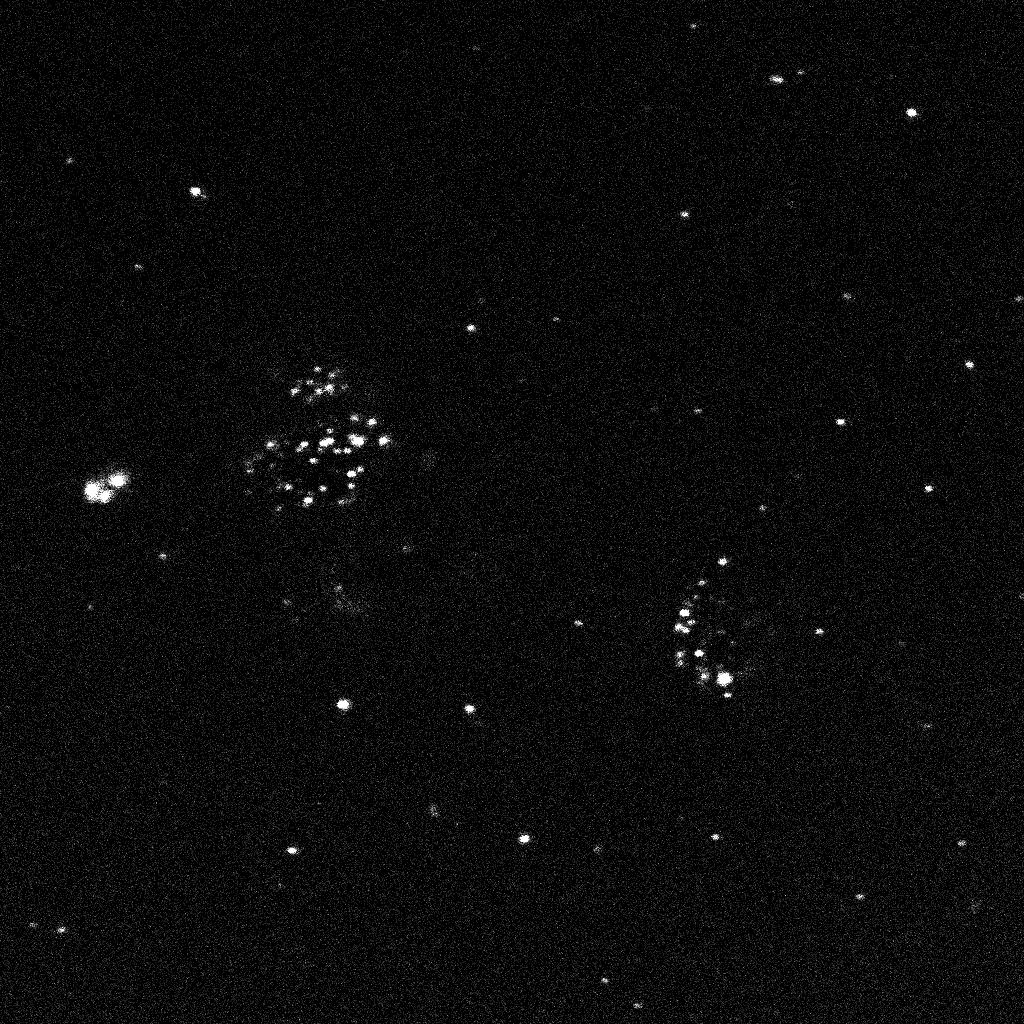

Supplement: Supplementary file 8 — Source data Fig. 1 [file 44319_2025_567_MOESM8_ESM.zip › Fig1/1D/Counted_nuclei/Mechanical/Nuc_10+11+12+13+14/Nuc_10+11+12+13+14_z04_RAW_ch00.tif]

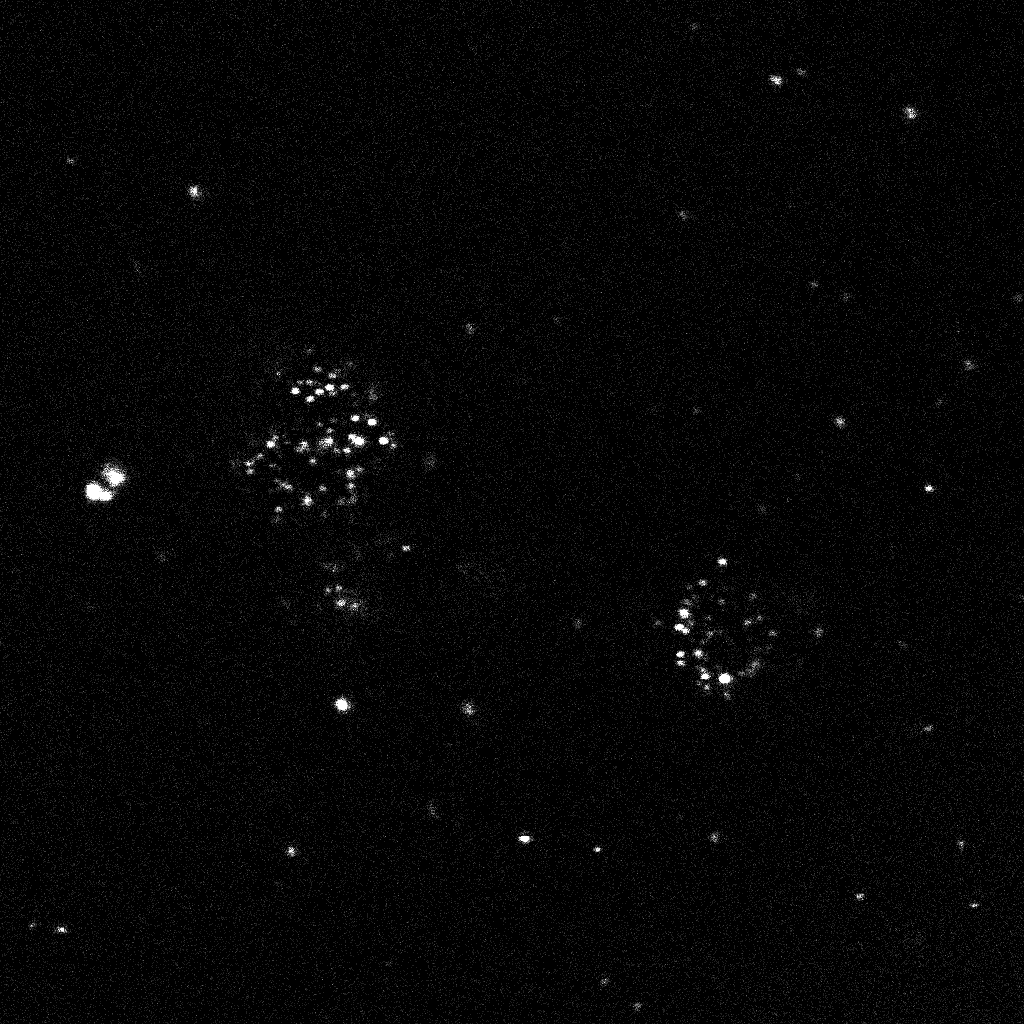

Supplement: Supplementary file 8 — Source data Fig. 1 [file 44319_2025_567_MOESM8_ESM.zip › Fig1/1D/Counted_nuclei/Mechanical/Nuc_10+11+12+13+14/Nuc_10+11+12+13+14_z05_RAW_ch00.tif]

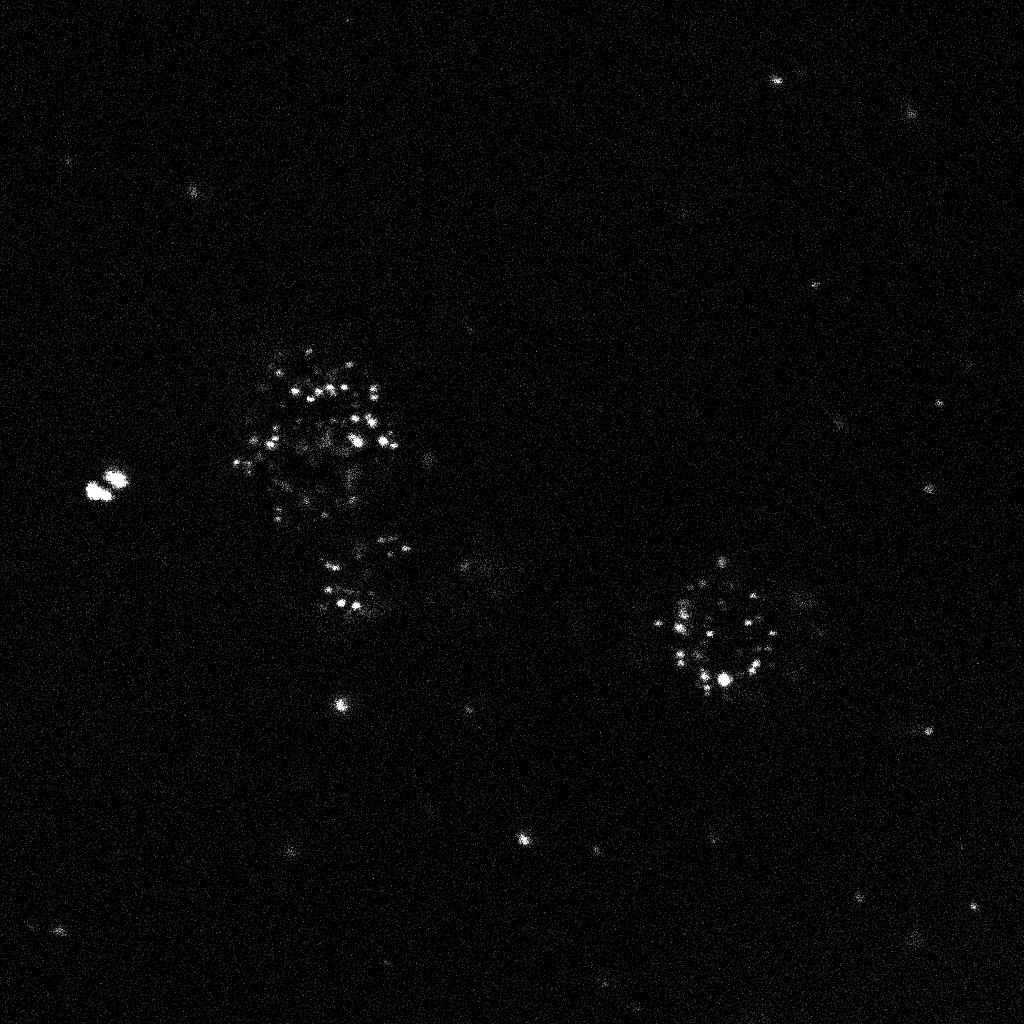

Supplement: Supplementary file 8 — Source data Fig. 1 [file 44319_2025_567_MOESM8_ESM.zip › Fig1/1D/Counted_nuclei/Mechanical/Nuc_10+11+12+13+14/Nuc_10+11+12+13+14_z06_RAW_ch00.tif]

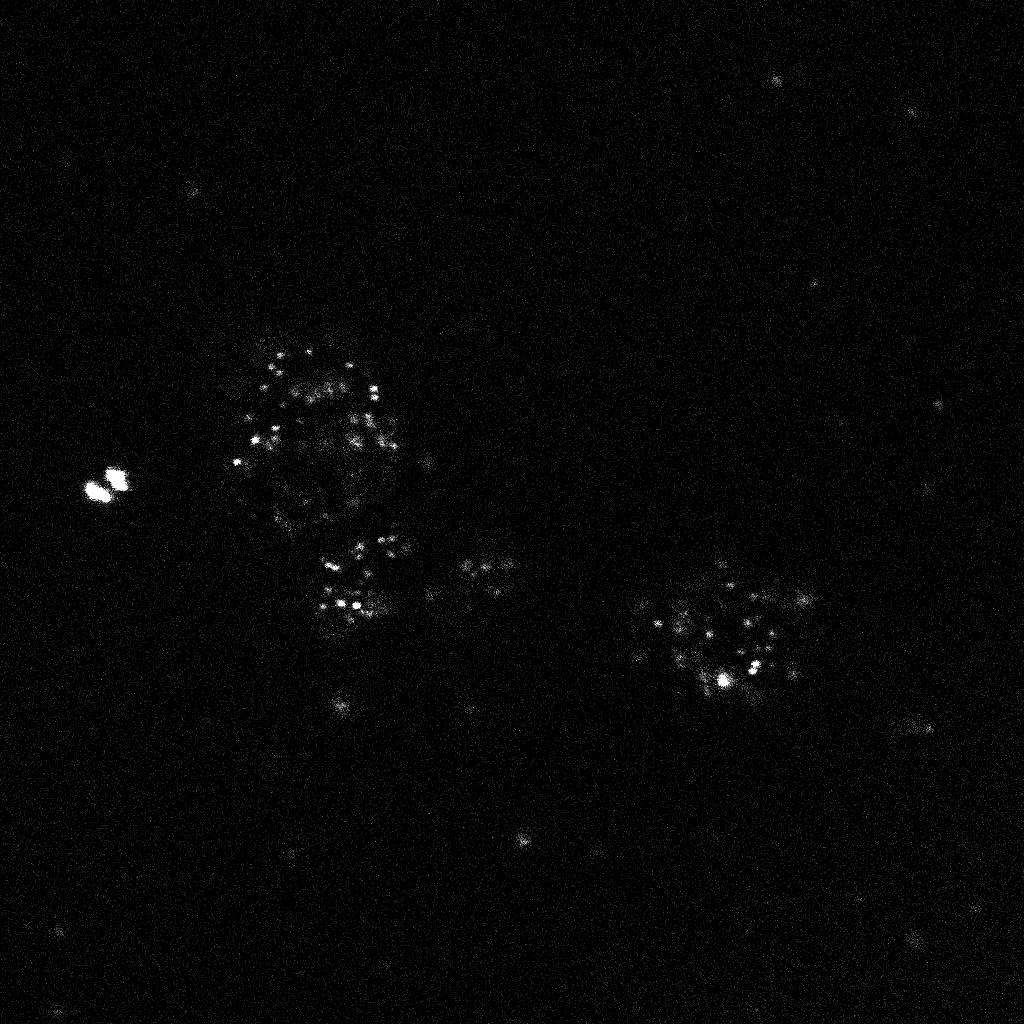

Supplement: Supplementary file 8 — Source data Fig. 1 [file 44319_2025_567_MOESM8_ESM.zip › Fig1/1D/Counted_nuclei/Mechanical/Nuc_10+11+12+13+14/Nuc_10+11+12+13+14_z07_RAW_ch00.tif]

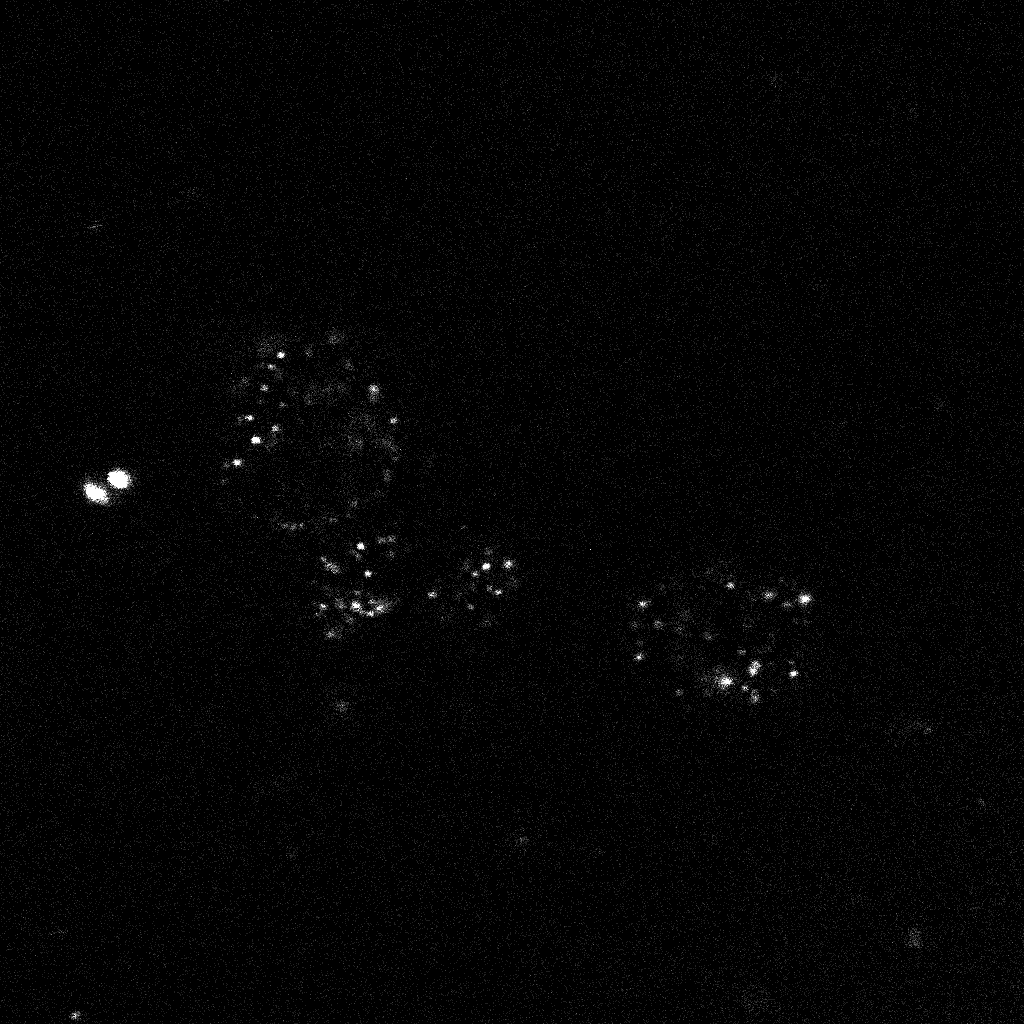

Supplement: Supplementary file 8 — Source data Fig. 1 [file 44319_2025_567_MOESM8_ESM.zip › Fig1/1D/Counted_nuclei/Mechanical/Nuc_10+11+12+13+14/Nuc_10+11+12+13+14_z08_RAW_ch00.tif]

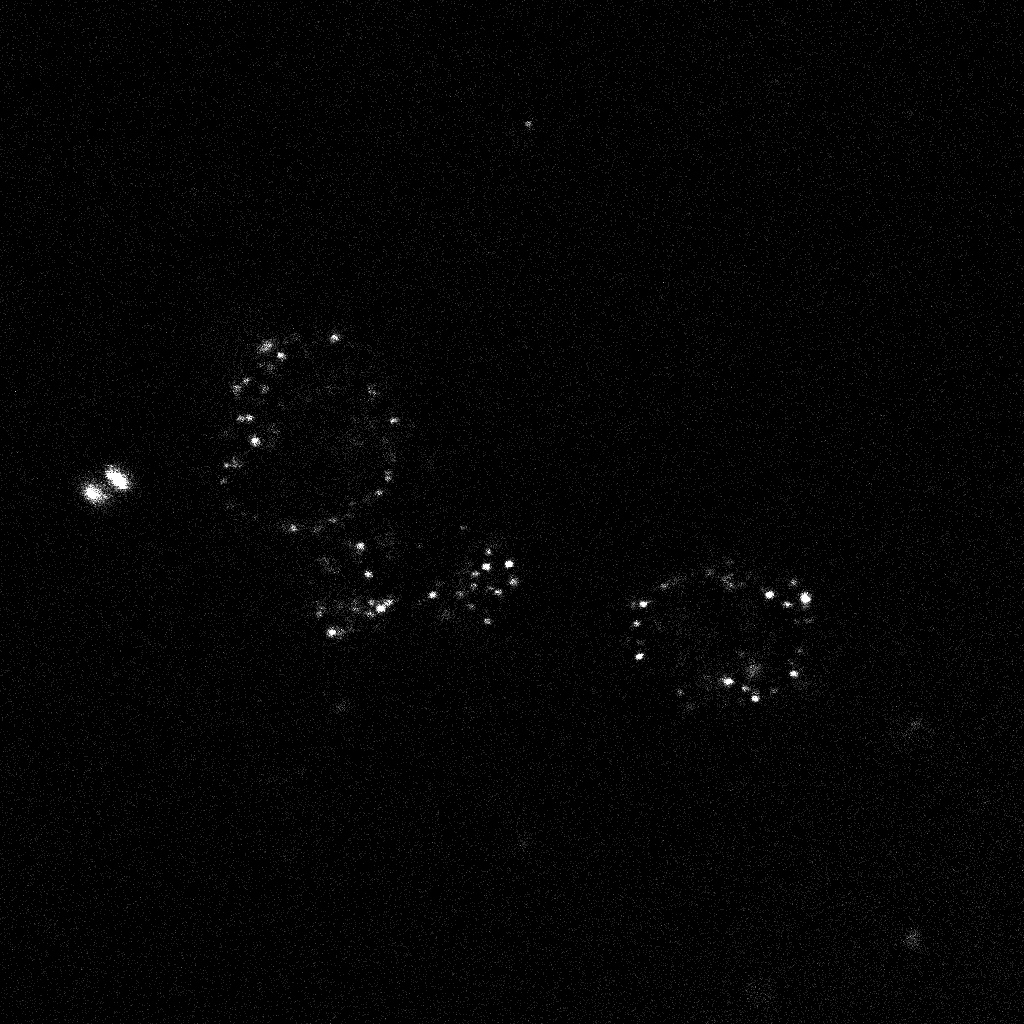

Supplement: Supplementary file 8 — Source data Fig. 1 [file 44319_2025_567_MOESM8_ESM.zip › Fig1/1D/Counted_nuclei/Mechanical/Nuc_10+11+12+13+14/Nuc_10+11+12+13+14_z09_RAW_ch00.tif]

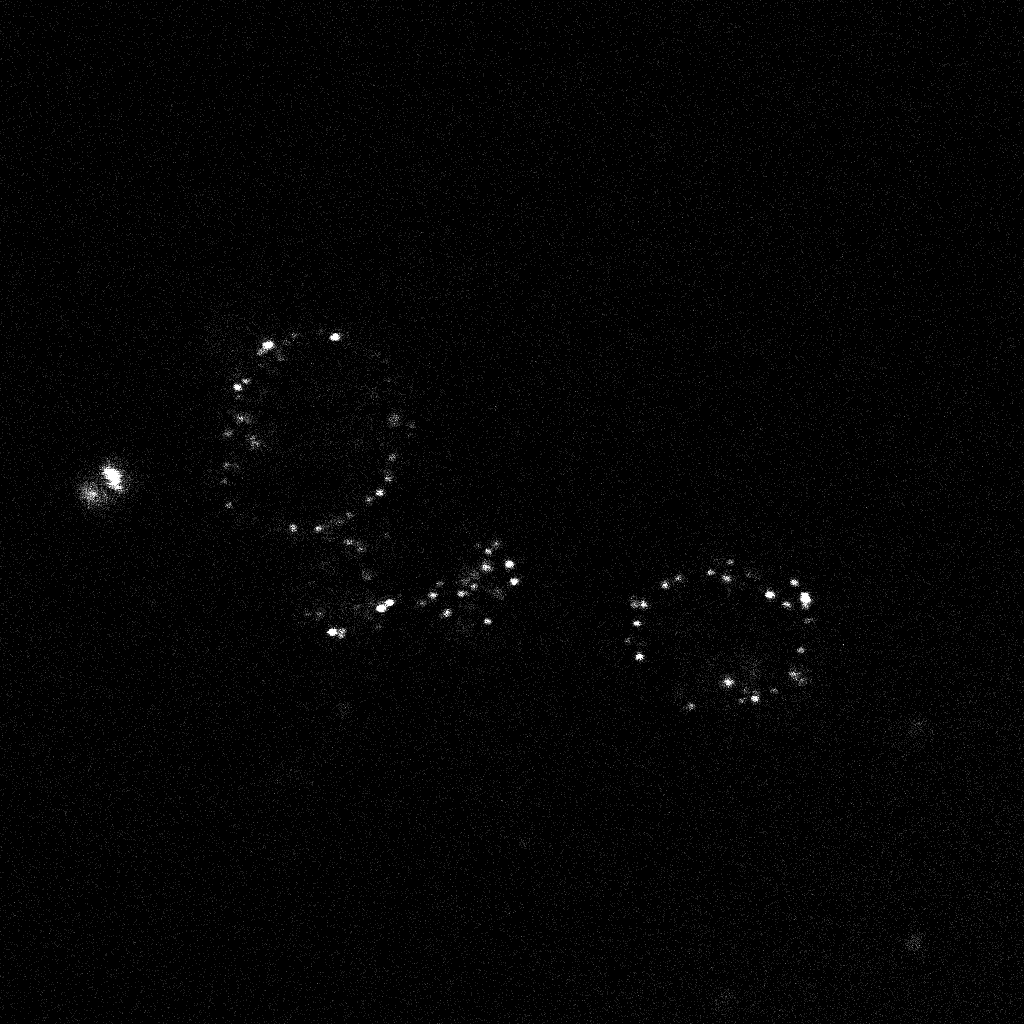

Supplement: Supplementary file 8 — Source data Fig. 1 [file 44319_2025_567_MOESM8_ESM.zip › Fig1/1D/Counted_nuclei/Mechanical/Nuc_10+11+12+13+14/Nuc_10+11+12+13+14_z10_RAW_ch00.tif]

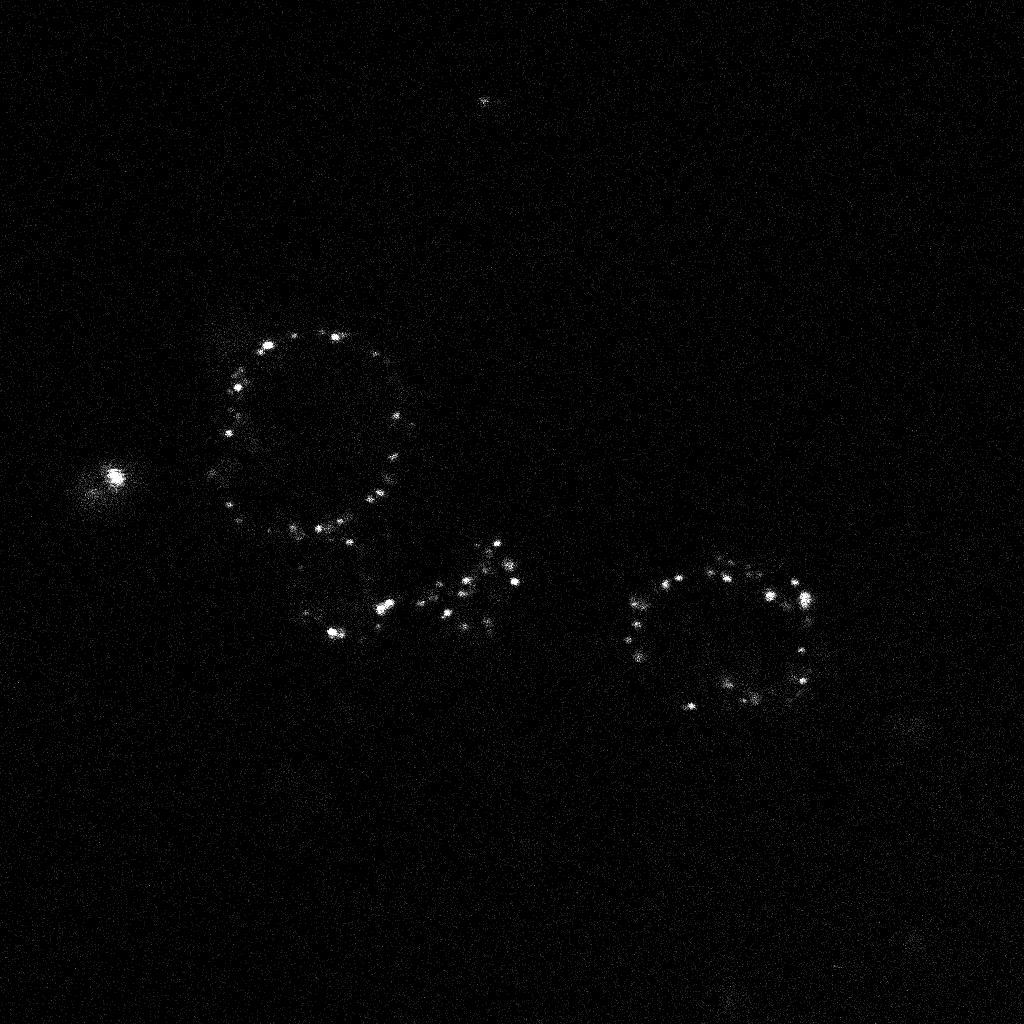

Supplement: Supplementary file 8 — Source data Fig. 1 [file 44319_2025_567_MOESM8_ESM.zip › Fig1/1D/Counted_nuclei/Mechanical/Nuc_10+11+12+13+14/Nuc_10+11+12+13+14_z11_RAW_ch00.tif]

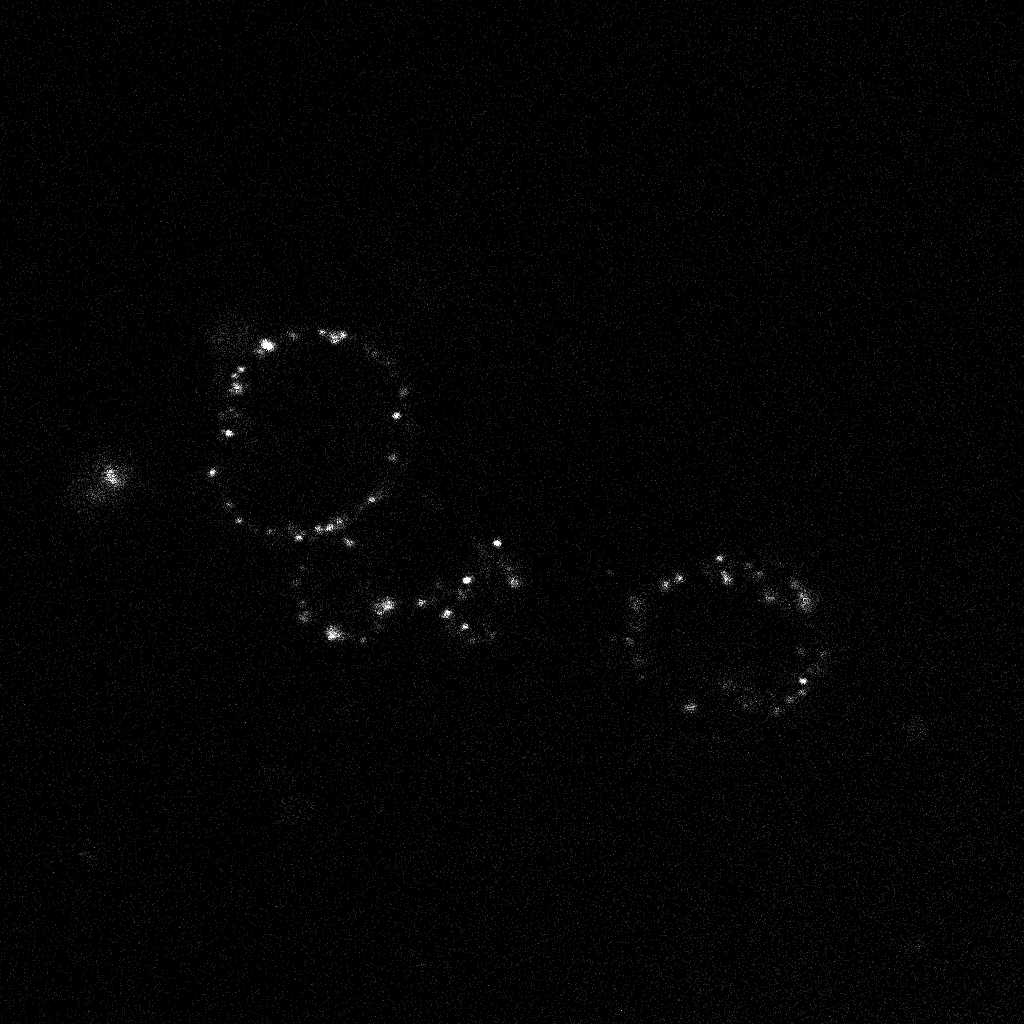

Supplement: Supplementary file 8 — Source data Fig. 1 [file 44319_2025_567_MOESM8_ESM.zip › Fig1/1D/Counted_nuclei/Mechanical/Nuc_10+11+12+13+14/Nuc_10+11+12+13+14_z12_RAW_ch00.tif]

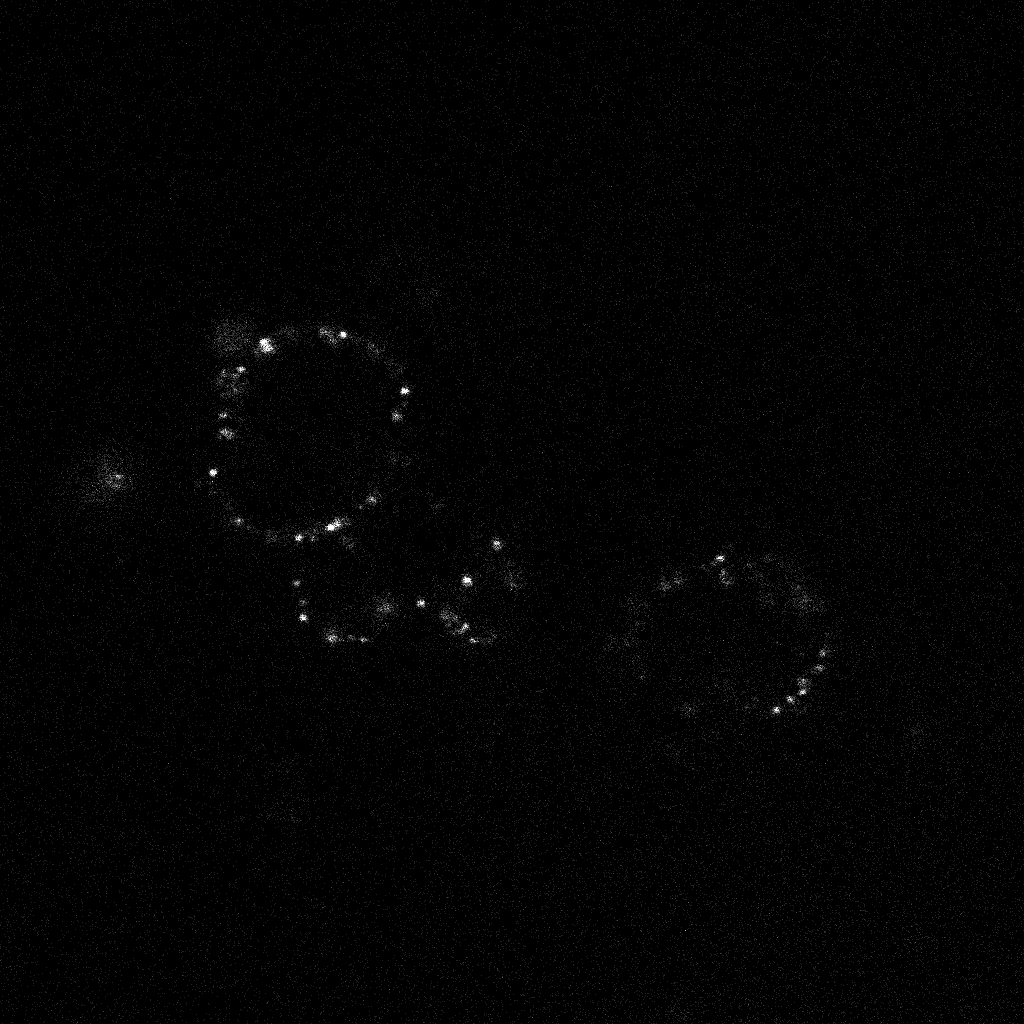

Supplement: Supplementary file 8 — Source data Fig. 1 [file 44319_2025_567_MOESM8_ESM.zip › Fig1/1D/Counted_nuclei/Mechanical/Nuc_10+11+12+13+14/Nuc_10+11+12+13+14_z13_RAW_ch00.tif]

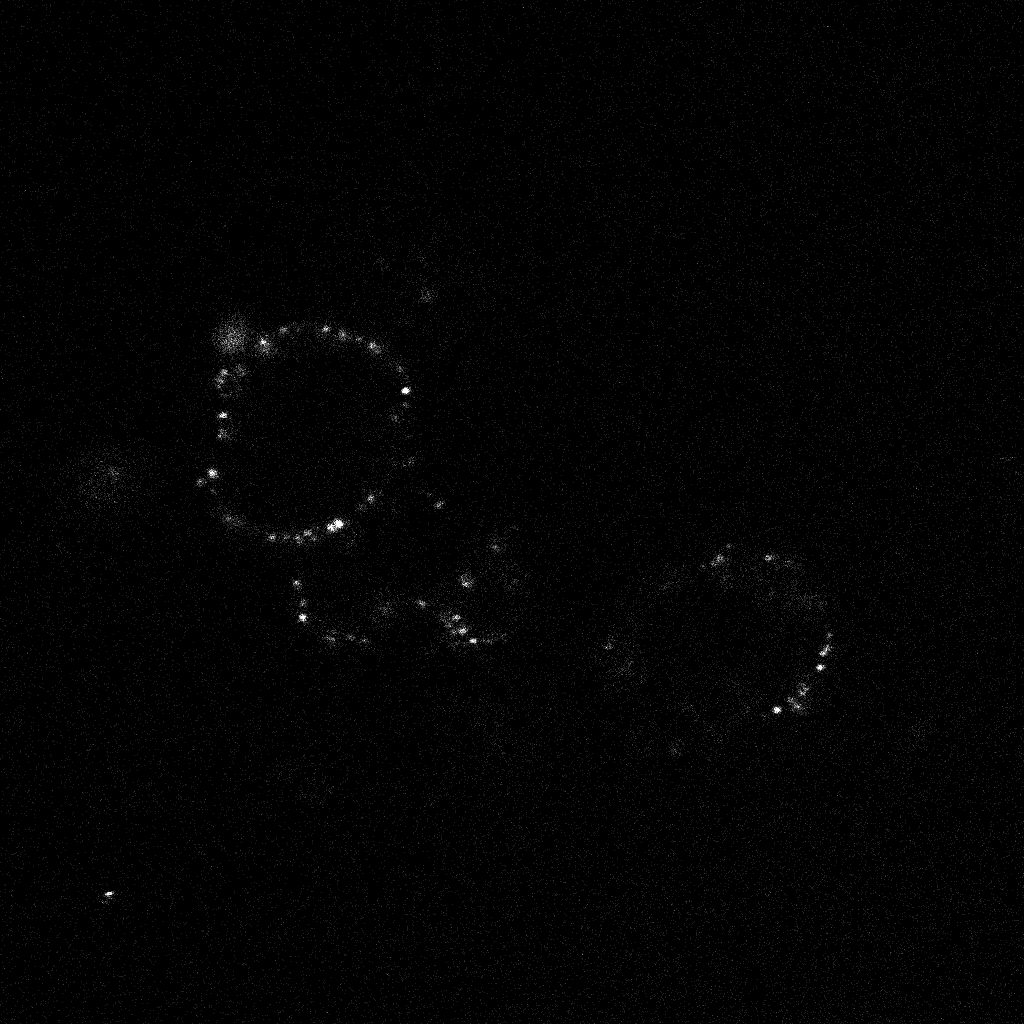

Supplement: Supplementary file 8 — Source data Fig. 1 [file 44319_2025_567_MOESM8_ESM.zip › Fig1/1D/Counted_nuclei/Mechanical/Nuc_10+11+12+13+14/Nuc_10+11+12+13+14_z14_RAW_ch00.tif]

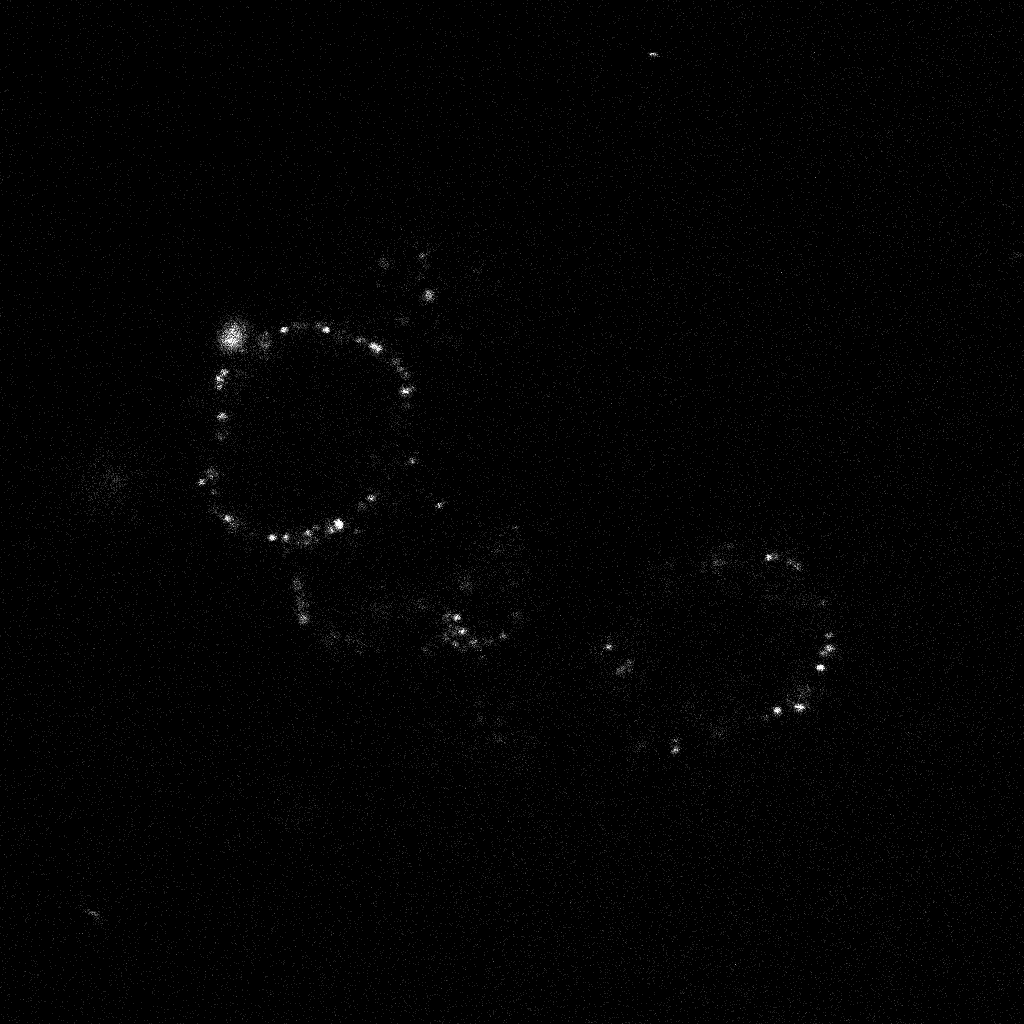

Supplement: Supplementary file 8 — Source data Fig. 1 [file 44319_2025_567_MOESM8_ESM.zip › Fig1/1D/Counted_nuclei/Mechanical/Nuc_10+11+12+13+14/Nuc_10+11+12+13+14_z15_RAW_ch00.tif]

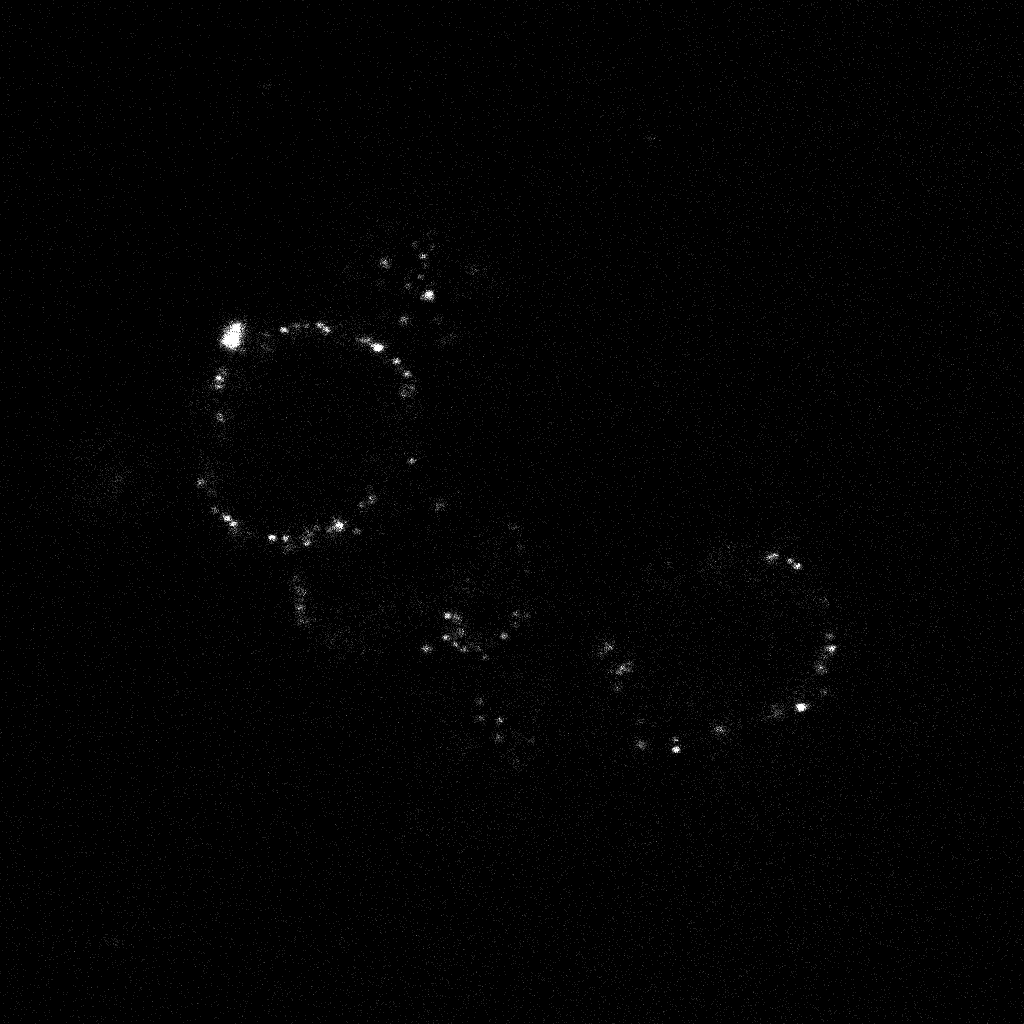

Supplement: Supplementary file 8 — Source data Fig. 1 [file 44319_2025_567_MOESM8_ESM.zip › Fig1/1D/Counted_nuclei/Mechanical/Nuc_10+11+12+13+14/Nuc_10+11+12+13+14_z16_RAW_ch00.tif]

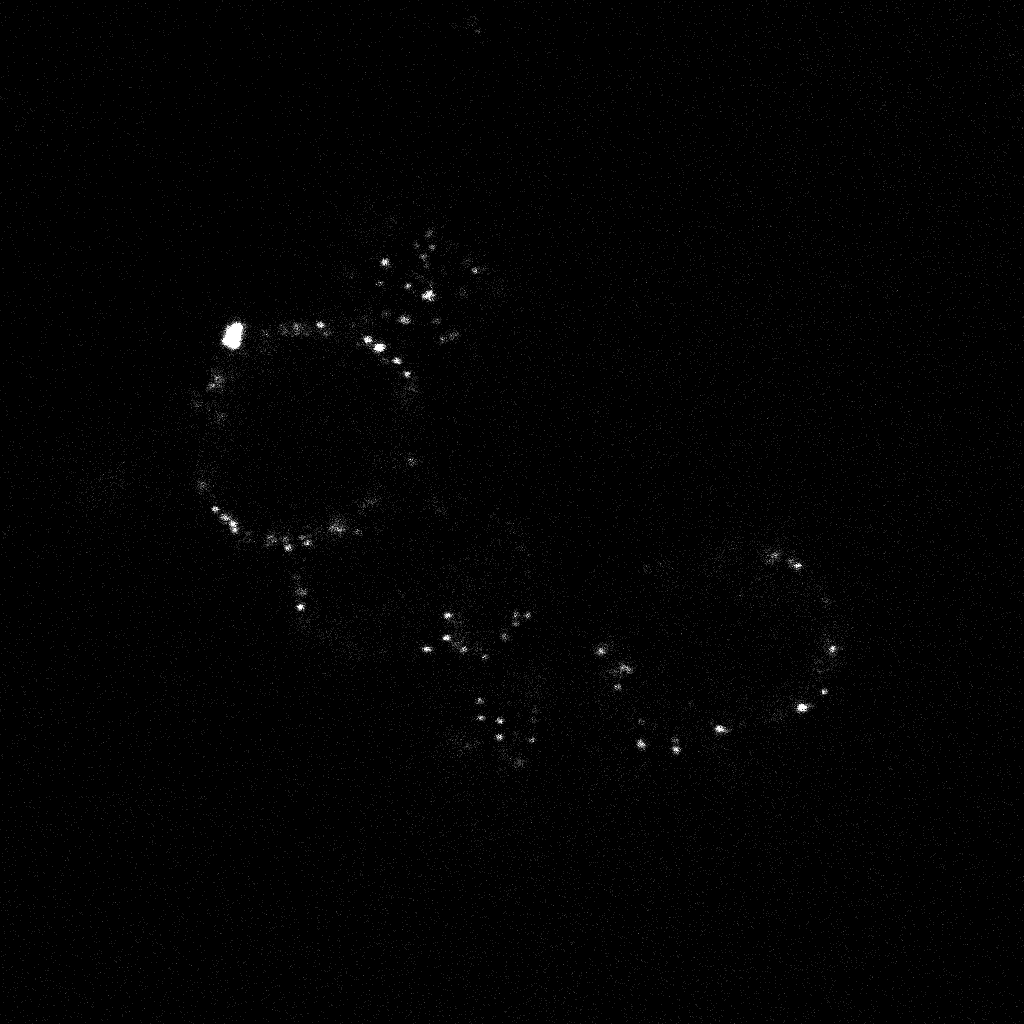

Supplement: Supplementary file 8 — Source data Fig. 1 [file 44319_2025_567_MOESM8_ESM.zip › Fig1/1D/Counted_nuclei/Mechanical/Nuc_10+11+12+13+14/Nuc_10+11+12+13+14_z17_RAW_ch00.tif]

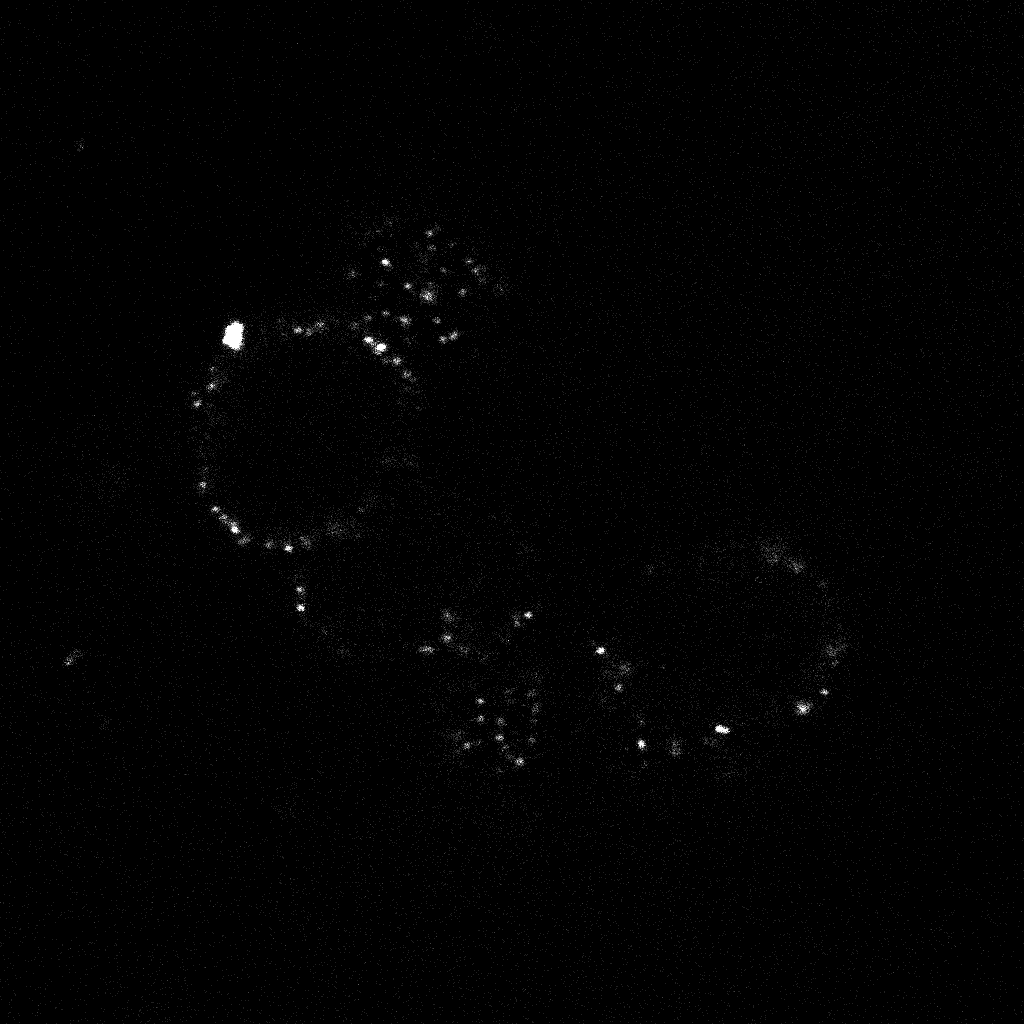

Supplement: Supplementary file 8 — Source data Fig. 1 [file 44319_2025_567_MOESM8_ESM.zip › Fig1/1D/Counted_nuclei/Mechanical/Nuc_10+11+12+13+14/Nuc_10+11+12+13+14_z18_RAW_ch00.tif]

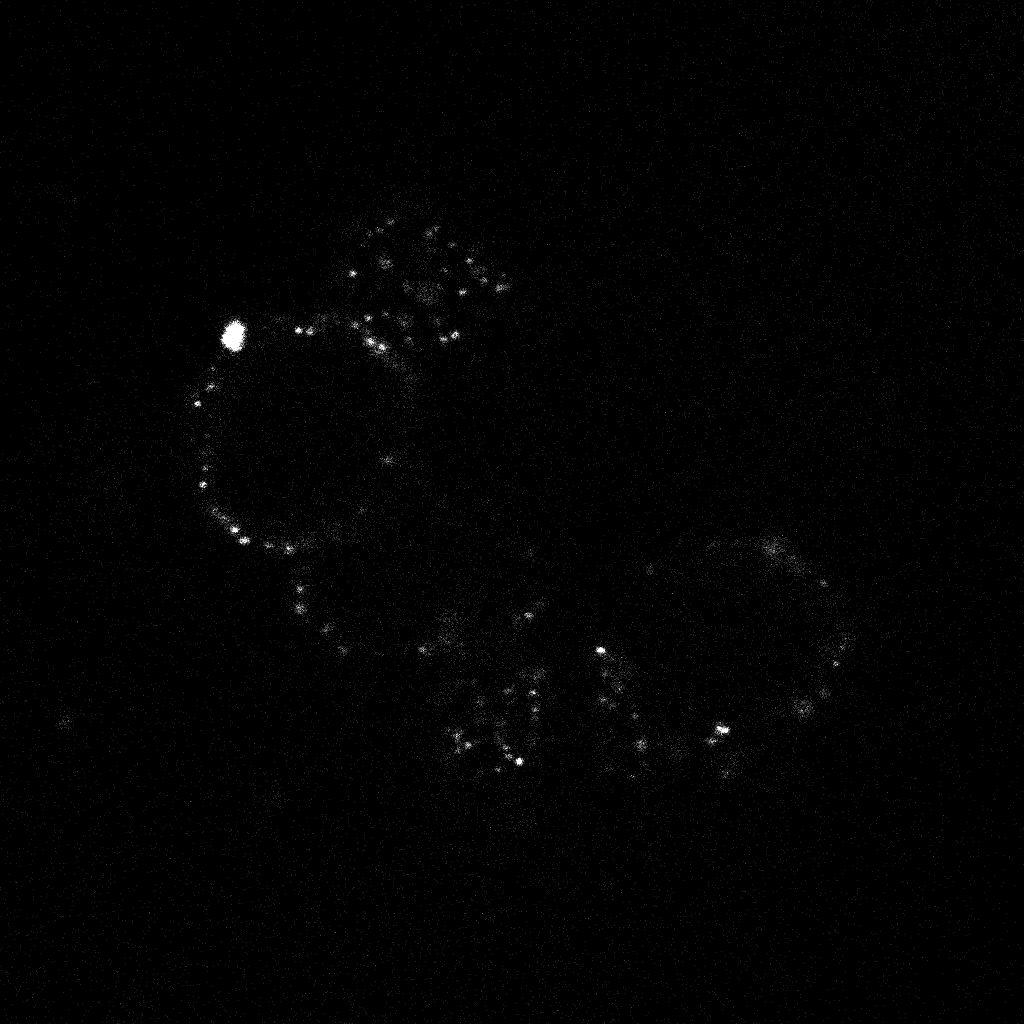

Supplement: Supplementary file 8 — Source data Fig. 1 [file 44319_2025_567_MOESM8_ESM.zip › Fig1/1D/Counted_nuclei/Mechanical/Nuc_10+11+12+13+14/Nuc_10+11+12+13+14_z19_RAW_ch00.tif]

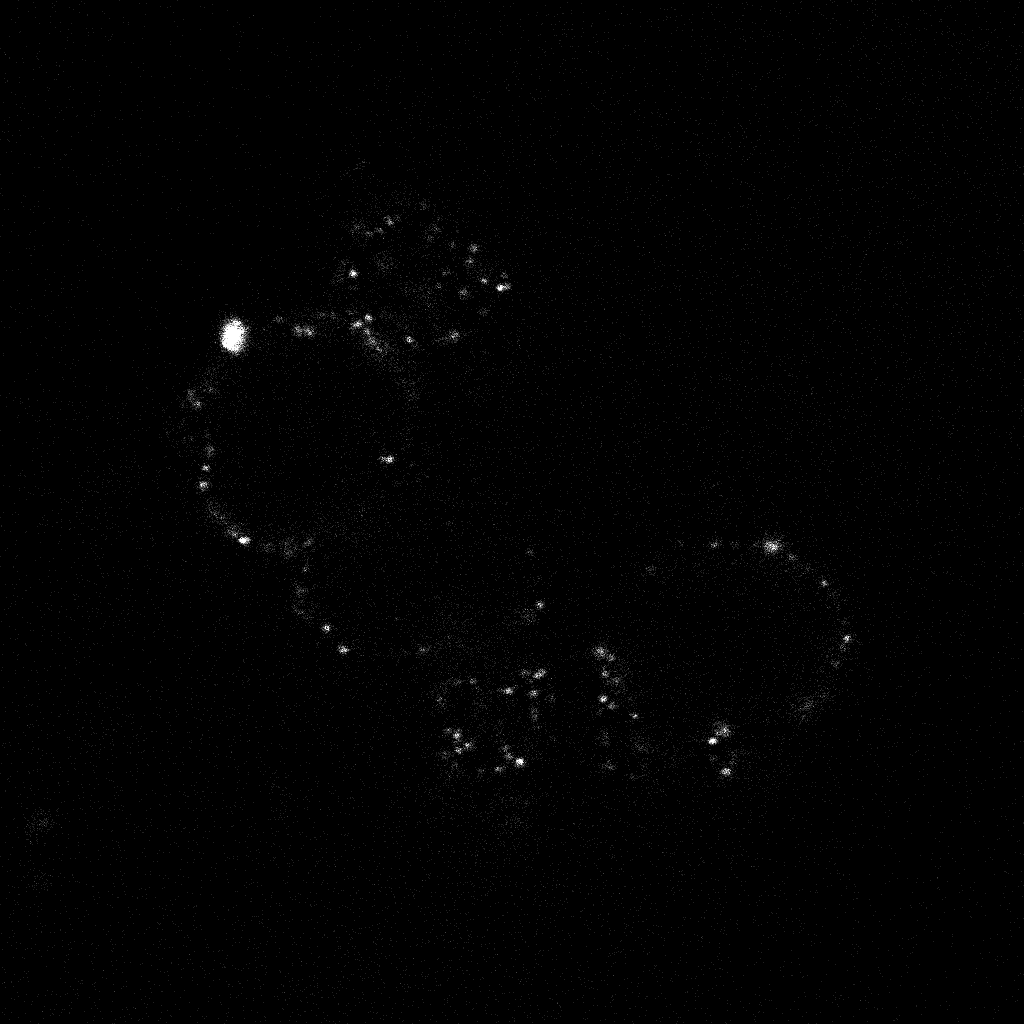

Supplement: Supplementary file 8 — Source data Fig. 1 [file 44319_2025_567_MOESM8_ESM.zip › Fig1/1D/Counted_nuclei/Mechanical/Nuc_10+11+12+13+14/Nuc_10+11+12+13+14_z20_RAW_ch00.tif]

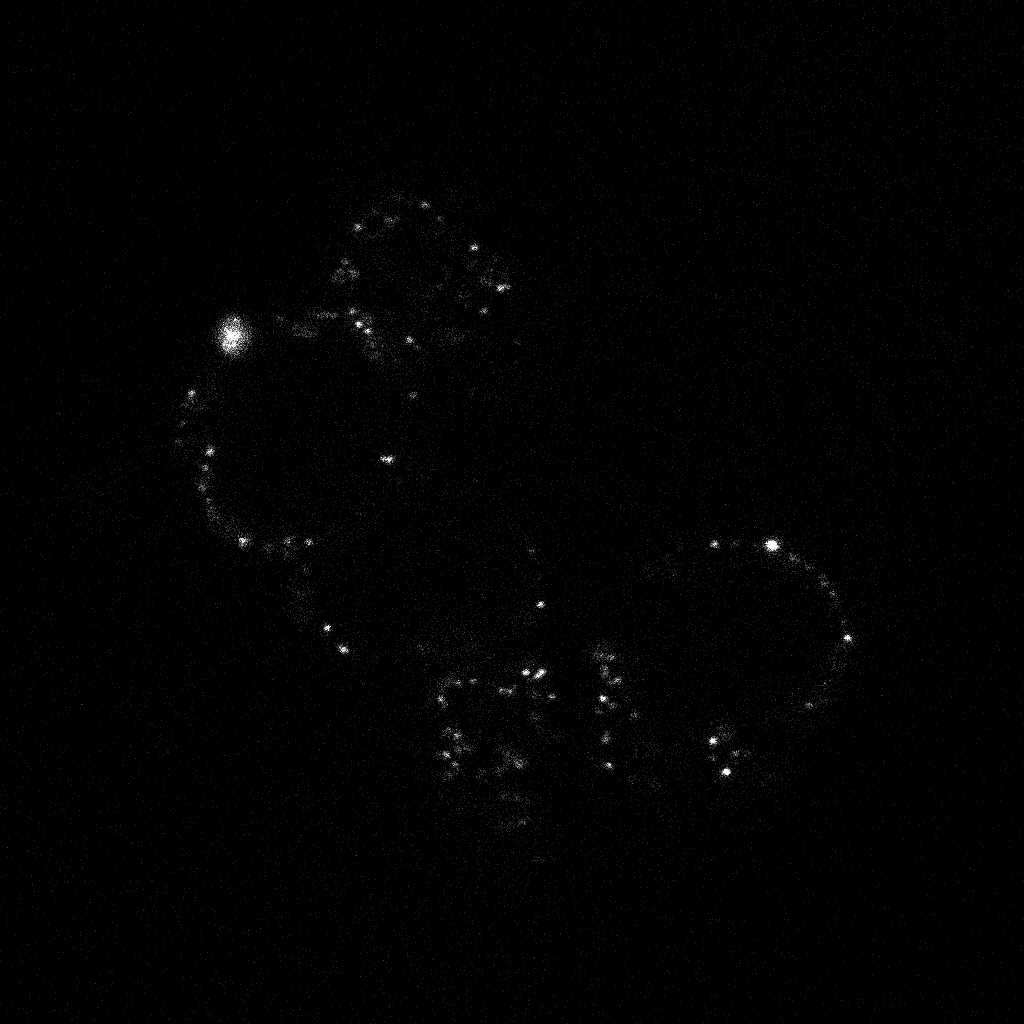

Supplement: Supplementary file 8 — Source data Fig. 1 [file 44319_2025_567_MOESM8_ESM.zip › Fig1/1D/Counted_nuclei/Mechanical/Nuc_10+11+12+13+14/Nuc_10+11+12+13+14_z21_RAW_ch00.tif]

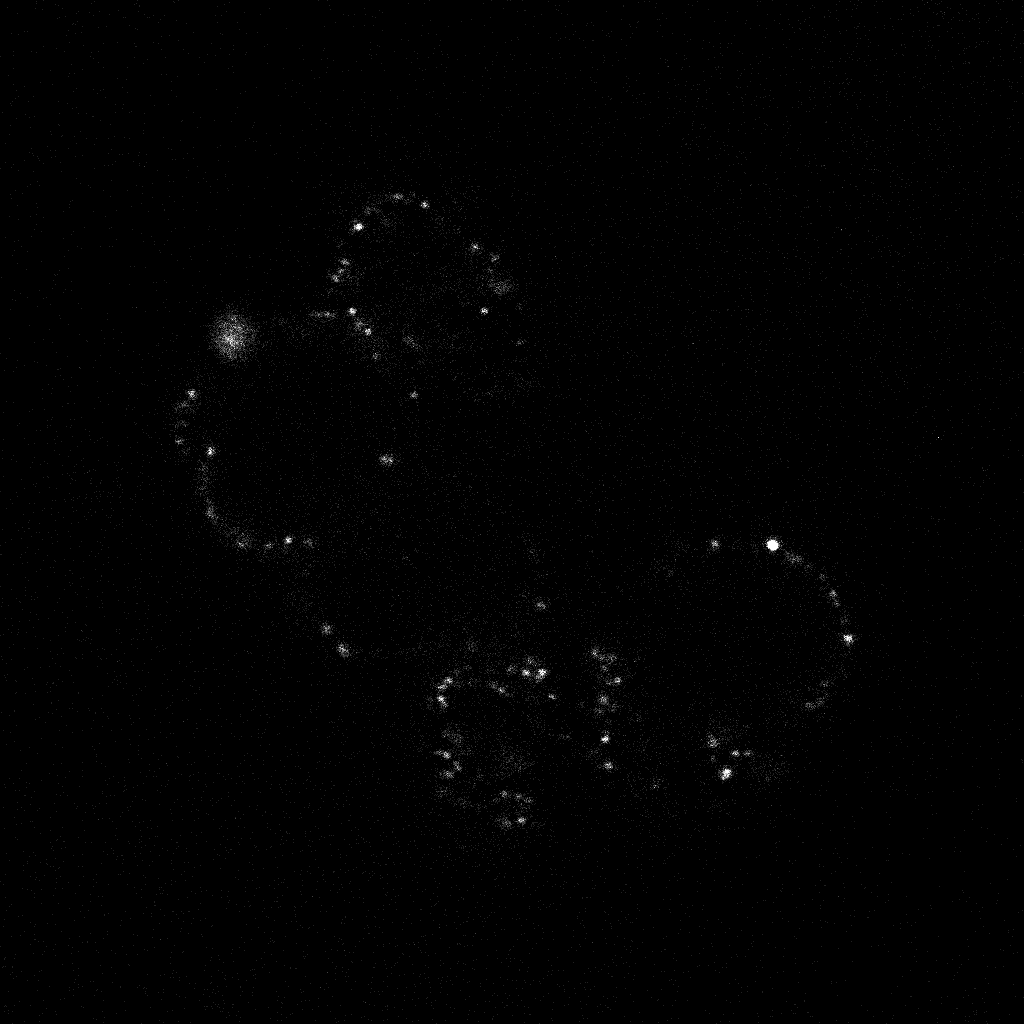

Supplement: Supplementary file 8 — Source data Fig. 1 [file 44319_2025_567_MOESM8_ESM.zip › Fig1/1D/Counted_nuclei/Mechanical/Nuc_10+11+12+13+14/Nuc_10+11+12+13+14_z22_RAW_ch00.tif]

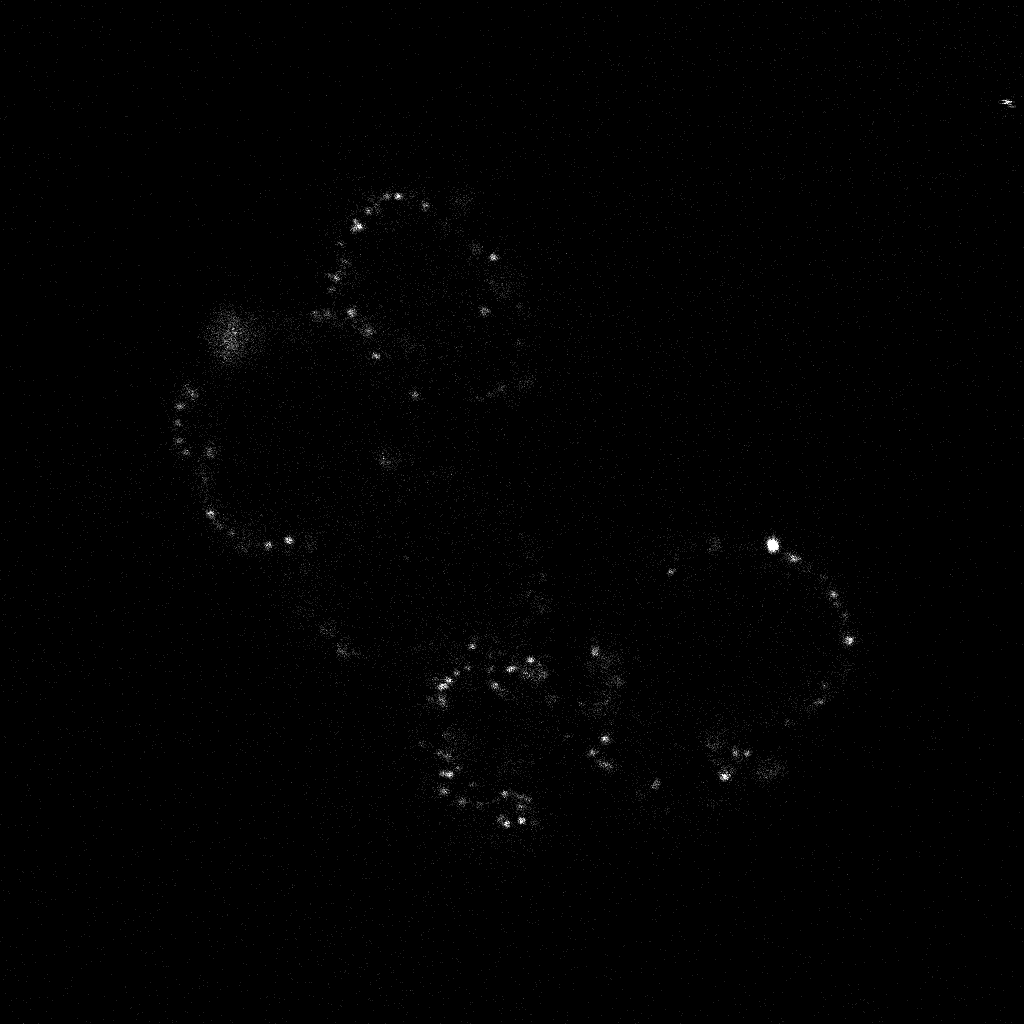

Supplement: Supplementary file 8 — Source data Fig. 1 [file 44319_2025_567_MOESM8_ESM.zip › Fig1/1D/Counted_nuclei/Mechanical/Nuc_10+11+12+13+14/Nuc_10+11+12+13+14_z23_RAW_ch00.tif]

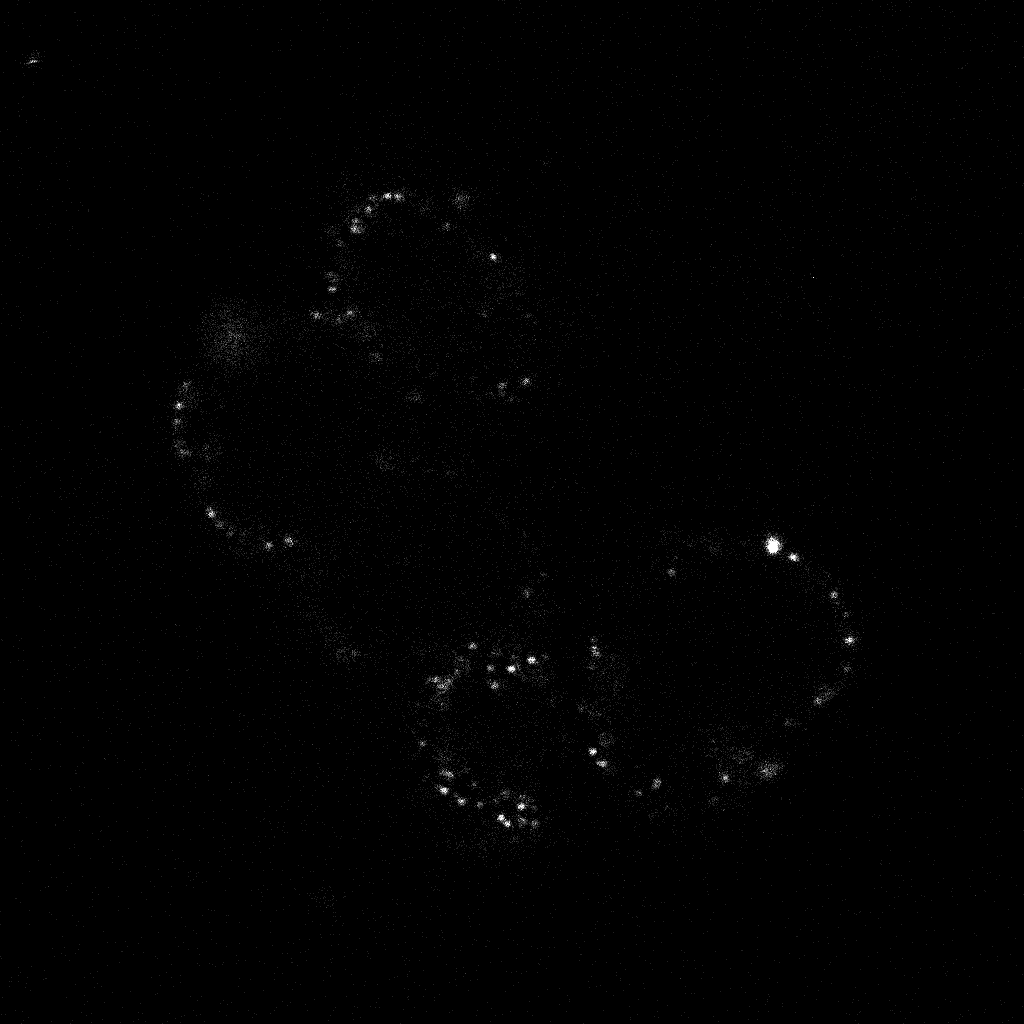

Supplement: Supplementary file 8 — Source data Fig. 1 [file 44319_2025_567_MOESM8_ESM.zip › Fig1/1D/Counted_nuclei/Mechanical/Nuc_10+11+12+13+14/Nuc_10+11+12+13+14_z24_RAW_ch00.tif]

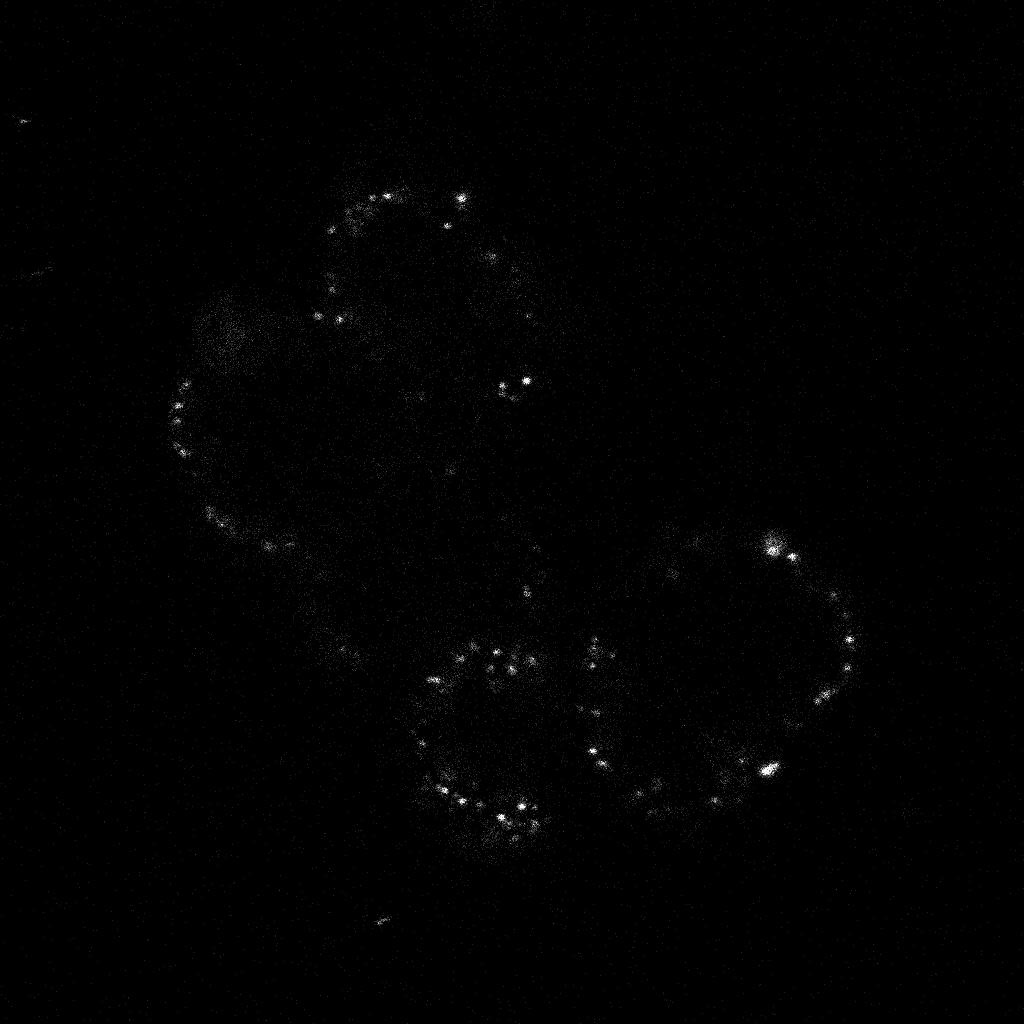

Supplement: Supplementary file 8 — Source data Fig. 1 [file 44319_2025_567_MOESM8_ESM.zip › Fig1/1D/Counted_nuclei/Mechanical/Nuc_10+11+12+13+14/Nuc_10+11+12+13+14_z25_RAW_ch00.tif]

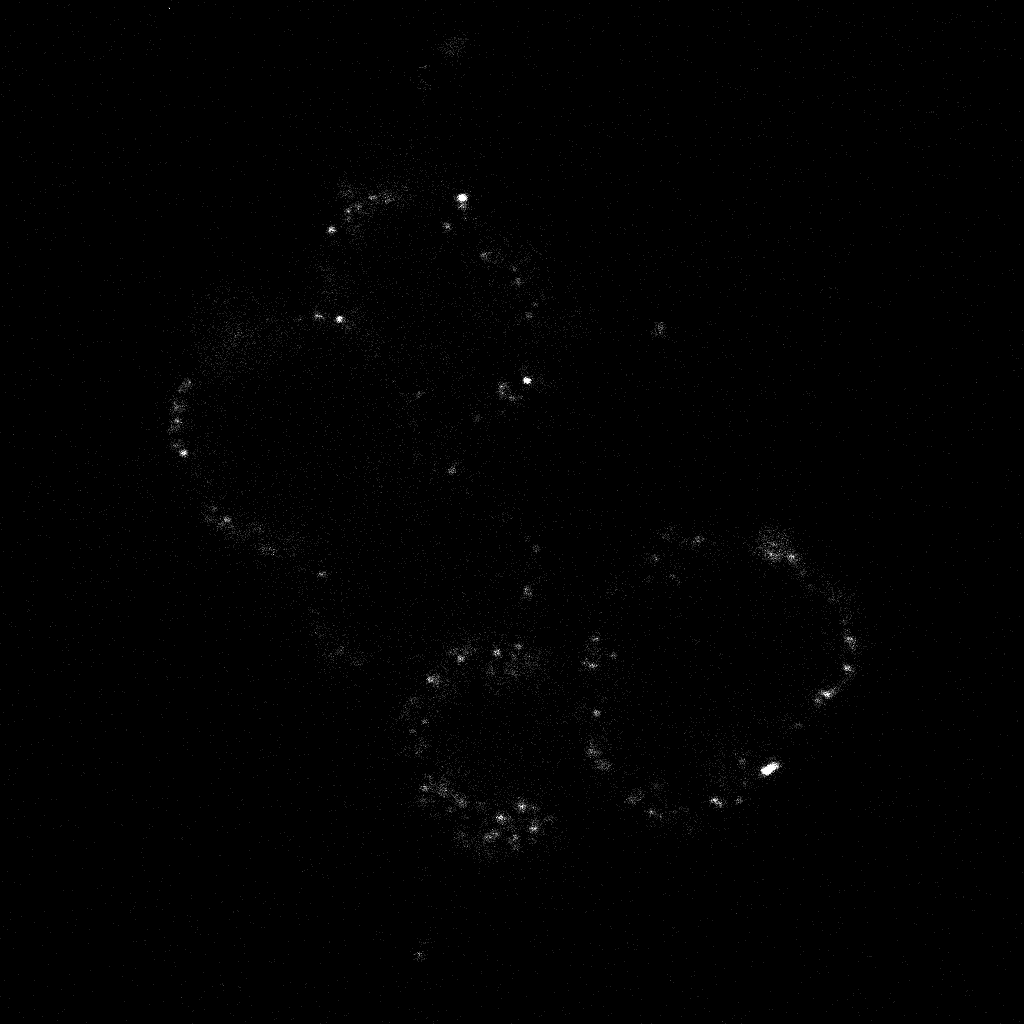

Supplement: Supplementary file 8 — Source data Fig. 1 [file 44319_2025_567_MOESM8_ESM.zip › Fig1/1D/Counted_nuclei/Mechanical/Nuc_10+11+12+13+14/Nuc_10+11+12+13+14_z26_RAW_ch00.tif]

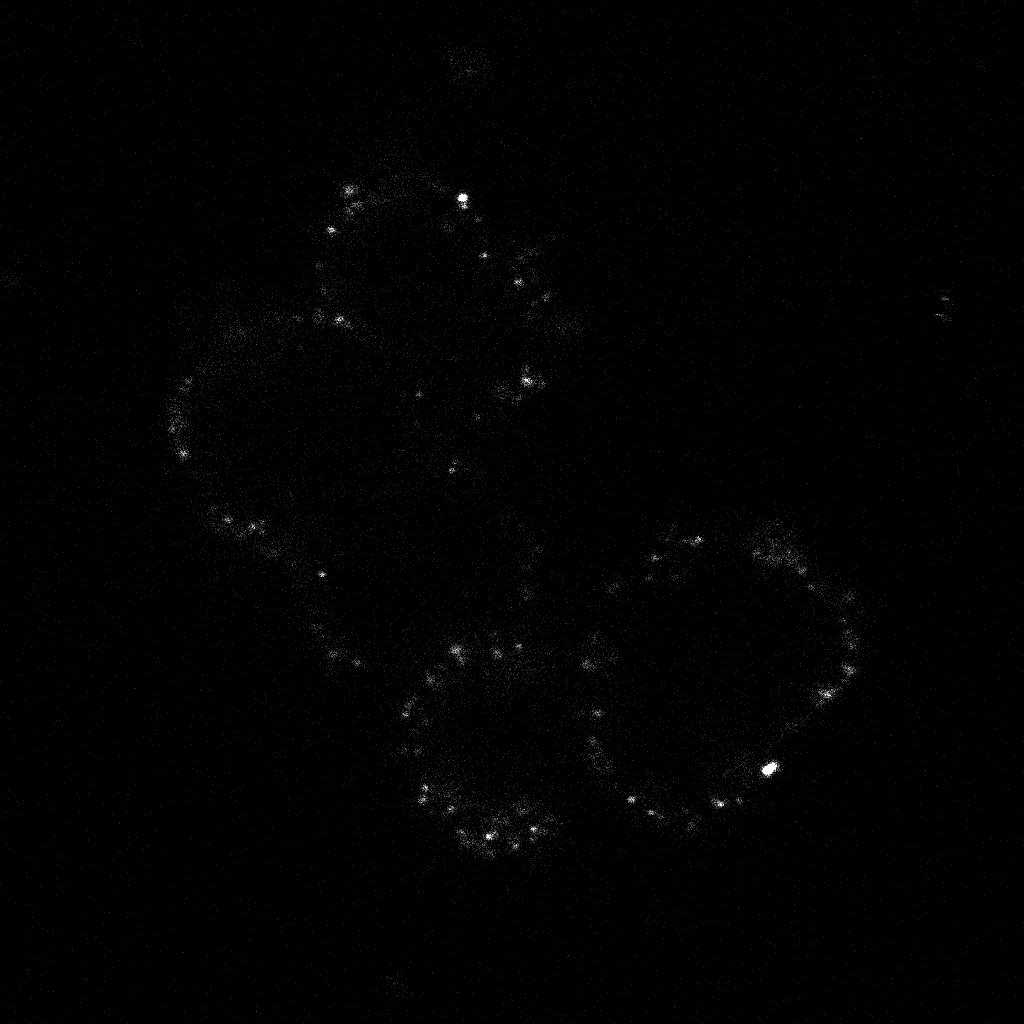

Supplement: Supplementary file 8 — Source data Fig. 1 [file 44319_2025_567_MOESM8_ESM.zip › Fig1/1D/Counted_nuclei/Mechanical/Nuc_10+11+12+13+14/Nuc_10+11+12+13+14_z27_RAW_ch00.tif]

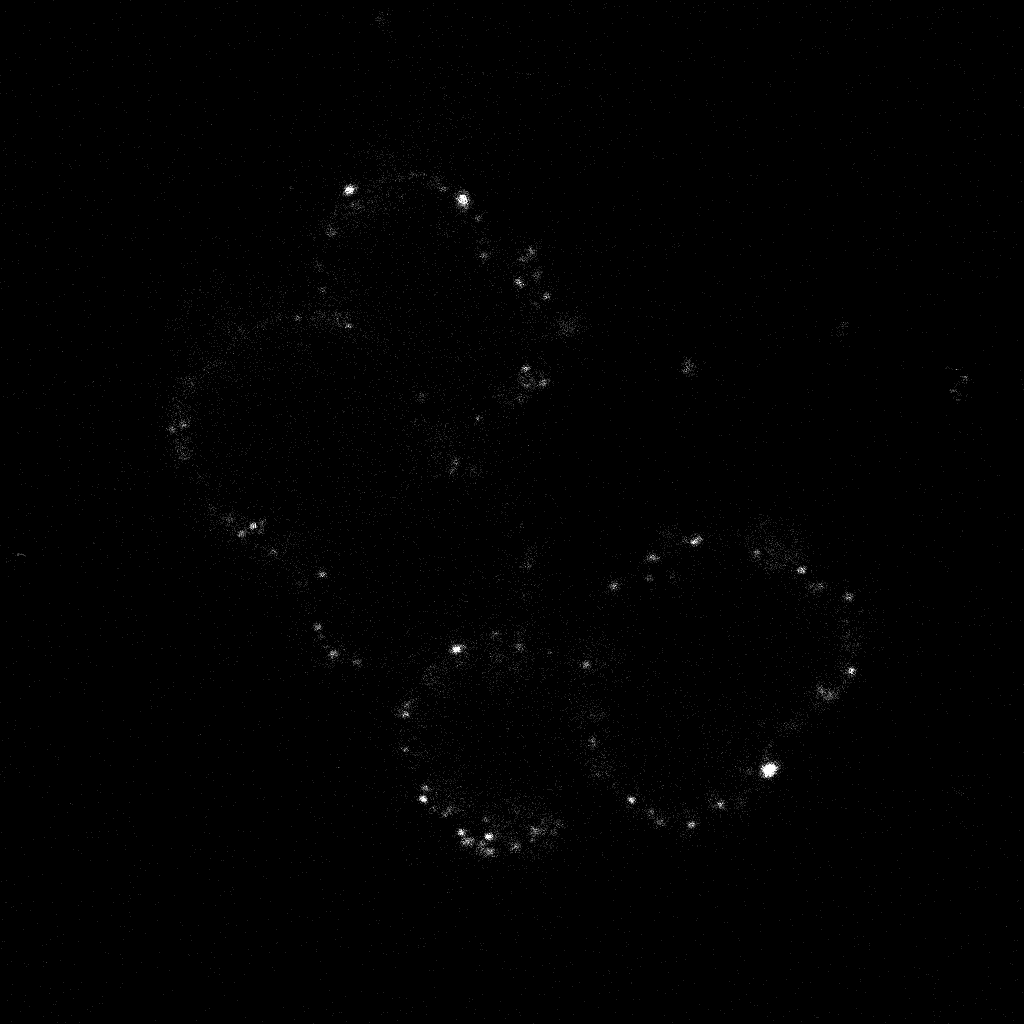

Supplement: Supplementary file 8 — Source data Fig. 1 [file 44319_2025_567_MOESM8_ESM.zip › Fig1/1D/Counted_nuclei/Mechanical/Nuc_10+11+12+13+14/Nuc_10+11+12+13+14_z28_RAW_ch00.tif]

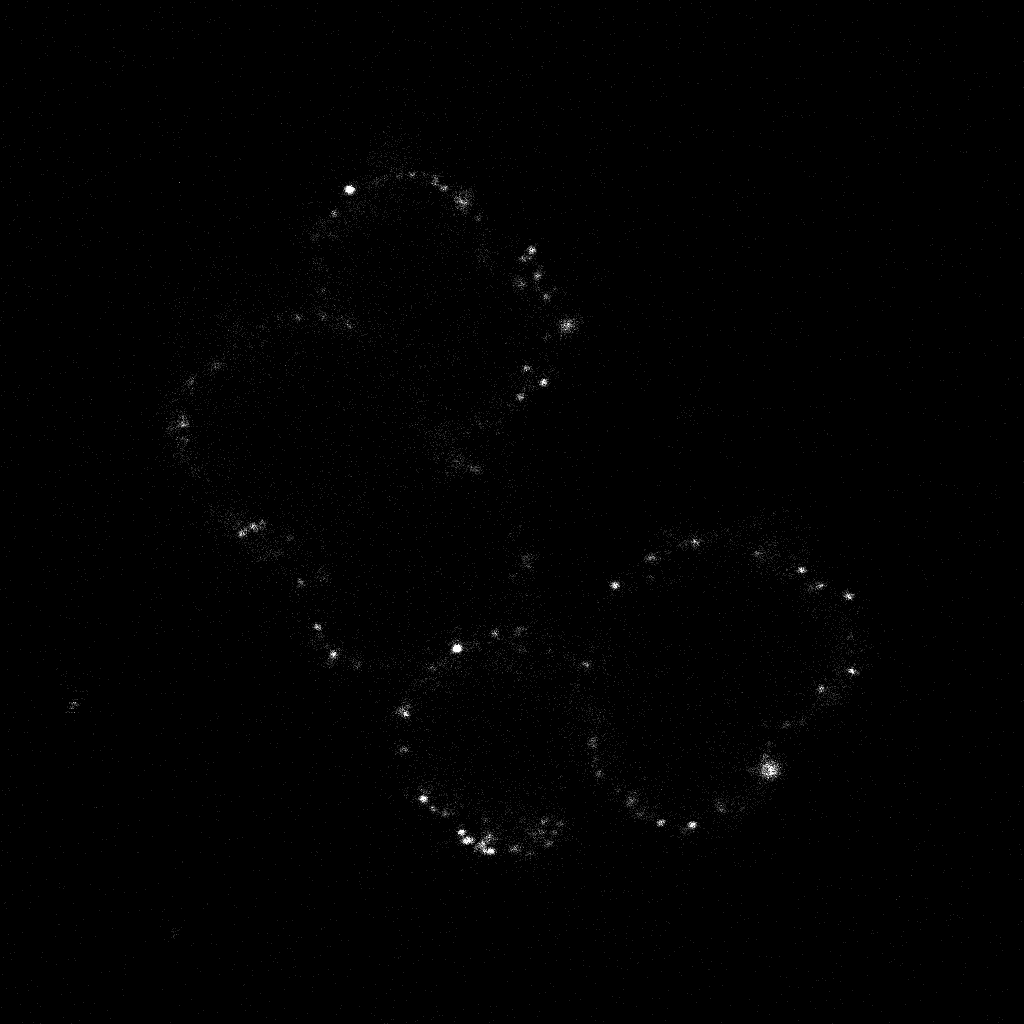

Supplement: Supplementary file 8 — Source data Fig. 1 [file 44319_2025_567_MOESM8_ESM.zip › Fig1/1D/Counted_nuclei/Mechanical/Nuc_10+11+12+13+14/Nuc_10+11+12+13+14_z29_RAW_ch00.tif]

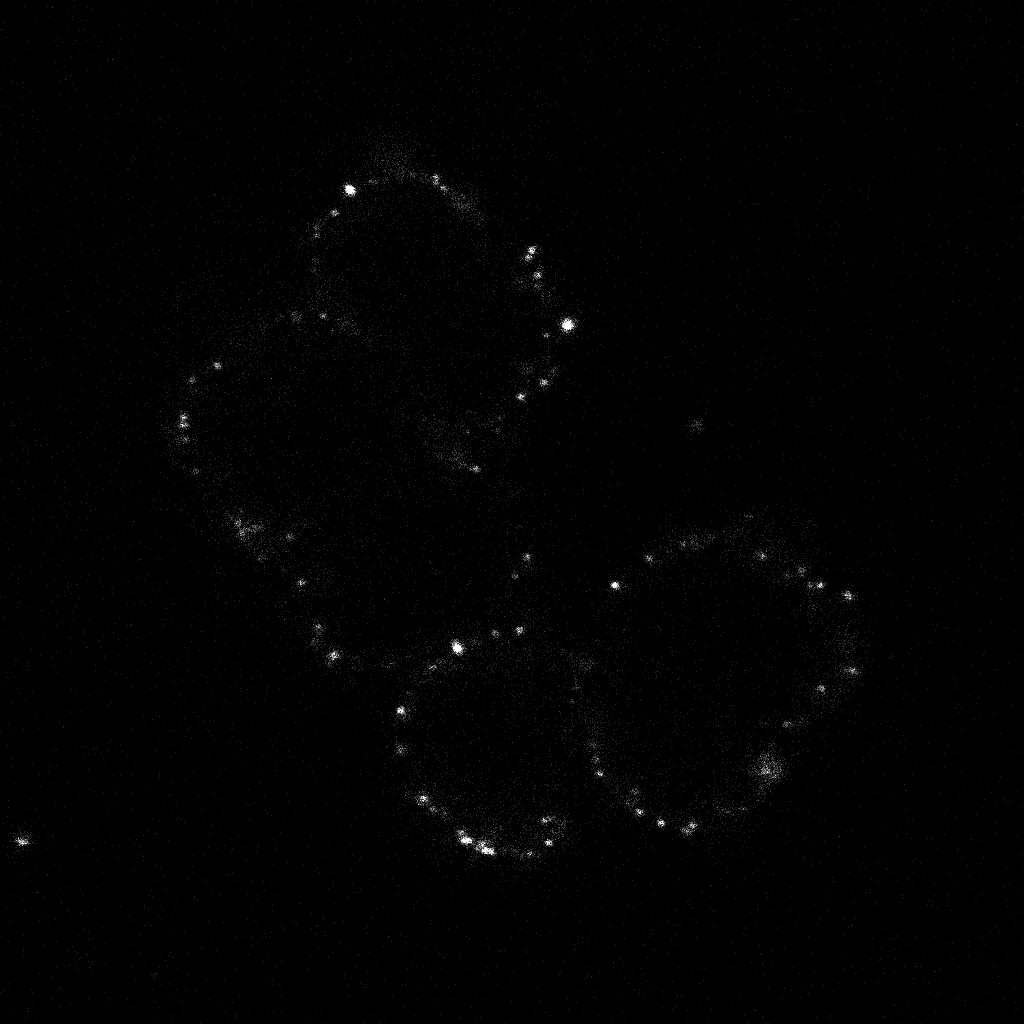

Supplement: Supplementary file 8 — Source data Fig. 1 [file 44319_2025_567_MOESM8_ESM.zip › Fig1/1D/Counted_nuclei/Mechanical/Nuc_10+11+12+13+14/Nuc_10+11+12+13+14_z30_RAW_ch00.tif]

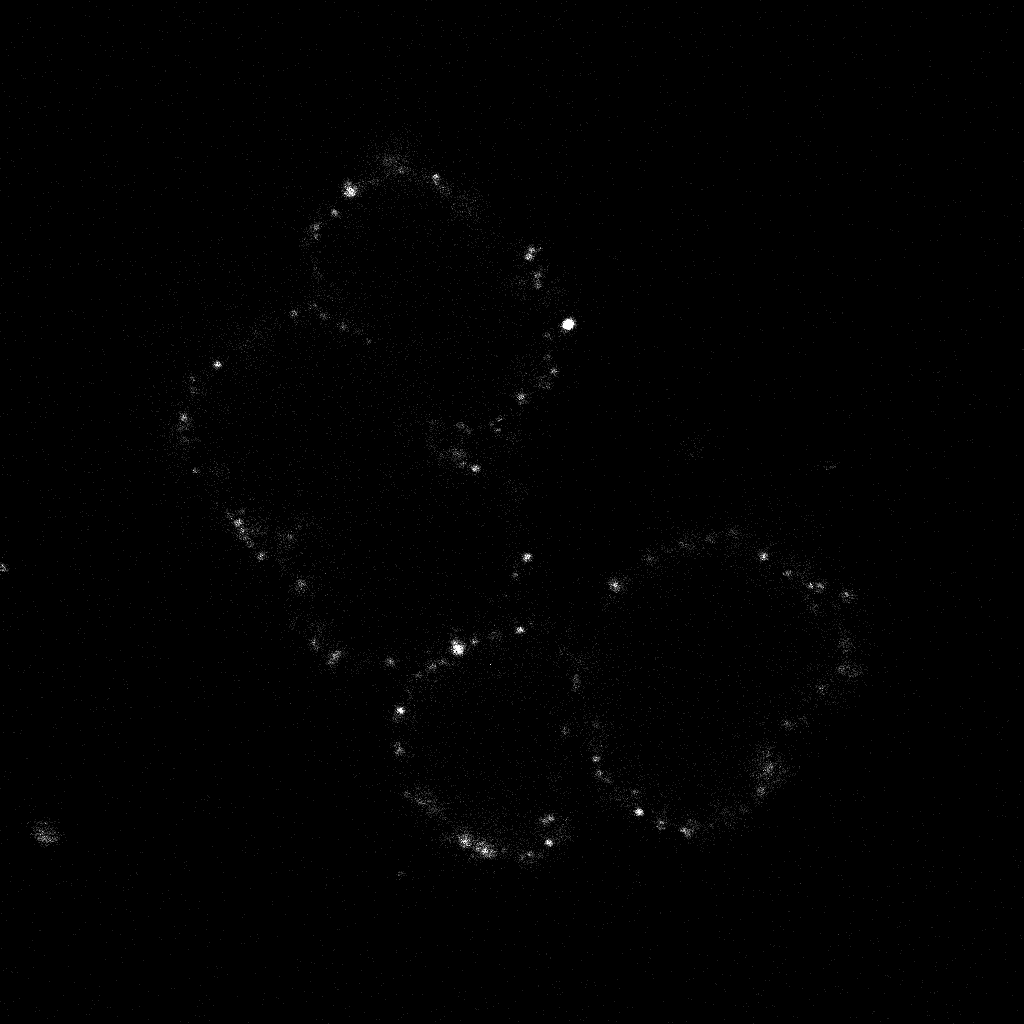

Supplement: Supplementary file 8 — Source data Fig. 1 [file 44319_2025_567_MOESM8_ESM.zip › Fig1/1D/Counted_nuclei/Mechanical/Nuc_10+11+12+13+14/Nuc_10+11+12+13+14_z31_RAW_ch00.tif]

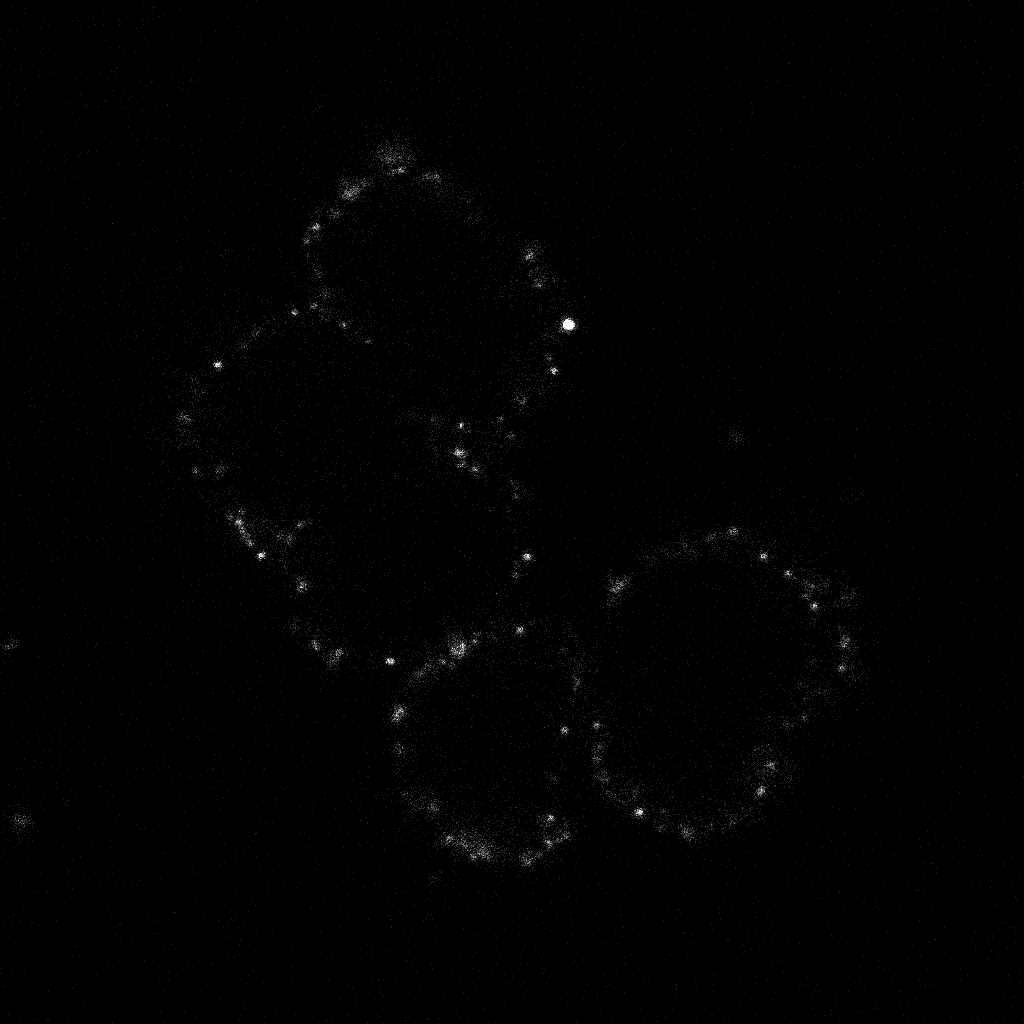

Supplement: Supplementary file 8 — Source data Fig. 1 [file 44319_2025_567_MOESM8_ESM.zip › Fig1/1D/Counted_nuclei/Mechanical/Nuc_10+11+12+13+14/Nuc_10+11+12+13+14_z32_RAW_ch00.tif]

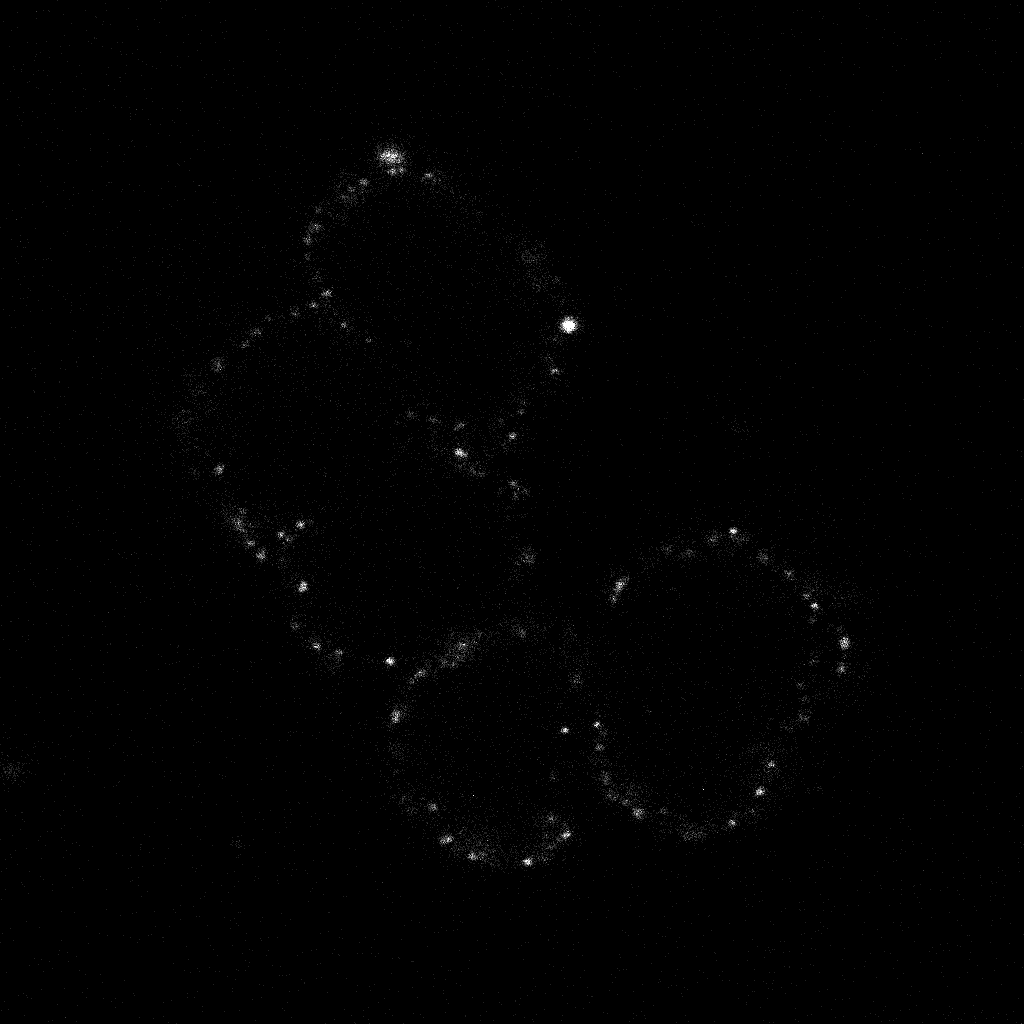

Supplement: Supplementary file 8 — Source data Fig. 1 [file 44319_2025_567_MOESM8_ESM.zip › Fig1/1D/Counted_nuclei/Mechanical/Nuc_10+11+12+13+14/Nuc_10+11+12+13+14_z33_RAW_ch00.tif]

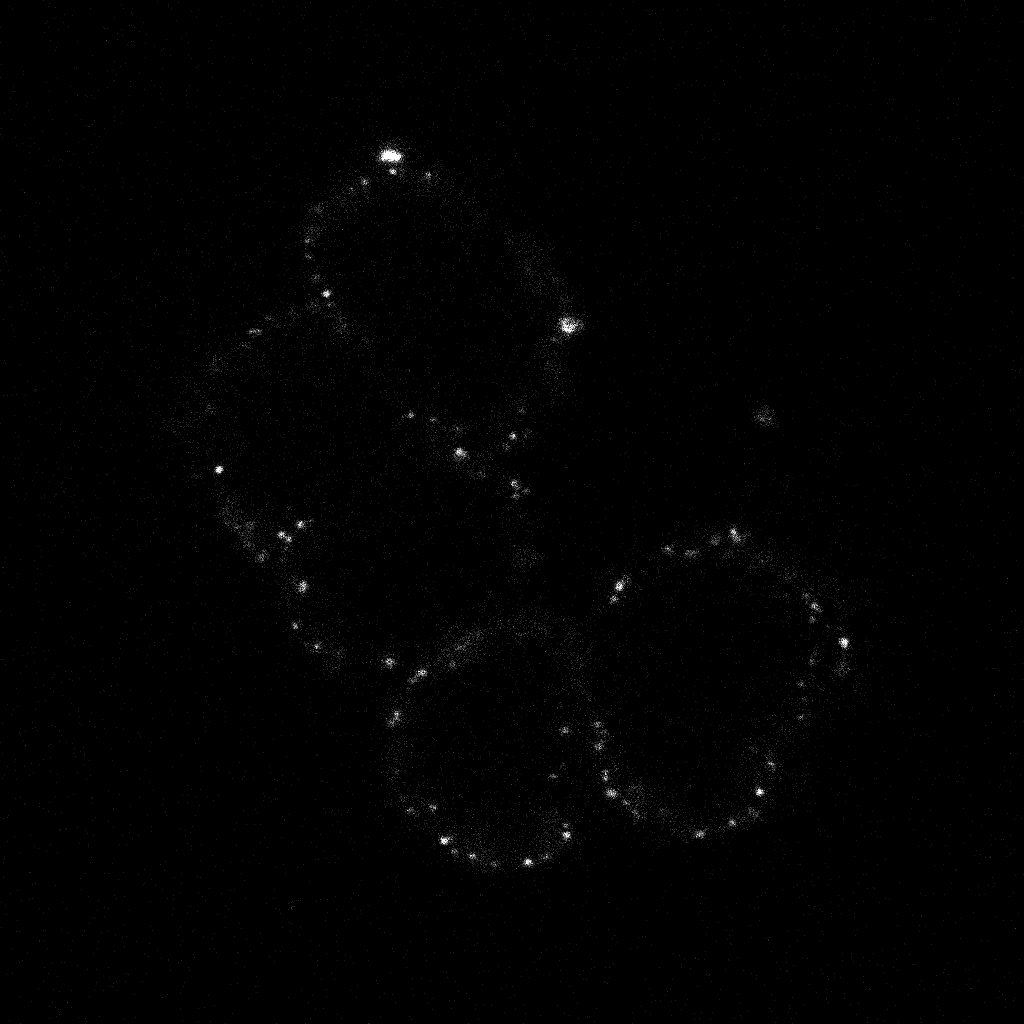

Supplement: Supplementary file 8 — Source data Fig. 1 [file 44319_2025_567_MOESM8_ESM.zip › Fig1/1D/Counted_nuclei/Mechanical/Nuc_10+11+12+13+14/Nuc_10+11+12+13+14_z34_RAW_ch00.tif]

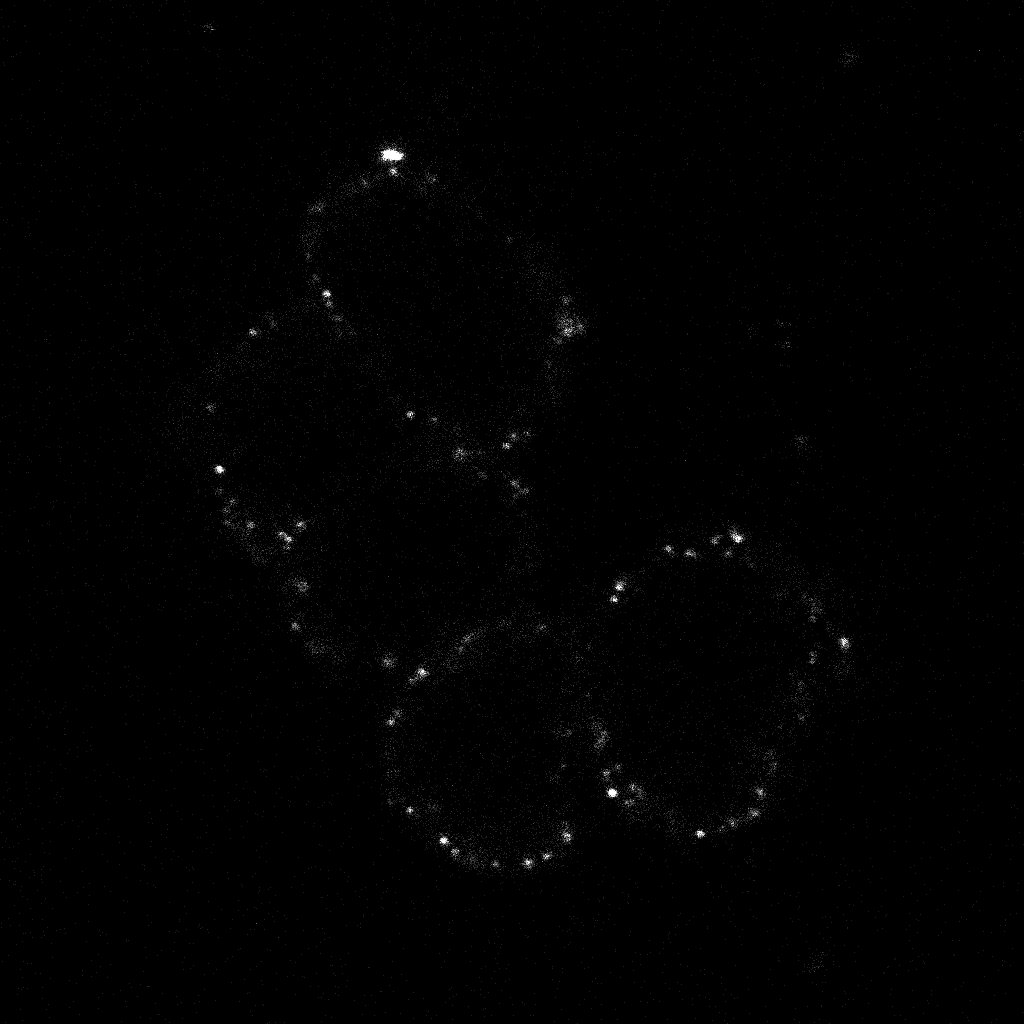

Supplement: Supplementary file 8 — Source data Fig. 1 [file 44319_2025_567_MOESM8_ESM.zip › Fig1/1D/Counted_nuclei/Mechanical/Nuc_10+11+12+13+14/Nuc_10+11+12+13+14_z35_RAW_ch00.tif]

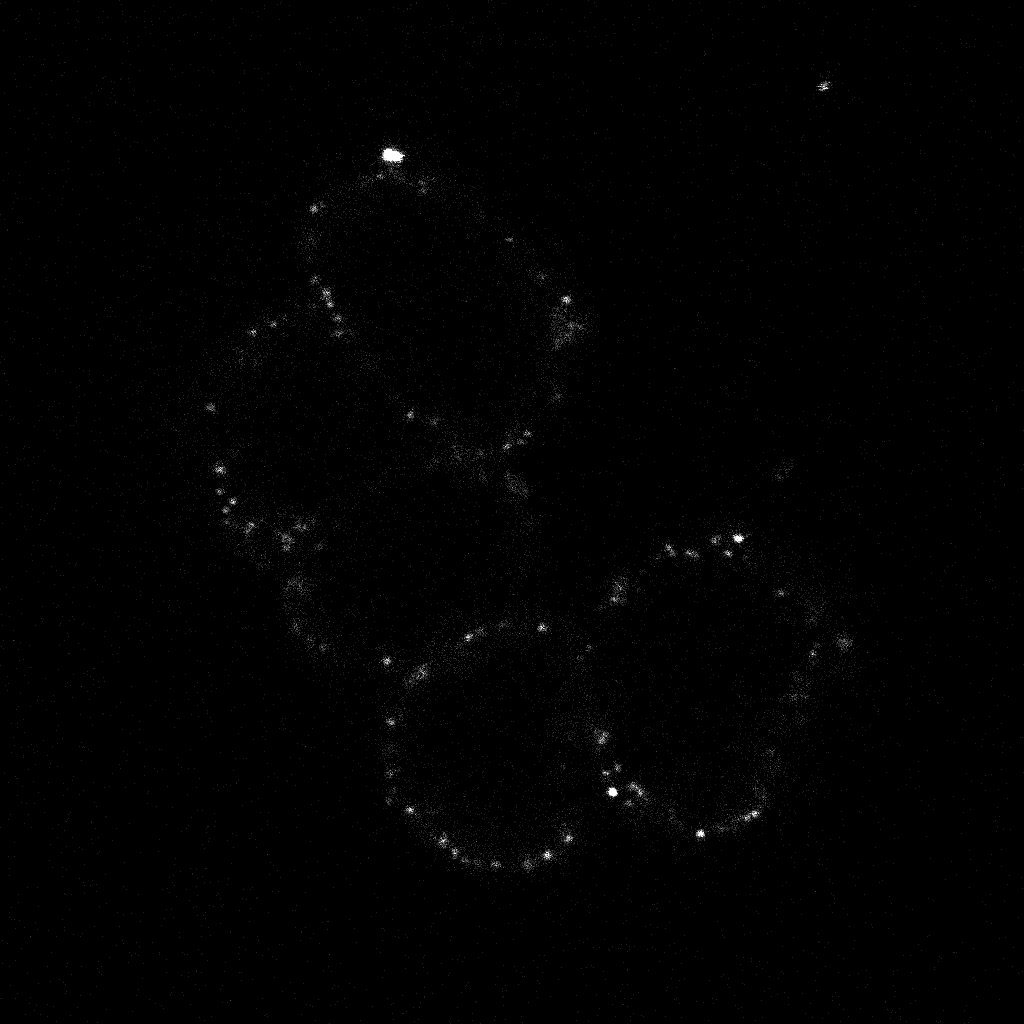

Supplement: Supplementary file 8 — Source data Fig. 1 [file 44319_2025_567_MOESM8_ESM.zip › Fig1/1D/Counted_nuclei/Mechanical/Nuc_10+11+12+13+14/Nuc_10+11+12+13+14_z36_RAW_ch00.tif]

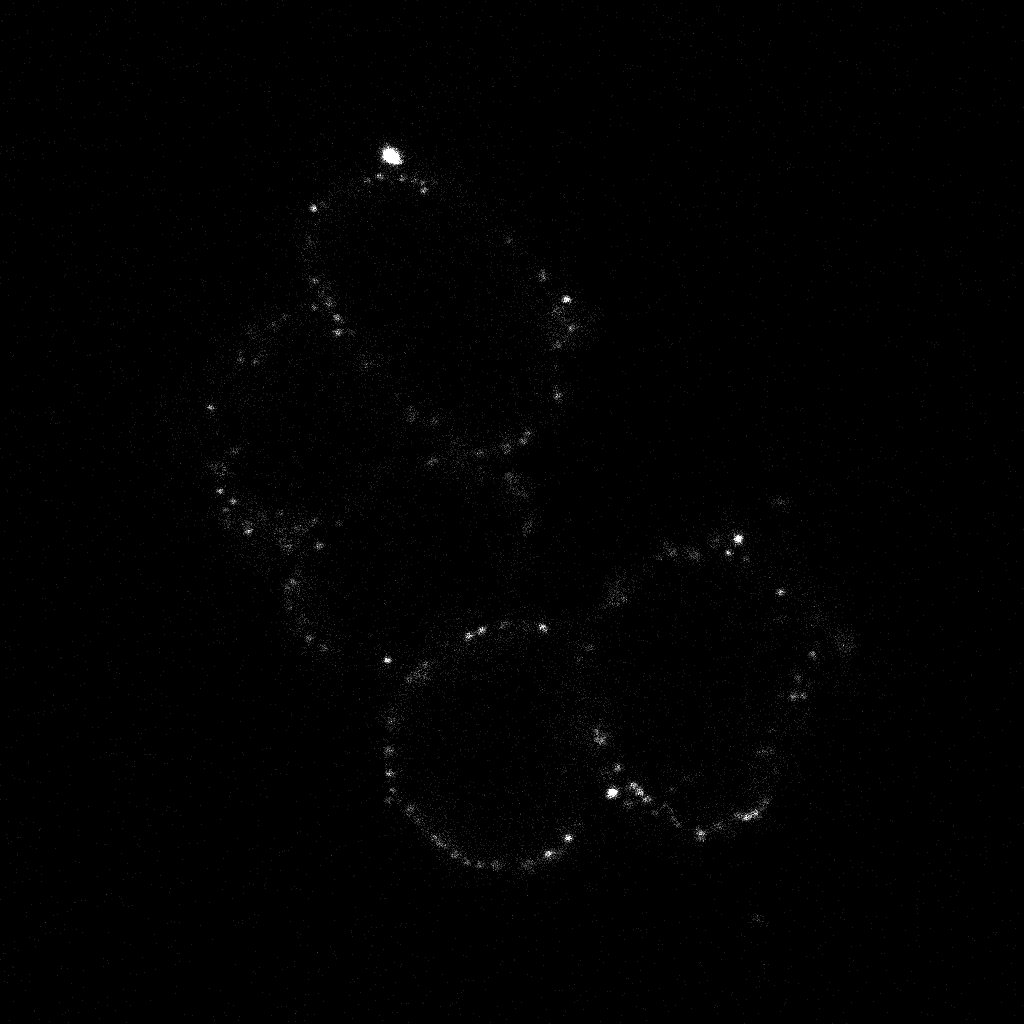

Supplement: Supplementary file 8 — Source data Fig. 1 [file 44319_2025_567_MOESM8_ESM.zip › Fig1/1D/Counted_nuclei/Mechanical/Nuc_10+11+12+13+14/Nuc_10+11+12+13+14_z37_RAW_ch00.tif]

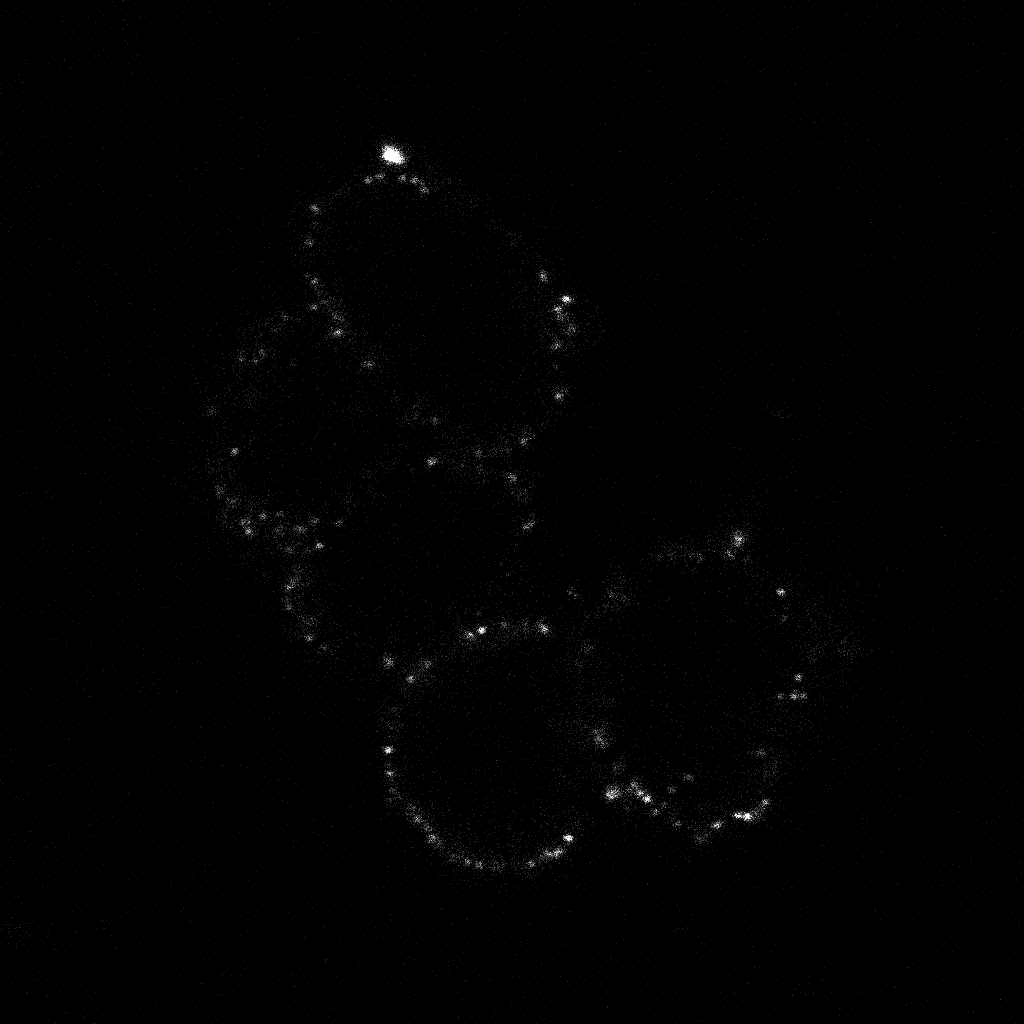

Supplement: Supplementary file 8 — Source data Fig. 1 [file 44319_2025_567_MOESM8_ESM.zip › Fig1/1D/Counted_nuclei/Mechanical/Nuc_10+11+12+13+14/Nuc_10+11+12+13+14_z38_RAW_ch00.tif]

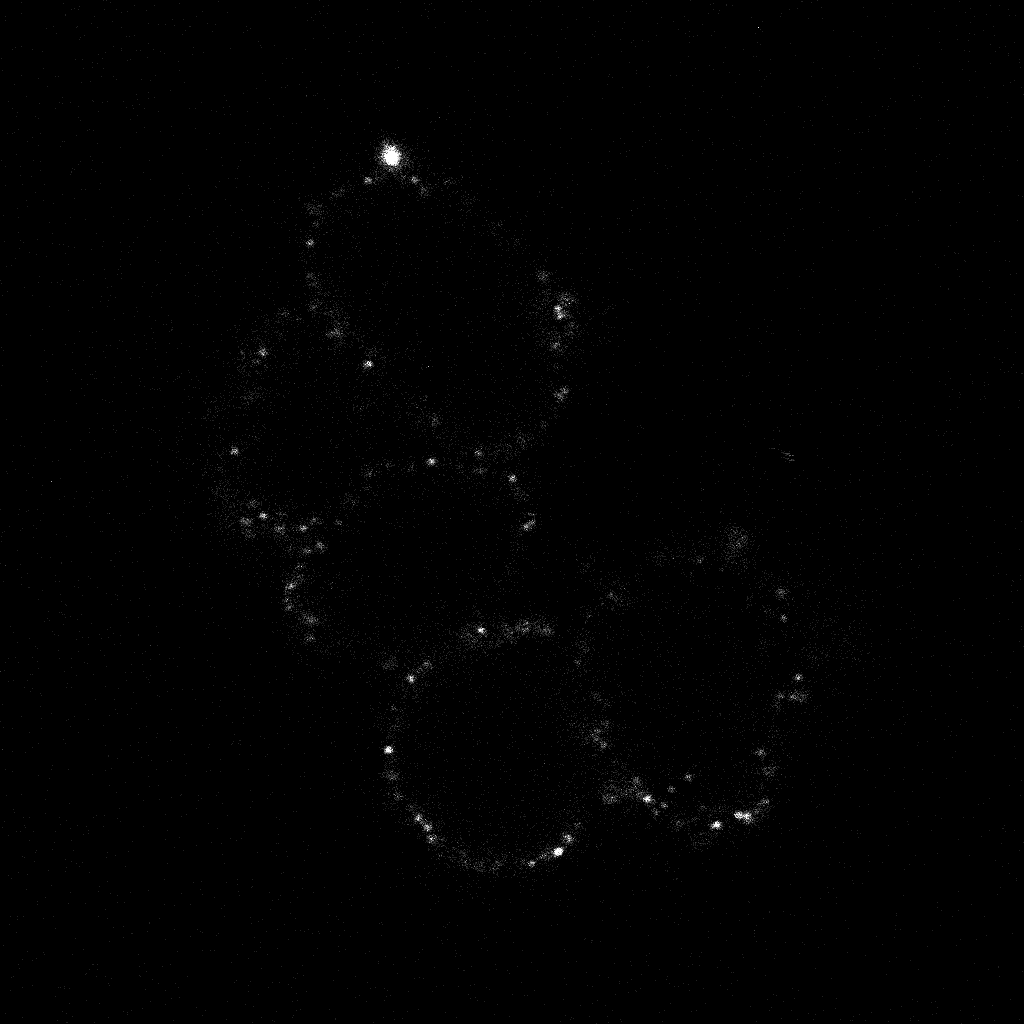

Supplement: Supplementary file 8 — Source data Fig. 1 [file 44319_2025_567_MOESM8_ESM.zip › Fig1/1D/Counted_nuclei/Mechanical/Nuc_10+11+12+13+14/Nuc_10+11+12+13+14_z39_RAW_ch00.tif]

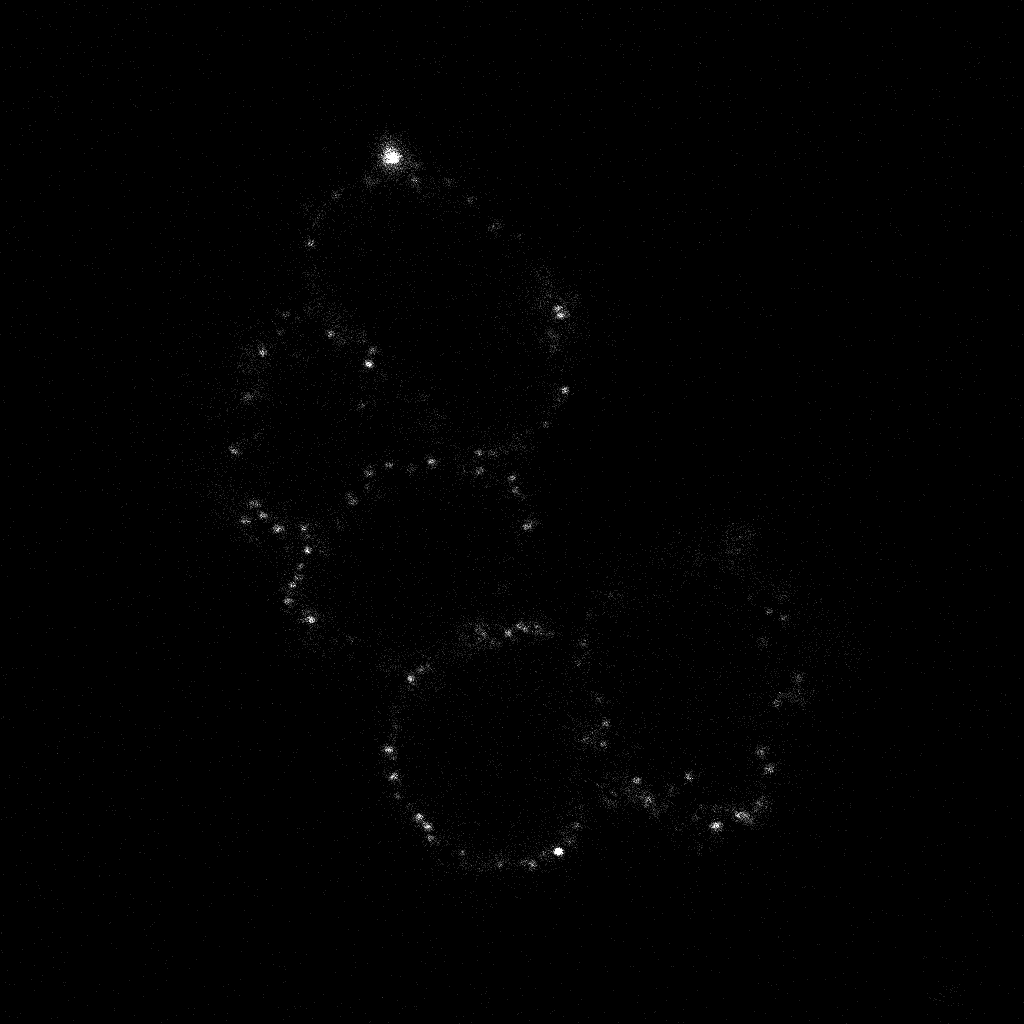

Supplement: Supplementary file 8 — Source data Fig. 1 [file 44319_2025_567_MOESM8_ESM.zip › Fig1/1D/Counted_nuclei/Mechanical/Nuc_10+11+12+13+14/Nuc_10+11+12+13+14_z40_RAW_ch00.tif]

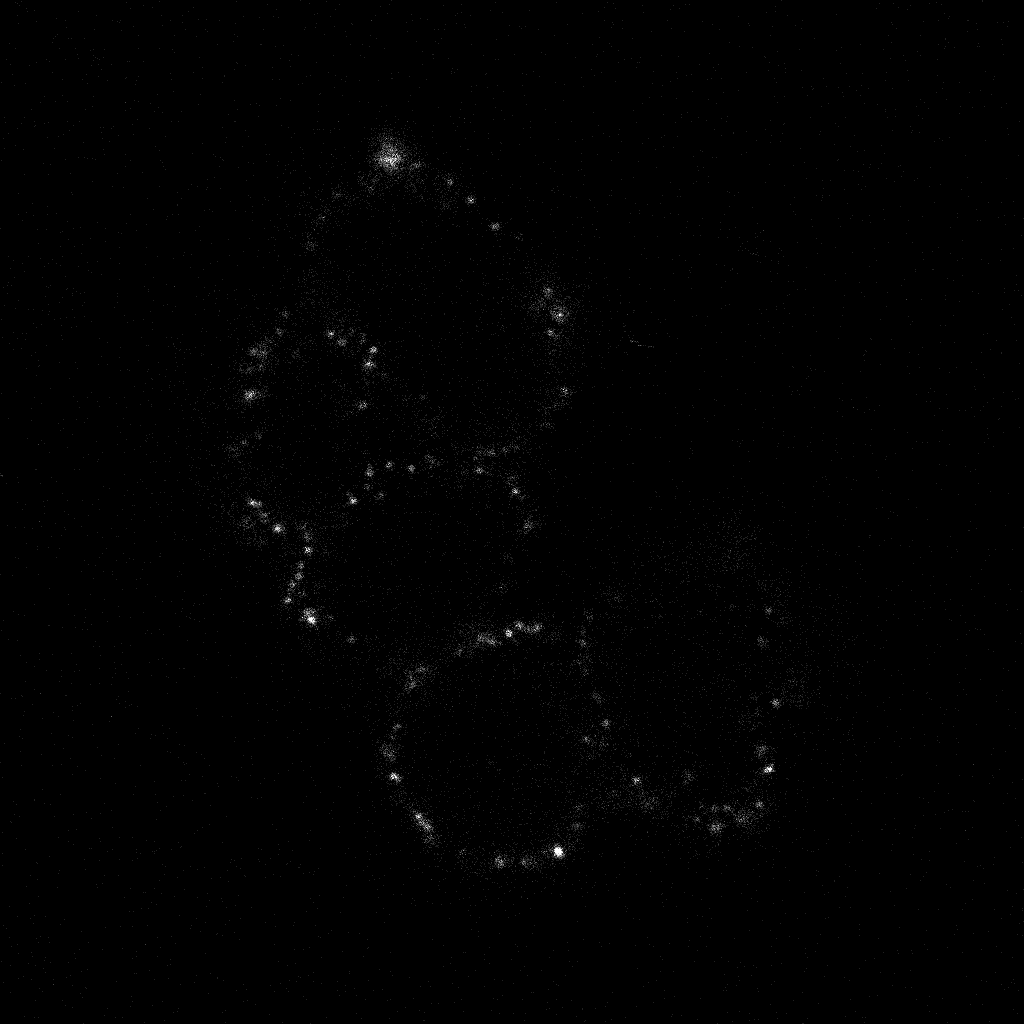

Supplement: Supplementary file 8 — Source data Fig. 1 [file 44319_2025_567_MOESM8_ESM.zip › Fig1/1D/Counted_nuclei/Mechanical/Nuc_10+11+12+13+14/Nuc_10+11+12+13+14_z41_RAW_ch00.tif]

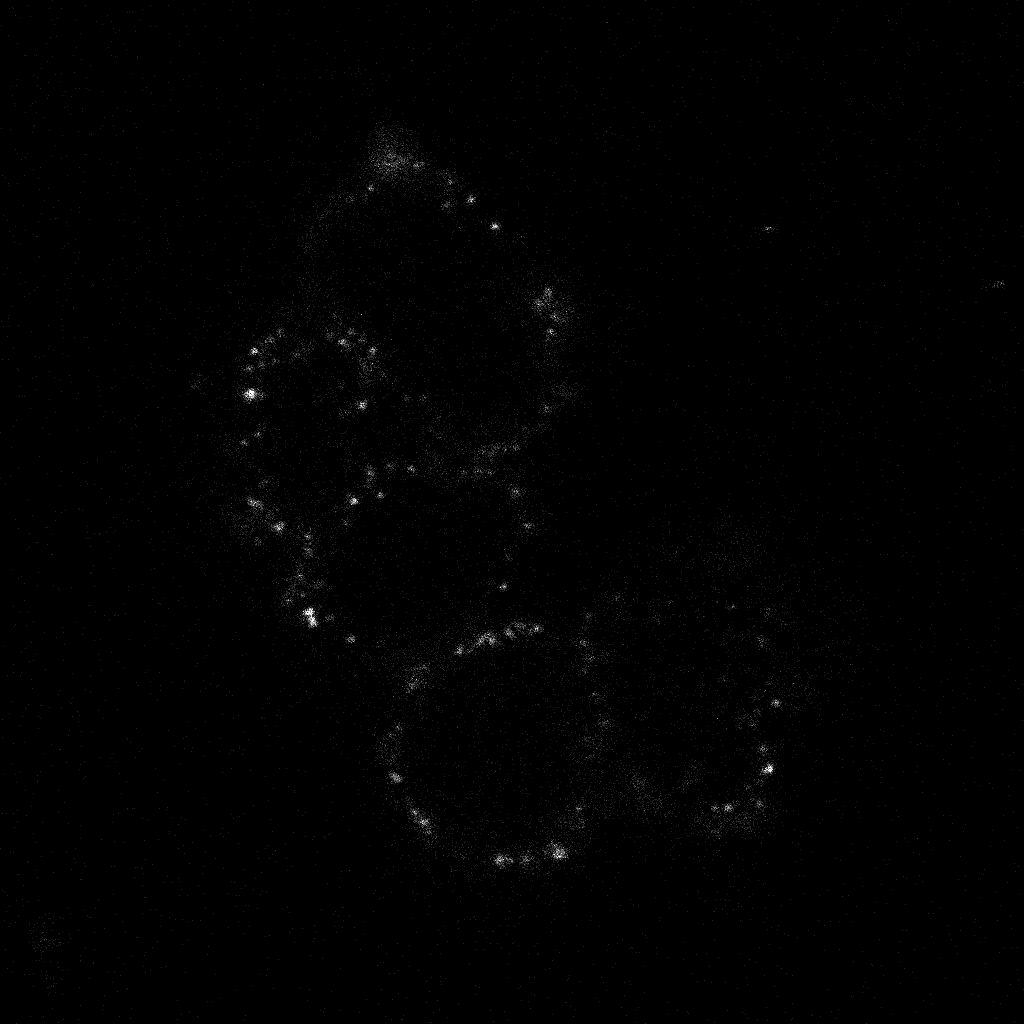

Supplement: Supplementary file 8 — Source data Fig. 1 [file 44319_2025_567_MOESM8_ESM.zip › Fig1/1D/Counted_nuclei/Mechanical/Nuc_10+11+12+13+14/Nuc_10+11+12+13+14_z42_RAW_ch00.tif]

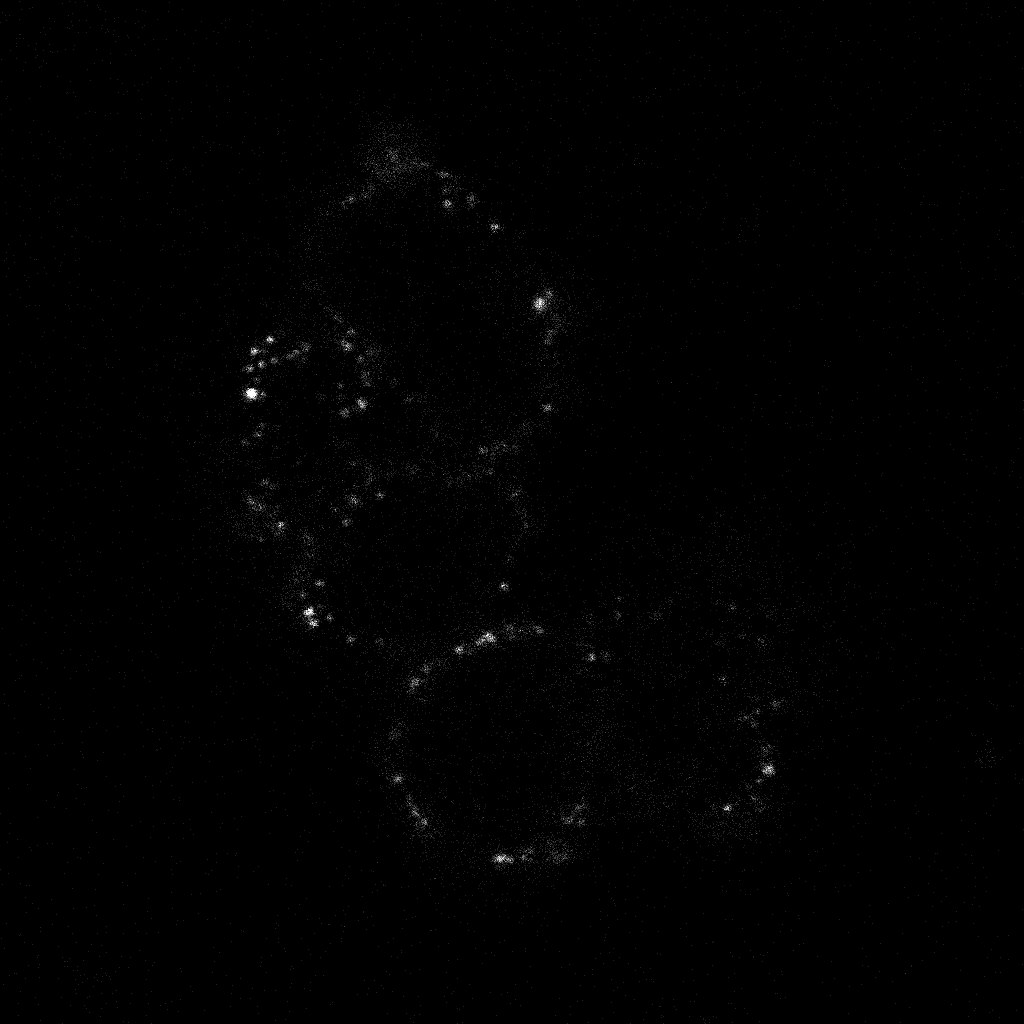

Supplement: Supplementary file 8 — Source data Fig. 1 [file 44319_2025_567_MOESM8_ESM.zip › Fig1/1D/Counted_nuclei/Mechanical/Nuc_10+11+12+13+14/Nuc_10+11+12+13+14_z43_RAW_ch00.tif]

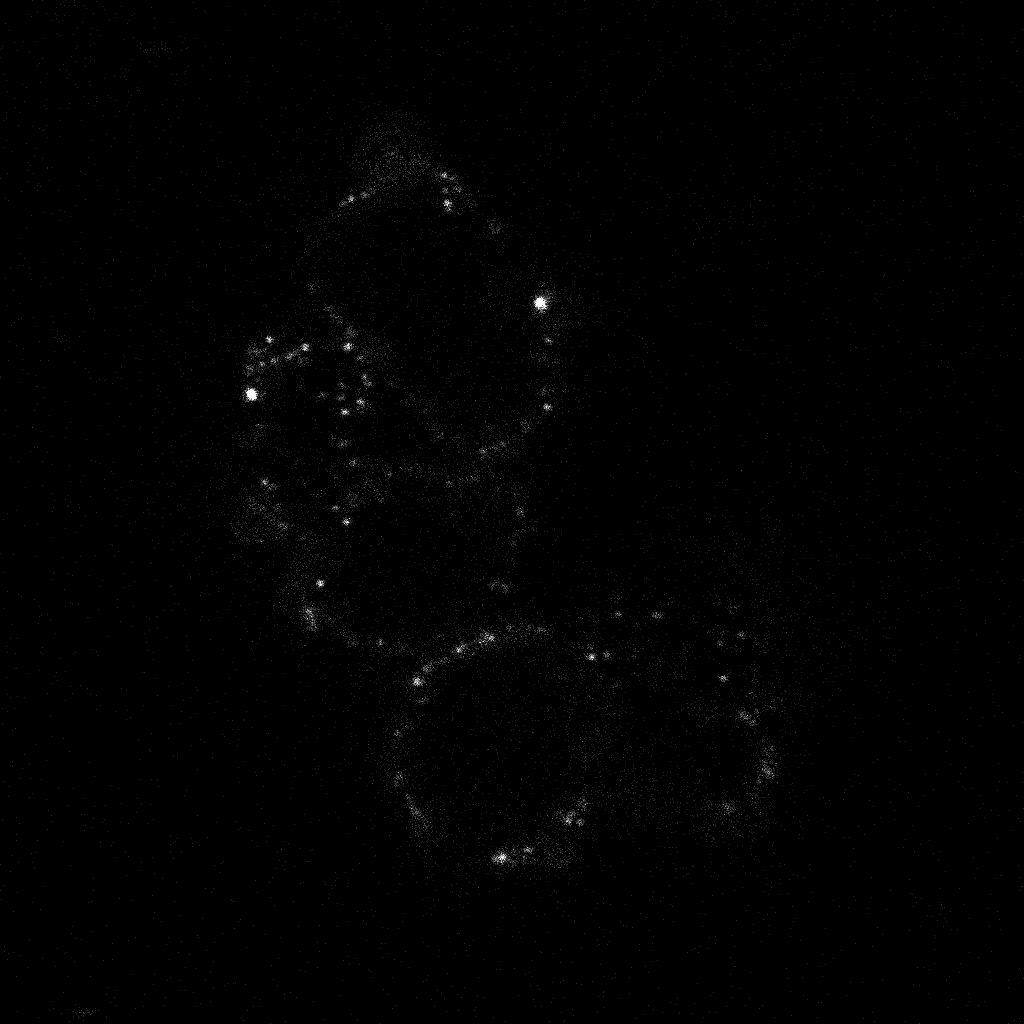

Supplement: Supplementary file 8 — Source data Fig. 1 [file 44319_2025_567_MOESM8_ESM.zip › Fig1/1D/Counted_nuclei/Mechanical/Nuc_10+11+12+13+14/Nuc_10+11+12+13+14_z44_RAW_ch00.tif]

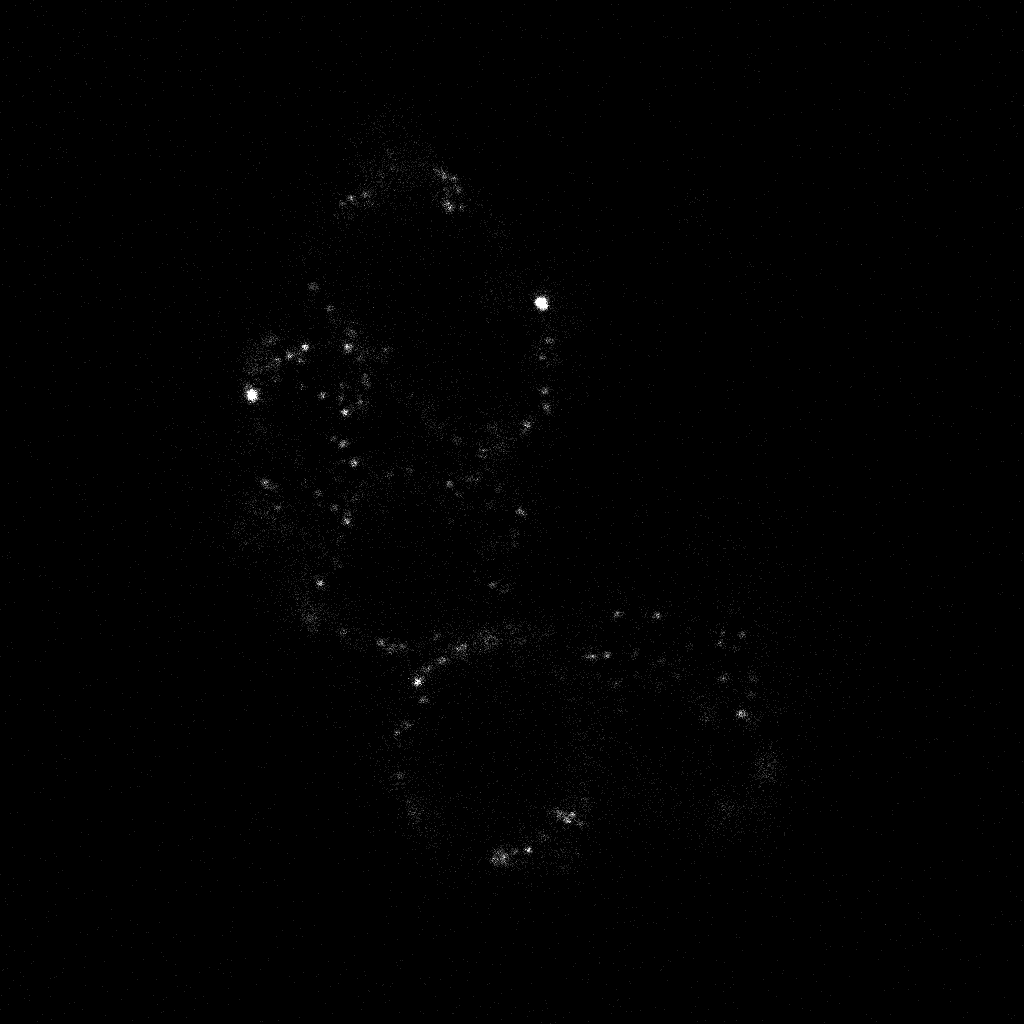

Supplement: Supplementary file 8 — Source data Fig. 1 [file 44319_2025_567_MOESM8_ESM.zip › Fig1/1D/Counted_nuclei/Mechanical/Nuc_10+11+12+13+14/Nuc_10+11+12+13+14_z45_RAW_ch00.tif]

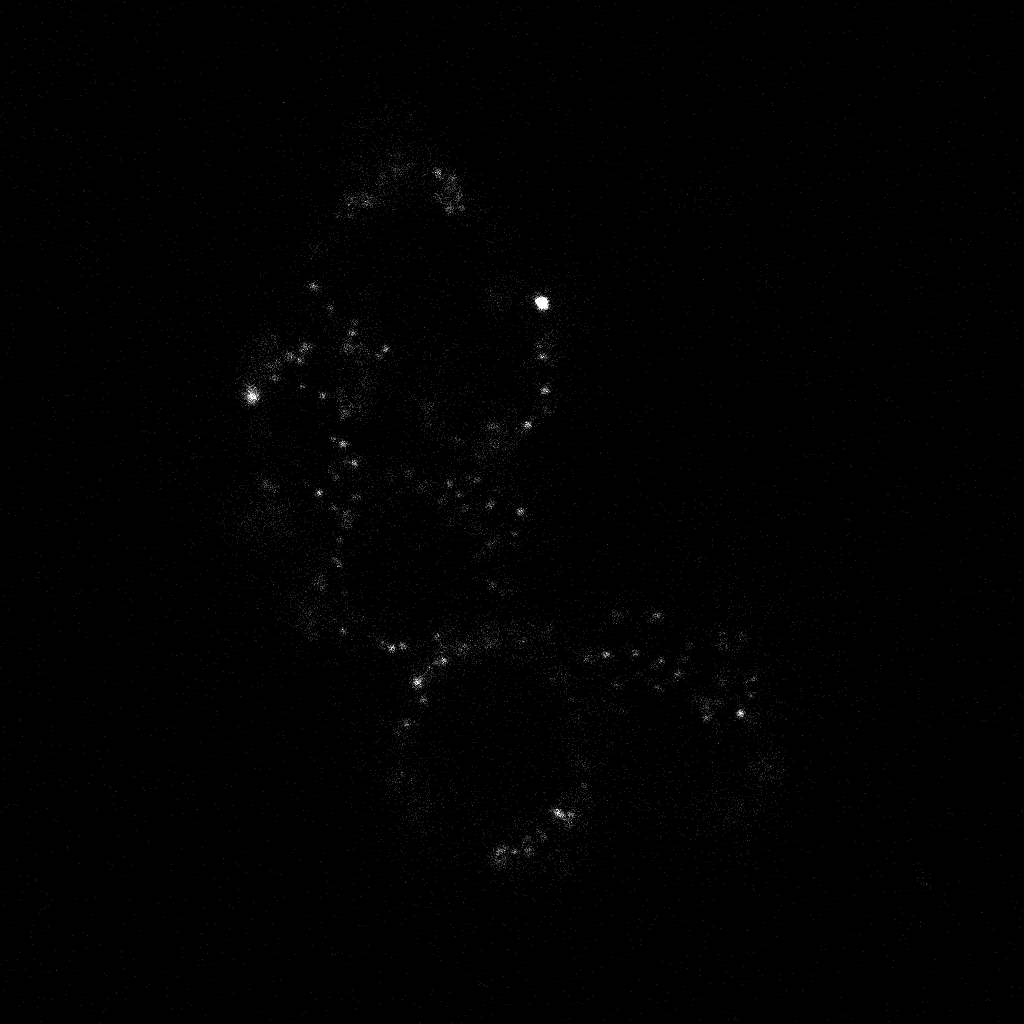

Supplement: Supplementary file 8 — Source data Fig. 1 [file 44319_2025_567_MOESM8_ESM.zip › Fig1/1D/Counted_nuclei/Mechanical/Nuc_10+11+12+13+14/Nuc_10+11+12+13+14_z46_RAW_ch00.tif]

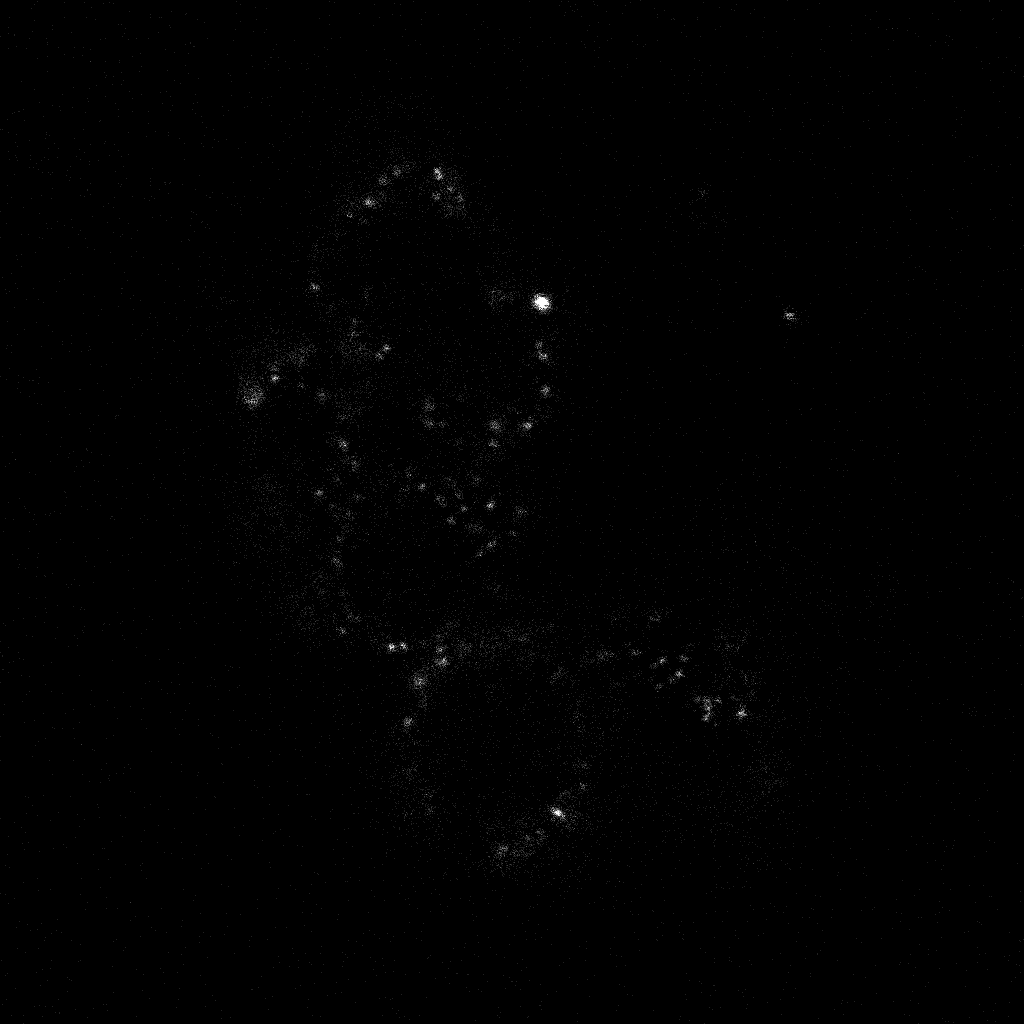

Supplement: Supplementary file 8 — Source data Fig. 1 [file 44319_2025_567_MOESM8_ESM.zip › Fig1/1D/Counted_nuclei/Mechanical/Nuc_10+11+12+13+14/Nuc_10+11+12+13+14_z47_RAW_ch00.tif]

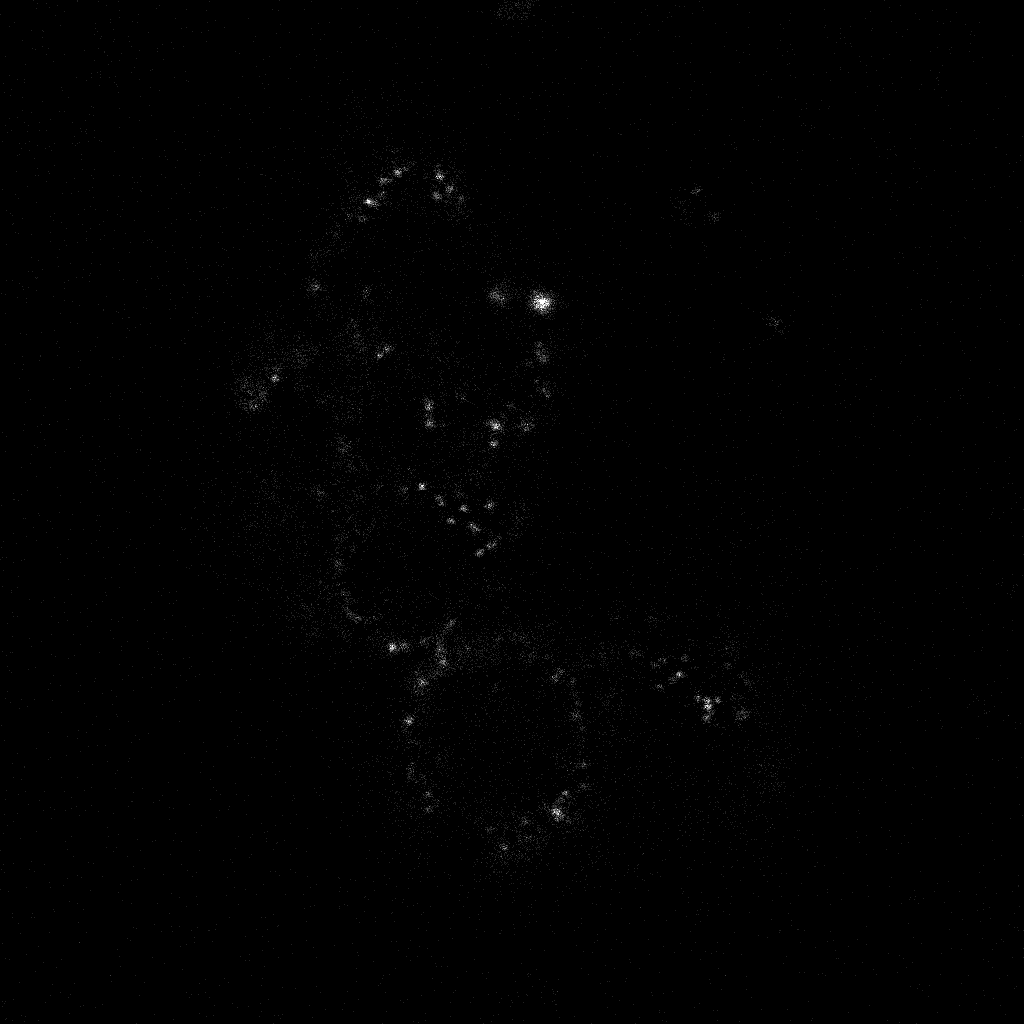

Supplement: Supplementary file 8 — Source data Fig. 1 [file 44319_2025_567_MOESM8_ESM.zip › Fig1/1D/Counted_nuclei/Mechanical/Nuc_10+11+12+13+14/Nuc_10+11+12+13+14_z48_RAW_ch00.tif]

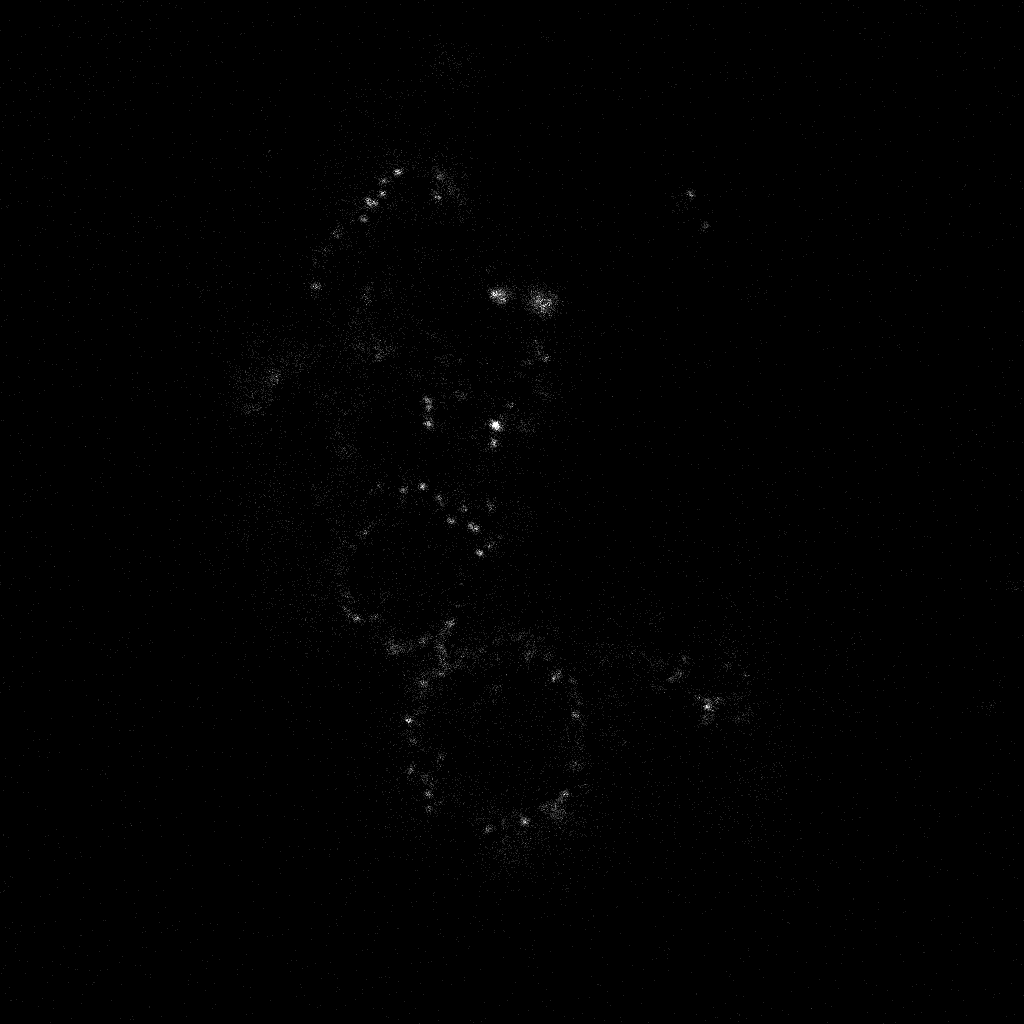

Supplement: Supplementary file 8 — Source data Fig. 1 [file 44319_2025_567_MOESM8_ESM.zip › Fig1/1D/Counted_nuclei/Mechanical/Nuc_10+11+12+13+14/Nuc_10+11+12+13+14_z49_RAW_ch00.tif]

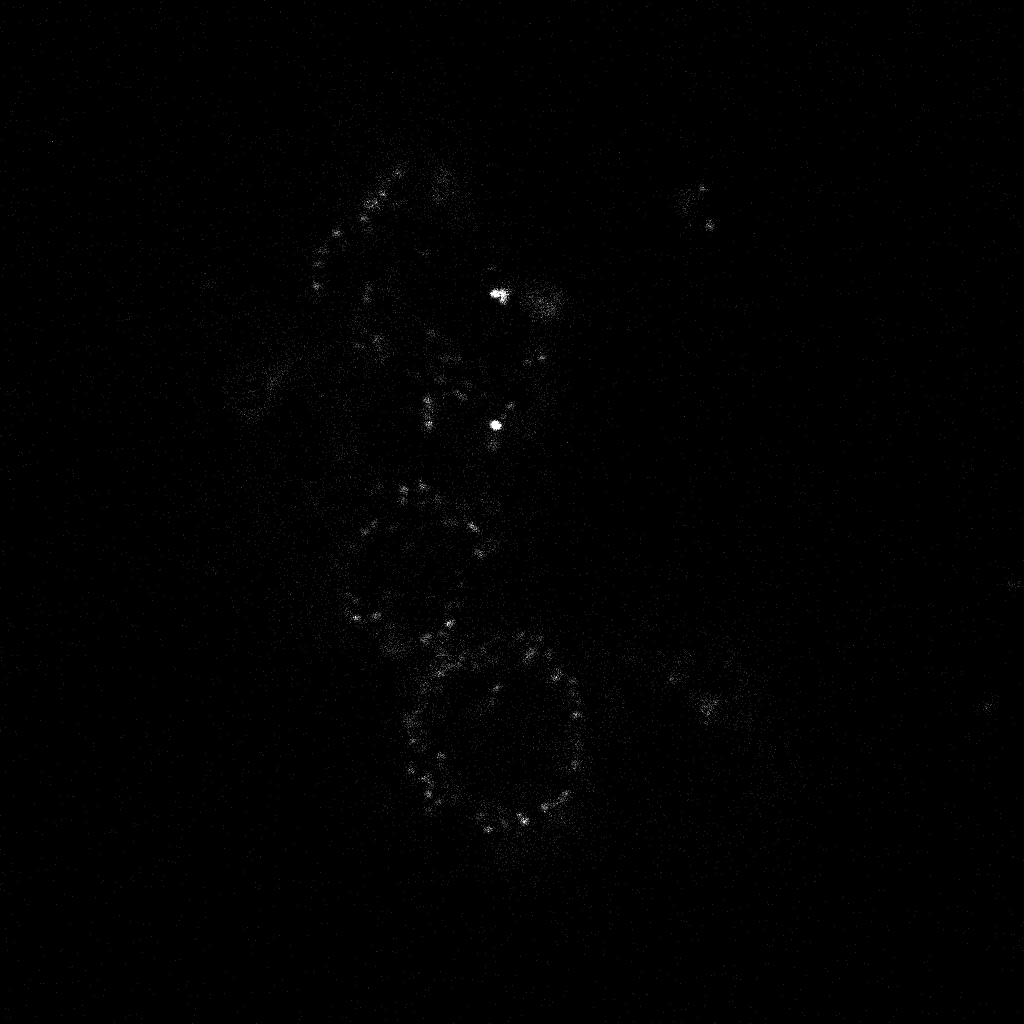

Supplement: Supplementary file 8 — Source data Fig. 1 [file 44319_2025_567_MOESM8_ESM.zip › Fig1/1D/Counted_nuclei/Mechanical/Nuc_10+11+12+13+14/Nuc_10+11+12+13+14_z50_RAW_ch00.tif]

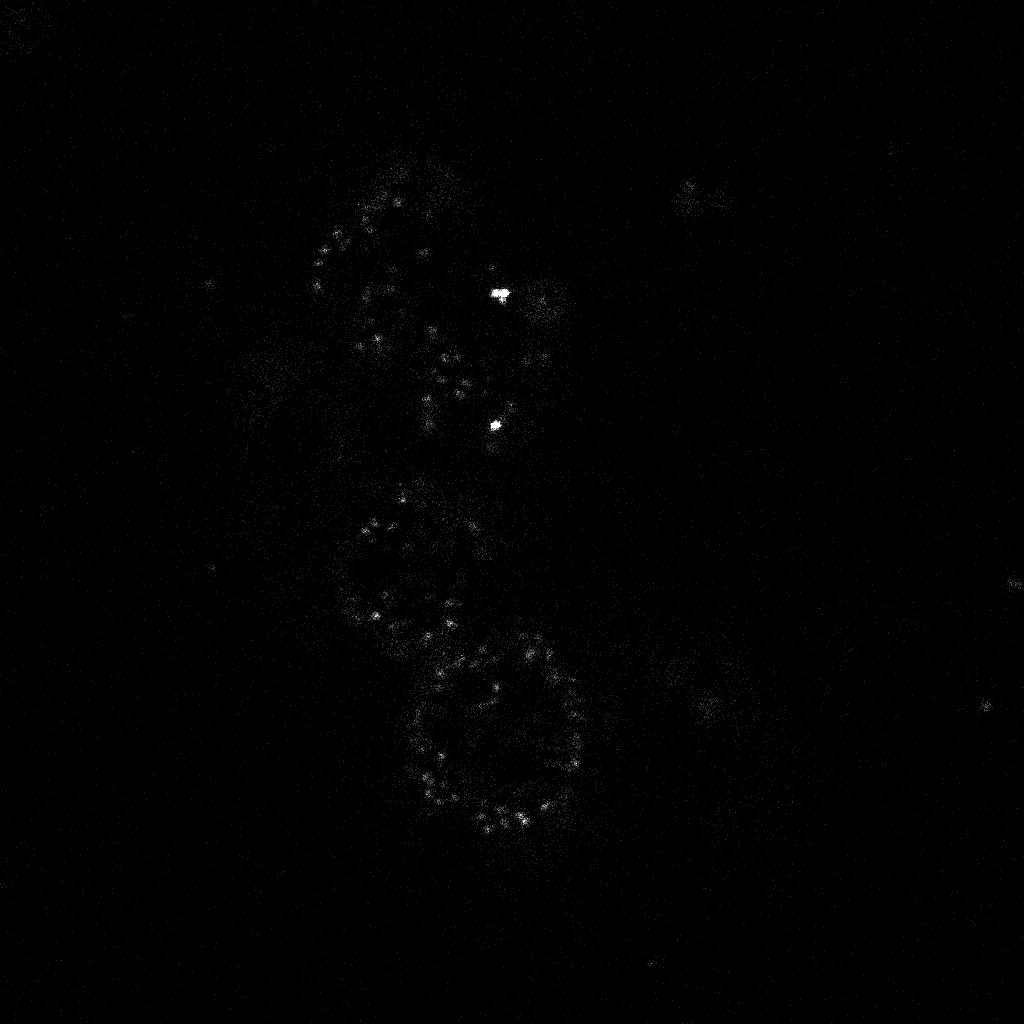

Supplement: Supplementary file 8 — Source data Fig. 1 [file 44319_2025_567_MOESM8_ESM.zip › Fig1/1D/Counted_nuclei/Mechanical/Nuc_10+11+12+13+14/Nuc_10+11+12+13+14_z51_RAW_ch00.tif]

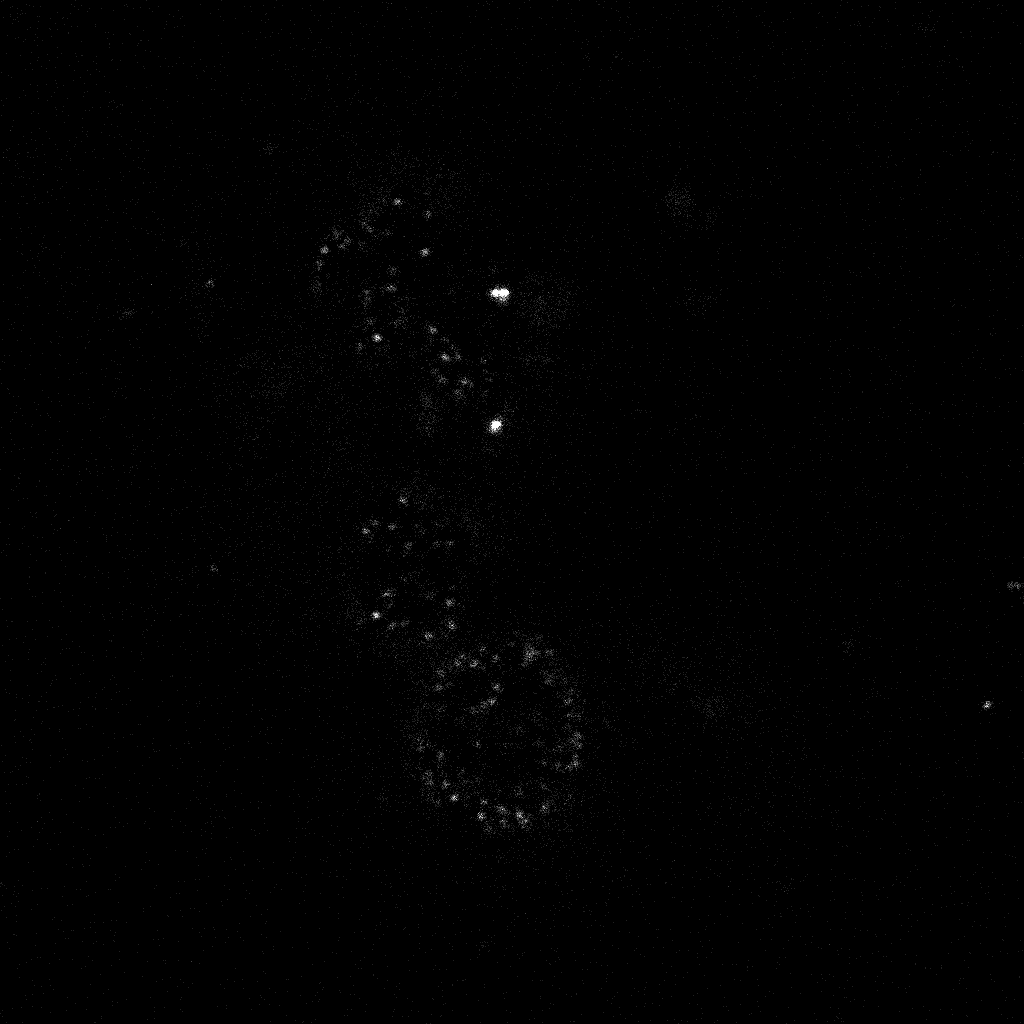

Supplement: Supplementary file 8 — Source data Fig. 1 [file 44319_2025_567_MOESM8_ESM.zip › Fig1/1D/Counted_nuclei/Mechanical/Nuc_10+11+12+13+14/Nuc_10+11+12+13+14_z52_RAW_ch00.tif]

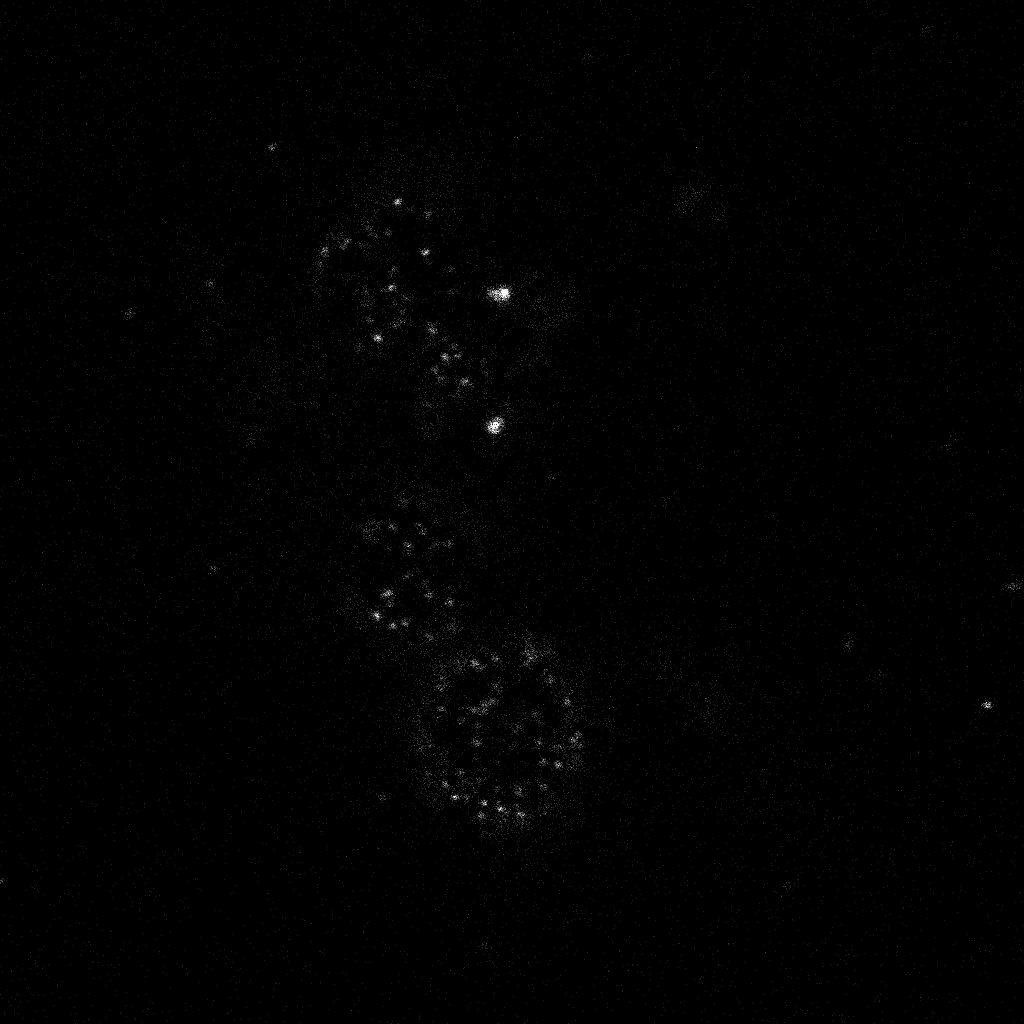

Supplement: Supplementary file 8 — Source data Fig. 1 [file 44319_2025_567_MOESM8_ESM.zip › Fig1/1D/Counted_nuclei/Mechanical/Nuc_10+11+12+13+14/Nuc_10+11+12+13+14_z53_RAW_ch00.tif]

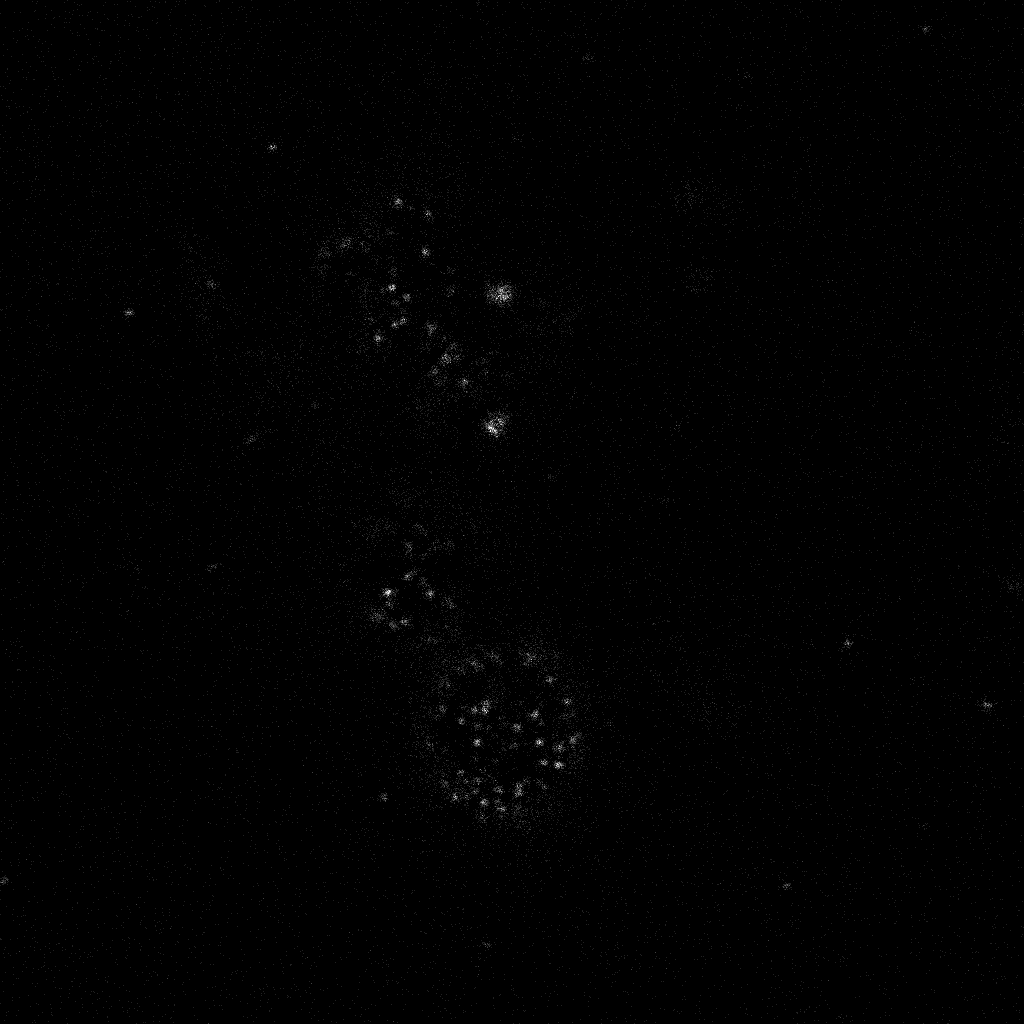

Supplement: Supplementary file 8 — Source data Fig. 1 [file 44319_2025_567_MOESM8_ESM.zip › Fig1/1D/Counted_nuclei/Mechanical/Nuc_10+11+12+13+14/Nuc_10+11+12+13+14_z54_RAW_ch00.tif]

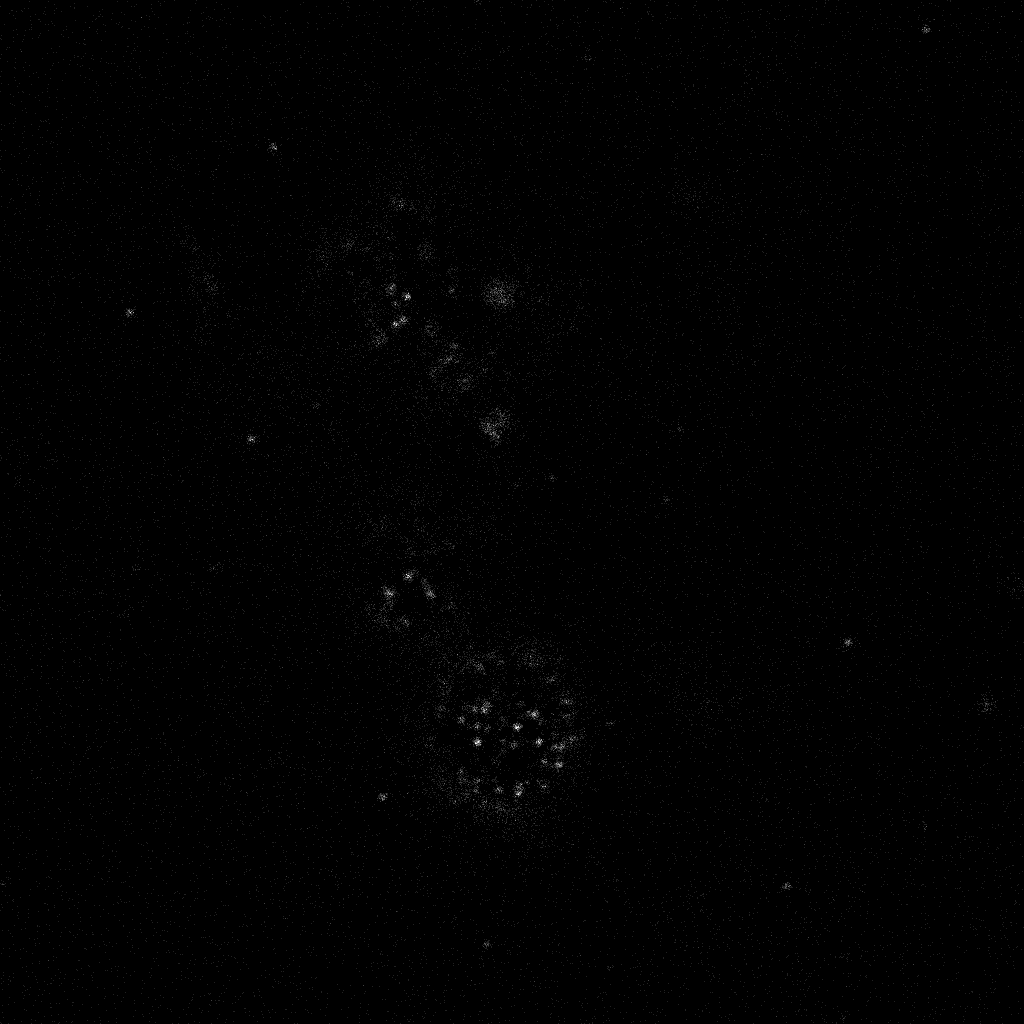

Supplement: Supplementary file 8 — Source data Fig. 1 [file 44319_2025_567_MOESM8_ESM.zip › Fig1/1D/Counted_nuclei/Mechanical/Nuc_10+11+12+13+14/Nuc_10+11+12+13+14_z55_RAW_ch00.tif]

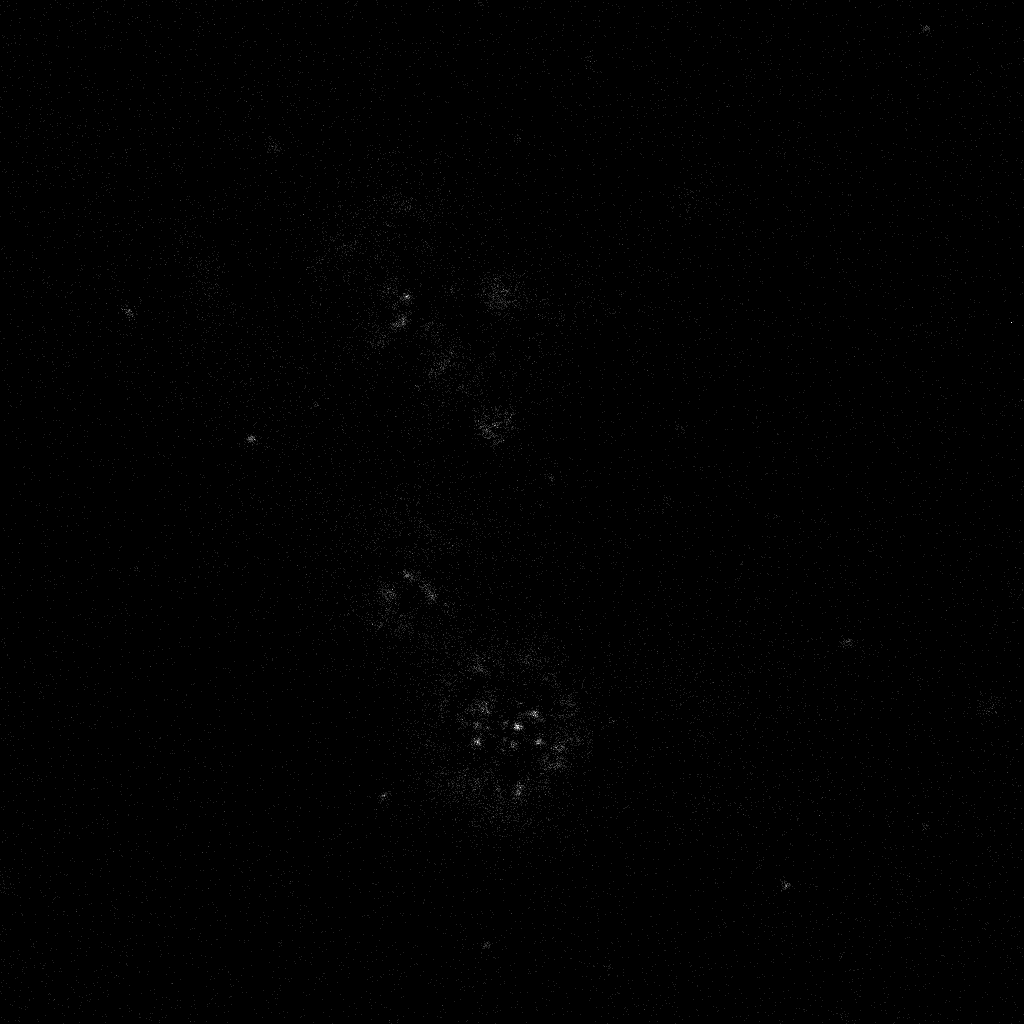

Supplement: Supplementary file 8 — Source data Fig. 1 [file 44319_2025_567_MOESM8_ESM.zip › Fig1/1D/Counted_nuclei/Mechanical/Nuc_10+11+12+13+14/Nuc_10+11+12+13+14_z56_RAW_ch00.tif]

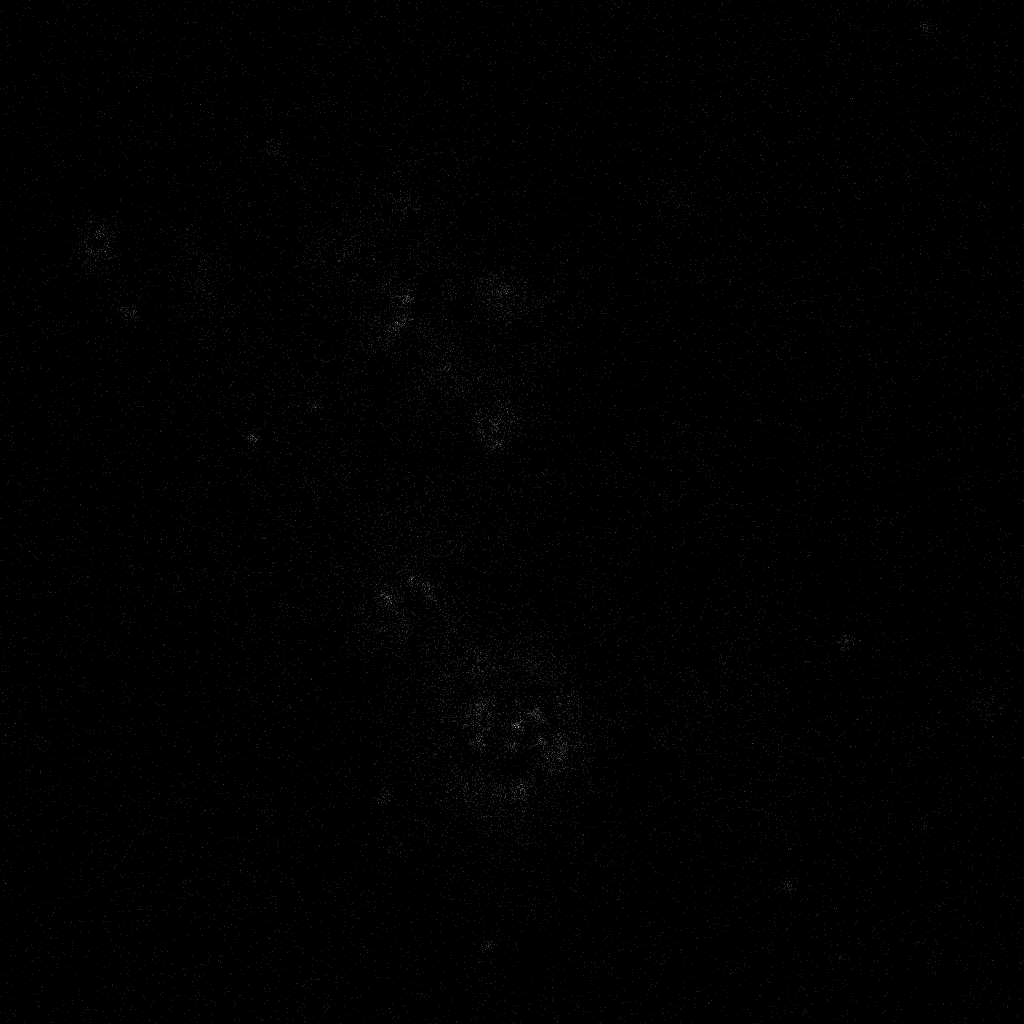

Supplement: Supplementary file 8 — Source data Fig. 1 [file 44319_2025_567_MOESM8_ESM.zip › Fig1/1D/Counted_nuclei/Mechanical/Nuc_10+11+12+13+14/Nuc_10+11+12+13+14_z57_RAW_ch00.tif]

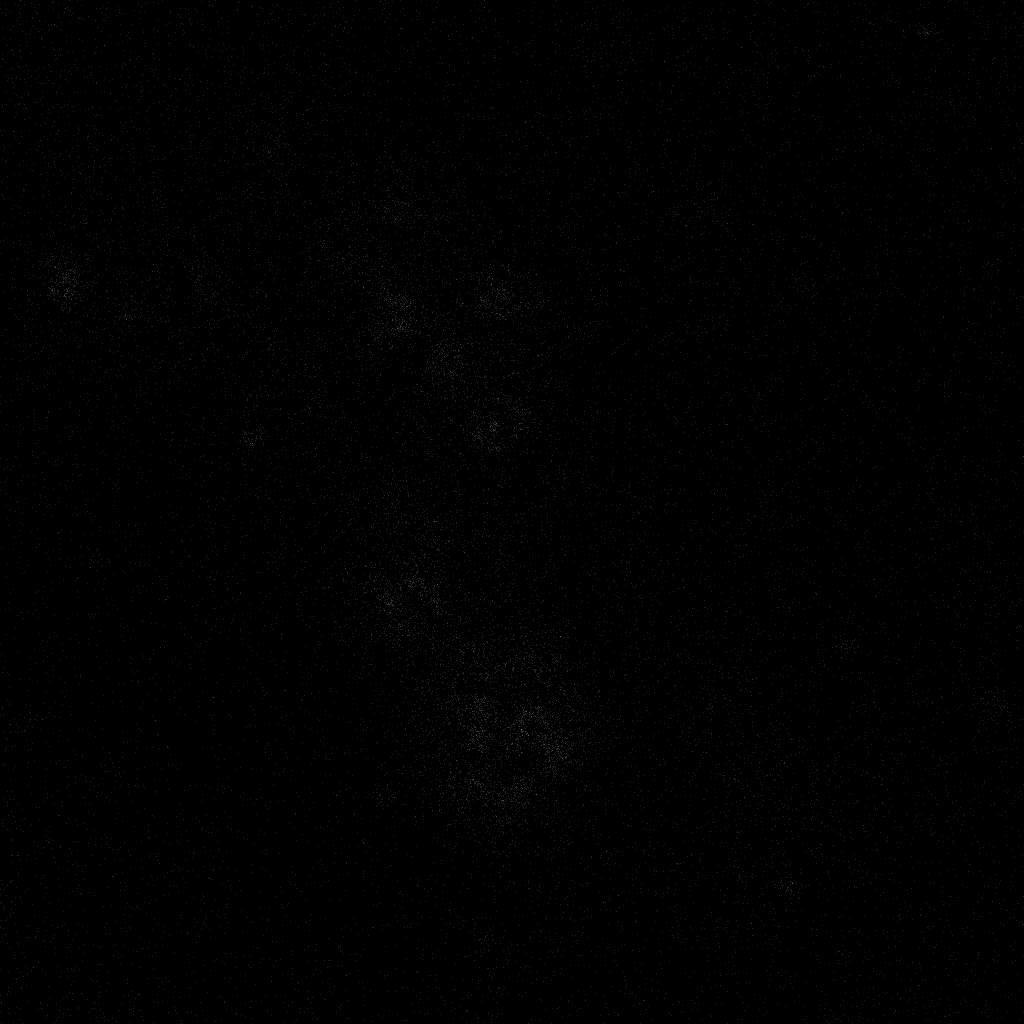

Supplement: Supplementary file 8 — Source data Fig. 1 [file 44319_2025_567_MOESM8_ESM.zip › Fig1/1D/Counted_nuclei/Mechanical/Nuc_10+11+12+13+14/Nuc_10+11+12+13+14_z58_RAW_ch00.tif]
