# Supplementary figures and images for: Direct visualization of HIV-1 core nuclear import and its interplay with the nuclear pore (part 3 of 3)
Source: EMBO Rep. 2025 Aug 29;26(21):5133–53. doi: 10.1038/s44319-025-00567-6 (PMC12592377; doi:10.1038/s44319-025-00567-6)

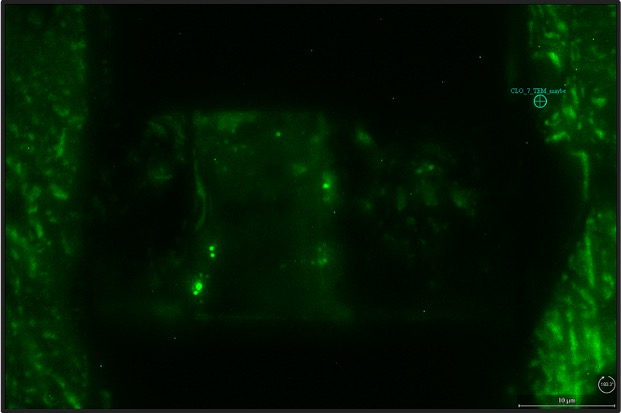

Supplement: Supplementary file 9 — Source data Fig. 2 [file 44319_2025_567_MOESM9_ESM.zip › Fig2/A/iv.jpg]

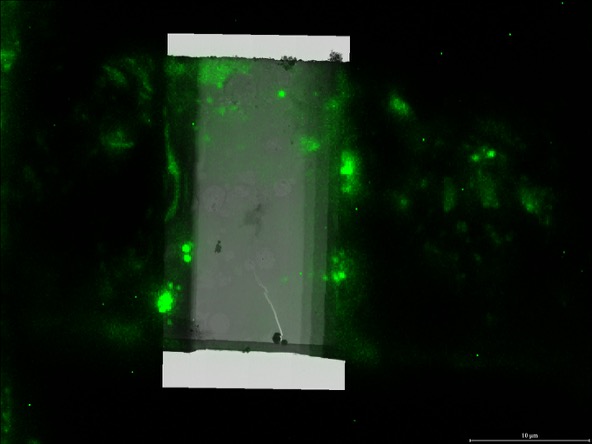

Supplement: Supplementary file 9 — Source data Fig. 2 [file 44319_2025_567_MOESM9_ESM.zip › Fig2/A/v.jpg]

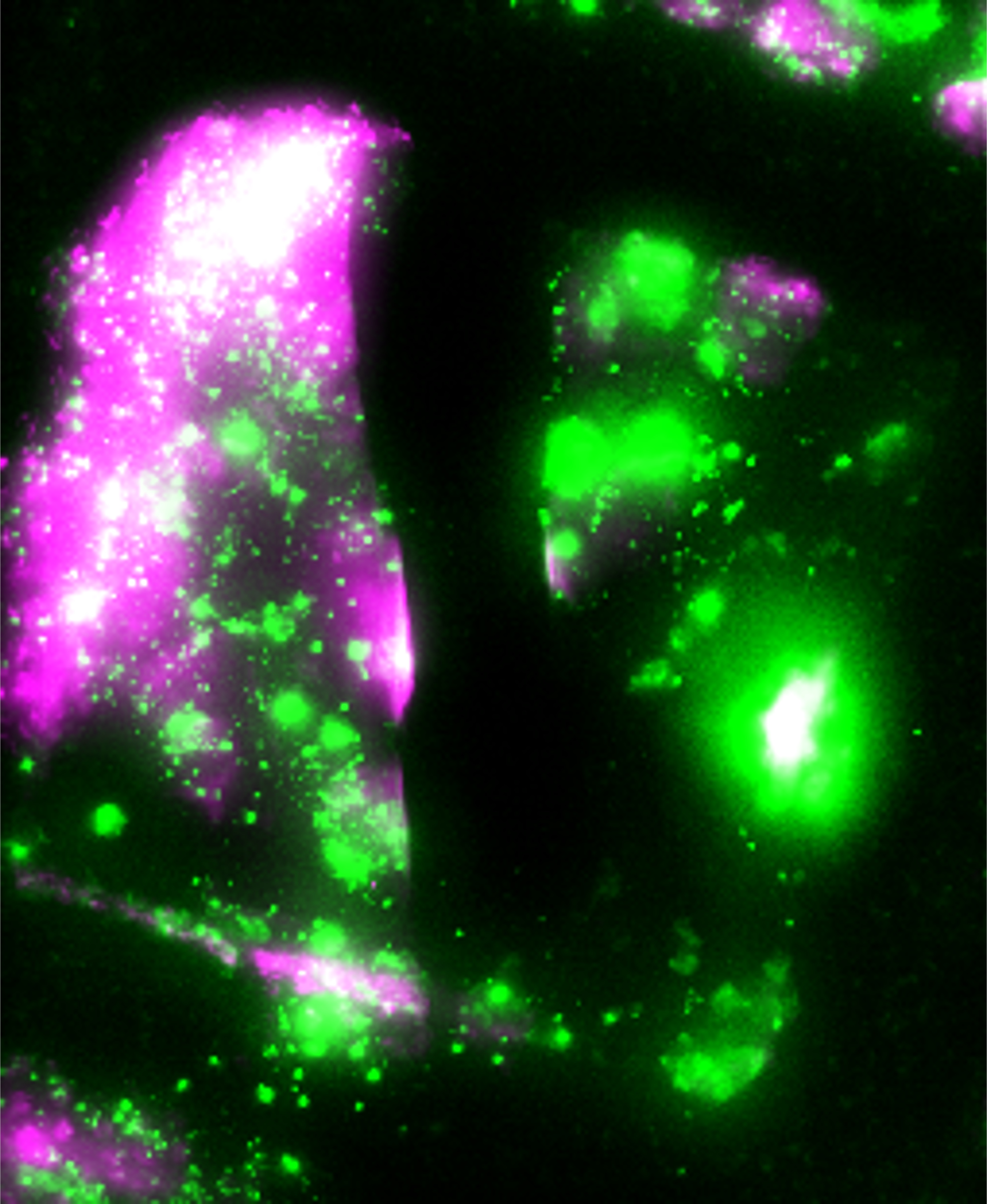

Supplement: Supplementary file 9 — Source data Fig. 2 [file 44319_2025_567_MOESM9_ESM.zip › Fig2/B/i.jpg]

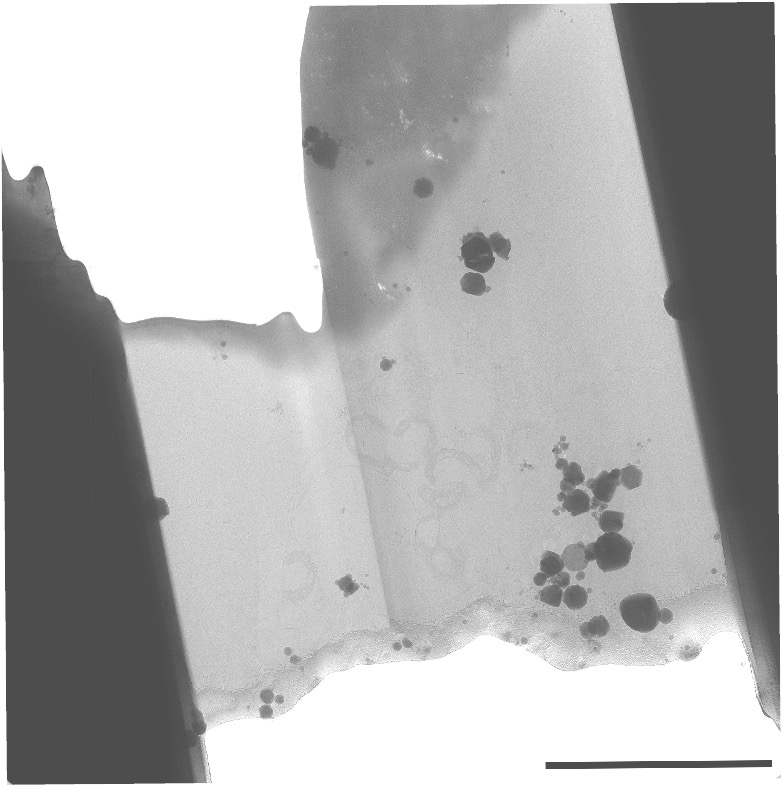

Supplement: Supplementary file 9 — Source data Fig. 2 [file 44319_2025_567_MOESM9_ESM.zip › Fig2/B/ii.jpg]
