# Supplementary material for: Direct visualization of HIV-1 core nuclear import and its interplay with the nuclear pore
Source: EMBO Rep. 2025 Aug 29;26(21):5133–53. doi: 10.1038/s44319-025-00567-6 (PMC12592377; doi:10.1038/s44319-025-00567-6)
Supplement: Supplementary file 13 — Expanded View Figures [file 44319_2025_567_MOESM13_ESM.pdf]

Expanded View Figures

Figure EV1. Characterization of HIV-1 cores, CEM cell permebilization, and core-nuclei association using confocal microscopy and cryo-EM.

(**A**) Representative confocal images of mechanically permeabilized CEM cells, shown in transillumination channel (left), SiR-DNA channel (middle) and merged with SiR-DNA in magenta (right). (**B**) Representative confocal images of nuclei of mechanically permeabilized CEM cells, mixed with 150 kDa FITC-dextran, shown in FITC-dextran channel (left), SiR-DNA channel (middle) and merged with SiR-DNA in magenta and FITC in green (right). (**C**) Two representative cryo-EM images of isolated mature cone-shaped and tube-shaped HIV-1 VLP cores. Inset, mNeonGreen-IN labelled HIV-1 VLP core bands after "spin thru" detergent treatment. The red arrowhead indicates the band extracted for this study. (**D**) A bar chart showing the distribution of cone-shaped and tube-shaped HIV-1 cores observed within virions and in cores purified from VLPs, respectively. Data were collected from a representative cryo-EM grid. (**E**) A violin plot showing the width of HIV-1 cores measured from within virions and from cores purified from VLPs, respectively. The width of cone-shape cores (at the wide end) from virions measures 56.79 ± 4.935 nm (SE = 0.3678, $n = 180$) and that from VLPs measures 56.43 ± 6.199 nm (SE = 0.7573, $n = 67$). The width of tube-shaped cores from virions measures 41.91 ± 5.056 nm (SE = 0.8672, $n = 34$) and that from VLPs measures 40.95 ± 3.314 nm (SE = 0.4143, $n = 64$). Black lines represent the medians, white lines represent the quartiles, and white dots represent individual HIV-1 cores (two-sided t test, ns = no significance). Data were collected from a representative cryo-EM grid. (**F**) Representative confocal images of nuclei from mechanical lysed (left) or detergent-permeabilized (right) T cells, mixed with isolated HIV-1 VLP cores, shown in single slice (top) and MIP (bottom). HIV-1 cores are labeled with mNeonGreen-IN (green) and nuclei labeled with SiR-DNA (magenta). (**G**) A box plot representing the number of mNeonGreen-IN puncta decorating a single nucleus obtained either by mechanical lysis or by digitonin permeabilization of CEM cells. The mechanical lysis condition yielded 150 ± 48 puncta per nucleus (Fig. 1D) based on two biological replicates: replicate 1: 160 ± 52 ($n = 9$) and replicate 2: 136 ± 42 ($n = 7$). The digitonin permeabilization condition yielded 217 ± 77 puncta per nucleus (Fig. 1D) based on two biological replicates: replicate 1: 243 ± 93 ($n = 9$) and replicate 2: 185 ± 32 ($n = 7$). In the box plot, the center line is the median, whiskers are min and max, and box bounds are the 25th and 75th percentiles.

 © The Author(s)

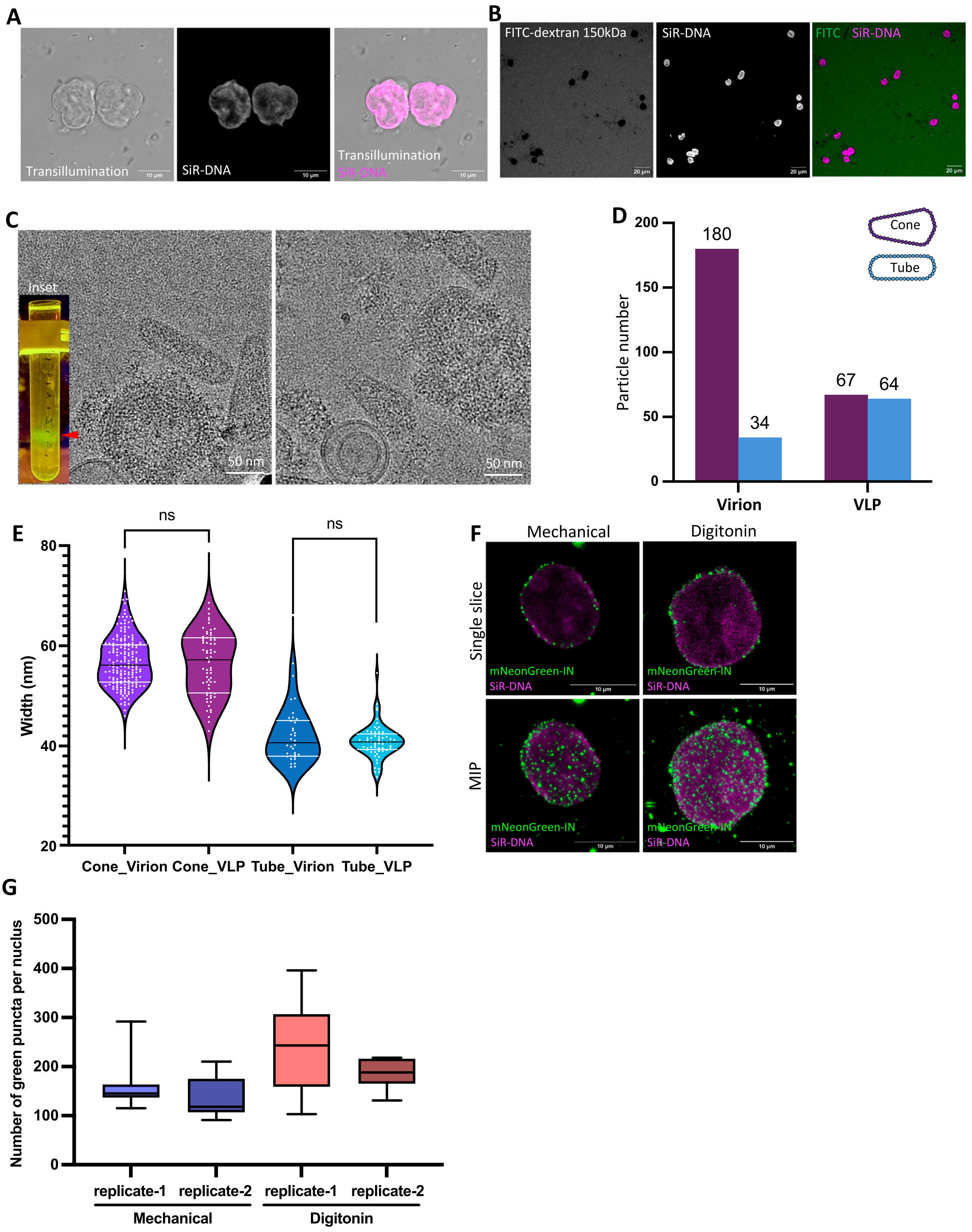
A
Transillumination
SiR-DNA
Transillumination
SiR-DNA
10 μm
B
FITC-dextran 150kDa
SiR-DNA
FITC / SiR-DNA
20 μm
C
Inset
50 nm
50 nm
D
Particle number
180
34
67
64
Cone
Tube
Virion
VLP
200
150
100
50
0
E
Width (nm)
ns
ns
80
60
40
20
Cone_Virion Cone_VLP Tube_Virion Tube_VLP
F
Mechanical
Digitonin
Single slice
MIP
mNeonGreen-IN
SiR-DNA
10 μm
G
Number of green puncta per nucleus
500
400
300
200
100
0
replicate-1 replicate-2 replicate-1 replicate-2
Mechanical
Digitonin

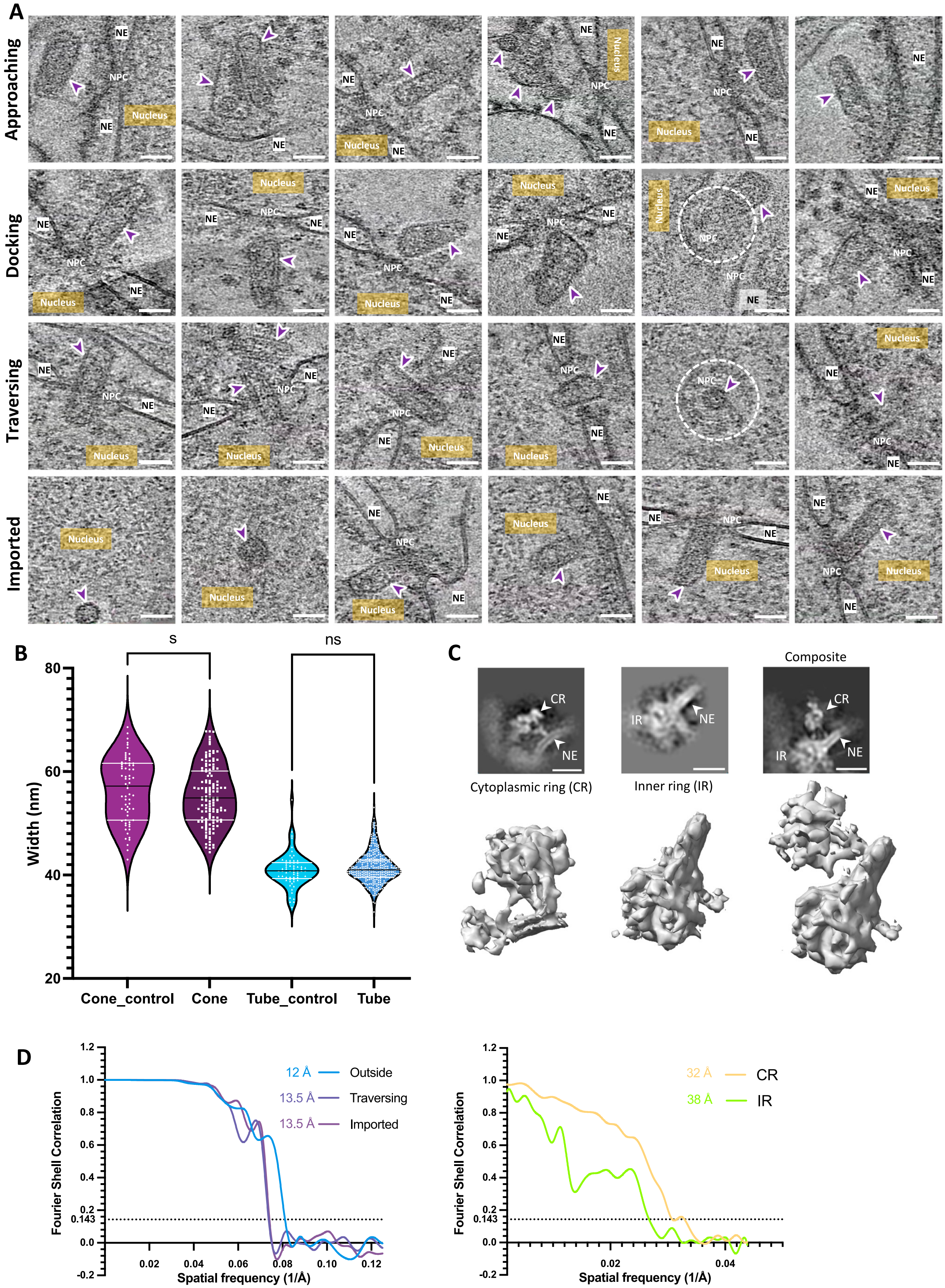
A
Approaching
Docking
Traversing
Imported
NE
NPC
Nucleus
B
s
ns
Width (nm)
80
60
40
20
Cone_control
Cone
Tube_control
Tube
C
Composite
CR
NE
IR
Cytoplasmic ring (CR)
Inner ring (IR)
D
Fourier Shell Correlation
Spatial frequency (1/Å)
12 Å Outside
13.5 Å Traversing
13.5 Å Imported
32 Å CR
38 Å IR
1.2
1.0
0.8
0.6
0.4
0.2
0.143
0.0
-0.2
0.02
0.04
0.06
0.08
0.10
0.12

© The Author(s)

Figure EV2. Structural characterization of HIV-1 cores during nuclear import and NPC ring moieties by cryo-ET.

(**A**) Gallery of HIV-1 cores in multiple states during the nuclear import. Six representative tomographic slices from each state are showcased. HIV-1 cores are indicated by purple arrowheads, the nucleus, NE, and NPC are annotated accordingly. Scale bars = 50 nm. (**B**) A violin plot of the width of HIV-1 cores associated with nuclei and from the input control, for both cone-shaped and tube-shaped cores. The wide end of cone-shaped HIV-1 cores measures 56.43 ± 6.199 nm (SE = 0.7573, $n = 67$) for the input control, and 55.28 ± 5.730 nm (SE = 0.4968, $n = 133$) for the associated with nuclei. The width of tube-shaped HIV-1 cores measures 40.95 ± 3.314 nm (SE = 0.4143, $n = 64$) for the input control, and 41.43 ± 2.994 nm (SE = 0.1638, $n = 334$) for the associated with nuclei. Black lines represent the medians, white lines represent the quartiles, and white dots represent individual HIV-1 cores (two-sided t test, ns = no significance). The images of input control were collected from a representative grid and the HIV-1 cores associated with nuclei were imaged from 12 grids used for cryo-CLEM. (**C**) Structures of NPC ring moieties determined by subtomogram averaging. Maps are aligned and contoured to the same level (3σ). Upper: central slices of the EM maps in the XZ plane; Lower: EM density maps. Cytoplasmic ring, CR; Inner ring, IR. Scale bar, 20 nm. (**D**) Gold-standard Fourier shell correlation (FSC) curves of subtomogram averaged maps. The resolution is indicated at 0.143 FSC cut-off.
